# Non-Coding RNA Prediction and Verification in *Saccharomyces cerevisiae*

**DOI:** 10.1371/journal.pgen.1000321

**Published:** 2009-01-02

**Authors:** Laura A. Kavanaugh, Fred S. Dietrich

**Affiliations:** Department of Molecular Genetics and Microbiology, Institute for Genome Sciences and Policy, Duke University Medical Center, Durham, North Carolina, United States of America; RIKEN Genomic Sciences Center, Japan

## Abstract

Non-coding RNA (ncRNA) play an important and varied role in cellular function. A significant amount of research has been devoted to computational prediction of these genes from genomic sequence, but the ability to do so has remained elusive due to a lack of apparent genomic features. In this work, thermodynamic stability of ncRNA structural elements, as summarized in a Z-score, is used to predict ncRNA in the yeast *Saccharomyces cerevisiae*. This analysis was coupled with comparative genomics to search for ncRNA genes on chromosome six of *S. cerevisiae* and *S. bayanus*. Sets of positive and negative control genes were evaluated to determine the efficacy of thermodynamic stability for discriminating ncRNA from background sequence. The effect of window sizes and step sizes on the sensitivity of ncRNA identification was also explored. Non-coding RNA gene candidates, common to both *S. cerevisiae* and *S. bayanus*, were verified using northern blot analysis, rapid amplification of cDNA ends (RACE), and publicly available cDNA library data. Four ncRNA transcripts are well supported by experimental data (*RUF10*, *RUF11*, *RUF12*, *RUF13*), while one additional putative ncRNA transcript is well supported but the data are not entirely conclusive. Six candidates appear to be structural elements in 5′ or 3′ untranslated regions of annotated protein-coding genes. This work shows that thermodynamic stability, coupled with comparative genomics, can be used to predict ncRNA with significant structural elements.

## Author Summary

Recent advances in DNA sequence technology have made it possible to sequence entire genomes. Once a genome is sequenced, it becomes necessary to identify the set of genes and other functional elements within the genome. This is particularly challenging as much of the genomic sequence does not appear to perform any function and is loosely referred to as “junk.” Identifying functional elements among the “junk” is difficult. Experimental methods have been developed for this purpose but they are time-consuming, expensive, and often provide an incomplete picture. Thus, it is important to develop the ability to identify these functional elements using computational methods. Protein-coding genes are relatively easy to identify computationally, but other categories of functional elements present a significantly greater challenge. In this work, we used a computational approach to identify genes that do not encode for a protein but rather function as an RNA molecule. We then used experimental methods to verify our predictions and thereby validate the computational method.

## Introduction

Non-coding RNA (ncRNA) are functional RNA transcripts that are not translated into protein (i.e., not messenger RNAs). Research, particularly over the last 10 years, has shown that they perform a wide range of functions in the cell [1]–[4]. Despite the growing body of knowledge about ncRNA, it is likely that many ncRNA remain undiscovered. Data from high-throughput experimental methods show that much of the intergenic DNA in eukaryotic genomes is transcribed and may be ncRNA [5]–[10]. Even in *Saccharomyces cerevisiae*, one of the most thoroughly studied model organisms, there is evidence that only a fraction of the ncRNA is known. Tiling arrays, large-scale cDNA libraries, and serial analysis of gene expression (SAGE) experiments have all shown transcription from many locations in the genome that appear to be unannotated ncRNA genes [11]–[14]. This along with recent identification of new protein coding genes such as YPR010C-A in 2006 shows that even in this best-studied Eukaryote, we still do not know the complete gene set [13].

Computational methods for accurate ncRNA gene prediction remain elusive. The development of such methods are crucial for identifying ncRNA that are difficult to detect experimentally such as those expressed at low levels or under unusual conditions. They are also needed to reduce the time and expense required to perform experimental methods, particularly when considering the large number of species of interest. The challenge of predicting ncRNA genes rests with the fact that they lack common primary sequence features and demonstrate poor cross-species sequence conservation [15],[16]. They do not have start codons, stop codons or open reading frames which serve as key signposts for protein-coding genes and cannot be located using simple sequence searches.

Some success with ncRNA gene prediction has been achieved by focusing on specific sub-classes of ncRNA that share common features. Examples include tRNAs, tmRNAs, snoRNAs (C/D box and H/ACA box), and miRNAs [17]–[32]. In *S. cerevisiae*, computational screens for C/D box [19] and H/ACA box snoRNAs [20] have identified several new snoRNA genes.

Additional ncRNA screens in *S. cerevisiae* have included searches for polymerase III promoters, searches in larger than average intergenic regions [33] and searches for ncRNA structural features using the QRNA program. The QRNA program was used to search pair-wise alignments for patterns of compensatory mutations consistent with base-paired secondary structure [34]. These regions were then tested experimentally to determine if they expressed a transcript likely to be ncRNA. Together, these three methods resulted in identification of 6 novel ncRNA that were supported by experimental evidence (*RNA170*, *snR161*, *snR82*, *snR83*, *snR84*, *RUF5-1/2*). In another study, the *S. cerevisiae* genome was analyzed using the RNAZ program [35]. This program is based on the same principals as the QRNA program and uses multiple, cross-species sequence alignments to search for patterns of compensatory changes suggestive of secondary structure. RNAZ also includes thermodynamic analysis. A total of 572 candidate regions were identified as potentially containing unannotated ncRNA candidates using the RNAZ program [35],[36]. Publicly available data sets were used to provide general support for these predictions but no detailed experimental analysis was performed on individual predictions.

In this work ncRNA genes are predicted in *S. cerevisiae* based solely on the thermodynamic stability of ncRNA structures as proposal by Maizel in the late 1980's [37]–[39]. Maizel theorized that structural ncRNA are thermodynamically more stable than random sequences. An influential paper by Rivas & Eddy entitled “Secondary structure alone is generally not statistically significant for the detection of noncoding RNAs” suggested that Maizel's approach was generally not effective for structural ncRNA discovery [40]. Based on this conclusion, many investigators turned away from thermodynamic based approaches for ncRNA discovery to methods based on compensatory changes in cross-species alignments[31]. However, a growing body of evidence has been accumulating suggesting that thermodynamic stability is a discriminating feature of many classes of structural ncRNA [41]–[43]. In this work, we build on this result to not only evaluate the thermodynamic stability of known structural ncRNA but also to use it for structural ncRNA discovery.

The work presented here demonstrates the value of thermodynamic structural stability, as summarized in a Z-score, for discovery of structural ncRNA. It also explores the impact of window size and step size on the sensitivity of ncRNA identification. Sets of positive and negative control genes were evaluated to determine the effectiveness of the approach. This approach was then applied to predict ncRNA genes on chromosome six of *S. cerevisiae*. The analysis was repeated independently in *S. bayanus* and the gene predictions common to both genomes comprised the final set of gene predictions. Experimental validation of these predictions show that four ncRNA transcripts are well supported by northern blot analysis, rapid amplification of cDNA ends (RACE), and publicly available cDNA data. One additional ncRNA candidate is also supported by experimental data but the data is not entirely conclusive. Six of the predicted candidates appear to be structural elements in 5′ or 3′ untranslated regions (UTRs) of annotated protein-coding genes.

## Results

### General Approach

The thermodynamic stability of potential ncRNA candidates was evaluated using a Z-score based on the minimum folding energy (MFE) determined by RNAfold [44]. The Z-score represents the number of standard deviations that the MFE of a native sequence, **x**, deviates from the mean MFE of a set of shuffled sequences of **x** (see Materials and Methods).

A key variable in calculating the Z-score for ncRNA discovery (as opposed to evaluating known structural ncRNA) is the length of the sequence to be evaluated. As ncRNA vary in length and structure, no single window size is expected to be optimal for ncRNA gene identification. Short structural elements will probably only be detected with relatively short window sizes while longer structural elements will probably only be detected with relatively longer window sizes. To identify the window sizes most appropriate for ncRNA discovery, values ranging from 20 nt to 200 nt were investigated and incremented in steps of 5 nt (window delta).

A scanning approach was used to computationally search for potential structural elements within a test sequence. A starting minimum window size was selected and this window was used to scan the test sequence starting at the beginning of the sequence and moving each time by the amount of the step size (our analysis used a step size of 5 nt). A Z-score was calculated for each window position. Once the entire test sequence was evaluated using this fixed window length, a new window length was selected by increasing window length by the amount of the window delta (our analysis used a window delta of 5 nt). The test sequence was evaluated in the same manner using the new window size. This process was repeated until all window sizes had been evaluated.

Since the same test sequence was evaluated using multiple window sizes, it was necessary to determine the impact of multiple hypothesis testing. In lieu of a Bonferroni correction, negative control sets were evaluated using the same number of window sizes and step sizes.

Any windows producing a “significant” Z-score during the scanning process were considered candidate regions for structural ncRNA. The Z-score cutoff considered to be “significant” was determined by evaluating positive and negative test sets. It was sometimes the case that multiple, overlapping windows, of several lengths, produced “significant” Z-scores. In such cases, the region encompassed by all the overlapping windows constituted the candidate region.

Once candidate regions were identified, primers were designed within these regions to determine whether they produced a transcript and to identify the transcript boundaries. The primers were designed as close as possible to the middle of the candidate regions. The exact position of the primer was dictated by the need to satisfy the fairly stringent requirements of the rapid amplification of cDNA ends (RACE) procedure (See Materials and Methods).

### Positive and Negative Control Sets

Positive and negative control sets were compiled to test if the Z-score could be used to distinguish known ncRNA from non-functional sequences as suggested by previous investigators [41]–[43]. The positive control set was drawn from the list of annotated ncRNA in the *Saccharomyces* Genome Database (SGD) [45] (Table 1). The tRNA and rRNA genes were not included in the positive control set as they can be identified with great accuracy using existing tools [17] and because tRNA are known to produce poor thermodynamic footprints [40],[41],[46]. The positive control set consisted of four snoRNA genes and all of the remaining known ncRNA (Table 2).

**Table 1:** Summary of all known nuclear encoded ncRNA in *S. cerevisiae*.

Non-coding RNA	Number	Comments
tRNA	275	Spread across genome
snoRNA (H/ACA box)	29	Spread across genome
snoRNA (C/D box)	47	Spread across genome
rRNA	2	Chr XII, 40–140 tandem repeats
snRNA	5	*LSR1*, *snR14*, *snR19*, *snR6*, *snR7-Long/short*
other	7	*NME1*, *RNA170*, *RPR1*, *RUF5-1/2*, *SCR1*, *SRG1*, *TLC1*

**Table 2:** Positive control set.

*S. cerevisiae* ncRNA	Description	%GC	Length
*SNR6*	mRNA splicing (U6)	39.29	112
*SNR7-L*	mRNA splicing (U5)	44.39	214
*SNR14*	mRNA splicing (U4)	38.75	160
*SNR19*	mRNA splicing (U1)	39.79	568
*LSR1*	mRNA splicing (U2)	40.85	1174
*RPR1*	tRNA cleavage (RNase P component)	51.49	369
*NME1*	Pre-rRNA cleavage (RNase MRP component)	38.94	339
*SRG1*	Regulates SER3	35.39	550
*RNA170*	Unknown function, RNA Pol III transcript	45.56	168
*RUF5-1*	Unknown function	34.08	709
*SCR1*	Cytoplasmic RNA	54.98	521
*TLC1*	Telomerase template	35.59	1300
*SNR76*	C/D box snoRNA	47.71	108
*SNR49*	H/ACA box snoRNA	33.94	164
*SNR83*	H/ACA box snoRNA (*RUF3*)	35.62	305
*SNR30*	H/ACA box snoRNA	46.76	600

Test genes selected to form the positive control set.

Three negative control sets were created to cover the full range of negative control cases. The first negative control set consisted of 20 randomly generated sequences of 300 nt in length. This set was used because it was known not to contain any unannotated genes. The shortcoming of this control set is that it likely fails to capture the nuances of nucleotide distributions in *S. cerevisiae*. The randomly generated sequences had a GC content of ∼40%, ranging from 35.0% to 49.3%, reflecting the GC content of *S. cerevisiae*. A second negative control set was created by randomly shuffling the positive control set. Each sequence was shuffled preserving sequence length as well as its mono- and di-nucleotide composition using the “squid” utilities [47]. The third negative control set was generated by selecting six intergenic regions from the *S. cerevisiae* genome. Intergenic regions were chosen as a control instead of coding regions because the GC content in the *S. cerevisiae* genome differs between protein coding regions and non-protein coding regions. Since the ultimate goal was to search for ncRNA in intergenic regions, it was best to select a test set representative of these regions. The untranslated regions (UTR) of most genes in *S. cerevisiae* are not mapped so the actual intergenic regions are generally unknown. In order to minimize the possibility of choosing a region that contained an unannotated structural element, six intergenic regions were chosen that are flanked on one side by a gene with a known, short (<40 nt) 5′ UTR, unlikely to form a structure. A window of 300 nts from the 5′ end of the open reading frame (ORF) of each of these genes was used as a negative control test sequence (Table S1).

### Positive and Negative Control Set Evaluation

Z-score values calculated for the 20 randomly generated negative control sequences revealed that large negative Z-scores are often generated when using window sizes of less than 65 nt. With these short window sizes, many shuffled sequences have a calculated minimum folding energy of zero or close to zero and the Z-score distribution of the shuffled sequences is narrow. This produces a small value for the standard deviation. If the MFE of the original, unshuffled sequence is even slightly above zero, it will be many standard deviations from the distribution mean and produce a large negative Z-score. When examining window sizes of 75 nt or greater, two (Random9 and Random13) of the 20 randomly generated sequences produced a Z-score less than −3.5 (Table S2, Figures S1 and S2). The total length of sequence producing a Z-score ≤−3.5 was 295 nt and represented 5.0% of the nucleotides in the entire randomly shuffled test set (Table 3).

**Table 3:** Percent of positive and negative control set with Z-score ≤−3.5.

Control set	Description	Total length evaluated	Total length of sequence with Z-score <−3.5	% length with Z-score <−3.5
**Negative Controls**	**Randomly shuffled**	6000	295	5.0
	**Intergenic**	1800	190	10.5
	**Shuffled Positive Controls**	7361	599	8.1
	**Overall**	15161	1084	7.2
**Positive Controls**	**Known ncRNA**	7361	3019	41.0

All numbers given are length (nt).

Z-score values calculated for the 6 intergenic sequences of the second negative control set produced a pattern very similar to that of the randomly generated sequences. For window sizes less than about 65 nt, large negative Z-scores were generated. Window sizes longer than 75 nt did not produce any Z-scores less than −3.5 with the exception of the intergenic sequence between genes *PTP1* and *SSB1*. The first 190 nt of this sequence produced Z-scores as low as −4.7 for various window sizes (Table S2). This may represent either a false positive or may suggest the presence of a structural feature (ncRNA or long *PTP1* 5′ UTR structure). This 190 nt region represents approximately 10.5% of the total length of the intergenic negative control set.

The final negative control set consisted of shuffled sequences of the positive control set (Table 2). Of these, portions of 5 out of 16 sequences (31%) produced Z-scores less than −3.5 (Table S2 and Figures S3 and S4). The total sequence length included in these regions represented 8.1% of the total negative control set length.

All of the sequences in the positive control set produced Z-scores less than −3.5 for multiple window sizes (Table S3, Figures S5 and S6) with the exception of three genes. These genes were *snR76*, *RNA170*, and *SRG1*.

The *snR76* gene is a C/D box snoRNA and it is questionable whether structure plays a significant role in the function of this gene. The SnoScan program was written explicitly to predict C/D box snoRNA and has been used successfully to predict these genes in both *D. melanogaster* and *S. cerevisiae* [19],[48]. Known C/D box snoRNA were used to identify features shared among this family of ncRNA. Only one of the six criteria identified is related to structure (terminal stem base pairings). This base pairing consists of only 4–8 bps and is not always present [19]. This is in stark contrast to the snoGPS program used to identify H/ACA snoRNA [20]. The snoGPS program was trained using known H/ACA snoRNA examples and includes secondary structure as a key element in H/ACA box snoRNA detection. Results from these snoRNA gene identification efforts strongly suggest that structure is generally not a significant component of C/D box snoRNA genes.

*SRG1* is a ncRNA gene that has been shown to repress the expression of its neighboring gene *SER3* [49]. Transcription of *SRG1* interferes with the binding of *SER3* activators in its promoter. This mechanism suggests that *SRG1* fulfills its role as a transcriptional repressor through its transcription rather than through a significant structural component.

The *RNA170* gene was discovered through a genome-wide search of Polymerase III box A and B consensus sequences [33]. Its function and mechanism of action are unknown. It seems likely that this ncRNA does not require a significant structural component to perform its function.

The total sequence length encompassed by a Z-score less than −3.5 in the positive control set represented 41% of the total sequence evaluated. If snR76, SER3 and RNA170 are removed from the set, 46% of the positive control set produces a Z-score <−3.5 (Table 3). Window sizes of 75 nt to 85 nt were crucial for identifying the short ncRNA such as snR6.

To summarize, three negative control sets were used consisting of a set of randomly generated sequences, a set of intergenic sequences, and a set of shuffled positive controls. The percent of sequence producing a false positive indication (i.e., Z-score ≤−3.5) for each of these sets was 5.0%, 10.5%, and 8.1%, respectively (Table 3). We examined the regions producing Z-scores ≤−3.5 for unusual GC content that might explain the large negative Z-score but found nothing significant in these regions (Table S4). For the positive control set, 13 of the 16 genes produced a Z-score ≤−3.5, encompassing 41% of the total sequence length of the set (Table 3). There is good reason to think that the three genes in this set failing to produce a Z-score ≤−3.5 do not contain structural features.

Analysis of the positive and negative control sets provided the following conclusions, (1) Evaluating window sizes less than 65 nt produces many false positives, (2) A Z-score value of −3.5 is useful for discriminating known ncRNA from non-functional sequence, (3) The percent of false positive sequence was observed to be ∼5.0–10.5% when using a cut-off Z-score value of −3.5.

### Identifying ncRNA in Background Sequence

Evaluation of the positive and negative control sets showed that the Z-score was useful for discriminating known structural ncRNA from non-functional sequence. To apply the approach to *de novo* gene prediction it is necessary to scan through a large test sequence (i.e., a chromosome) in search of regions that produce Z-score values indicative of structural ncRNA. To test the effectiveness of our approach for ncRNA discovery, and to determine the optimal parameters for the search, we performed two tests. We evaluated our ability to detect known ncRNA (Table 1), then we performed a detailed analysis of optimal search parameters using a small subset of ncRNA.

First, each annotated, nuclear encoded ncRNA (excluding rRNA), along with 200 nt upstream and downstream of the gene, was used as a test sequence. Z-scores were calculated on the ncRNA strand using the following parameters: window sizes = 75 to 200 nt, step size = 5 nt, window delta = 5 nt. The known ncRNA were considered detected if the center of the window(s) producing a Z-score ≤−3.5 overlapped the gene.

100% of the snRNA were detected, 72.4% of the H/ACA box snoRNA were detected, and 23.9% of the C/D box snoRNA genes were detected. Only 16% of the tRNA genes were detected. This result is consistent with previous reports of poor detection of tRNA based on a Z-score-type search criteria [40],[41],[43]. Clote et al [41] suggested that this may, in part, be due to the extensive post-transcriptional modifications that occur to tRNA that are not accounted for in the MFE calculation based on unmodified sequence. The percent of tRNA detected was a function of the tRNA length. 10.4% of the tRNA shorter than 75 nt (192 total) were detected while 34.6% of tRNA greater than 75 nt (83 total) were detected.

This ncRNA data can also be used to show the impact of using a single window size or a large step size on ncRNA detection (Table 4). The table provides the percent of H/ACA box snoRNAs detected when only a single window size was used to perform the analysis. The impact of using different step sizes (5 nt, 25 nt and 50 nt) is also presented. Using a single window size, as opposed to several sizes, reduces the number of snoRNA detected. The number of H/ACA snoRNA detected by evaluating all window sizes from 75 nt to 200 nt was 72.4%, which is greater than the number detected by using any single window size. The number of H/ACA snoRNA detected for a given window size decreases as the step size increases. These results can provide guidance for choosing a subset of window sizes to perform a ncRNA screen. Tradeoffs can be made between the percent of ncRNA detected and the computational investment required to perform the analysis.

**Table 4:** Effect of using a fixed window size with different step sizes.

	Single Fixed Window Size												
step size	80	90	100	110	120	130	140	150	160	170	180	190	200
**5**	62.1	44.8	51.7	51.7	51.7	51.7	41.4	48.3	48.3	41.4	44.8	44.8	41.4
**25**	34.5	20.7	31.0	27.6	37.9	34.5	34.5	20.7	31.0	17.2	31.0	27.6	27.6
**50**	13.8	6.9	17.2	17.2	31.0	17.2	17.2	17.2	17.2	13.8	24.1	20.7	20.7

The ability to identify the H/ACA snoRNA in background sequence was evaluated using a variety of fixed window sizes and step sizes. The percent of H/ACA snoRNA identified using a fixed window size and step size is provided for window sizes from 80 to 200 and step sizes of 5, 25 and 50. There are a total of 29 known H/ACA snoRNA. The percent of snoRNA detected for a given window size drops with increasing step size. The number of H/ACA snoRNA detected using all of the window sizes with a step size of 5 was 72.4%.

**Figure 1:**
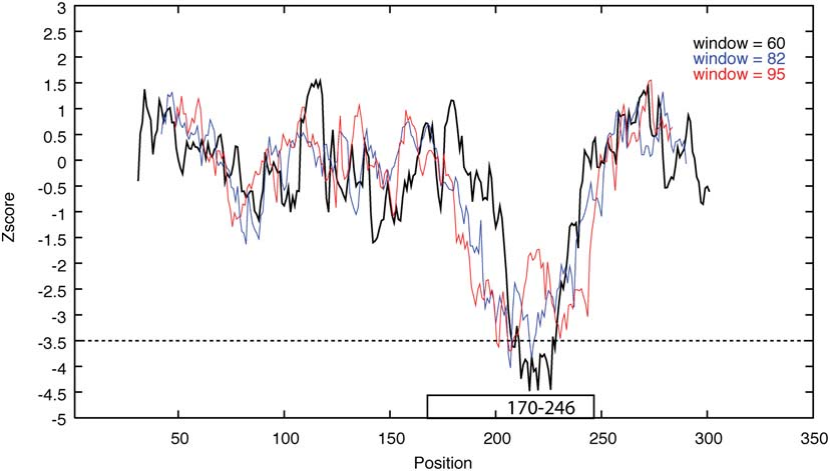
Z-score vs. position. The tRNA (K00228.1), length 82 nt, is embedded in mRNA sequence (AF452886, 22–270 nt) at position 170–246 (represented as a black box). The Z-score for the sliding window (step size = 1) is plotted vs. position. The Z-score value is placed in the center of the window. Three different window lengths (black-60 nt; blue-82 nt; red-95 nt) are plotted. The blue plot is a scan using the exact tRNA length (82 nt) as the window size. This tRNA was detected using window lengths as short as 60 nt and as long as 95 nt.

**Figure 2:**
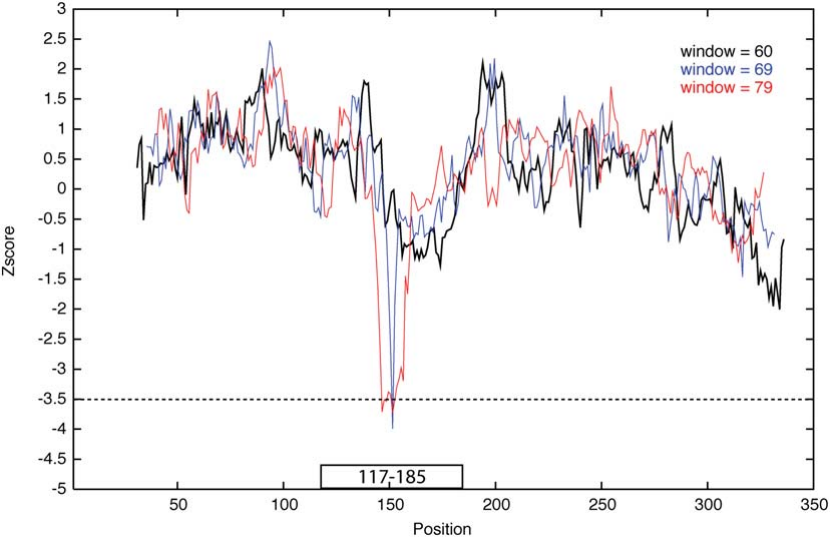
Z-score vs. position. The tRNA (AF076356.1), length 69 nt, is embedded in mRNA sequence (NM_001003966, 1–366 nt) at position 117–185 (represented as a black box). The Z-score for the sliding window (step size = 1) is plotted vs. position. The Z-score value is placed in the center of the window. Three different window lengths (black-60 nt; blue-69 nt; red-79 nt) are plotted. The blue plot is a scan using the exact tRNA length (69 nt) as the window size. This tRNA was not detected using window length of 60 nt and detected only by a single point using a window length of 79 nt.

A second experiment was performed to further explore the question of optimal values for step size and window delta. Ten tRNA from the Rfam database [50] were embedded at random locations within 300 nt background sequences (Table S5). The selected tRNA ranged in length from 68 nt to 91 nt and generated large negative Z-scores (<−4.0) when evaluated in isolation. The background sequences used were mRNA transcripts that had no significant Z-score along their length. A Z-score was calculated at each position along the total sequence (step size = 1) for each window sizes from 60 to 95 nt (window delta = 1). In most cases it was possible to detect the tRNA in the embedded sequences using a step size of 5 and a window delta of 5 (Figure 1). However, in some cases the window size and window delta needed to be smaller than this to be certain of finding the transcript (Figure 2).

Based on the above results, we chose to use a step size of 5 nt and a window delta of 5 nt for the remainder of our analysis. This provided a high probability of detecting most ncRNA while keeping computational time manageable.

### ncRNA Prediction on Chromosome VI of *S. cerevisiae* and *S. bayanus*

The ncRNA prediction method was applied to intergenic regions of *S. cerevisiae* chromosome VI using window sizes from 75 to 200 nt, a window delta size of 5 nt, and a step size of 5 nt. The UTRs of most genes in the *S. cerevisiae* genome are unknown so the term intergenic used here refers to the distance between ORFs of adjacent annotated genes. Genes classified as dubious in SGD [45] were ignored. The UTRs of the flanking genes are thus included in the intergenic region, and those containing structure [51] may be detected. The limited data available on *S. cerevisiae* 5′ and 3′ UTRs shows that most UTRs are short (3′ UTR median length 91 nt, 5′ UTR median length 68 nt) [12],[13], suggesting that most of the structural signals detected should come from independent ncRNA rather than UTRs. Only intergenic regions greater than 90 nt in length were evaluated.

Forward and reverse DNA strands were evaluated independently since the GU pairing in ncRNA confers different folding potential to the complementary strands. In an attempt to reduce the rate of false positives produced by the screen, the analysis was repeated in syntenic regions of *S. bayanus* (MCYC623) [52]. For a region to be considered syntenic, it had to have the same flanking genes with the same orientation in both *S. bayanus* and *S. cerevisiae*. A total of 66 syntenic regions satisfying these criteria were identified. The percent identity between these regions in *S. cerevisiae* and *S. bayanus* varied between 18.0% and 76.5% with an average of 57.0% (Table S6). Predicted structural elements common to both species were taken as ncRNA candidates. There were no constraints placed on the relative position of the structural predictions in syntenic regions, only that they appeared between the same two flanking genes in both species.

There were 23 intergenic regions in *S. cerevisiae* that produced Z-scores ≤−3.5 and 24 intergenic regions in *S. bayanus* that produced Z-scores ≤−3.5. Fourteen of these regions were common to both *S. cerevisiae* and *S. bayanus* and resulted in a total of 16 high priority candidates (two syntenic regions produced two separate candidates) (Table 5). In many cases, a Z-score below the cutoff criterion was generated from both the Watson and Crick strand. For this reason, experimental testing was performed on both strands independently for all candidates. An example of the Z-score values generated by evaluating the Watson strand for each position in the intergenic region between SEC4 and VTC2 for all window sizes is provided in Table S7. The position of windows producing Z-scores ≤−3.5 within selected intergenic regions are given in Figures S7, S8, S9, and S10.

**Table 5:** Candidate transcripts on chromosome VI.

Candidate	Flanking Genes	Strand	Start (5′)	End (3′)	Length	Comments
*RUF20*	SEC4-VTC2	Crick	131056	131498	442	Complete transcript (flanking genes on Watson)
*RUF21*	TUB2-RPO41 (1)	Crick	58520	57814	706	Complete transcript (flanking genes on Watson)
*RUF22*	ROG3-PES4 (1&2)	Crick	199801	∼199287	>514	3′ end uncertain (flanking genes on Watson)
*RUF23*	RPL2A-YFR032C	Watson	221702	∼221955	>253	3′ end uncertain (flanking genes on Crick)
Gene?	YFL051C-ALR2	Watson				Complex, possibly 3 transcripts
Gene?	IES1-YFL012W	Crick	∼109984	110374	>390	Cap only. No data from 5′ UTR of IES1 so observed cap could be IES1 5′ end.
UTR	TUB2-RPO41 (2)	Watson				3′ end TUB2
UTR	YFR017C-YFR018C	Crick				3′ end YFR018C
UTR	CDC4-SMC1	Watson				3′ end CDC4
UTR	ALR2-SWP82	Crick				5′ end ALR2
UTR	GYP8-STE2	Crick				5′ end GYP8
UTR	ACT1-YPT1	Crick				5′ end of long ACT1 transcripts
?	DUG1-YFR045W					no transcript ends
?	GSY1-YFR016C					no transcript ends
?	RIM15-HAC1					no transcript ends

Each candidate was mapped using RACE. This data was combined with cDNA data from Miura et al, 2006 to determine transcript ends.

### Experimental Verification

Northern blots and rapid amplification of cDNA ends (RACE) were used to test the validity of the ncRNA candidates. Since the environmental conditions required for expression of the ncRNA gene candidates were unknown, nine conditions were tested. Conditions were selected that have been shown to generate high overall transcript expression [53],[54]. These nine conditions were: heat shock (25°C to 37°C), diamide treatment, growth in minimal media, saturated growth in minimal media, anaerobic growth, sporulation, schmooing, YPGlycerol (non-fermentable carbon source), and YPD growth. RNA was isolated and northern blotting was performed (see Materials and Methods). Strand specific blotting protocol was used for the northern blot analysis to identify the transcribed strand and to help rule out DNA contamination. Northern blotting confirmed expression of transcripts between SEC4 and VTC2 (*RUF20*) on the Crick strand and between *YFL051C* and *ALR2* on the Watson strand (Figure S11). The ACT1-YPT1 transcript showed strong expression on the Crick strand under all conditions but later proved to be part of the *ACT1* 5′ UTR (data not shown).

Rapid amplification of cDNA ends (RACE) was used to measure the 5′ or 3′ end of flanking genes as well as map candidate gene ends (Table 5, Table S8, Table S9). The cDNA was generated using a poly-T primer from RNA collected from anaerobic or heat shock conditions (see Materials and Methods). The RACE analysis proved considerably more sensitive than northern blotting.

In addition to this experimental data, several publicly available data sets were evaluated for their value in substantiating these ncRNA predictions. Tiling array data [11],[12] has been used by several investigators to substantiate computational ncRNA predictions. However, we found this data quite noisy and difficult to interpret with a high degree of confidence. It also remains a point of debate whether all of the transcription measured by microarray tiling experiments represents true functional transcripts or whether some of it represents spurious transcription or experimental artifact [3], [4], [9], [55]–[58]. The sequenced cDNA library data appears to be more useful in verification of ncRNA predictions [13]. The data included information on transcript ends and as such was likely to derive from a functional transcript. A summary of all the experimental data is provided in Table 6.

**Table 6:** Summary of experimental data for the 16 ncRNA candidates evaluated.

Name	Flanking Genes	Strand	cDNA	Northern	RACE	Comments
*RUF20*	SEC4-VTC2	Crick	Yes	Yes	Yes	Strong Support
*RUF21*	TUB2-RPO41 (candidate1)	Crick	Yes	-	Yes	Strong Support
*RUF22*	ROG3-PES4 (candidate 1 & 2)	Crick	Yes	-	Yes	Strong Support (same transcript)
*RUF23*	RPL2A-YFR032C	Watson	-	-	Yes	Strong Support
Gene?	YFL051C-ALR2	Watson	-	Yes	Yes	Complex (3 transcripts?)
Gene?	IES1-YFL012W	Crick	Yes	np	Yes	Good Support
UTR	TUB2-RPO41 (candidate 2)	Watson	-	-	Yes	3′ UTR TUB2 (223 nt)
UTR	YFR017C-YFR018C	Crick	Yes	-	Yes	3′ UTR YFR018C (164 nt)
UTR	CDC4-SMC1	Watson	-	np	Yes	3′ UTR CDC4 (101 nt)
UTR	ALR2-SWP82	Crick	-	-	Yes	5′ UTR ALR2 (750 nt)
UTR	GYP8-STE2	Crick	-	-	Yes	5′ UTR GYP8 (≥249 nt)
UTR	ACT1-YPT1	Crick	Yes	Yes	-	5′ UTR ACT1 (120 nt)
?	DUG1-YFR045W	unknown	-	np	-	Insufficient support
?	RIM15-HAC1	unknown	-	-	-	Insufficient support
?	GSY1-YFR016C	unknown	-	np	-	Insufficient support

These candidate regions produced Z-scores ≤−3.5 in both *S. cerevisiae* and *S. bayanus*. RACE data was evaluated from RNA collected under two different conditions (anaerobic growth, heat shock from 25°C to 37°C). The cDNA data is taken from Miura et al, 2006. The northern contained total RNA from 9 different conditions as described in Materials and Methods. A “-” in the column indicates that no signal was detected. A “np” in the northern column identifies candidates that were not probed using northern blot.

**Figure 3:**
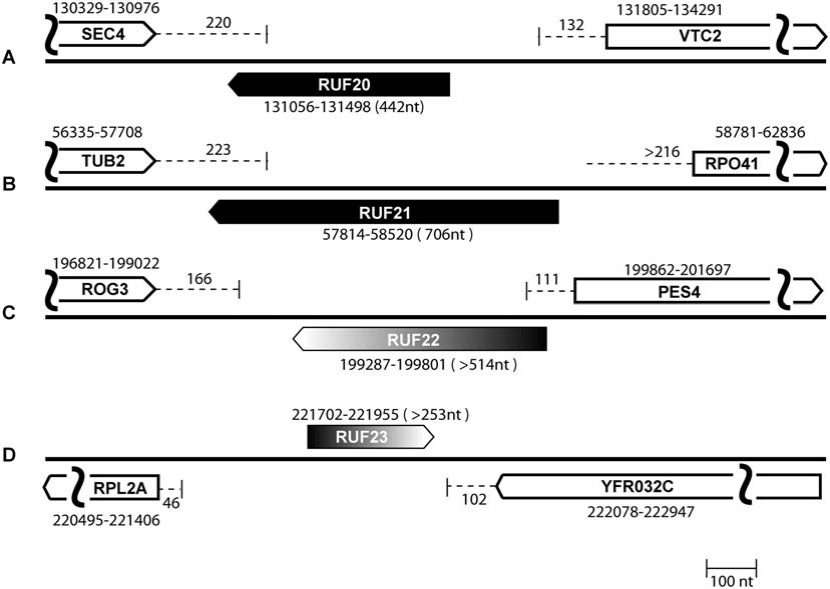
Schematic of ncRNA candidates. The genes annotated in SGD are represented as open boxes containing the name of the gene. Position numbers above the genes on chromosome VI are taken from SGD. Dotted lines extending from the boxes represent UTR regions and numbers above the lines indicate the measured length of the UTR. The curved vertical lines signify that the entire length of the flanking genes is not included in the figure. The ncRNAs for which complete RACE data are available are shown as black boxes, and the candidates for which there is incomplete RACE data are shown as gray or black-to-gray gradient boxes. (A) *RUF20* between SEC4 and VTC2 (B) *RUF21* between TUB2 and RPO41 (C) *RUF22* between ROG3 and PES4 (D) *RUF23* between RPL2A and YFR032C.

The candidates in Table 6 are listed in order of increasing experimental support. The top four ncRNA candidates have been assigned names *RUF20* (RNA of unknown function) to *RUF23* (Figure 3). The RUF name was chosen to follow the naming convention established by previous investigators [34]. These transcripts do not appear to be snoRNA or to encode an ORF (see Materials and Methods). One of the candidates, *RUF22*, overlaps with an autonomously replicating sequence, ARS607. One other ncRNA candidate, IES1-YFL012W, partially overlaps (120 bp) with the dubious ORF *YFL012W-A* which is on the opposite strand (Watson). This dubious gene also partially overlaps (120 bp) the *IES1* gene. According to SGD, this dubious ORF is unlikely to encode a protein based on available experimental and comparative sequence data [45].

It is reasonable to question whether our computational screen provided an improved ability to identify ncRNA relative to simple random experimental searches. Previous investigators have shown that randomly probing intergenic regions of the *S. cerevisiae* is unlikely to reveal ncRNA. In the work by McCutcheon & Eddy, 20 intergenic regions were chosen randomly and probed by northern blot [34]. None of these regions produced a transcript. Olivas, Muhlrand and Parker also provided evidence that probing intergenic regions is unlikely to produce a transcript even though they were conducting a directed search for ncRNA [33]. They performed two different screens in an effort to discover ncRNA. In one case, they used a computational approach to identify 10 locations in the genome that contained potential RNA polymerase III binding motifs. When they probed the 10 regions, only one was found to express a transcript. In their second screen, they identified regions within the genome with large gaps between genes. They expected these regions to contain ncRNA transcripts because the high density of genes in the *Saccharomyces* genome suggested that any large gaps were likely to be occupied by unannotated genes. Probing 59 such regions revealed 15 potential transcripts. It is clear that even probing regions expected to contain ncRNA transcripts is often unsuccessful. Our experimental screen of 16 candidates produced 4 ncRNAs with strong support, 2 potential ncRNA with weaker support, and 6 UTRs likely to contain structure (Table 6). Thus, it appears that our computational method improves ncRNA identification over simple random searches.

### SEC4-VTC2 Candidate in *S. bayanus* and *Ashbya gossypii*

**Figure 4:**
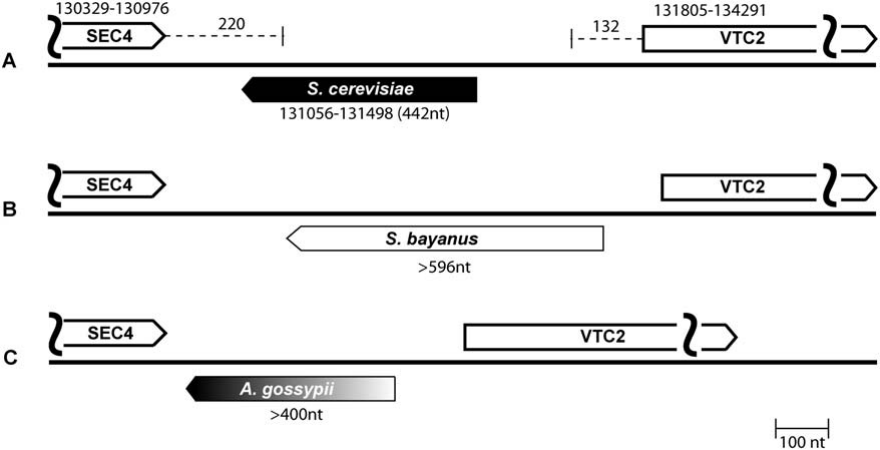
Schematic of *RUF20* in *S. cerevisiae*, *S. bayanus*, and *A. gossypii*. Open boxes represent the flanking genes, SEC4 and VTC2. The transcripts for which complete RACE data are available are shown as black boxes, and the candidates for which there is incomplete RACE data are shown as blank or black-to-gray gradient boxes. The coordinates for the bounds of the genes are noted in *S. cerevisiae*. The curved vertical lines signify that the entire length of the flanking genes is not included in the figure. (A) *RUF20* in *S. cerevisiae*. (B) *RUF20* in *S. bayanus*. (C) *RUF20* in *A. gossypii*.

To further validate the SEC4-VTC2 ncRNA candidate, RACE was performed in syntenic regions of *S. bayanus* and the more distantly related hemiascomycete species *Ashbya gossypii*. This species diverged from *S. cerevisiae* prior to the *S. cerevisiae* whole genome duplication. However, *A. gossypii* still retains many syntenic regions with *S. cerevisiae* and, in the case of the SEC4-VTC2 gene candidate, gene order and orientation are preserved. RACE products were obtained from both *S. bayanus* and *A. gossypii* (Figure 4). The fact that the transcript is preserved over such a large evolutionary distance provides strong evidence that this is a *bona fide* ncRNA gene.

## Discussion

A computational screen for structural ncRNA in *S. cerevisiae* was performed using thermodynamic stability to discriminate structural ncRNA from background sequence. The method was tested on positive and negative control sets to determine its effectiveness for identifying known ncRNA and to develop optimal search parameters. These parameters were determined to be a Z-score <−3.5, window sizes 75 nt to 200 nt, step size of 5 nt, and window delta of 5 nt. The parameters were then used to screen for novel ncRNA in the intergenic regions of *S. cerevisiae* chromosome VI. To reduce the number of false positive predictions, an independent analysis was performed on syntenic regions of *S. bayanus*. The set of predictions found in common in both species were subjected to further experimental verification. Like all computational ncRNA gene discovery approaches currently available, our method can only provide guidance on regions likely to contain structural elements. It cannot predict the exact location of the ncRNA gene or its precise ends. These must be determined experimentally.

Northern blots, rapid amplification of cDNA ends (RACE), and publicly available cDNA library data were used to test the predictions. Each of these methods was selected for specific reasons. The strength of northern blot analysis is that it does not rely on transcript amplification and hence avoids artifacts that can result from an amplification step. However, it is not as sensitive as other methods and this can be a significant limitation when testing for ncRNA that may be expressed at low levels. RACE provides greater sensitivity than northern blot analysis but may be subject to amplification artifacts. The potential for artifacts is reduced because the 5′ and 3′ ends of the transcript are captured. The presence of a cap and poly-A tail provides strong evidence that the transcript has been processed by the cellular machinery and is a legitimate functional transcript. This makes the approach superior to methods such as tiling arrays that provide information on transcription but for which it is difficult to distinguish transcriptional noise from genuine transcripts. The publicly available cDNA data used here also has the advantage of capturing the transcript 5′ and 3′ ends, providing strong evidence for a legitimate, processed transcript.

The initial computational screen presented here produced sixteen ncRNA gene candidates on chromosome VI of *S. cerevisiae*. Four candidates are well supported by experimental data and have been given the names *RUF20* to *RUF23* (Table 5). The *RUF20* candidate is also expressed in *S. bayanus* and in the more distantly related species *A. gossypii* (Figure 4). All of the transcripts were evaluated for the possibility that they might be snoRNA or encode a protein but this was shown to be unlikely (see Materials and Methods). Two additional candidates are also supported by experimental evidence but further experimental testing is needed to confirm their legitimacy. Six of the candidates were found to be part of the 5′ or 3′ untranslated regions (UTRs) of annotated protein-coding genes. These structures are interesting because they may play a functional role in the UTRs of these genes (Table 5). Additional experimental analysis will be needed to determine the function of the structures as well as the function of the four new ncRNA, *RUF20* to *RUF23*.

There are several possible explanations why experimental data could not be obtained to support three of the ncRNA predictions. These predictions may represent false positives, they may not be expressed under the conditions tested, or they may be expressed at such a low level that they could not be detected. It has been shown that transcript abundance in yeast varies over six orders of magnitude and that some important transcription factors are expressed at levels as low as one transcript per thousand cells [59]. It is also possible that these transcripts are not transcribed by RNA polymerase II, the method used in this study to generate cDNA is dependent on a poly-A tail in the RNA transcript. If the ncRNA candidates are transcribed by polymerase I or III, they would likely not be captured in the cDNA library.

It should be noted that there were three genes in the positive control set (Table 2) that did not generate a Z-score <−3.5 (*snR76*, *SER3*, *RNA170*). It is questionable whether these genes actually contain significant structural elements. One of them, *snR76*, is a C/D box snoRNA and data from other investigators [19] shows that structural features are only present in a subset of these genes. It is not surprising that this category of ncRNA was not easily detected in this screen based on structural thermodynamic stability. It is clear that some classes of ncRNA will not be identified very well in structural screens. The other two genes in the positive control set were *RNA170* (unknown function) and *SER3*. The *SER3* gene suppresses expression of its neighboring gene, *SRG1*, by blocking access to the SRG1 promoter region via its transcription. *SER3* and *RNA170* are unlikely to contain significant structural features so the fact that they did not generate Z-scores less than −3.5 tends to validate the method.

Two previous investigators have performed computational genome-wide screens for ncRNA in *S. cerevisiae*. McCutchen and Eddy, 2003 used the QRNA program to search for structural elements based on observed compensatory changes in pair-wise alignments of *S. cerevisiae* species. A fixed window size of 150 nts and a step size of 50 nt were used to perform the analysis. Two structural ncRNA candidates were found on chromosome VI. One prediction, between *RIM15* and *HAC1* (74738–74738), was near one of the candidates predicted in this study between the same genes (74926–75006). They were unable to obtain sufficient experimental support for expression of this transcript. This is consistent with our experimental results as well. The second McCutchen and Eddy prediction, between *SMC1* and *BLM10*, did not correspond to any predictions generated in this study. They obtained northern blot and RACE data to support expression of this second predicted gene.

A second screen for ncRNA was performed by Steigele et al using the RNAZ program [35]. This program searches for compensatory changes in multiple sequence alignments as well as for thermodynamic stability cues indicative of structural elements. The relative contribution of these two factors in the prediction is not specified. A fixed window size of 120 nt and step size of 40 nt was used to perform the analysis. They reported a sensitivity (true positives/total) for identifying snoRNA of 47% (pooling H/ACA box and C/D box snoRNA), sensitivity for identifying snRNA of 66%, and a sensitivity of 72% for tRNA. The screen generated a total of 18 novel intergenic structural predictions on chromosome VI. Of these, 8 were predicted to be on the Crick strand and 8 on the Watson strand. Five of these intergenic regions were shared by our predictions (YFL051C-ALR2, ACT1-YPT1, TUB2-RPO41, GYP8-STE2 and YFR017C-YFR018C). All 5 of the Steigele et al predictions were on the Watson strand in these regions. Two of the predictions overlapped with our predictions (ACT1-YPT1 and YFR017C-YFR018C).

Our experimental data suggested that the YFL051C-ALR2 region is transcriptionally complex and is likely to produce more than a single transcript. This could account for the fact that both studies predicted structural elements in this region. Our RACE analysis of the ACT1-YPT1 region showed that the predicted structural element was contained within the ACT1 UTR on the Crick strand. The Steigele et al prediction overlaps within the ACT1 UTR but is predicted to be on the opposite strand (Watson). For the TUB2-RPO41 region, we experimentally confirmed a transcript on the Crick strand encompassing our predictions. This transcript overlaps with the Steigele et al prediction but is again on the opposite strand (Watson). Our GYP8-STE2 prediction proved to be part of the GYP8 5′ UTR on the Crick strand. The Steigele et al prediction in this region was on the Watson strand and is beyond the region we measured for the GYP8 UTR (although we were unable to map the end of this 5′ UTR). In the YFR017C-YFR018C region, we obtained RACE results that mapped our prediction to the Crick strand as part of the YFR018C 3′ UTR. The Steigele et al prediction, which largely overlaps our prediction, was for a gene on the Watson strand. Hence, while our predictions and those of Steigele et al are close to one another or overlapping in five regions, in all five cases they are on opposite strands.

It is interesting that there is no overlap between the QRNA and the RNAZ predictions of chromosome VI since both programs consider compensatory changes within alignments to identify structural elements. The reason for this is unclear.

There are two primary differences between the search for ncRNA presented here and the work of previous investigators. First, this method does not require sequence alignments in the analysis. Instead, it relies entirely on thermodynamic stability in unaligned syntenic regions of related species to predict ncRNA structure. The approach is capable of finding ncRNA that have moved out of register within syntenic regions and can be applied in situations where accurate alignments may be difficult to obtain.

The second difference in this work is its examination of the impact of various window sizes and step sizes on ncRNA detection. The analysis shows that small step sizes are necessary to ensure that most ncRNA are identified. It also shows that more than one window size is needed when screening for ncRNA. Some ncRNA are detected only when using short window sizes while others are detected when using only long window sizes (Table 7). Limiting the search to a single window size, as has traditionally been done, is likely to bias the screen toward a subset of ncRNA for which that window size is optimal.

**Table 7:** Detection of each snoRNA for each window size.

	Single Window Size (step size = 5)												
sno RNA	80	90	100	110	120	130	140	150	160	170	180	190	200
snR30	X	X	X	X	X	X	X	X	X	X	X	X	X
snR32	X	X	X	X	X	X	X	X	X	X	X	X	X
snR37	X	X	X	X	X	X	X	X	X	X	X	X	X
snR44	X	X	X	X	X	X	X	X	X	X	X	X	X
snR49	X	X	X	X	X	X	X	X	X	X	X	X	X
snR161	X	X	X	X	X	X	X	X	X	X	X	X	X
snR42	X	X	X	X	X	X	X	X	X	X	X	X	X
snR83	X	X	X	X	X	X	X	X		X	X	X	X
snR84	X		X	X		X	X	X	X	X	X	X	X
snR191	X		X	X	X				X		X	X	X
snR36	X	X	X	X	X	X	X	X	X	X	X	X	
snR34	X	X	X	X	X	X	X	X	X	X		X	
snR46	X	X	X		X	X		X	X				
snR86	X	X	X	X	X	X							
snR10	X			X	X	X							
snR3	X												
snR11			X										
snR82					X	X	X	X	X				
snR81	X	X							X	X	X		X
snR80	X										X		
snR35								X					
snR8				X								X	X
snR5													
snR9													
snR31													
snR33													
snR43													
snR85													
snR189													

The table provides a list of each of the 29 H/ACA snoRNA and the window sizes at which the snoRNA generated a Z-score ≤−3.5 (indicated by ‘X’). A blank space means that the snoRNA was undetected using the window size specified at the top of the column. A step size of 5 was used. See supplementary material for data using a step size of 25 and 50 nt.

The need for multiple window sizes and step sizes in the screening algorithm increases the computational investment necessary to perform the analysis. However, with the rapid increase in computer performance and the availability of computer clusters, these computations are not unreasonable. The increased computational investment will be rewarded by increased sensitivity.

Our analysis suggests that a few carefully selected window sizes will be nearly as effective at detecting ncRNA as the entire set between 75 nt and 200 nt (total of 26 window sizes). For example, when we used the entire set of window sizes from 75 nt to 200 nt, we detected 22 of the 29 known H/ACA snoRNA within embedded sequences (Table 7). If we had used only 4 window sizes (80 nt, 120 nt, 160 nt, 200 nt), we would have succeeded in identifying 90% of these H/ACA box snoRNA (20 of the 22) while reducing computational requirements by approximately 85% (4 of 26 window sizes). If these four window sizes were used with a step size of 25 nt, 77% (17 of 22) of the H/ACA box snoRNA would be detected (Table S10). This becomes 64% (14 of 22) if the step size is increased to 50 nt (Table S11).

Tradeoffs between sensitivity and computational requirements should be evaluated when performing computational screens. We recommend using a range of four window sizes when screening for ncRNA in a genome (one short, one long, and two intermediate values appears to be optimal). Our results suggest that the values of 80, 120, 160 and 200 should provide good results. A step size between 5 and 10 should also provide a good screen. These parameters should provide good ncRNA detection while keeping computational time manageable. The development of an efficient computational algorithm implementing the methodology presented here would also significantly reduce computational run time.

This screen used a simple cutoff Z-score value (≤−3.5) to discriminate ncRNA. The sensitivity of the screen could probably be improved if a more sophisticated cutoff criteria were developed in which the Z-score cutoff was a function of window size. The number of aberrant negative Z-scores dropped as a function of window length in the negative control sets demonstrating that the likelihood of producing large negative Z-score drops with increasing window length. Developing a Z-score cut-off value as a function of window length would probably improve the sensitivity of the screen at longer window sizes.

This work demonstrates that structural thermodynamic stability is an effective tool for predicting ncRNA genes. As examples of ncRNA are accumulated through computational screens such as this, it may become possible to determine ncRNA key features and gain insight into their biological function. Computational methods can complement experimental approaches in the effort to gain a deeper understanding of these genes.

## Materials and Methods

### Strains

S288C was used for all growth conditions except for sporulation (SK1) and pheromone treatment (BY4741).

### Heat Shock from 25°C to 37°C

Cells grown continuously at 25°C were collected by centrifugation, resuspended in an equal volume of 37°C medium, and returned to 37°C for an additional 20 minutes. The RNA was then isolated as described below. RNA was collected after twenty minutes as it has been shown to be the point of maximum RNA expression [53].

### Schmooing

Pheromone treatment stimulates yeast cells to increase the expression of mating genes, arrest cell division in the G1 phase, and form polarizing mating projections directed toward the pheromone source [60]. Overnight yeast cultures grown in YPD at 30°C were treated with 50 nM α-factor (GenScript Corporation). Cells were examined under a microscope to ensure schmooing was induced. Total RNA was extracted 75 minutes after pheromone treatment.

### Diamide Treatment

A strong cellular response to diamide treatment has been shown previously [53]. It resembles a composite response to heat shock, H_2_O_2_ treatment and menadione treatment. It induces cellular redox genes and genes associated with defense against reactive oxygen species. Diamide (Research Organics) was added to cell cultures grown in YPD at 30°C in late log phase to a final concentration of 1.5 mM. Cells were returned to 30°C for growth for 30 minutes. RNA was then isolated as described above.

### Synchronized Sporulation

This growth condition induces expression of genes involved in meiosis and spore morphogenesis. SK1 yeast cells were sporulated in a synchronous meiosis as described previously [61]. Briefly, yeast cultures were pre-grown in YPD to saturation at 30°C, diluted 200-fold into 100 ml of YPA (1% yeast extract, 2% Bacto-peptone, 2% acetate), and grown to early stationary phase (about 5×10^7^ cells/ml). Cells were then washed with water and resuspended into 100 ml of SPM (sporulation media consisting of 0.3% potassium acetate and 0.02% raffinose). Sporulation was carried out at 30°C under conditions that allowed good aeration. Expression data suggested that metabolic, early, middle and late genes were active 11 hours after transfer to sporulation media so total RNA was collected at this time point [54]. Cells were inspected under a microscope to ensure that sporulation of at least some of the cells had taken place. RNA was then isolated as described below.

### Anaerobic Growth

S288C yeast cells were grown for approximately 55 hours in 100 ml of minimal media (YNB) in an anaerobic chamber using an anaerobic gas generating system (Mitsubishi Gas Chemical Company, Inc.). Four minimal media plates were also streaked with S288C and grown anaerobically for the same time period. The anaerobic chamber was then opened and the cells growing on the plates were added to the cells in the liquid growth by washing. Total RNA from all of the cells was isolated immediately as described below.

### Saturated, Rich Media Growth, and YPG

Saturated growth has been shown to activate gene expression, presumably allowing the cells to adapt to nutrient depleted conditions [53]. S288C cultures were grown to saturation (OD 3) in minimal media (YNB). They were also grown to logarithmic phase in rich media (YPD) and on a nonfermentable carbon source, YPGlycerol. All three cultures were grown at 30°C and aerated by shaking at 250–300 rpm.

### RNA Isolation

A phenol-chloroform extraction protocol was used as described previously [62] to extract total RNA from *S. cerevisiae*, *S. bayanus* and *A. gossypii*. All glassware used in the procedure was baked for 4 hours to destroy RNase activity. Reusable plasticware and laboratory bench surfaces were treated with RNAzap (Biohit, Inc.). RNAse-free water was prepared by treating with Diethyl pyrocarbonate for one hour and then autoclaving. Cells were harvested from 50 ml cultures at an OD_600_ of 1–3 (1 OD = 3×10^7^ cells/ml) unless otherwise specified. The cells were collected via centrifugation (except *A. gossypii* cells which were collected using a vacuum filter). The cell wall was disrupted by vortexing at high speed with acid-washed glass beads in a solution containing guanidine thiocyanate. Total RNA was purified using multiple washes with a combination of hot phenol and chloroform.

The total RNA was treated with TURBO DNase (Ambion) and incubated at 37°C for 30 minutes prior to using for RACE or northern applications. The DNase activity was destroyed by heating to 70°C for 5 minutes per the standard protocol. RNA quality was assessed by measuring absorbance at a wavelength of 260 nm on a NanoDrop (ND-1000) spectrometer.

### Northern

A 6%, 7 M urea, 1× TBE denaturing polyacrylamide gel was used to make a northern blot with total RNA as described previously [63]. Total RNA was treated with TURBO DNase (Ambion) and incubated at 37°C for 30 minutes prior to gel loading to ensure that no DNA was present. It was loaded onto the gel and run at 150 V for 3 hours. The total RNA was transferred from the gel to a nylon membrane using the OWL Scientific Panther Semi-Dry Electroblotter (Model # HEP-1) with a current of 200 milliamperes for a period of 1 hr. The RNA was fixed to the blot with UV crosslinking. Radioactive strand-specific probes were produced using the Strip-EZ system with α-P^32^ dATP (Ambion). Each probe was used to on a separate northern blot. This provided a check that the observed signal derived from only a single strand and was not the result of DNA contamination (which would produce signal from both strands). The probes were detected by exposing the blot to BioMax XAR film (Kodak) at −80°C 24–48 hours.

### Rapid Amplification of cDNA Ends (RACE)

The SMART RACE cDNA Amplification Kit (Clontech) was used to map transcript ends. Total RNA was isolated from S288C under two different growth conditions: anaerobic growth and heat shock from 25°C to 37°C. It was treated with TURBO DNase (Ambion) prior to making the cDNA.

To obtain RACE products for the ncRNA candidates, a RACE reaction and nested reaction were performed for both the Watson and Crick strand since it was uncertain which strand the transcript might be generated from. The temperature profiles developed to optimize the reaction are given in Appendix A. A hot start approach was used to minimize extraneous amplification by allowing the reaction tubes to reach a temperature of 94°C for 1 minute before adding the Ex Taq (Takara) polymerase. The RACE products were electrophoresed on a 1% agarose gel and the resulting bands were cut out of the gel. They were purified using one of two methods. The first was to use the QIAquick Gel Extraction Kit (QIAGEN), according to the standard protocol. Alternatively, the gel slices were frozen at −20°C and then spun on a tabletop centrifuge at 1400 rpm for 20 minutes. The sample was then drawn from the top of the resulting liquid. This proved a quick and reliable method for obtaining purified product. The purified RACE products were sequenced using standard BigDye chemistry, version 1.3, according to standard protocols (Applied Biosystems).

RACE primers were designed according to guidelines provided in the SMART RACE kit. They were 20–28 nt in length, had a GC content between 50–70%, a melting temperature ≥72°C, and had no more than 2 C's or G's in the last 5 nucleotides of the oligonucleotide. Each primer was confirmed to be unique in the genome using the “fuzznuc” program that is part of the EMBOSS utilities [64].

### Calculating Z-score

The Z-score compares the minimum folding energy (MFE) of a sequence, **x**, to the distribution of MFE generated by permuted versions of **x** having the same di-nucleotide composition. The di-nucleotide composition must be preserved because of the importance of stacked base-pairs in the MFE calculation [65]. The MFE of each sequence, **x**, was calculated using the RNAfold program [44]. Each sequence was then shuffled 500 times using the shuffle program provided in Sean Eddy's squid utilities [47] and a mean and standard deviation were calculated for the resulting distribution. The Z-score was then calculated using the equation
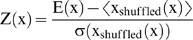
where <·> and σ (·) denote the mean and the standard deviation of the MFEs of the sequences in x_shuffled_(**x**). Hence, the Z-score represents the number of standard deviations that the sequence **x** deviates from the mean MFE of the shuffled sequences.

### Genome Sequences

The genome sequence data used for ncRNA prediction and subsequent evaluation of open reading frame coding potential is listed in Table 8.

**Table 8:** Genomic sequences used for ncRNA prediction.

Genome	Strain	Date Downloaded	Reference
*Saccharomyces cerevisiae*	S288C	11/04/06	[45]
*Saccharomyces paradoxous*	NRRL Y-17217	11/04/06	[66]
*Saccharomyces mikatae*	IFO1815	10/12/07	[66]
*Saccharomyces kudriavzevii*	IFO1802	11/04/06	[52]
*Saccharomyces bayanus*	MCYC623	11/04/06	[66]
*Candida glabrata*	CBS138	10/12/07	[69]
*Ashbya gossypii*	ATCC 10895	6/11/07	[70]

### ORF Evaluation of ncRNA Candidates

It was important to investigate the possibility that the ncRNA candidates might be protein-coding genes. Comparative genomics was used to investigate this possibility for the four ncRNA gene candidates *RUF20*, *RUF21*, *RUF22* and *RUF23* (Table 5). This approach has been applied by other investigators with a high degree of success [66].

There are no conserved ORFs within the three candidates *RUF21*, *RUF22* and *RUF23* among the closely related species *S. cerevisiae*, *S. paradoxus*, and *S. bayanus* (*sensu stricto*). These transcripts are thus unlikely to be protein-coding genes.

The *RUF20* candidate contains one ORF consisting of 8 amino acids conserved among *S. kudriavzevii*, *S. bayanus*, *S. paradoxus*, and *S. mikatae* (*sensu stricto*). However, the pattern of substitution among these species is not consistent with synonymous amino acid substitutions as would be expected for a protein-coding gene (two mutations are in the 1^st^ codon position, one mutation is in the 3^rd^ position). The ORF is not conserved in *Candida glabrata* or *A. gossypii*. This is significant because our RACE data confirmed expression of the transcript in *A. gossypii*. In addition, the 8 amino acid ORF does not contain any splice signals suggesting that it is spliced to another exon. While a number of short ORFs have been identified in yeast [67], there are none known to be as short as 8 amino acids. Taken together, this data strongly suggests that the short *RUF20* ORF conserved among the *sensu stricto* does not encode a protein.

### SnoRNA Evaluation of ncRNA Candidates

The SnoScan [19] and SnoGPS [20] programs were used to test if the ncRNA candidates were likely to be snoRNA. The SnoScan program searches for features characteristic of C/D box snoRNA. None of the *RUF20* to *RUF23* candidate genes have features characteristic of C/D box snoRNA according to the program. The SnoGPS program searches for features characteristic of H/ACA box snoRNA. According to the program, *RUF23* is unlikely to be a H/ACA box snoRNA. The program found some features of H/ACA box snoRNA evident in the *RUF20*, *RUF21* and *RUF22* candidates, although their overall bit score was low (28.4, 29.3, and 29.9 respectively). A bit score value of 36 is recommended as the cutoff value when searching for new H/ACA snoRNA. To further evaluate the possibility that *RUF20*, *RUF21* and *RUF22* might to be H/ACA snoRNA, sequence from two closely related species was used. The homologous gene sequences from *S. paradoxus* and *S. bayanus* were evaluated using the snoGPS program. The *RUF20* candidates in these species were found to be unlikely to be a H/ACA snoRNA by the program. The *RUF21* and *RUF22* genes did generate possible H/ACA snoRNA candidates in the related species but there was no common rRNA target identified among the homologous sequences. Hence, the candidates appear to be unlikely H/ACA snoRNA genes.

## Supporting Information

Figure S1 Regions producing Z-scores ≤−3.5 for Random9 sequence of the negative control set. Positions along the 300 bp sequence are shown on the scale at the top of the figure. All windows producing a Z-score ≤−3.5 are plotted below the scale as a rectangle. The Z-score value for each window is shown above the rectangle along with the position of the window in the sequence (in parenthesis). The total length of the window is shown in brackets. Overlapping windows are combined to determine the total length of sequence producing the false positive indications (in this case, positions 61–285, for a total length of 225 bp). The figure was drawn using resources in the BioPerl toolkit [68]. See Table S12 for all the Random sequences used in this study.
(0.85 MB TIF)

Figure S2 Regions producing Z-scores ≤−3.5 for Random13 sequence of the negative control set. Positions along the 300 bp sequence are shown on the scale at the top of the figure. All windows producing a Z-score ≤−3.5 are plotted below the scale as a rectangle. The Z-score value for each window is shown above the rectangle along with the position of the window in the sequence (in parenthesis). The total length of the window is shown in brackets. The figure was drawn using resources in the BioPerl toolkit [68]. See Table S12 for all the Random sequences used in this study.
(0.05 MB TIF)

Figure S3 Regions producing Z-scores ≤−3.5 for the shuffled LSR1 sequence of the negative control set. Positions along the 1175 bp sequence are shown on the scale at the top of the figure. All windows producing a Z-score ≤−3.5 are plotted below the scale as a rectangle. The Z-score value for each window is shown above the rectangle along with the position of the window in the sequence (in parenthesis). The total length of the window is shown in brackets. Overlapping windows are combined to determine the total length of sequence producing the false positive indications. The figure was drawn using resources in the BioPerl toolkit [68]. See Table S12 for all the shuffled sequences used in this study.
(0.09 MB TIF)

Figure S4 Regions producing Z-scores ≤−3.5 for the shuffled RUF5-1 sequence of the negative control set. Positions along the 710 bp sequence are shown on the scale at the top of the figure. All windows producing a Z-score ≤−3.5 are plotted below the scale as a rectangle. The Z-score value for each window is shown above the rectangle along with the position of the window in the sequence (in parenthesis). The total length of the window is shown in brackets. The figure was drawn using resources in the BioPerl toolkit [68]. See Table S12 for all the shuffled sequences used in this study.
(0.05 MB TIF)

Figure S5 Regions producing Z-scores ≤−3.5 for the LSR1 sequence of the positive control set. Positions along the 1175 bp sequence are shown on the scale at the top of the figure. All windows producing a Z-score ≤−3.5 are plotted below the scale as a rectangle. The Z-score value for each window is shown above the rectangle along with the position of the window in the sequence (in parenthesis). The total length of the window is shown in brackets. Overlapping windows are combined to determine the total length of sequence producing the true positive indication. The figure was drawn using resources in the BioPerl toolkit [68].
(1.39 MB TIF)

Figure S6 Regions producing Z-scores ≤−3.5 for the RUF5-1 sequence of the positive control set. Positions along the 710 bp sequence are shown on the scale at the top of the figure. All windows producing a Z-score ≤−3.5 are plotted below the scale as a rectangle. The Z-score value for each window is shown above the rectangle along with the position of the window in the sequence (in parenthesis). The total length of the window is shown in brackets. Overlapping windows are combined to determine the total length of sequence producing the true positive indication. The figure was drawn using resources in the BioPerl toolkit [68].
(0.09 MB TIF)

Figure S7 Regions producing Z-scores ≤−3.5 for intergenic sequence between SEC4 (YFL005W) and VTC1 (YFL004W). Positions along the 828 bp sequence are shown on the scale at the top of the figure. All windows producing a Z-score ≤−3.5 are plotted below the scale as a rectangle. The Z-score value for each window is shown above the rectangle along with the position of the window in the sequence (in parenthesis). The total length of the window is shown in brackets. Overlapping windows were combined to obtain a candidate region for experimental testing. The figure was drawn using resources in the BioPerl toolkit [68].
(0.34 MB TIF)

Figure S8 Regions producing Z-scores ≤−3.5 for intergenic sequence between TUB2 (YFL037W) and RPO41 (YFL036W). Positions along the 1072 bp sequence are shown on the scale at the top of the figure. All windows producing a Z-score ≤−3.5 are plotted below the scale as a rectangle. The Z-score value for each window is shown above the rectangle along with the position of the window in the sequence (in parenthesis). The total length of the window is shown in brackets. Overlapping windows were combined to obtain a candidate region for experimental testing. The figure was drawn using resources in the BioPerl toolkit [68].
(3.05 MB TIF)

Figure S9 Regions producing Z-scores ≤−3.5 for intergenic sequence between ROG3 (YFR022W) and PES4 (YFR023W). Positions along the 839 bp sequence are shown on the scale at the top of the figure. All windows producing a Z-score ≤−3.5 are plotted below the scale as a rectangle. The Z-score value for each window is shown above the rectangle along with the position of the window in the sequence (in parenthesis). The total length of the window is shown in brackets. Overlapping windows were combined to obtain a candidate region for experimental testing. This intergenic sequence produced two separate candidate regions. The figure was drawn using resources in the BioPerl toolkit [68].
(0.21 MB TIF)

Figure S10 Regions producing Z-scores ≤−3.5 for intergenic sequence between RPL2A (YFR031C-A) and YFR032C. Positions along the 671 bp sequence are shown on the scale at the top of the figure. All windows producing a Z-score ≤−3.5 are plotted below the scale as a rectangle. The Z-score value for each window is shown above the rectangle along with the position of the window in the sequence (in parenthesis). The total length of the window is shown in brackets. Overlapping windows were combined to obtain a candidate region for experimental testing. The figure was drawn using resources in the BioPerl toolkit [68].
(0.07 MB TIF)

Figure S11 Northern blot analysis. Nine different environmental conditions were tested as labeled across the top of each blot. (A) Watson, SEC4-VTC2. (B) Crick, SEC4-VTC2. Expression was observed under all conditions except schmooing, with the strongest expression under anaerobic conditions. (C) Watson, YFL051C-ALR2. Expression was observed under all conditions except schmooing and sporulation. The strongest expression was observed in YPG and YPD. (D) Crick, YFL051C-ALR2.
(5.98 MB TIF)

Table S1 Negative control sequences for six intergenic regions. The table gives the genes flanking the selected intergenic region as well as the measured transcription start site for the genes (when this data is available). The number of times each start site was measured is given in parentheses if more than one measurement was obtained. Transcription start site data is from [14].
(1.26 MB DOC)

Table S2 Z-scores for sequences in negative control set producing Z-scores ≤−3.5.
(0.05 MB DOC)

Table S3 Z-scores for sequences in positive control set producing Z-scores ≤−3.5.
(0.15 MB DOC)

Table S4 GC content of regions in the negative and positive control sets producing Z-scores ≤−3.5.
(0.13 MB DOC)

Table S5 ncRNA embedded in longer sequence. The GenBank accession numbers and descriptions for sequences used for the embedded ncRNA analysis. The column labeled Z-score provides the Z-score that is computed when the exact tRNA length is used.
(0.07 MB DOC)

Table S6 Percent nucleotide identity in syntenic regions of S. cerevisiae and S. bayanus. The “needle” program contained in the EMBOSS package was used to align intergenic regions and compute the percent identity [71]. A gap open penalty of 10.0 and a gap extend penalty of 0.5 was used to perform the alignment. It is important to note that many of the syntenic regions between the two species differ in length.
(0.16 MB DOC)

Table S7 All Z-score values for Watson strand of intergenic region between SEC4 and VTC2. The table provides the Z-score calculated for each position of the intergenic region for each window size (75 nt to 200 nt). The first column provides the sequence name. This region lies between SEC4 (YFL005W) and VTC2 (YFL004W) and is 828 bp long. The sequence name ends with the boundary values for the window being evaluated. The second column (Pos) specifies the beginning position of the window. The 3rd column (Length) gives the length of the window. The 4th column (MFE) gives the minimum folding energy of the native sequence. The 5th column (#Shuffles) gives the number of shuffled sequence used to generate a mean and standard deviation. The 6th column (Mean) gives the mean of the distribution of minimum folding energies for the shuffled sequences. The 7th column (Std. dev) gives the standard deviation for the distribution of minimum folding energies of the shuffled sequences. The 8th column (Z-score) gives the Z-score for the window being evaluated.
(0.67 MB DOC)

Table S8 The 5′ UTRs mapped by RACE. A “W” means the gene is on the Watson strand and a “C” means the gene is on the Crick strand. The cap for GYP8 was not obtained so the UTR is shown as greater than 249 nt long.
(0.10 MB DOC)

Table S9 3′ UTRs mapped by RACE. A “W” means the gene is on the Watson strand and a “C” means the gene is on the Crick strand.
(0.04 MB DOC)

Table S10 Detection of each snoRNA for each window size (step size = 25). The table provides a list of each H/ACA snoRNA and the window sizes at which the snoRNA was detected (X in box). A blank box means that the snoRNA was undetected using the window size specified at the top of the column. A step size of 25 was used for all cases.
(0.13 MB DOC)

Table S11 Detection of each snoRNA for each window size (step size = 50). The table provides a list of each H/ACA snoRNA and the window sizes at which the snoRNA was detected (X in box). A blank box means that the snoRNA was undetected using the window size specified at the top of the column. A step size of 50 was used for all cases.
(0.13 MB DOC)

Table S12 All Random and shuffled sequences used in this study. All sequences provided in fasta format.
(0.11 MB DOC)
