# Supplementary figures and images for: Non-Coding RNA Prediction and Verification in Saccharomyces cerevisiae
Source: PLoS Genet. 2009 Jan 2;5(1):e1000321. doi: 10.1371/journal.pgen.1000321 (PMC2603021; doi:10.1371/journal.pgen.1000321)

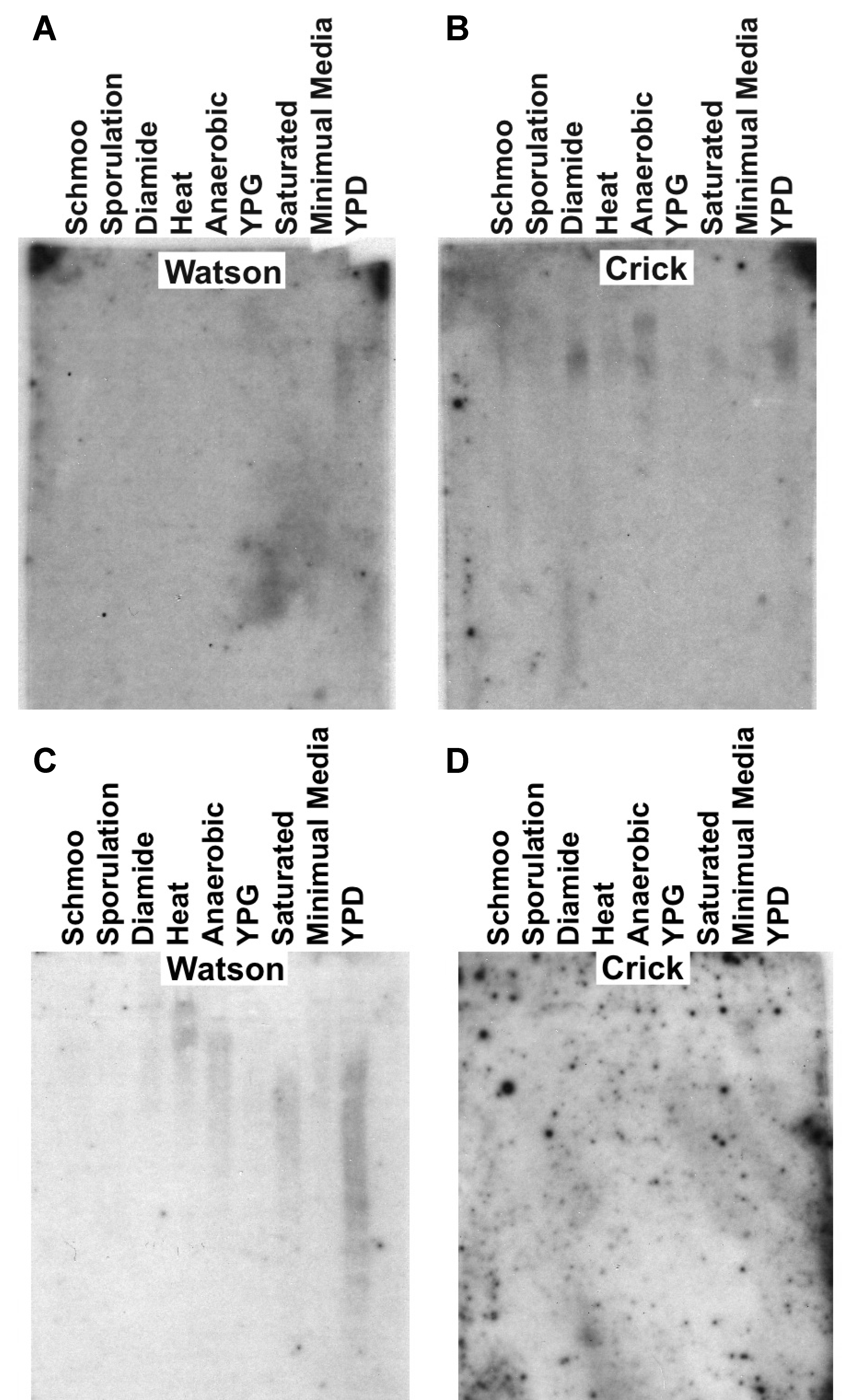

Supplement: Figure S11 — Northern blot analysis. Nine different environmental conditions were tested as labeled across the top of each blot. (A) Watson, SEC4-VTC2. (B) Crick, SEC4-VTC2. Expression was observed under all conditions except schmooing, with the strongest expression under anaerobic conditions. (C) Watson, YFL051C-ALR2. Expression was observed under all conditions except schmooing and sporulation. The strongest expression was observed in YPG and YPD. (D) Crick, YFL051C-ALR2. (5.98 MB TIF) [file pgen.1000321.s011.tif]
