# Supplementary material for: Non-Coding RNA Prediction and Verification in Saccharomyces cerevisiae
Source: PLoS Genet. 2009 Jan 2;5(1):e1000321. doi: 10.1371/journal.pgen.1000321 (PMC2603021; doi:10.1371/journal.pgen.1000321)
Supplement: Table S1 — Negative control sequences for six intergenic regions. The table gives the genes flanking the selected intergenic region as well as the measured transcription start site for the genes (when this data is available). The number of times each start site was measured is given in parentheses if more than one measurement was obtained. Transcription start site data is from [14]. (1.26 MB DOC) [file pgen.1000321.s012.doc]

Table S1. Negative control sequences for six intergenic regions. The table gives the genes flanking the selected intergenic region as well as the measured transcription start site for the genes (when this data is available). The number of times each start site was measured is given in parentheses if more than one measurement was obtained. Transcription start site data is from [14].

| **Gene 1** | **Comment** | **Gene 2** | **Comment** |
| --- | --- | --- | --- |
| RPL16B | -31, -15, -11, -8 | FKH2 | Test window is 400nt from FKH2 5' ORF |
| RPS14A | -30,-21(4), -20 | snr189 | Test window is 90nt from snr189 3' ORF |
| SSA1 | Test window is 443nt from SSA1 5' ORF | EFB1 | -33, -21(9), -11, -6, -3 |
| SPC97 | Test window is 221nt from SPC97 3' ORF | ENO2 | -38(3), -30(6) |
| YPR098C | Test window includes 37nt of YPR098C 5' ORF | MRPL51 | -20(6), -10 |
| PTP1 | Test window is 58nt from PTP1 5' ORF | SSB1 | -30(3) |
