# Supplementary material for: Non-Coding RNA Prediction and Verification in Saccharomyces cerevisiae
Source: PLoS Genet. 2009 Jan 2;5(1):e1000321. doi: 10.1371/journal.pgen.1000321 (PMC2603021; doi:10.1371/journal.pgen.1000321)
Supplement: Table S2 — Z-scores for sequences in negative control set producing Z-scores ≤−3.5. (0.05 MB DOC) [file pgen.1000321.s013.doc]

Table S2. Z-scores for sequences in negative control set producing Z-scores ≤ -3.5.

Randomly Generated sequences

Gene Start End Length Z-score

# Random13 (300 bp)

21 95 75 -3.502

# Random9 (300 bp)

61 245 185 -3.533

61 255 195 -3.576

66 245 180 -4.269

66 255 190 -4.132

66 260 195 -3.660

71 245 175 -4.032

71 250 180 -3.814

71 255 185 -4.039

71 260 190 -4.424

71 265 195 -3.693

71 270 200 -3.883

76 245 170 -4.146

76 250 175 -3.699

76 255 180 -3.709

76 260 185 -4.110

76 265 190 -3.694

76 270 195 -4.282

81 260 180 -4.239

81 265 185 -3.933

81 270 190 -3.670

106 245 140 -3.525

106 260 155 -3.571

111 270 160 -3.554

116 260 145 -4.338

116 265 150 -3.584

116 280 165 -3.735

116 285 170 -3.537

121 245 125 -3.791

121 255 135 -3.511

121 260 140 -4.182

121 265 145 -3.880

126 245 120 -3.703

126 255 130 -3.510

126 260 135 -4.124

126 265 140 -3.882

126 270 145 -3.624

131 245 115 -4.302

131 250 120 -3.766

131 255 125 -3.992

131 260 130 -3.671

136 245 110 -4.012

136 255 120 -4.522

136 260 125 -3.513

171 260 90 -3.800

176 255 80 -3.521

181 255 75 -4.115

Intergenic Regions

Gene Start End Length Z-score

# PTP1-SSB1 (300 bps)

1 155 155 -3.890

1 160 160 -4.239

1 165 165 -4.749

1 170 170 -4.238

1 175 175 -3.871

1 185 185 -3.624

6 155 150 -3.753

6 160 155 -4.214

6 165 160 -4.563

6 170 165 -4.152

6 175 170 -3.740

6 180 175 -3.757

6 190 185 -3.564

11 165 155 -3.898

11 170 160 -3.822

16 165 150 -3.608

71 170 100 -3.598

71 165 95 -3.843

76 155 80 -4.024

81 155 75 -3.877

Shuffled Positive Controls

>Gene Start End Length Z-score

# LSR1scramble (1175 bp)

491 570 80 -3.611

491 565 75 -3.523

496 570 75 -3.580

# NME1scramble (339 bp)

1 190 190 -3.804

6 190 185 -3.571

6 170 165 -3.645

6 165 160 -3.750

11 180 170 -3.597

11 170 160 -3.635

16 200 185 -3.669

26 190 165 -3.812

31 195 165 -3.704

31 190 160 -4.482

31 185 155 -3.564

31 175 145 -3.598

31 165 135 -4.599

31 160 130 -3.987

31 180 150 -3.919

36 200 165 -3.517

36 195 160 -3.674

36 190 155 -4.056

36 180 145 -4.242

36 175 140 -3.910

36 170 135 -3.609

36 165 130 -3.974

36 160 125 -4.042

36 185 150 -3.776

41 195 155 -3.872

41 185 145 -3.756

41 180 140 -3.953

41 175 135 -3.886

41 190 150 -4.081

46 205 160 -3.932

46 200 155 -3.942

46 190 145 -4.598

46 185 140 -3.975

46 180 135 -4.095

46 175 130 -4.379

46 170 125 -3.669

46 195 150 -4.293

51 205 155 -3.714

51 195 145 -4.546

51 190 140 -5.224

51 185 135 -4.311

51 180 130 -4.197

51 175 125 -4.484

51 170 120 -3.981

51 200 150 -4.287

56 235 180 -3.527

56 215 160 -3.598

56 200 145 -4.184

56 195 140 -4.726

56 190 135 -5.588

56 185 130 -4.935

56 180 125 -4.661

56 175 120 -4.530

56 170 115 -3.656

56 205 150 -4.496

61 240 180 -3.754

61 235 175 -3.694

61 230 170 -3.759

61 215 155 -3.561

61 205 145 -4.330

61 200 140 -4.774

61 195 135 -5.123

61 190 130 -5.403

61 185 125 -5.359

61 180 120 -4.810

61 175 115 -5.272

61 170 110 -4.385

61 165 105 -3.763

61 210 150 -3.608

66 205 140 -3.705

66 200 135 -4.494

66 195 130 -4.370

66 190 125 -5.164

66 185 120 -5.339

66 180 115 -5.329

66 175 110 -4.972

66 170 105 -4.190

71 210 140 -3.815

71 205 135 -3.630

71 200 130 -4.432

71 195 125 -4.188

71 190 120 -4.857

71 185 115 -5.323

71 180 110 -5.702

71 175 105 -5.300

71 170 100 -4.796

71 165 95 -3.765

71 160 90 -3.761

76 235 160 -3.598

76 210 135 -3.850

76 205 130 -3.865

76 200 125 -4.173

76 195 120 -4.439

76 190 115 -5.313

76 185 110 -5.326

76 180 105 -5.559

76 175 100 -5.514

76 170 95 -4.814

76 165 90 -3.825

76 160 85 -3.757

81 210 130 -3.543

81 205 125 -3.526

81 200 120 -4.012

81 195 115 -4.682

81 190 110 -4.833

81 185 105 -4.645

81 180 100 -5.844

81 175 95 -5.457

81 170 90 -5.772

81 165 85 -4.857

81 160 80 -4.364

101 180 80 -3.513

106 185 80 -3.512

106 180 75 -3.604

# RUF5-1scramble (710 bp)

271 355 85 -3.563

# SNR19scramble (568 bp)

411 485 75 -3.682

# TLC1scramble (1301 bp)

366 565 200 -3.515

371 570 200 -3.733

376 575 200 -3.601

376 570 195 -4.128

376 565 190 -3.991

376 560 185 -3.582

381 580 200 -3.802

381 575 195 -3.755

381 570 190 -4.319

381 565 185 -4.402

381 560 180 -3.963

386 580 195 -3.834

386 575 190 -3.742

386 570 185 -4.420

386 565 180 -4.916

386 560 175 -4.603

391 570 180 -3.885

391 565 175 -4.060

391 560 170 -4.490

426 540 115 -3.856
