# Supplementary material for: Non-Coding RNA Prediction and Verification in Saccharomyces cerevisiae
Source: PLoS Genet. 2009 Jan 2;5(1):e1000321. doi: 10.1371/journal.pgen.1000321 (PMC2603021; doi:10.1371/journal.pgen.1000321)
Supplement: Table S3 — Z-scores for sequences in positive control set producing Z-scores ≤−3.5. (0.15 MB DOC) [file pgen.1000321.s014.doc]

Table S3. Z-scores for sequences in positive control set producing Z-scores ≤ -3.5.

Gene Start End Length Z-score

# LSR1 (1175 bp)

496 695 200 -3.573

501 695 195 -3.875

501 700 200 -4.007

506 695 190 -3.794

506 700 195 -4.045

506 705 200 -4.237

511 695 185 -4.048

511 700 190 -4.822

511 705 195 -4.462

511 710 200 -5.349

516 695 180 -4.376

516 700 185 -4.519

516 705 190 -4.670

516 710 195 -4.990

516 715 200 -4.856

521 695 175 -4.552

521 700 180 -4.786

521 705 185 -5.302

521 710 190 -5.207

521 715 195 -5.170

521 720 200 -6.600

526 685 160 -3.590

526 690 165 -3.729

526 700 175 -5.100

526 705 180 -5.051

526 710 185 -5.615

526 715 190 -5.771

526 720 195 -5.676

526 725 200 -4.918

531 690 160 -3.923

531 695 165 -5.476

531 705 175 -5.952

531 710 180 -6.122

531 715 185 -5.471

531 720 190 -4.672

531 725 195 -4.192

531 730 200 -5.001

536 690 155 -4.329

536 695 160 -5.622

536 700 165 -6.445

536 710 175 -4.974

536 715 180 -4.430

536 720 185 -3.834

536 730 195 -4.241

536 735 200 -4.295

541 690 150 -4.414

541 695 155 -5.406

541 700 160 -5.842

541 705 165 -5.405

541 715 175 -3.941

546 685 140 -3.512

546 690 145 -4.968

546 695 150 -6.783

546 700 155 -5.487

546 705 160 -5.343

546 710 165 -4.322

546 720 175 -3.609

546 735 190 -3.847

551 685 135 -4.540

551 690 140 -5.643

551 695 145 -5.390

551 700 150 -4.456

551 705 155 -4.450

551 710 160 -4.081

551 715 165 -4.238

556 680 125 -4.113

556 685 130 -5.208

556 690 135 -4.644

556 695 140 -4.645

556 705 150 -3.844

561 675 115 -3.602

561 680 120 -4.016

561 685 125 -3.765

566 670 105 -3.767

566 675 110 -3.993

566 680 115 -3.556

566 685 120 -3.510

# NME1 (339 bp)

21 220 200 -3.720

41 225 185 -3.713

66 260 195 -4.234

66 265 200 -3.640

71 260 190 -3.596

76 255 180 -3.566

76 260 185 -3.677

76 275 200 -3.536

81 260 180 -3.511

81 275 195 -3.560

86 260 175 -3.513

86 270 185 -3.614

86 275 190 -3.623

91 255 165 -3.714

91 260 170 -3.883

91 265 175 -4.042

91 270 180 -3.946

91 275 185 -4.335

91 280 190 -3.749

91 285 195 -3.581

91 290 200 -4.021

96 225 130 -3.641

96 235 140 -3.592

96 250 155 -3.521

96 255 160 -4.596

96 260 165 -4.592

96 265 170 -4.734

96 270 175 -5.032

96 275 180 -4.963

96 280 185 -4.895

96 285 190 -4.531

96 290 195 -5.305

96 295 200 -4.599

101 220 120 -3.754

101 225 125 -3.910

101 230 130 -3.868

101 235 135 -3.555

101 250 150 -3.940

101 255 155 -4.826

101 260 160 -4.493

101 265 165 -4.831

101 270 170 -4.666

101 275 175 -4.764

101 280 180 -4.370

101 285 185 -4.051

101 290 190 -5.097

101 295 195 -4.592

101 300 200 -4.046

101 190 90 -3.502

106 225 120 -3.885

106 230 125 -3.806

106 235 130 -3.672

106 255 150 -4.414

106 260 155 -5.201

106 265 160 -5.280

106 270 165 -4.649

106 275 170 -4.596

106 280 175 -4.061

106 285 180 -4.207

106 290 185 -3.948

106 300 195 -4.043

106 305 200 -3.737

111 255 145 -4.110

111 260 150 -4.347

111 265 155 -4.139

111 270 160 -4.632

111 275 165 -4.428

111 280 170 -3.982

111 300 190 -3.958

111 305 195 -3.772

111 310 200 -3.555

111 195 85 -3.517

116 255 140 -3.810

116 260 145 -4.331

116 265 150 -4.093

116 270 155 -3.918

116 275 160 -4.071

116 280 165 -3.662

116 300 185 -3.572

116 305 190 -3.913

116 315 200 -3.726

116 190 75 -3.664

121 220 100 -3.870

121 255 135 -4.011

121 260 140 -4.883

121 265 145 -4.546

121 270 150 -4.324

121 275 155 -4.511

121 280 160 -3.868

121 285 165 -3.724

121 300 180 -3.988

121 305 185 -4.233

121 320 200 -3.774

126 255 130 -3.530

126 260 135 -4.268

126 265 140 -3.763

126 270 145 -3.593

126 275 150 -3.970

126 280 155 -3.653

126 285 160 -3.538

126 300 175 -3.755

131 260 130 -3.943

131 265 135 -3.579

131 270 140 -3.795

131 275 145 -3.945

131 280 150 -3.854

131 285 155 -3.891

131 300 170 -3.582

136 260 125 -4.215

136 265 130 -3.871

136 275 140 -4.075

136 280 145 -3.533

141 260 120 -4.191

141 265 125 -4.099

141 275 135 -3.638

141 280 140 -3.582

141 315 175 -3.673

146 255 110 -3.603

146 260 115 -4.299

146 265 120 -3.869

146 270 125 -3.822

146 275 130 -3.628

146 315 170 -3.541

151 255 105 -3.556

151 260 110 -4.396

151 265 115 -4.066

151 270 120 -3.816

151 275 125 -3.949

151 280 130 -3.605

151 285 135 -3.617

151 300 150 -3.511

151 305 155 -3.683

156 255 100 -3.646

156 260 105 -4.245

156 265 110 -4.067

156 270 115 -3.734

161 260 100 -4.001

161 265 105 -3.511

161 270 110 -3.511

161 315 155 -3.525

161 320 160 -3.641

181 315 135 -3.517

186 260 75 -3.626

186 265 80 -3.725

# RPR1 (269 bp)

111 305 195 -3.596

116 260 145 -3.520

116 295 180 -3.787

121 260 140 -4.063

121 265 145 -3.723

121 270 150 -3.560

121 295 175 -3.805

121 300 180 -3.856

121 305 185 -3.792

121 310 190 -3.838

121 315 195 -4.388

121 320 200 -3.967

151 260 110 -3.827

151 315 165 -3.546

156 260 105 -4.269

156 300 145 -3.543

161 260 100 -4.822

161 265 105 -4.186

161 300 140 -4.089

161 305 145 -3.917

161 310 150 -3.661

161 315 155 -3.555

161 340 180 -3.575

161 255 95 -3.543

166 265 100 -4.181

166 270 105 -3.814

166 300 135 -4.178

166 305 140 -4.497

166 310 145 -4.135

166 315 150 -4.173

166 320 155 -3.660

166 325 160 -3.946

166 330 165 -3.615

166 340 175 -3.815

166 255 90 -3.744

166 260 95 -5.092

171 270 100 -4.019

171 300 130 -4.226

171 305 135 -4.418

171 310 140 -4.360

171 315 145 -4.037

171 320 150 -3.953

171 325 155 -3.771

171 330 160 -3.532

171 340 170 -3.581

171 255 85 -3.817

171 260 90 -5.239

171 265 95 -4.287

176 310 135 -3.508

176 260 85 -4.281

181 315 135 -3.760

181 260 80 -3.800

186 300 115 -4.024

186 305 120 -4.014

186 310 125 -3.810

186 315 130 -3.817

186 260 75 -4.765

186 265 80 -4.308

191 300 110 -3.973

191 305 115 -3.851

191 325 135 -3.544

191 265 75 -4.049

196 300 105 -4.154

196 305 110 -3.652

196 310 115 -3.863

196 315 120 -4.015

196 320 125 -3.966

196 325 130 -3.554

196 340 145 -3.503

196 270 75 -3.847

201 300 100 -4.859

201 305 105 -4.552

201 310 110 -4.419

201 315 115 -4.298

201 320 120 -3.730

201 325 125 -3.695

201 275 75 -4.598

201 295 95 -3.715

206 305 100 -4.971

206 310 105 -4.707

206 315 110 -4.076

206 325 120 -3.635

206 295 90 -3.583

206 300 95 -4.663

211 310 100 -4.036

211 315 105 -3.950

211 300 90 -4.926

211 305 95 -4.397

215 end 154 -3.690

216 315 100 -3.522

216 325 110 -3.724

216 345 130 -3.566

216 295 80 -3.508

216 300 85 -4.972

216 305 90 -4.717

216 310 95 -4.285

# RUF5-1 (710 bp)

451 565 115 -3.529

471 555 85 -3.720

476 555 80 -3.938

# SCR1 (522 bp)

26 220 195 -4.242

26 225 200 -3.706

31 155 125 -3.623

31 165 135 -4.323

31 170 140 -3.512

31 190 160 -3.961

31 195 165 -3.989

31 200 170 -3.604

36 155 120 -3.730

36 130 95 -3.642

41 145 105 -3.846

41 150 110 -3.951

41 155 115 -3.771

41 195 155 -3.698

41 130 90 -4.244

41 135 95 -3.664

366 470 105 -3.534

371 470 100 -3.502

371 465 95 -3.606

376 460 85 -3.848

376 465 90 -4.334

376 470 95 -3.931

# SNR14 (160 bps)

56 145 90 -3.524

65 end 95 -3.547

66 155 90 -4.016

70 end 90 -3.660

71 155 85 -3.900

75 end 85 -3.925

76 155 80 -4.229

81 155 75 -3.702

# SNR19 (568 bp)

321 520 200 -4.109

326 500 175 -3.627

326 515 190 -4.031

326 520 195 -3.813

371 505 135 -3.584

# SNR30 (601 bp)

136 330 195 -4.083

136 335 200 -4.959

141 330 190 -4.027

141 335 195 -4.809

141 340 200 -4.722

146 320 175 -3.768

146 325 180 -3.557

146 330 185 -4.296

146 335 190 -5.517

146 340 195 -4.648

146 345 200 -4.284

151 310 160 -3.947

151 325 175 -3.907

151 330 180 -5.053

151 335 185 -3.996

151 340 190 -3.865

151 345 195 -3.749

151 350 200 -3.852

156 310 155 -3.953

156 320 165 -3.509

156 325 170 -4.281

156 330 175 -4.699

156 335 180 -3.875

156 340 185 -3.751

161 300 140 -3.643

161 305 145 -3.892

161 310 150 -3.718

161 320 160 -4.183

166 300 135 -3.857

166 305 140 -4.341

166 310 145 -4.006

166 315 150 -3.619

166 320 155 -3.690

171 300 130 -3.782

171 305 135 -4.144

171 310 140 -4.236

171 315 145 -3.734

171 320 150 -3.513

171 325 155 -3.515

176 290 115 -3.526

176 300 125 -4.338

176 305 130 -3.927

176 310 135 -4.383

176 315 140 -3.771

176 320 145 -4.377

176 325 150 -3.719

176 335 160 -3.550

181 290 110 -4.180

181 295 115 -4.325

181 300 120 -4.003

181 305 125 -4.750

181 310 130 -5.314

181 315 135 -4.196

181 320 140 -4.054

181 330 150 -3.597

186 285 100 -3.571

186 290 105 -4.923

186 295 110 -4.205

186 305 120 -5.273

186 310 125 -5.593

186 315 130 -5.161

186 320 135 -4.597

186 325 140 -3.822

186 355 170 -3.794

191 290 100 -4.723

191 295 105 -3.823

191 300 110 -3.983

191 305 115 -3.960

191 310 120 -4.556

191 315 125 -3.635

191 280 90 -3.776

191 285 95 -4.729

196 295 100 -3.845

196 315 120 -3.755

196 320 125 -3.666

196 280 85 -4.658

196 285 90 -4.581

196 290 95 -4.189

201 315 115 -3.887

201 320 120 -3.708

201 280 80 -4.320

201 285 85 -5.004

201 290 90 -4.330

201 295 95 -3.901

206 310 105 -3.891

206 295 90 -4.278

206 300 95 -3.511

211 300 90 -3.645

386 535 150 -3.654

391 535 145 -3.753

396 530 135 -3.502

396 535 140 -3.874

396 540 145 -3.848

396 545 150 -3.598

401 525 125 -3.597

401 530 130 -3.675

406 510 105 -3.607

406 515 110 -4.212

406 520 115 -3.627

411 510 100 -3.592

421 500 80 -3.820

# SNR6 (112 bp)

1 75 75 -4.036

1 85 85 -3.706

# SNR7-L (214 bp)

1 145 145 -3.742

26 125 100 -3.559

26 130 105 -3.605

26 135 110 -3.589

36 110 75 -3.721

# SNR83 (306 bp)

21 140 120 -3.759

26 130 105 -3.896

26 135 110 -3.937

26 170 145 -3.682

26 175 150 -3.774

26 180 155 -3.557

26 100 75 -3.555

26 105 80 -3.754

31 130 100 -3.751

31 135 105 -3.847

31 140 110 -3.534

31 165 135 -3.760

31 170 140 -3.547

31 180 150 -3.705

31 105 75 -3.686

36 135 100 -4.182

36 180 145 -3.864

36 125 90 -3.563

36 130 95 -4.191

41 140 100 -3.647

41 165 125 -3.531

41 180 140 -4.288

41 120 80 -3.518

41 125 85 -3.719

41 130 90 -4.506

41 135 95 -4.300

46 120 75 -3.991

46 125 80 -3.506

# TLC1 (1301 bp)

271 395 125 -3.586

271 400 130 -3.601

276 400 125 -3.562

276 420 145 -3.575

281 390 110 -4.094

281 395 115 -4.184

281 400 120 -4.464

281 405 125 -3.883

281 410 130 -4.154

281 415 135 -4.057

281 420 140 -3.677

281 430 150 -3.506

281 445 165 -3.796

286 390 105 -3.675

286 395 110 -3.750

286 400 115 -4.558

286 405 120 -4.241

286 410 125 -4.087

286 415 130 -4.181

286 420 135 -4.023

286 425 140 -3.609

286 430 145 -3.661

286 445 160 -3.771

291 395 105 -3.617

291 400 110 -3.879

291 405 115 -3.715

291 410 120 -3.551

291 415 125 -3.642

291 420 130 -3.586

306 455 150 -3.633

311 410 100 -4.048

311 415 105 -4.209

311 420 110 -3.730

311 425 115 -3.925

311 430 120 -3.962

311 435 125 -3.704

311 440 130 -3.762

311 445 135 -3.579

311 450 140 -3.772

311 395 85 -3.795

311 400 90 -4.105

311 405 95 -3.659

316 415 100 -4.346

316 420 105 -3.872

316 425 110 -3.860

316 430 115 -3.825

316 435 120 -3.597

316 440 125 -3.855

316 445 130 -3.967

316 450 135 -4.070

316 395 80 -3.775

316 400 85 -4.030

316 405 90 -4.171

316 410 95 -3.871

321 420 100 -4.372

321 425 105 -4.171

321 430 110 -4.154

321 435 115 -4.362

321 440 120 -4.331

321 445 125 -4.535

321 450 130 -4.533

321 455 135 -3.978

321 400 80 -4.015

321 405 85 -3.914

321 410 90 -4.875

321 415 95 -4.470

326 425 100 -4.927

326 430 105 -4.905

326 435 110 -4.510

326 440 115 -4.716

326 445 120 -4.635

326 450 125 -4.296

326 455 130 -3.883

326 520 195 -3.927

326 400 75 -4.828

326 405 80 -4.562

326 410 85 -5.145

326 415 90 -5.461

326 420 95 -5.361

331 430 100 -4.665

331 435 105 -4.568

331 440 110 -4.483

331 445 115 -5.266

331 450 120 -4.393

331 455 125 -4.365

331 520 190 -3.938

331 405 75 -4.971

331 410 80 -5.554

331 415 85 -5.111

331 420 90 -4.733

331 425 95 -4.718

336 435 100 -4.994

336 440 105 -4.772

336 445 110 -5.314

336 450 115 -5.131

336 455 120 -4.978

336 460 125 -3.507

336 465 130 -3.712

336 470 135 -3.739

336 475 140 -3.817

336 480 145 -3.748

336 410 75 -5.812

336 415 80 -6.267

336 420 85 -5.725

336 425 90 -5.520

336 430 95 -5.630

341 440 100 -4.855

341 445 105 -4.972

341 450 110 -4.809

341 455 115 -4.040

341 460 120 -3.503

341 475 135 -3.618

341 415 75 -5.748

341 420 80 -6.029

341 425 85 -5.172

341 430 90 -5.066

341 435 95 -5.335

346 445 100 -3.972

346 450 105 -3.756

346 420 75 -4.937

346 425 80 -4.224

346 430 85 -4.075

346 435 90 -4.358

346 440 95 -4.154

506 705 200 -3.956

511 705 195 -3.870

516 705 190 -4.250

516 710 195 -3.720

516 715 200 -4.157

521 705 185 -4.662

521 710 190 -4.338

521 715 195 -4.050

521 720 200 -4.168

526 695 170 -3.516

526 700 175 -3.905

526 705 180 -4.122

531 690 160 -3.503

531 695 165 -4.093

531 700 170 -3.689

536 685 150 -3.773

536 690 155 -3.812

536 695 160 -4.015

536 700 165 -3.601

541 690 150 -3.573

546 685 140 -3.602

556 660 105 -3.625

556 670 115 -3.797

561 670 110 -4.042

561 675 115 -3.703

561 680 120 -3.588

566 670 105 -3.752

946 1125 180 -3.818

951 1110 160 -3.888

951 1115 165 -3.544

956 1110 155 -4.166

956 1115 160 -3.799

956 1120 165 -3.723

956 1125 170 -3.707

961 1105 145 -3.541

961 1110 150 -4.519

961 1115 155 -4.398

961 1120 160 -4.154

961 1125 165 -3.813

966 1105 140 -3.874

966 1110 145 -3.957

966 1120 155 -3.554

971 1105 135 -3.734

971 1120 150 -3.879

986 1085 100 -3.503

# snR49 (165 bp)

0 165 165 -4.390

0 165 165 -4.428

0 165 165 -4.125

0 165 165 -4.407

0 165 165 -4.180

0 165 165 -4.251

0 165 165 -4.453

1 150 150 -3.787

1 155 155 -3.874

1 160 160 -4.338

5 165 160 -4.321

6 150 145 -3.701

6 155 150 -4.296

6 160 155 -4.636

10 165 155 -4.294

11 150 140 -4.100

11 155 145 -3.978

11 160 150 -4.456

15 165 150 -4.799

16 150 135 -4.441

16 155 140 -4.951

16 160 145 -5.265

20 165 145 -5.197

21 150 130 -4.267

21 155 135 -3.590

21 160 140 -5.443

25 165 140 -4.930

26 150 125 -4.762

26 155 130 -3.879

26 160 135 -5.075

30 165 135 -5.609

31 145 115 -3.599

31 150 120 -5.182

31 155 125 -3.921

31 160 130 -5.800

35 165 130 -5.693

36 145 110 -3.673

36 150 115 -5.180

36 155 120 -4.245

36 160 125 -5.798

40 165 125 -5.064

41 150 110 -3.985

41 155 115 -3.674

41 160 120 -5.402

45 165 120 -5.072

46 150 105 -4.375

46 155 110 -3.975

46 160 115 -5.740

50 165 115 -5.307

51 150 100 -5.200

51 155 105 -4.338

51 160 110 -6.377

51 145 95 -3.712

55 165 110 -5.575

56 155 100 -4.206

56 160 105 -6.185

56 145 90 -3.778

56 150 95 -4.889

60 165 105 -5.800

61 160 100 -6.201

61 145 85 -3.704

61 150 90 -5.101

61 155 95 -4.990

65 165 100 -5.340

66 145 80 -4.134

66 150 85 -5.274

66 155 90 -4.847

66 160 95 -5.291

70 165 95 -3.996

71 145 75 -4.021

71 150 80 -5.681

71 155 85 -4.901

71 160 90 -4.179

76 150 75 -3.830

76 155 80 -3.722
