# Supplementary material for: Non-Coding RNA Prediction and Verification in Saccharomyces cerevisiae
Source: PLoS Genet. 2009 Jan 2;5(1):e1000321. doi: 10.1371/journal.pgen.1000321 (PMC2603021; doi:10.1371/journal.pgen.1000321)
Supplement: Table S4 — GC content of regions in the negative and positive control sets producing Z-scores ≤−3.5. (0.13 MB DOC) [file pgen.1000321.s015.doc]

Table S4. GC content of regions in the negative and positive control sets producing Z-scores ≤ -3.5.

| **Descrip-tion** | **Sequence** | **Total Length (nt)** | **Overall %GC** | **Region producing ≤ -3.5 (nt)** | **Length Region ≤ -3.5 (nt)** | **Min Z-score in region** | **%GC** |
| --- | --- | --- | --- | --- | --- | --- | --- |
|  | Random9 | 300 | 41.7 | 61-285 | 225 | -4.522 | 39.6 |
|  | Random13 | 300 | 41.0 | 21-95 | 75 | -3.502 | 45.3 |
|  | PTP1-SSB1 intergenic | 300 | 28.2 | 1-190 | 190 | -4.749 | 29.5 |
| Negative Control | LSR1 shuffled | 1175 | 40.9 | 491-570 | 80 | -3.611 | 33.8 |
| Set | NME1 shuffled | 339 | 38.9 | 1-235 | 235 | -5.844 | 38.3 |
|  | RUF5-1 shuffled | 710 | 34.1 | 271-355 | 85 | -3.563 | 28.2 |
|  | snR19 shuffled | 568 | 39.8 | 411-485 | 75 | -3.682 | 44.0 |
|  | TLC1 shuffled | 1301 | 35.6 | 366-580 | 215 | -4.916 | 34.0 |
|  | LSR1 | 1175 | 40.9 | 496-735 | 240 | -6.783 | 43.3 |
|  | NME1 | 339 | 39.1 | 21-320 | 300 | -5.201 | 38.0 |
|  | RPR1 | 369 | 51.5 | 111-340 | 230 | -5.239 | 53.9 |
|  | RUF5-1 | 710 | 34.1 | 451-565 | 115 | -3.938 | 40.0 |
| Positive Control | SCR1 | 522 | 55.0 | 26-225  366-470 | 200  105 | -4.323  -4.334 | 57.0  52.4 |
| Set | snR14 | 160 | 38.8 | 56-160 | 105 | -4.229 | 36.2 |
|  | snR19 | 568 | 39.8 | 321-520 | 200 | -4.109 | 38.5 |
|  | snR30 | 601 | 46.7 | 136-355  386-535 | 220  150 | -5.593  -4.212 | 49.6  44.7 |
|  | snR6 | 112 | 39.3 | 1-85 | 85 | -4.036 | 44.7 |
|  | snR7-L | 214 | 44.4 | 1-145 | 145 | -3.742 | 44.1 |
|  | snR83 | 306 | 35.6 | 21-180 | 160 | -4.506 | 39.4 |
|  | TLC1 | 1301 | 35.6 | 271-520  506-720  946-1125 | 250  215  180 | -6.267  -4.662  -4.519 | 35.2  34.0  33.3 |
|  | snR49 | 165 | 33.9 | 1-165 | 165 | -6.377 | 33.9 |
