# Supplementary material for: Non-Coding RNA Prediction and Verification in Saccharomyces cerevisiae
Source: PLoS Genet. 2009 Jan 2;5(1):e1000321. doi: 10.1371/journal.pgen.1000321 (PMC2603021; doi:10.1371/journal.pgen.1000321)
Supplement: Table S5 — ncRNA embedded in longer sequence. The GenBank accession numbers and descriptions for sequences used for the embedded ncRNA analysis. The column labeled Z-score provides the Z-score that is computed when the exact tRNA length is used. (0.07 MB DOC) [file pgen.1000321.s016.doc]

Table S5. ncRNA embedded in longer sequence. The GenBank accession numbers and descriptions for sequences used for the embedded ncRNA analysis. The column labeled Z-score provides the Z-score that is computed when the exact tRNA length is used.

| **Species** | **tRNA** | **Length (nt)** | **Z-score** | **mRNA** | **Species** | **Embedded location** |
| --- | --- | --- | --- | --- | --- | --- |
| *S. cerevisiae* | K00228.1 | 82 | -4.3 | AF452886  (22-270) | *Ornithodoros savignyi* | 170-246 |
| *Chlamydomonas moewusii* | X51398.1 | 74 | -4.2 | NM_052872  (347-676) | *Homo sapiens* | 164-237 |
| *Drosophila melanogaster* | AC002512.1 | 73 | -4.1 | NM_177233  (423-831) | *Mus musculus* | 77-149 |
| *Gallus gallus* | X52392.1 | 68 | -4.3 | AY182163  (1-225) | *Trigonella foenum-graecum* | 174-240 |
| *Xenopus laevis* | M10217.1 | 69 | -4.3 | NM_145157  66-317 | *Mus musculus* | 241-309 |
| *E. coli* | AE005527.1 | 73 | -4.2 | AY555511  (1-409) | *Ictalurus furcatus* | 244-316 |
| *Bacillus halodurans* | AB031211.1 | 85 | -4.9 | NM_001003967  (1-400) | *Canis lupus familiaris* | 165-250 |
| *Gallus gallus* | AF076356.1 | 69 | -4.1 | NM_001003966.1 (1-366) | *Canis lupus familiaris* | 117-185 |
| *Salmonella typhimurium* | AE008848.1 | 91 | -4.2 | NM_001024644.1  (1-210) | *Homo sapiens* | 91-163 |
| *Emericella nidulans* | J01391.1 | 71 | -4.2 | NM_131070  (131-571) | *Danio rerio* | 8-78 |
