# Supplementary material for: Non-Coding RNA Prediction and Verification in Saccharomyces cerevisiae
Source: PLoS Genet. 2009 Jan 2;5(1):e1000321. doi: 10.1371/journal.pgen.1000321 (PMC2603021; doi:10.1371/journal.pgen.1000321)
Supplement: Table S6 — Percent nucleotide identity in syntenic regions of S. cerevisiae and S. bayanus. The “needle” program contained in the EMBOSS package was used to align intergenic regions and compute the percent identity [71]. A gap open penalty of 10.0 and a gap extend penalty of 0.5 was used to perform the alignment. It is important to note that many of the syntenic regions between the two species differ in length. (0.16 MB DOC) [file pgen.1000321.s017.doc]

Table S6. Percent nucleotide identity in syntenic regions of *S. cerevisiae* and *S. bayanus*. The “needle” program contained in the EMBOSS package was used to align intergenic regions and compute the percent identity [71]. A gap open penalty of 10.0 and a gap extend penalty of 0.5 was used to perform the alignment. It is important to note that many of the syntenic regions between the two species differ in length.

|  | **Intergenic region** | **Number identical nucleotides/total length** | **Percent Identity** |
| --- | --- | --- | --- |
| 1 | YFL002C--YFL001 | 138/278 | 49.60% |
| 2 | YFL004W--YFL003C | 142/276 | 51.40% |
| 3 | YFL005W--YFL004W | 591/948 | 62.30% |
| 4 | YFL009W--YFL008W | 306/972 | 31.50% |
| 5 | YFL010C--YFL009W | 310/432 | 71.80% |
| 6 | YFL012W--YFL011W | 628/1410 | 44.50% |
| 7 | YFL013C--YFL012W | 459/839 | 54.70% |
| 8 | YFL014W--YFL013C | 180/299 | 60.20% |
| 9 | YFL016C--YFL014W | 703/1177 | 59.70% |
| 10 | YFL017C--YFL016C | 176/272 | 64.70% |
| 11 | YFL018C--YFL017W-A | 365/637 | 57.30% |
| 12 | YFL023W--YFL022C | 75/147 | 51.00% |
| 13 | YFL025C--YFL024C | 435/688 | 63.20% |
| 14 | YFL026W--YFL025C | 147/332 | 44.30% |
| 15 | YFL027C--YFL026W | 318/757 | 42.00% |
| 16 | YFL028C--YFL027C | 143/224 | 63.80% |
| 17 | YFL029C--YFL028C | 129/196 | 65.80% |
| 18 | YFL031W--YFL030W | 452/997 | 45.30% |
| 19 | YFL033C--YFL031W | 512/887 | 57.70% |
| 20 | YFL034W--YFL033C | 284/583 | 48.70% |
| 21 | YFL036W--YFL034C-B | 129/240 | 53.80% |
| 22 | YFL037W--YFL036W | 687/1265 | 54.30% |
| 23 | YFL038C--YFL037W | 274/358 | 76.50% |
| 24 | YFL039C--YFL038C | 528/1165 | 45.30% |
| 25 | YFL040W--YFL039C | 229/302 | 75.80% |
| 26 | YFL041W--YFL040W | 231/363 | 63.60% |
| 27 | YFL044C--YFL042C | 123/172 | 71.50% |
| 28 | YFL045C--YFL044C | 205/280 | 73.20% |
| 29 | YFL046W--YFL045C | 143/200 | 71.50% |
| 30 | YFL047W--YFL046W | 158/271 | 58.30% |
| 31 | YFL048C--YFL047W | 137/268 | 51.10% |
| 32 | YFL049W--YFL048C | 127/190 | 66.80% |
| 33 | YFL050C--YFL049W | 620/1186 | 52.30% |
| 34 | YFL051C--YFL050C | 1708/3843 | 44.40% |
| 35 | YFL054C--YFL053W | 402/1144 | 35.10% |
| 36 | YFR001W--YFR002W | 187/330 | 56.70% |
| 37 | YFR002W--YFR003C | 92/159 | 57.90% |
| 38 | YFR003C--YFR004W | 189/292 | 64.70% |
| 39 | YFR004W--YFR005C | 131/250 | 52.40% |
| 40 | YFR005C--YFR006W | 198/289 | 68.50% |
| 41 | YFR007W--YFR008W | 111/202 | 55.00% |
| 42 | YFR009W--YFR010W | 229/352 | 65.10% |
| 43 | YFR010W--YFR011C | 141/209 | 67.50% |
| 44 | YFR013W--YFR014C | 175/287 | 61.00% |
| 45 | YFR014C--YFR015C | 265/441 | 60.10% |
| 46 | YFR015C--YFR016C | 430/764 | 56.30% |
| 47 | YFR017C--YFR018C | 193/317 | 60.90% |
| 48 | YFR021W--YFR022W | 385/551 | 69.90% |
| 49 | YFR022W--YFR023W | 544/1090 | 49.90% |
| 50 | YFR027W--YFR028C | 70/116 | 60.30% |
| 51 | YFR031C-A--YFR032C | 424/1562 | 27.10% |
| 52 | YFR031C--YFR031C-A | 287/446 | 64.30% |
| 53 | YFR032C--YFR032C-A | 54/300 | 18.00% |
| 54 | YFR036W--YFR037C | 65/175 | 37.10% |
| 55 | YFR039C--YFR040W | 506/755 | 67.00% |
| 56 | YFR040W--YFR041C | 99/134 | 73.90% |
| 57 | YFR041C--YFR042W | 144/222 | 64.90% |
| 58 | YFR043C--YFR044C | 109/186 | 58.60% |
| 59 | YFR044C--YFR045W | 390/771 | 50.60% |
| 60 | YFR046C--YFR047C | 86/127 | 67.70% |
| 61 | YFR047C--YFR048W | 518/1163 | 44.50% |
| 62 | YFR048W--YFR049W | 293/420 | 69.80% |
| 63 | YFR049W--YFR050C | 115/195 | 59.00% |
| 64 | YFR050C--YFR051C | 196/352 | 55.70% |
| 65 | YFR051C--YFR052W | 436/861 | 50.60% |
| 66 | YFR052W--YFR053C | 189/318 | 59.40% |
|  |  | Average: | 56.93% |
