# Supplementary material for: Non-Coding RNA Prediction and Verification in Saccharomyces cerevisiae
Source: PLoS Genet. 2009 Jan 2;5(1):e1000321. doi: 10.1371/journal.pgen.1000321 (PMC2603021; doi:10.1371/journal.pgen.1000321)
Supplement: Table S7 — All Z-score values for Watson strand of intergenic region between SEC4 and VTC2. The table provides the Z-score calculated for each position of the intergenic region for each window size (75 nt to 200 nt). The first column provides the sequence name. This region lies between SEC4 (YFL005W) and VTC2 (YFL004W) and is 828 bp long. The sequence name ends with the boundary values for the window being evaluated. The second column (Pos) specifies the beginning position of the window. The 3rd column (Length) gives the length of the window. The 4th column (MFE) gives the minimum folding energy of the native sequence. The 5th column (#Shuffles) gives the number of shuffled sequence used to generate a mean and standard deviation. The 6th column (Mean) gives the mean of the distribution of minimum folding energies for the shuffled sequences. The 7th column (Std. dev) gives the standard deviation for the distribution of minimum folding energies of the shuffled sequences. The 8th column (Z-score) gives the Z-score for the window being evaluated. (0.67 MB DOC) [file pgen.1000321.s018.doc]

Table S7. All Z-score values for Watson strand of intergenic region between *SEC4* and *VTC2*. The table provides the Z-score calculated for each position of the intergenic region for each window size (75nt to 200nt). The first column provides the sequence name. This region lies between *SEC4* (*YFL005W*) and *VTC2* (*YFL004W*) and is 828bp long. The sequence name ends with the boundary values for the window being evaluated. The second column (Pos) specifies the beginning position of the window. The 3^rd^ column (Length) gives the length of the window. The 4^th^ column (MFE) gives the minimum folding energy of the native sequence. The 5^th^ column (#Shuffles) gives the number of shuffled sequence used to generate a mean and standard deviation. The 6^th^ column (Mean) gives the mean of the distribution of minimum folding energies for the shuffled sequences. The 7^th^ column (Std. dev) gives the standard deviation for the distribution of minimum folding energies of the shuffled sequences. The 8^th^ column (Z-score) gives the Z-score for the window being evaluated.

> Sequence Name Pos Length MFE #Shuffles Mean Std. dev. Z-score

Window size = 75

YFL005W-YFL004W_1_75 1 75 -12.700 500 -12.749 2.680 0.018

YFL005W-YFL004W_6_80 6 75 -11.200 500 -11.853 2.369 0.276

YFL005W-YFL004W_11_85 11 75 -8.500 500 -9.693 2.353 0.507

YFL005W-YFL004W_16_90 16 75 -15.000 500 -10.497 2.329 -1.933

YFL005W-YFL004W_21_95 21 75 -15.000 500 -9.752 2.396 -2.190

YFL005W-YFL004W_26_100 26 75 -9.350 500 -11.380 2.562 0.792

YFL005W-YFL004W_31_105 31 75 -11.150 500 -9.957 2.378 -0.502

YFL005W-YFL004W_36_110 36 75 -9.450 500 -9.677 2.419 0.094

YFL005W-YFL004W_41_115 41 75 -8.200 500 -8.348 2.205 0.067

YFL005W-YFL004W_46_120 46 75 -5.400 500 -5.595 2.089 0.093

YFL005W-YFL004W_51_125 51 75 -3.200 500 -4.025 1.883 0.438

YFL005W-YFL004W_56_130 56 75 -2.300 500 -3.393 1.680 0.651

YFL005W-YFL004W_61_135 61 75 -3.600 500 -3.640 1.790 0.022

YFL005W-YFL004W_66_140 66 75 -3.600 500 -2.231 1.493 -0.917

YFL005W-YFL004W_71_145 71 75 -4.500 500 -2.749 1.595 -1.098

YFL005W-YFL004W_76_150 76 75 -4.500 500 -3.245 1.535 -0.818

YFL005W-YFL004W_81_155 81 75 -4.500 500 -3.581 1.686 -0.545

YFL005W-YFL004W_86_160 86 75 -4.400 500 -4.442 1.888 0.022

YFL005W-YFL004W_91_165 91 75 -4.090 500 -3.571 1.668 -0.311

YFL005W-YFL004W_96_170 96 75 -4.090 500 -4.491 2.028 0.198

YFL005W-YFL004W_101_175 101 75 -3.600 500 -3.325 1.843 -0.149

YFL005W-YFL004W_106_180 106 75 -3.600 500 -4.225 1.989 0.314

YFL005W-YFL004W_111_185 111 75 -5.500 500 -6.634 2.068 0.548

YFL005W-YFL004W_116_190 116 75 -6.000 500 -5.795 2.079 -0.099

YFL005W-YFL004W_121_195 121 75 -6.000 500 -5.486 1.901 -0.270

YFL005W-YFL004W_126_200 126 75 -6.000 500 -4.933 1.883 -0.567

YFL005W-YFL004W_131_205 131 75 -5.400 500 -4.420 1.818 -0.539

YFL005W-YFL004W_136_210 136 75 -5.400 500 -4.209 1.799 -0.662

YFL005W-YFL004W_141_215 141 75 -5.200 500 -4.632 1.887 -0.301

YFL005W-YFL004W_146_220 146 75 -3.900 500 -3.026 1.657 -0.528

YFL005W-YFL004W_151_225 151 75 -3.900 500 -3.272 1.764 -0.356

YFL005W-YFL004W_156_230 156 75 -3.800 500 -3.149 1.735 -0.375

YFL005W-YFL004W_161_235 161 75 -3.800 500 -2.507 1.618 -0.799

YFL005W-YFL004W_166_240 166 75 -3.800 500 -2.853 1.783 -0.532

YFL005W-YFL004W_171_245 171 75 -3.800 500 -1.696 1.449 -1.452

YFL005W-YFL004W_176_250 176 75 -5.400 500 -3.550 1.817 -1.018

YFL005W-YFL004W_181_255 181 75 -1.900 500 -2.250 1.659 0.211

YFL005W-YFL004W_186_260 186 75 -1.900 500 -2.077 1.463 0.121

YFL005W-YFL004W_191_265 191 75 -5.800 500 -4.360 1.844 -0.780

YFL005W-YFL004W_196_270 196 75 -7.100 500 -6.419 2.042 -0.334

YFL005W-YFL004W_201_275 201 75 -7.100 500 -6.561 2.125 -0.253

YFL005W-YFL004W_206_280 206 75 -7.100 500 -8.537 2.204 0.652

YFL005W-YFL004W_211_285 211 75 -7.100 500 -9.477 2.288 1.039

YFL005W-YFL004W_216_290 216 75 -8.600 500 -11.339 2.384 1.149

YFL005W-YFL004W_221_295 221 75 -9.600 500 -10.548 2.459 0.385

YFL005W-YFL004W_226_300 226 75 -9.900 500 -12.170 2.699 0.841

YFL005W-YFL004W_231_305 231 75 -9.600 500 -12.579 2.406 1.238

YFL005W-YFL004W_236_310 236 75 -9.600 500 -11.636 2.620 0.777

YFL005W-YFL004W_241_315 241 75 -7.600 500 -10.110 2.577 0.974

YFL005W-YFL004W_246_320 246 75 -8.100 500 -10.953 2.557 1.115

YFL005W-YFL004W_251_325 251 75 -8.900 500 -10.326 2.494 0.572

YFL005W-YFL004W_256_330 256 75 -8.100 500 -8.765 2.366 0.281

YFL005W-YFL004W_261_335 261 75 -5.800 500 -7.040 1.939 0.639

YFL005W-YFL004W_266_340 266 75 -5.800 500 -8.869 2.268 1.353

YFL005W-YFL004W_271_345 271 75 -4.720 500 -9.199 2.404 1.863

YFL005W-YFL004W_276_350 276 75 -11.700 500 -10.884 2.541 -0.321

YFL005W-YFL004W_281_355 281 75 -12.800 500 -11.163 2.641 -0.620

YFL005W-YFL004W_286_360 286 75 -13.030 500 -10.297 2.548 -1.073

YFL005W-YFL004W_291_365 291 75 -15.200 500 -11.350 2.463 -1.563

YFL005W-YFL004W_296_370 296 75 -15.700 500 -11.620 2.603 -1.568

YFL005W-YFL004W_301_375 301 75 -16.900 500 -11.198 2.505 -2.276

YFL005W-YFL004W_306_380 306 75 -17.600 500 -11.628 2.692 -2.218

YFL005W-YFL004W_311_385 311 75 -15.800 500 -11.948 2.663 -1.446

YFL005W-YFL004W_316_390 316 75 -16.600 500 -11.885 2.515 -1.874

YFL005W-YFL004W_321_395 321 75 -16.600 500 -11.629 2.420 -2.054

YFL005W-YFL004W_326_400 326 75 -15.900 500 -9.443 2.397 -2.694

YFL005W-YFL004W_331_405 331 75 -15.900 500 -9.467 2.345 -2.743

YFL005W-YFL004W_336_410 336 75 -14.200 500 -7.087 2.051 -3.468

YFL005W-YFL004W_341_415 341 75 -7.700 500 -6.885 2.164 -0.377

YFL005W-YFL004W_346_420 346 75 -7.700 500 -6.801 2.156 -0.417

YFL005W-YFL004W_351_425 351 75 -7.300 500 -6.243 2.120 -0.499

YFL005W-YFL004W_356_430 356 75 -5.500 500 -6.624 2.134 0.527

YFL005W-YFL004W_361_435 361 75 -5.700 500 -7.210 2.276 0.663

YFL005W-YFL004W_366_440 366 75 -7.300 500 -6.658 2.011 -0.320

YFL005W-YFL004W_371_445 371 75 -11.100 500 -7.535 2.242 -1.590

YFL005W-YFL004W_376_450 376 75 -11.100 500 -8.039 2.353 -1.301

YFL005W-YFL004W_381_455 381 75 -8.900 500 -7.941 2.333 -0.411

YFL005W-YFL004W_386_460 386 75 -6.700 500 -6.779 2.103 0.038

YFL005W-YFL004W_391_465 391 75 -5.200 500 -7.391 2.258 0.971

YFL005W-YFL004W_396_470 396 75 -5.200 500 -7.654 2.213 1.109

YFL005W-YFL004W_401_475 401 75 -8.400 500 -9.416 2.297 0.442

YFL005W-YFL004W_406_480 406 75 -8.400 500 -10.406 2.298 0.873

YFL005W-YFL004W_411_485 411 75 -9.300 500 -10.642 2.362 0.568

YFL005W-YFL004W_416_490 416 75 -9.900 500 -13.066 2.576 1.229

YFL005W-YFL004W_421_495 421 75 -10.000 500 -12.114 2.658 0.795

YFL005W-YFL004W_426_500 426 75 -8.900 500 -10.343 2.422 0.596

YFL005W-YFL004W_431_505 431 75 -8.600 500 -9.282 2.324 0.294

YFL005W-YFL004W_436_510 436 75 -8.300 500 -9.288 2.199 0.449

YFL005W-YFL004W_441_515 441 75 -7.600 500 -8.534 2.331 0.401

YFL005W-YFL004W_446_520 446 75 -7.600 500 -8.419 2.357 0.348

YFL005W-YFL004W_451_525 451 75 -8.900 500 -9.900 2.360 0.424

YFL005W-YFL004W_456_530 456 75 -8.900 500 -11.335 2.191 1.111

YFL005W-YFL004W_461_535 461 75 -8.900 500 -12.482 2.615 1.370

YFL005W-YFL004W_466_540 466 75 -8.000 500 -11.077 2.378 1.294

YFL005W-YFL004W_471_545 471 75 -7.700 500 -11.620 2.389 1.641

YFL005W-YFL004W_476_550 476 75 -10.200 500 -11.527 2.522 0.526

YFL005W-YFL004W_481_555 481 75 -12.700 500 -12.647 2.610 -0.020

YFL005W-YFL004W_486_560 486 75 -12.500 500 -11.946 2.683 -0.206

YFL005W-YFL004W_491_565 491 75 -12.000 500 -10.909 2.617 -0.417

YFL005W-YFL004W_496_570 496 75 -13.400 500 -11.363 2.667 -0.764

YFL005W-YFL004W_501_575 501 75 -15.800 500 -11.183 2.352 -1.963

YFL005W-YFL004W_506_580 506 75 -15.800 500 -11.629 2.318 -1.799

YFL005W-YFL004W_511_585 511 75 -15.800 500 -12.170 2.581 -1.407

YFL005W-YFL004W_516_590 516 75 -19.300 500 -14.535 2.654 -1.795

YFL005W-YFL004W_521_595 521 75 -18.700 500 -14.416 2.541 -1.686

YFL005W-YFL004W_526_600 526 75 -16.800 500 -15.272 2.665 -0.573

YFL005W-YFL004W_531_605 531 75 -15.600 500 -13.535 2.861 -0.722

YFL005W-YFL004W_536_610 536 75 -15.600 500 -14.151 2.690 -0.539

YFL005W-YFL004W_541_615 541 75 -15.400 500 -15.580 2.831 0.063

YFL005W-YFL004W_546_620 546 75 -21.100 500 -13.816 2.589 -2.813

YFL005W-YFL004W_551_625 551 75 -18.800 500 -11.753 2.610 -2.700

YFL005W-YFL004W_556_630 556 75 -18.800 500 -11.954 2.689 -2.545

YFL005W-YFL004W_561_635 561 75 -18.800 500 -12.127 2.652 -2.516

YFL005W-YFL004W_566_640 566 75 -19.800 500 -12.226 2.624 -2.886

YFL005W-YFL004W_571_645 571 75 -12.300 500 -11.620 2.480 -0.274

YFL005W-YFL004W_576_650 576 75 -11.430 500 -11.513 2.452 0.034

YFL005W-YFL004W_581_655 581 75 -9.030 500 -9.721 2.370 0.292

YFL005W-YFL004W_586_660 586 75 -8.930 500 -9.539 2.342 0.260

YFL005W-YFL004W_591_665 591 75 -11.100 500 -8.980 2.385 -0.889

YFL005W-YFL004W_596_670 596 75 -6.030 500 -5.893 2.041 -0.067

YFL005W-YFL004W_601_675 601 75 -6.030 500 -5.411 1.874 -0.330

YFL005W-YFL004W_606_680 606 75 -6.030 500 -5.084 1.819 -0.520

YFL005W-YFL004W_611_685 611 75 -6.430 500 -7.219 2.337 0.338

YFL005W-YFL004W_616_690 616 75 -8.000 500 -8.418 2.284 0.183

YFL005W-YFL004W_621_695 621 75 -8.030 500 -8.433 2.264 0.178

YFL005W-YFL004W_626_700 626 75 -7.930 500 -8.721 2.326 0.340

YFL005W-YFL004W_631_705 631 75 -6.700 500 -5.941 2.011 -0.377

YFL005W-YFL004W_636_710 636 75 -6.700 500 -5.723 1.929 -0.507

YFL005W-YFL004W_641_715 641 75 -6.700 500 -6.100 1.914 -0.313

YFL005W-YFL004W_646_720 646 75 -8.000 500 -9.743 2.318 0.752

YFL005W-YFL004W_651_725 651 75 -8.000 500 -9.590 2.455 0.648

YFL005W-YFL004W_656_730 656 75 -8.900 500 -11.394 2.382 1.047

YFL005W-YFL004W_661_735 661 75 -12.300 500 -13.316 2.672 0.380

YFL005W-YFL004W_666_740 666 75 -15.200 500 -14.415 2.625 -0.299

YFL005W-YFL004W_671_745 671 75 -16.400 500 -15.510 2.588 -0.344

YFL005W-YFL004W_676_750 676 75 -13.000 500 -13.259 2.488 0.104

YFL005W-YFL004W_681_755 681 75 -12.800 500 -14.744 2.733 0.711

YFL005W-YFL004W_686_760 686 75 -11.900 500 -13.008 2.698 0.411

YFL005W-YFL004W_691_765 691 75 -15.100 500 -12.295 2.646 -1.060

YFL005W-YFL004W_696_770 696 75 -14.100 500 -10.010 2.465 -1.659

YFL005W-YFL004W_701_775 701 75 -11.400 500 -8.861 2.415 -1.051

YFL005W-YFL004W_706_780 706 75 -6.720 500 -7.190 2.118 0.222

YFL005W-YFL004W_711_785 711 75 -6.320 500 -4.143 1.685 -1.292

YFL005W-YFL004W_716_790 716 75 -3.920 500 -3.116 1.534 -0.524

YFL005W-YFL004W_721_795 721 75 -3.900 500 -2.075 1.379 -1.323

YFL005W-YFL004W_726_800 726 75 -3.120 500 -2.642 1.609 -0.297

YFL005W-YFL004W_731_805 731 75 -5.700 500 -3.404 1.714 -1.339

YFL005W-YFL004W_736_810 736 75 -5.800 500 -4.279 1.871 -0.813

YFL005W-YFL004W_741_815 741 75 -5.600 500 -4.470 1.997 -0.566

YFL005W-YFL004W_746_820 746 75 -4.500 500 -2.706 1.619 -1.108

YFL005W-YFL004W_751_825 751 75 -4.500 500 -4.316 1.739 -0.106

YFL005W-YFL004W_755_828 755 73 -5.100 500 -5.670 2.030 0.281

Window size = 80

YFL005W-YFL004W_1_80 1 80 -13.700 500 -13.639 2.512 -0.024

YFL005W-YFL004W_6_85 6 80 -11.200 500 -12.759 2.550 0.612

YFL005W-YFL004W_11_90 11 80 -15.000 500 -10.681 2.436 -1.773

YFL005W-YFL004W_16_95 16 80 -15.000 500 -10.673 2.596 -1.667

YFL005W-YFL004W_21_100 21 80 -15.000 500 -12.813 2.722 -0.804

YFL005W-YFL004W_26_105 26 80 -11.350 500 -11.610 2.609 0.100

YFL005W-YFL004W_31_110 31 80 -11.150 500 -10.263 2.528 -0.351

YFL005W-YFL004W_36_115 36 80 -9.500 500 -9.565 2.519 0.026

YFL005W-YFL004W_41_120 41 80 -9.300 500 -8.664 2.406 -0.264

YFL005W-YFL004W_46_125 46 80 -5.400 500 -5.766 2.137 0.171

YFL005W-YFL004W_51_130 51 80 -3.200 500 -4.792 1.920 0.829

YFL005W-YFL004W_56_135 56 80 -3.950 500 -4.126 1.897 0.093

YFL005W-YFL004W_61_140 61 80 -3.600 500 -3.256 1.666 -0.206

YFL005W-YFL004W_66_145 66 80 -4.500 500 -3.447 1.666 -0.632

YFL005W-YFL004W_71_150 71 80 -4.500 500 -3.152 1.591 -0.847

YFL005W-YFL004W_76_155 76 80 -4.500 500 -3.635 1.681 -0.515

YFL005W-YFL004W_81_160 81 80 -4.500 500 -3.982 1.799 -0.288

YFL005W-YFL004W_86_165 86 80 -4.400 500 -4.101 1.904 -0.157

YFL005W-YFL004W_91_170 91 80 -4.090 500 -4.126 1.923 0.018

YFL005W-YFL004W_96_175 96 80 -4.800 500 -5.633 2.037 0.409

YFL005W-YFL004W_101_180 101 80 -3.600 500 -4.164 1.802 0.313

YFL005W-YFL004W_106_185 106 80 -5.500 500 -5.910 2.132 0.193

YFL005W-YFL004W_111_190 111 80 -6.000 500 -6.524 2.154 0.243

YFL005W-YFL004W_116_195 116 80 -6.000 500 -6.381 2.252 0.169

YFL005W-YFL004W_121_200 121 80 -6.000 500 -5.623 1.786 -0.211

YFL005W-YFL004W_126_205 126 80 -6.000 500 -5.552 2.089 -0.214

YFL005W-YFL004W_131_210 131 80 -5.400 500 -4.509 1.879 -0.474

YFL005W-YFL004W_136_215 136 80 -6.100 500 -4.501 1.869 -0.856

YFL005W-YFL004W_141_220 141 80 -5.200 500 -4.697 1.902 -0.264

YFL005W-YFL004W_146_225 146 80 -3.900 500 -2.918 1.760 -0.558

YFL005W-YFL004W_151_230 151 80 -3.900 500 -3.117 1.660 -0.472

YFL005W-YFL004W_156_235 156 80 -3.800 500 -2.585 1.562 -0.778

YFL005W-YFL004W_161_240 161 80 -3.800 500 -3.160 1.729 -0.370

YFL005W-YFL004W_166_245 166 80 -3.800 500 -2.925 1.709 -0.512

YFL005W-YFL004W_171_250 171 80 -5.700 500 -3.747 1.733 -1.127

YFL005W-YFL004W_176_255 176 80 -5.400 500 -4.066 1.849 -0.722

YFL005W-YFL004W_181_260 181 80 -1.900 500 -2.831 1.622 0.574

YFL005W-YFL004W_186_265 186 80 -5.800 500 -4.419 1.857 -0.744

YFL005W-YFL004W_191_270 191 80 -7.100 500 -6.412 2.093 -0.329

YFL005W-YFL004W_196_275 196 80 -7.100 500 -6.317 2.063 -0.379

YFL005W-YFL004W_201_280 201 80 -7.100 500 -8.370 2.151 0.590

YFL005W-YFL004W_206_285 206 80 -7.100 500 -9.637 2.308 1.099

YFL005W-YFL004W_211_290 211 80 -8.600 500 -11.528 2.366 1.237

YFL005W-YFL004W_216_295 216 80 -9.600 500 -10.741 2.485 0.459

YFL005W-YFL004W_221_300 221 80 -9.900 500 -12.616 2.650 1.025

YFL005W-YFL004W_226_305 226 80 -13.300 500 -13.479 2.796 0.064

YFL005W-YFL004W_231_310 231 80 -9.600 500 -13.125 2.503 1.408

YFL005W-YFL004W_236_315 236 80 -9.600 500 -10.895 2.282 0.567

YFL005W-YFL004W_241_320 241 80 -11.100 500 -11.763 2.700 0.245

YFL005W-YFL004W_246_325 246 80 -11.300 500 -11.807 2.668 0.190

YFL005W-YFL004W_251_330 251 80 -9.200 500 -10.666 2.483 0.590

YFL005W-YFL004W_256_335 256 80 -8.100 500 -10.052 2.384 0.819

YFL005W-YFL004W_261_340 261 80 -8.600 500 -10.415 2.347 0.773

YFL005W-YFL004W_266_345 266 80 -5.800 500 -10.119 2.565 1.684

YFL005W-YFL004W_271_350 271 80 -11.700 500 -11.815 2.570 0.045

YFL005W-YFL004W_276_355 276 80 -13.600 500 -12.719 2.721 -0.324

YFL005W-YFL004W_281_360 281 80 -14.100 500 -12.615 2.732 -0.543

YFL005W-YFL004W_286_365 286 80 -15.730 500 -11.313 2.490 -1.774

YFL005W-YFL004W_291_370 291 80 -15.700 500 -11.587 2.438 -1.687

YFL005W-YFL004W_296_375 296 80 -16.900 500 -11.894 2.654 -1.886

YFL005W-YFL004W_301_380 301 80 -17.600 500 -12.332 2.747 -1.918

YFL005W-YFL004W_306_385 306 80 -17.600 500 -12.327 2.549 -2.069

YFL005W-YFL004W_311_390 311 80 -16.600 500 -12.381 2.489 -1.695

YFL005W-YFL004W_316_395 316 80 -22.600 500 -12.594 2.596 -3.854

YFL005W-YFL004W_321_400 321 80 -16.600 500 -11.675 2.433 -2.024

YFL005W-YFL004W_326_405 326 80 -19.500 500 -10.055 2.502 -3.774

YFL005W-YFL004W_331_410 331 80 -15.900 500 -9.702 2.314 -2.679

YFL005W-YFL004W_336_415 336 80 -14.200 500 -7.600 2.032 -3.248

YFL005W-YFL004W_341_420 341 80 -7.700 500 -6.918 2.200 -0.356

YFL005W-YFL004W_346_425 346 80 -9.100 500 -9.446 2.423 0.143

YFL005W-YFL004W_351_430 351 80 -9.100 500 -7.383 2.208 -0.778

YFL005W-YFL004W_356_435 356 80 -7.200 500 -7.568 2.270 0.162

YFL005W-YFL004W_361_440 361 80 -8.900 500 -7.623 2.219 -0.575

YFL005W-YFL004W_366_445 366 80 -11.100 500 -7.866 2.230 -1.450

YFL005W-YFL004W_371_450 371 80 -11.100 500 -8.029 2.269 -1.353

YFL005W-YFL004W_376_455 376 80 -11.100 500 -7.891 2.160 -1.486

YFL005W-YFL004W_381_460 381 80 -8.900 500 -8.514 2.322 -0.166

YFL005W-YFL004W_386_465 386 80 -6.700 500 -8.720 2.366 0.854

YFL005W-YFL004W_391_470 391 80 -5.200 500 -7.892 2.188 1.230

YFL005W-YFL004W_396_475 396 80 -8.400 500 -9.591 2.285 0.521

YFL005W-YFL004W_401_480 401 80 -8.400 500 -10.740 2.394 0.977

YFL005W-YFL004W_406_485 406 80 -9.300 500 -11.339 2.521 0.809

YFL005W-YFL004W_411_490 411 80 -9.900 500 -13.311 2.463 1.385

YFL005W-YFL004W_416_495 416 80 -10.800 500 -13.904 2.711 1.145

YFL005W-YFL004W_421_500 421 80 -10.700 500 -12.155 2.511 0.579

YFL005W-YFL004W_426_505 426 80 -8.900 500 -10.571 2.407 0.694

YFL005W-YFL004W_431_510 431 80 -8.600 500 -10.396 2.357 0.762

YFL005W-YFL004W_436_515 436 80 -8.300 500 -9.169 2.420 0.359

YFL005W-YFL004W_441_520 441 80 -7.600 500 -8.881 2.410 0.532

YFL005W-YFL004W_446_525 446 80 -8.900 500 -10.214 2.445 0.537

YFL005W-YFL004W_451_530 451 80 -8.900 500 -11.743 2.367 1.201

YFL005W-YFL004W_456_535 456 80 -10.890 500 -12.772 2.565 0.734

YFL005W-YFL004W_461_540 461 80 -8.900 500 -12.927 2.656 1.516

YFL005W-YFL004W_466_545 466 80 -10.100 500 -12.830 2.727 1.001

YFL005W-YFL004W_471_550 471 80 -11.200 500 -14.035 2.719 1.043

YFL005W-YFL004W_476_555 476 80 -13.100 500 -13.120 2.466 0.008

YFL005W-YFL004W_481_560 481 80 -12.700 500 -13.920 2.674 0.456

YFL005W-YFL004W_486_565 486 80 -13.490 500 -12.880 2.634 -0.232

YFL005W-YFL004W_491_570 491 80 -13.400 500 -11.491 2.530 -0.754

YFL005W-YFL004W_496_575 496 80 -15.800 500 -11.856 2.504 -1.575

YFL005W-YFL004W_501_580 501 80 -15.800 500 -12.762 2.588 -1.174

YFL005W-YFL004W_506_585 506 80 -15.800 500 -11.709 2.330 -1.756

YFL005W-YFL004W_511_590 511 80 -19.300 500 -14.336 2.667 -1.861

YFL005W-YFL004W_516_595 516 80 -20.100 500 -15.505 2.720 -1.689

YFL005W-YFL004W_521_600 521 80 -21.300 500 -17.598 2.720 -1.361

YFL005W-YFL004W_526_605 526 80 -19.300 500 -15.656 2.799 -1.302

YFL005W-YFL004W_531_610 531 80 -15.600 500 -14.585 2.719 -0.373

YFL005W-YFL004W_536_615 536 80 -16.900 500 -16.278 2.920 -0.213

YFL005W-YFL004W_541_620 541 80 -21.200 500 -15.566 2.744 -2.053

YFL005W-YFL004W_546_625 546 80 -21.100 500 -15.179 2.886 -2.052

YFL005W-YFL004W_551_630 551 80 -18.800 500 -12.948 2.768 -2.114

YFL005W-YFL004W_556_635 556 80 -18.800 500 -13.165 2.852 -1.976

YFL005W-YFL004W_561_640 561 80 -20.100 500 -13.150 2.592 -2.681

YFL005W-YFL004W_566_645 566 80 -19.800 500 -13.065 2.777 -2.425

YFL005W-YFL004W_571_650 571 80 -13.600 500 -12.585 2.442 -0.416

YFL005W-YFL004W_576_655 576 80 -12.630 500 -11.985 2.503 -0.258

YFL005W-YFL004W_581_660 581 80 -9.030 500 -9.531 2.437 0.206

YFL005W-YFL004W_586_665 586 80 -11.100 500 -9.598 2.223 -0.676

YFL005W-YFL004W_591_670 591 80 -11.100 500 -9.115 2.302 -0.862

YFL005W-YFL004W_596_675 596 80 -6.030 500 -6.986 2.061 0.464

YFL005W-YFL004W_601_680 601 80 -6.030 500 -6.121 2.186 0.042

YFL005W-YFL004W_606_685 606 80 -8.030 500 -8.018 2.123 -0.006

YFL005W-YFL004W_611_690 611 80 -9.400 500 -10.031 2.443 0.258

YFL005W-YFL004W_616_695 616 80 -8.030 500 -8.432 2.228 0.181

YFL005W-YFL004W_621_700 621 80 -8.130 500 -8.665 2.349 0.228

YFL005W-YFL004W_626_705 626 80 -7.930 500 -8.605 2.115 0.319

YFL005W-YFL004W_631_710 631 80 -6.700 500 -5.687 1.853 -0.547

YFL005W-YFL004W_636_715 636 80 -6.700 500 -6.050 1.960 -0.332

YFL005W-YFL004W_641_720 641 80 -8.000 500 -9.807 2.377 0.760

YFL005W-YFL004W_646_725 646 80 -9.500 500 -10.852 2.594 0.521

YFL005W-YFL004W_651_730 651 80 -8.900 500 -11.431 2.461 1.028

YFL005W-YFL004W_656_735 656 80 -12.600 500 -14.109 2.691 0.561

YFL005W-YFL004W_661_740 661 80 -15.200 500 -15.747 2.757 0.199

YFL005W-YFL004W_666_745 666 80 -18.000 500 -16.147 2.902 -0.639

YFL005W-YFL004W_671_750 671 80 -16.400 500 -15.302 2.742 -0.401

YFL005W-YFL004W_676_755 676 80 -13.000 500 -13.923 2.620 0.352

YFL005W-YFL004W_681_760 681 80 -12.800 500 -14.520 2.666 0.645

YFL005W-YFL004W_686_765 686 80 -15.100 500 -14.782 2.739 -0.116

YFL005W-YFL004W_691_770 691 80 -17.800 500 -12.542 2.423 -2.170

YFL005W-YFL004W_696_775 696 80 -14.100 500 -11.251 2.524 -1.129

YFL005W-YFL004W_701_780 701 80 -12.200 500 -9.172 2.270 -1.334

YFL005W-YFL004W_706_785 706 80 -6.720 500 -6.766 2.074 0.022

YFL005W-YFL004W_711_790 711 80 -6.320 500 -4.814 1.929 -0.781

YFL005W-YFL004W_716_795 716 80 -3.920 500 -2.889 1.650 -0.625

YFL005W-YFL004W_721_800 721 80 -3.900 500 -3.731 1.752 -0.097

YFL005W-YFL004W_726_805 726 80 -6.100 500 -4.949 1.952 -0.590

YFL005W-YFL004W_731_810 731 80 -10.000 500 -4.378 1.995 -2.818

YFL005W-YFL004W_736_815 736 80 -5.800 500 -4.744 1.818 -0.580

YFL005W-YFL004W_741_820 741 80 -5.600 500 -4.691 1.952 -0.465

YFL005W-YFL004W_746_825 746 80 -4.500 500 -4.197 1.835 -0.165

YFL005W-YFL004W_750_828 750 78 -5.100 500 -5.734 1.902 0.333

Window size = 85

YFL005W-YFL004W_1_85 1 85 -13.700 500 -14.226 2.643 0.199

YFL005W-YFL004W_6_90 6 85 -17.200 500 -13.793 2.600 -1.310

YFL005W-YFL004W_11_95 11 85 -15.000 500 -10.688 2.514 -1.715

YFL005W-YFL004W_16_100 16 85 -17.600 500 -14.026 2.769 -1.291

YFL005W-YFL004W_21_105 21 85 -15.000 500 -12.773 2.588 -0.860

YFL005W-YFL004W_26_110 26 85 -12.650 500 -12.096 2.635 -0.210

YFL005W-YFL004W_31_115 31 85 -11.150 500 -10.410 2.415 -0.307

YFL005W-YFL004W_36_120 36 85 -10.600 500 -10.034 2.298 -0.246

YFL005W-YFL004W_41_125 41 85 -9.300 500 -8.689 2.444 -0.250

YFL005W-YFL004W_46_130 46 85 -5.400 500 -6.355 2.060 0.464

YFL005W-YFL004W_51_135 51 85 -4.500 500 -5.526 2.019 0.508

YFL005W-YFL004W_56_140 56 85 -3.950 500 -3.882 1.866 -0.036

YFL005W-YFL004W_61_145 61 85 -4.500 500 -4.517 1.732 0.010

YFL005W-YFL004W_66_150 66 85 -4.500 500 -3.534 1.724 -0.560

YFL005W-YFL004W_71_155 71 85 -4.500 500 -3.161 1.706 -0.785

YFL005W-YFL004W_76_160 76 85 -4.500 500 -3.714 1.738 -0.452

YFL005W-YFL004W_81_165 81 85 -4.500 500 -3.802 1.777 -0.393

YFL005W-YFL004W_86_170 86 85 -4.400 500 -4.836 2.021 0.216

YFL005W-YFL004W_91_175 91 85 -4.800 500 -5.240 2.056 0.214

YFL005W-YFL004W_96_180 96 85 -4.800 500 -6.642 2.238 0.823

YFL005W-YFL004W_101_185 101 85 -5.500 500 -5.799 2.054 0.146

YFL005W-YFL004W_106_190 106 85 -6.000 500 -5.825 1.986 -0.088

YFL005W-YFL004W_111_195 111 85 -6.000 500 -6.949 2.115 0.449

YFL005W-YFL004W_116_200 116 85 -6.000 500 -6.584 2.140 0.273

YFL005W-YFL004W_121_205 121 85 -6.390 500 -6.447 1.941 0.029

YFL005W-YFL004W_126_210 126 85 -6.000 500 -5.846 2.114 -0.073

YFL005W-YFL004W_131_215 131 85 -6.100 500 -4.738 2.026 -0.672

YFL005W-YFL004W_136_220 136 85 -6.100 500 -4.423 1.961 -0.855

YFL005W-YFL004W_141_225 141 85 -5.200 500 -4.523 1.931 -0.350

YFL005W-YFL004W_146_230 146 85 -3.900 500 -2.793 1.588 -0.698

YFL005W-YFL004W_151_235 151 85 -3.900 500 -2.680 1.674 -0.729

YFL005W-YFL004W_156_240 156 85 -3.800 500 -3.314 1.658 -0.293

YFL005W-YFL004W_161_245 161 85 -3.800 500 -3.063 1.680 -0.439

YFL005W-YFL004W_166_250 166 85 -5.700 500 -5.074 2.095 -0.299

YFL005W-YFL004W_171_255 171 85 -5.700 500 -4.036 1.892 -0.879

YFL005W-YFL004W_176_260 176 85 -5.400 500 -4.938 2.030 -0.228

YFL005W-YFL004W_181_265 181 85 -5.800 500 -5.677 2.107 -0.058

YFL005W-YFL004W_186_270 186 85 -7.100 500 -6.394 2.096 -0.337

YFL005W-YFL004W_191_275 191 85 -7.100 500 -6.541 2.001 -0.280

YFL005W-YFL004W_196_280 196 85 -7.100 500 -8.402 2.249 0.579

YFL005W-YFL004W_201_285 201 85 -7.100 500 -9.799 2.301 1.173

YFL005W-YFL004W_206_290 206 85 -8.600 500 -11.236 2.314 1.139

YFL005W-YFL004W_211_295 211 85 -9.600 500 -11.242 2.421 0.678

YFL005W-YFL004W_216_300 216 85 -9.900 500 -13.110 2.563 1.252

YFL005W-YFL004W_221_305 221 85 -13.400 500 -13.710 2.632 0.118

YFL005W-YFL004W_226_310 226 85 -13.300 500 -13.841 2.676 0.202

YFL005W-YFL004W_231_315 231 85 -9.600 500 -12.641 2.664 1.142

YFL005W-YFL004W_236_320 236 85 -11.100 500 -12.780 2.688 0.625

YFL005W-YFL004W_241_325 241 85 -15.600 500 -13.246 2.670 -0.882

YFL005W-YFL004W_246_330 246 85 -11.300 500 -12.542 2.595 0.479

YFL005W-YFL004W_251_335 251 85 -9.200 500 -12.412 2.501 1.285

YFL005W-YFL004W_256_340 256 85 -10.100 500 -13.206 2.470 1.258

YFL005W-YFL004W_261_345 261 85 -8.600 500 -11.650 2.580 1.182

YFL005W-YFL004W_266_350 266 85 -13.000 500 -12.667 2.867 -0.116

YFL005W-YFL004W_271_355 271 85 -15.200 500 -13.956 2.935 -0.424

YFL005W-YFL004W_276_360 276 85 -14.900 500 -14.283 2.777 -0.222

YFL005W-YFL004W_281_365 281 85 -16.800 500 -13.927 3.008 -0.955

YFL005W-YFL004W_286_370 286 85 -15.730 500 -11.443 2.900 -1.478

YFL005W-YFL004W_291_375 291 85 -16.900 500 -11.874 2.632 -1.910

YFL005W-YFL004W_296_380 296 85 -17.600 500 -12.965 2.543 -1.822

YFL005W-YFL004W_301_385 301 85 -17.600 500 -13.233 2.888 -1.512

YFL005W-YFL004W_306_390 306 85 -17.600 500 -12.946 2.823 -1.649

YFL005W-YFL004W_311_395 311 85 -23.300 500 -13.135 2.746 -3.702

YFL005W-YFL004W_316_400 316 85 -22.600 500 -12.902 2.694 -3.599

YFL005W-YFL004W_321_405 321 85 -19.500 500 -12.363 2.605 -2.740

YFL005W-YFL004W_326_410 326 85 -19.600 500 -10.161 2.262 -4.173 ***

YFL005W-YFL004W_331_415 331 85 -15.900 500 -10.528 2.386 -2.251

YFL005W-YFL004W_336_420 336 85 -14.200 500 -7.756 2.111 -3.052

YFL005W-YFL004W_341_425 341 85 -9.100 500 -9.557 2.390 0.191

YFL005W-YFL004W_346_430 346 85 -9.300 500 -10.529 2.441 0.504

YFL005W-YFL004W_351_435 351 85 -10.100 500 -8.340 2.320 -0.759

YFL005W-YFL004W_356_440 356 85 -8.900 500 -8.002 2.194 -0.409

YFL005W-YFL004W_361_445 361 85 -11.700 500 -8.683 2.375 -1.270

YFL005W-YFL004W_366_450 366 85 -11.100 500 -8.364 2.348 -1.165

YFL005W-YFL004W_371_455 371 85 -11.100 500 -7.786 2.165 -1.530

YFL005W-YFL004W_376_460 376 85 -11.100 500 -8.573 2.293 -1.102

YFL005W-YFL004W_381_465 381 85 -8.900 500 -10.331 2.554 0.560

YFL005W-YFL004W_386_470 386 85 -6.700 500 -9.385 2.378 1.129

YFL005W-YFL004W_391_475 391 85 -8.400 500 -10.007 2.448 0.656

YFL005W-YFL004W_396_480 396 85 -8.400 500 -10.931 2.557 0.990

YFL005W-YFL004W_401_485 401 85 -9.300 500 -11.647 2.433 0.965

YFL005W-YFL004W_406_490 406 85 -10.100 500 -13.579 2.585 1.346

YFL005W-YFL004W_411_495 411 85 -10.800 500 -13.859 2.547 1.201

YFL005W-YFL004W_416_500 416 85 -11.500 500 -14.037 2.613 0.971

YFL005W-YFL004W_421_505 421 85 -10.700 500 -12.235 2.618 0.586

YFL005W-YFL004W_426_510 426 85 -9.100 500 -11.670 2.530 1.016

YFL005W-YFL004W_431_515 431 85 -8.600 500 -10.248 2.415 0.683

YFL005W-YFL004W_436_520 436 85 -8.300 500 -9.484 2.242 0.528

YFL005W-YFL004W_441_525 441 85 -9.400 500 -10.316 2.408 0.380

YFL005W-YFL004W_446_530 446 85 -8.900 500 -12.282 2.388 1.416

YFL005W-YFL004W_451_535 451 85 -10.890 500 -13.124 2.663 0.839

YFL005W-YFL004W_456_540 456 85 -10.890 500 -13.308 2.790 0.867

YFL005W-YFL004W_461_545 461 85 -12.000 500 -15.405 2.759 1.234

YFL005W-YFL004W_466_550 466 85 -13.600 500 -14.947 2.762 0.488

YFL005W-YFL004W_471_555 471 85 -14.100 500 -15.085 2.790 0.353

YFL005W-YFL004W_476_560 476 85 -13.100 500 -14.820 2.708 0.635

YFL005W-YFL004W_481_565 481 85 -15.600 500 -15.122 2.898 -0.165

YFL005W-YFL004W_486_570 486 85 -13.490 500 -13.730 2.890 0.083

YFL005W-YFL004W_491_575 491 85 -15.800 500 -12.222 2.607 -1.373

YFL005W-YFL004W_496_580 496 85 -15.800 500 -13.516 2.577 -0.886

YFL005W-YFL004W_501_585 501 85 -15.860 500 -12.850 2.629 -1.145

YFL005W-YFL004W_506_590 506 85 -19.300 500 -13.953 2.646 -2.021

YFL005W-YFL004W_511_595 511 85 -20.100 500 -15.082 2.748 -1.826

YFL005W-YFL004W_516_600 516 85 -21.600 500 -18.632 2.982 -0.995

YFL005W-YFL004W_521_605 521 85 -21.400 500 -18.158 2.848 -1.138

YFL005W-YFL004W_526_610 526 85 -20.700 500 -17.278 3.059 -1.119

YFL005W-YFL004W_531_615 531 85 -19.200 500 -16.660 2.937 -0.865

YFL005W-YFL004W_536_620 536 85 -22.900 500 -16.519 2.814 -2.268

YFL005W-YFL004W_541_625 541 85 -21.200 500 -16.634 2.942 -1.552

YFL005W-YFL004W_546_630 546 85 -21.100 500 -15.900 2.589 -2.008

YFL005W-YFL004W_551_635 551 85 -18.800 500 -14.029 2.816 -1.694

YFL005W-YFL004W_556_640 556 85 -20.100 500 -14.140 2.897 -2.057

YFL005W-YFL004W_561_645 561 85 -20.100 500 -13.976 2.632 -2.327

YFL005W-YFL004W_566_650 566 85 -21.400 500 -14.093 2.829 -2.583

YFL005W-YFL004W_571_655 571 85 -13.600 500 -13.183 2.637 -0.158

YFL005W-YFL004W_576_660 576 85 -12.630 500 -12.123 2.528 -0.200

YFL005W-YFL004W_581_665 581 85 -11.100 500 -9.702 2.374 -0.589

YFL005W-YFL004W_586_670 586 85 -11.100 500 -9.684 2.395 -0.591

YFL005W-YFL004W_591_675 591 85 -11.100 500 -10.238 2.392 -0.360

YFL005W-YFL004W_596_680 596 85 -6.030 500 -7.665 2.199 0.744

YFL005W-YFL004W_601_685 601 85 -8.030 500 -9.119 2.486 0.438

YFL005W-YFL004W_606_690 606 85 -9.530 500 -10.833 2.582 0.505

YFL005W-YFL004W_611_695 611 85 -9.430 500 -9.913 2.408 0.200

YFL005W-YFL004W_616_700 616 85 -8.130 500 -8.873 2.345 0.317

YFL005W-YFL004W_621_705 621 85 -8.130 500 -8.239 2.218 0.049

YFL005W-YFL004W_626_710 626 85 -7.930 500 -8.523 2.287 0.259

YFL005W-YFL004W_631_715 631 85 -6.700 500 -5.774 1.962 -0.472

YFL005W-YFL004W_636_720 636 85 -8.000 500 -9.921 2.452 0.784

YFL005W-YFL004W_641_725 641 85 -9.500 500 -11.180 2.592 0.648

YFL005W-YFL004W_646_730 646 85 -9.700 500 -12.705 2.608 1.152

YFL005W-YFL004W_651_735 651 85 -13.600 500 -14.487 2.661 0.333

YFL005W-YFL004W_656_740 656 85 -15.200 500 -16.608 2.804 0.502

YFL005W-YFL004W_661_745 661 85 -18.000 500 -17.103 2.822 -0.318

YFL005W-YFL004W_666_750 666 85 -18.000 500 -16.175 2.835 -0.644

YFL005W-YFL004W_671_755 671 85 -16.400 500 -16.567 2.750 0.061

YFL005W-YFL004W_676_760 676 85 -13.000 500 -13.711 2.630 0.270

YFL005W-YFL004W_681_765 681 85 -15.100 500 -16.048 2.793 0.339

YFL005W-YFL004W_686_770 686 85 -18.700 500 -15.220 2.735 -1.272

YFL005W-YFL004W_691_775 691 85 -18.800 500 -13.881 2.793 -1.761

YFL005W-YFL004W_696_780 696 85 -14.100 500 -11.921 2.376 -0.917

YFL005W-YFL004W_701_785 701 85 -12.200 500 -8.826 2.457 -1.373

YFL005W-YFL004W_706_790 706 85 -7.120 500 -7.302 2.038 0.089

YFL005W-YFL004W_711_795 711 85 -6.320 500 -4.420 1.942 -0.978

YFL005W-YFL004W_716_800 716 85 -5.220 500 -4.580 1.882 -0.340

YFL005W-YFL004W_721_805 721 85 -6.100 500 -5.595 1.898 -0.266

YFL005W-YFL004W_726_810 726 85 -10.000 500 -6.292 2.123 -1.746

YFL005W-YFL004W_731_815 731 85 -10.000 500 -4.673 1.888 -2.821

YFL005W-YFL004W_736_820 736 85 -5.800 500 -5.048 2.026 -0.371

YFL005W-YFL004W_741_825 741 85 -5.600 500 -6.271 2.127 0.316

YFL005W-YFL004W_745_828 745 83 -5.800 500 -4.986 1.870 -0.435

Window size = 90

YFL005W-YFL004W_1_90 1 90 -17.900 500 -15.460 2.766 -0.882

YFL005W-YFL004W_6_95 6 90 -17.200 500 -14.021 2.592 -1.226

YFL005W-YFL004W_11_100 11 90 -18.700 500 -14.125 2.721 -1.681

YFL005W-YFL004W_16_105 16 90 -17.600 500 -14.057 2.633 -1.346

YFL005W-YFL004W_21_110 21 90 -16.100 500 -13.201 2.635 -1.100

YFL005W-YFL004W_26_115 26 90 -12.650 500 -12.073 2.677 -0.215

YFL005W-YFL004W_31_120 31 90 -11.150 500 -10.823 2.549 -0.128

YFL005W-YFL004W_36_125 36 90 -10.600 500 -10.107 2.534 -0.195

YFL005W-YFL004W_41_130 41 90 -9.300 500 -9.211 2.304 -0.039

YFL005W-YFL004W_46_135 46 90 -7.050 500 -7.283 2.253 0.104

YFL005W-YFL004W_51_140 51 90 -4.500 500 -4.991 1.833 0.268

YFL005W-YFL004W_56_145 56 90 -4.900 500 -5.269 1.958 0.188

YFL005W-YFL004W_61_150 61 90 -4.500 500 -4.826 1.865 0.175

YFL005W-YFL004W_66_155 66 90 -4.500 500 -3.714 1.760 -0.447

YFL005W-YFL004W_71_160 71 90 -4.500 500 -3.376 1.710 -0.657

YFL005W-YFL004W_76_165 76 90 -4.500 500 -3.508 1.734 -0.572

YFL005W-YFL004W_81_170 81 90 -4.500 500 -4.220 1.750 -0.160

YFL005W-YFL004W_86_175 86 90 -5.200 500 -5.790 1.965 0.300

YFL005W-YFL004W_91_180 91 90 -4.800 500 -6.158 2.149 0.632

YFL005W-YFL004W_96_185 96 90 -6.700 500 -8.023 2.416 0.548

YFL005W-YFL004W_101_190 101 90 -6.000 500 -5.545 1.949 -0.234

YFL005W-YFL004W_106_195 106 90 -6.000 500 -6.348 2.163 0.161

YFL005W-YFL004W_111_200 111 90 -6.000 500 -7.187 2.185 0.543

YFL005W-YFL004W_116_205 116 90 -7.400 500 -7.527 2.219 0.057

YFL005W-YFL004W_121_210 121 90 -7.590 500 -6.678 2.042 -0.447

YFL005W-YFL004W_126_215 126 90 -8.800 500 -6.139 2.153 -1.236

YFL005W-YFL004W_131_220 131 90 -6.100 500 -4.866 1.930 -0.639

YFL005W-YFL004W_136_225 136 90 -6.100 500 -4.357 1.829 -0.953

YFL005W-YFL004W_141_230 141 90 -5.200 500 -4.705 2.019 -0.245

YFL005W-YFL004W_146_235 146 90 -3.900 500 -2.501 1.522 -0.920

YFL005W-YFL004W_151_240 151 90 -3.900 500 -3.225 1.737 -0.389

YFL005W-YFL004W_156_245 156 90 -3.800 500 -3.376 1.813 -0.234

YFL005W-YFL004W_161_250 161 90 -5.700 500 -5.423 1.995 -0.139

YFL005W-YFL004W_166_255 166 90 -5.700 500 -5.501 2.004 -0.100

YFL005W-YFL004W_171_260 171 90 -5.700 500 -4.921 1.956 -0.398

YFL005W-YFL004W_176_265 176 90 -9.300 500 -7.565 2.231 -0.777

YFL005W-YFL004W_181_270 181 90 -7.100 500 -7.667 2.083 0.272

YFL005W-YFL004W_186_275 186 90 -7.100 500 -6.352 2.041 -0.366

YFL005W-YFL004W_191_280 191 90 -7.100 500 -8.449 2.209 0.611

YFL005W-YFL004W_196_285 196 90 -7.100 500 -9.668 2.337 1.099

YFL005W-YFL004W_201_290 201 90 -8.600 500 -11.756 2.316 1.363

YFL005W-YFL004W_206_295 206 90 -9.600 500 -11.129 2.309 0.662

YFL005W-YFL004W_211_300 211 90 -9.900 500 -13.260 2.574 1.305

YFL005W-YFL004W_216_305 216 90 -13.400 500 -14.287 2.690 0.330

YFL005W-YFL004W_221_310 221 90 -13.400 500 -14.393 2.711 0.366

YFL005W-YFL004W_226_315 226 90 -13.300 500 -13.433 2.442 0.054

YFL005W-YFL004W_231_320 231 90 -13.800 500 -14.347 2.564 0.213

YFL005W-YFL004W_236_325 236 90 -15.600 500 -13.942 2.603 -0.637

YFL005W-YFL004W_241_330 241 90 -15.600 500 -13.776 2.625 -0.695

YFL005W-YFL004W_246_335 246 90 -12.800 500 -14.082 2.534 0.506

YFL005W-YFL004W_251_340 251 90 -10.900 500 -15.071 2.971 1.404

YFL005W-YFL004W_256_345 256 90 -10.560 500 -14.466 2.697 1.448

YFL005W-YFL004W_261_350 261 90 -13.600 500 -14.763 2.627 0.443

YFL005W-YFL004W_266_355 266 90 -17.000 500 -14.619 2.719 -0.876

YFL005W-YFL004W_271_360 271 90 -16.500 500 -15.044 2.721 -0.535

YFL005W-YFL004W_276_365 276 90 -17.600 500 -15.314 2.847 -0.803

YFL005W-YFL004W_281_370 281 90 -17.300 500 -13.856 2.635 -1.307

YFL005W-YFL004W_286_375 286 90 -16.900 500 -11.716 2.511 -2.064

YFL005W-YFL004W_291_380 291 90 -17.600 500 -12.909 2.718 -1.726

YFL005W-YFL004W_296_385 296 90 -17.600 500 -13.857 2.803 -1.336

YFL005W-YFL004W_301_390 301 90 -22.300 500 -13.837 2.740 -3.089

YFL005W-YFL004W_306_395 306 90 -23.300 500 -13.537 2.611 -3.739

YFL005W-YFL004W_311_400 311 90 -23.400 500 -13.265 2.574 -3.938

YFL005W-YFL004W_316_405 316 90 -22.600 500 -13.408 2.677 -3.434

YFL005W-YFL004W_321_410 321 90 -20.000 500 -12.836 2.576 -2.781

YFL005W-YFL004W_326_415 326 90 -19.600 500 -11.254 2.573 -3.244

YFL005W-YFL004W_331_420 331 90 -16.700 500 -10.626 2.232 -2.721

YFL005W-YFL004W_336_425 336 90 -14.200 500 -10.587 2.502 -1.444

YFL005W-YFL004W_341_430 341 90 -9.300 500 -10.660 2.338 0.582

YFL005W-YFL004W_346_435 346 90 -10.100 500 -11.206 2.649 0.418

YFL005W-YFL004W_351_440 351 90 -11.300 500 -8.804 2.212 -1.128

YFL005W-YFL004W_356_445 356 90 -11.700 500 -9.237 2.426 -1.015

YFL005W-YFL004W_361_450 361 90 -11.700 500 -9.444 2.389 -0.945

YFL005W-YFL004W_366_455 366 90 -11.100 500 -8.382 2.336 -1.164

YFL005W-YFL004W_371_460 371 90 -11.100 500 -8.256 2.316 -1.228

YFL005W-YFL004W_376_465 376 90 -11.100 500 -10.311 2.518 -0.313

YFL005W-YFL004W_381_470 381 90 -8.900 500 -10.928 2.371 0.855

YFL005W-YFL004W_386_475 386 90 -10.900 500 -11.313 2.489 0.166

YFL005W-YFL004W_391_480 391 90 -8.400 500 -11.099 2.511 1.075

YFL005W-YFL004W_396_485 396 90 -9.300 500 -11.633 2.524 0.924

YFL005W-YFL004W_401_490 401 90 -10.100 500 -14.308 2.684 1.568

YFL005W-YFL004W_406_495 406 90 -12.500 500 -14.607 2.686 0.784

YFL005W-YFL004W_411_500 411 90 -11.500 500 -14.300 2.565 1.091

YFL005W-YFL004W_416_505 416 90 -11.500 500 -13.912 2.566 0.940

YFL005W-YFL004W_421_510 421 90 -10.700 500 -13.406 2.581 1.048

YFL005W-YFL004W_426_515 426 90 -9.300 500 -11.757 2.678 0.918

YFL005W-YFL004W_431_520 431 90 -8.600 500 -10.603 2.395 0.836

YFL005W-YFL004W_436_525 436 90 -10.800 500 -11.053 2.368 0.107

YFL005W-YFL004W_441_530 441 90 -9.400 500 -12.410 2.607 1.155

YFL005W-YFL004W_446_535 446 90 -10.890 500 -13.498 2.712 0.962

YFL005W-YFL004W_451_540 451 90 -12.090 500 -13.905 2.519 0.721

YFL005W-YFL004W_456_545 456 90 -12.200 500 -15.105 2.599 1.118

YFL005W-YFL004W_461_550 461 90 -14.500 500 -17.383 2.784 1.036

YFL005W-YFL004W_466_555 466 90 -16.500 500 -16.153 2.731 -0.127

YFL005W-YFL004W_471_560 471 90 -14.100 500 -16.821 2.675 1.017

YFL005W-YFL004W_476_565 476 90 -16.100 500 -15.711 2.471 -0.157

YFL005W-YFL004W_481_570 481 90 -15.600 500 -15.936 2.871 0.117

YFL005W-YFL004W_486_575 486 90 -15.800 500 -14.533 2.822 -0.449

YFL005W-YFL004W_491_580 491 90 -15.800 500 -13.806 2.668 -0.747

YFL005W-YFL004W_496_585 496 90 -15.860 500 -13.723 2.717 -0.786

YFL005W-YFL004W_501_590 501 90 -19.300 500 -14.861 2.751 -1.613

YFL005W-YFL004W_506_595 506 90 -20.100 500 -14.758 2.729 -1.957

YFL005W-YFL004W_511_600 511 90 -22.000 500 -18.207 2.934 -1.293

YFL005W-YFL004W_516_605 516 90 -21.700 500 -18.926 2.893 -0.959

YFL005W-YFL004W_521_610 521 90 -23.000 500 -19.470 2.877 -1.227

YFL005W-YFL004W_526_615 526 90 -24.800 500 -19.131 3.041 -1.864

YFL005W-YFL004W_531_620 531 90 -25.200 500 -17.040 3.023 -2.699

YFL005W-YFL004W_536_625 536 90 -22.900 500 -17.531 2.997 -1.792

YFL005W-YFL004W_541_630 541 90 -21.200 500 -17.647 2.912 -1.220

YFL005W-YFL004W_546_635 546 90 -21.100 500 -17.121 2.779 -1.432

YFL005W-YFL004W_551_640 551 90 -20.100 500 -15.065 2.838 -1.774

YFL005W-YFL004W_556_645 556 90 -20.100 500 -15.144 2.853 -1.737

YFL005W-YFL004W_561_650 561 90 -21.700 500 -15.560 2.800 -2.193

YFL005W-YFL004W_566_655 566 90 -21.400 500 -14.394 2.678 -2.616

YFL005W-YFL004W_571_660 571 90 -13.600 500 -13.134 2.662 -0.175

YFL005W-YFL004W_576_665 576 90 -14.600 500 -12.039 2.654 -0.965

YFL005W-YFL004W_581_670 581 90 -11.100 500 -9.781 2.385 -0.553

YFL005W-YFL004W_586_675 586 90 -11.100 500 -10.751 2.471 -0.141

YFL005W-YFL004W_591_680 591 90 -11.100 500 -10.832 2.511 -0.107

YFL005W-YFL004W_596_685 596 90 -8.030 500 -10.775 2.530 1.085

YFL005W-YFL004W_601_690 601 90 -10.930 500 -12.081 2.655 0.433

YFL005W-YFL004W_606_695 606 90 -9.530 500 -10.969 2.361 0.609

YFL005W-YFL004W_611_700 611 90 -9.530 500 -10.318 2.286 0.345

YFL005W-YFL004W_616_705 616 90 -8.130 500 -8.595 2.189 0.213

YFL005W-YFL004W_621_710 621 90 -8.130 500 -8.503 2.189 0.170

YFL005W-YFL004W_626_715 626 90 -7.930 500 -8.545 2.197 0.280

YFL005W-YFL004W_631_720 631 90 -8.000 500 -9.632 2.297 0.710

YFL005W-YFL004W_636_725 636 90 -9.500 500 -11.023 2.463 0.618

YFL005W-YFL004W_641_730 641 90 -9.700 500 -12.981 2.547 1.288

YFL005W-YFL004W_646_735 646 90 -15.800 500 -15.557 2.677 -0.091

YFL005W-YFL004W_651_740 651 90 -17.900 500 -16.663 2.782 -0.445

YFL005W-YFL004W_656_745 656 90 -18.000 500 -17.988 2.706 -0.004

YFL005W-YFL004W_661_750 661 90 -18.000 500 -17.165 2.824 -0.296

YFL005W-YFL004W_666_755 666 90 -18.000 500 -16.910 2.778 -0.393

YFL005W-YFL004W_671_760 671 90 -16.400 500 -16.047 2.917 -0.121

YFL005W-YFL004W_676_765 676 90 -15.100 500 -15.093 2.678 -0.003

YFL005W-YFL004W_681_770 681 90 -18.700 500 -16.215 2.607 -0.953

YFL005W-YFL004W_686_775 686 90 -25.000 500 -16.240 2.767 -3.166

YFL005W-YFL004W_691_780 691 90 -18.800 500 -14.925 2.547 -1.521

YFL005W-YFL004W_696_785 696 90 -14.100 500 -11.381 2.464 -1.103

YFL005W-YFL004W_701_790 701 90 -12.200 500 -9.549 2.380 -1.114

YFL005W-YFL004W_706_795 706 90 -8.620 500 -6.945 2.013 -0.832

YFL005W-YFL004W_711_800 711 90 -6.320 500 -6.214 1.987 -0.053

YFL005W-YFL004W_716_805 716 90 -6.100 500 -6.507 2.110 0.193

YFL005W-YFL004W_721_810 721 90 -10.000 500 -6.865 2.022 -1.550

YFL005W-YFL004W_726_815 726 90 -10.000 500 -6.528 2.106 -1.648

YFL005W-YFL004W_731_820 731 90 -10.000 500 -5.431 1.944 -2.350

YFL005W-YFL004W_736_825 736 90 -5.800 500 -6.640 2.113 0.397

YFL005W-YFL004W_740_828 740 88 -5.800 500 -7.185 2.256 0.614

Window size = 95

YFL005W-YFL004W_1_95 1 95 -18.400 500 -15.800 2.718 -0.957

YFL005W-YFL004W_6_100 6 95 -18.700 500 -17.169 2.757 -0.555

YFL005W-YFL004W_11_105 11 95 -18.700 500 -14.086 2.805 -1.645

YFL005W-YFL004W_16_110 16 95 -17.600 500 -14.296 2.740 -1.206

YFL005W-YFL004W_21_115 21 95 -16.200 500 -13.173 2.559 -1.183

YFL005W-YFL004W_26_120 26 95 -12.650 500 -12.350 2.794 -0.107

YFL005W-YFL004W_31_125 31 95 -11.150 500 -10.774 2.528 -0.149

YFL005W-YFL004W_36_130 36 95 -10.600 500 -10.667 2.522 0.027

YFL005W-YFL004W_41_135 41 95 -10.600 500 -10.160 2.456 -0.179

YFL005W-YFL004W_46_140 46 95 -7.050 500 -6.764 2.095 -0.137

YFL005W-YFL004W_51_145 51 95 -5.400 500 -6.498 2.098 0.523

YFL005W-YFL004W_56_150 56 95 -5.600 500 -5.406 1.920 -0.101

YFL005W-YFL004W_61_155 61 95 -4.500 500 -4.803 1.953 0.155

YFL005W-YFL004W_66_160 66 95 -4.500 500 -4.050 1.853 -0.243

YFL005W-YFL004W_71_165 71 95 -4.500 500 -3.242 1.641 -0.767

YFL005W-YFL004W_76_170 76 95 -4.500 500 -4.166 1.845 -0.181

YFL005W-YFL004W_81_175 81 95 -5.200 500 -5.156 1.899 -0.023

YFL005W-YFL004W_86_180 86 95 -5.300 500 -6.705 2.146 0.655

YFL005W-YFL004W_91_185 91 95 -6.700 500 -7.721 2.271 0.450

YFL005W-YFL004W_96_190 96 95 -7.200 500 -7.952 2.345 0.321

YFL005W-YFL004W_101_195 101 95 -6.000 500 -6.151 2.003 0.076

YFL005W-YFL004W_106_200 106 95 -6.000 500 -6.534 2.179 0.245

YFL005W-YFL004W_111_205 111 95 -7.400 500 -7.958 2.115 0.264

YFL005W-YFL004W_116_210 116 95 -7.590 500 -7.680 2.091 0.043

YFL005W-YFL004W_121_215 121 95 -8.800 500 -6.962 1.927 -0.954

YFL005W-YFL004W_126_220 126 95 -8.800 500 -6.416 1.950 -1.222

YFL005W-YFL004W_131_225 131 95 -6.100 500 -4.562 1.965 -0.783

YFL005W-YFL004W_136_230 136 95 -6.100 500 -4.331 1.962 -0.901

YFL005W-YFL004W_141_235 141 95 -5.200 500 -4.090 1.995 -0.556

YFL005W-YFL004W_146_240 146 95 -3.900 500 -3.149 1.660 -0.453

YFL005W-YFL004W_151_245 151 95 -3.900 500 -3.403 1.768 -0.281

YFL005W-YFL004W_156_250 156 95 -5.700 500 -5.521 2.093 -0.086

YFL005W-YFL004W_161_255 161 95 -5.700 500 -5.804 2.016 0.051

YFL005W-YFL004W_166_260 166 95 -5.700 500 -6.541 1.998 0.421

YFL005W-YFL004W_171_265 171 95 -9.600 500 -7.699 2.165 -0.878

YFL005W-YFL004W_176_270 176 95 -10.600 500 -9.496 2.413 -0.458

YFL005W-YFL004W_181_275 181 95 -7.100 500 -7.535 2.012 0.216

YFL005W-YFL004W_186_280 186 95 -7.100 500 -8.694 2.247 0.709

YFL005W-YFL004W_191_285 191 95 -7.100 500 -9.544 2.357 1.037

YFL005W-YFL004W_196_290 196 95 -8.600 500 -11.780 2.315 1.374

YFL005W-YFL004W_201_295 201 95 -9.600 500 -11.114 2.447 0.619

YFL005W-YFL004W_206_300 206 95 -9.900 500 -13.367 2.435 1.424

YFL005W-YFL004W_211_305 211 95 -13.400 500 -14.857 2.829 0.515

YFL005W-YFL004W_216_310 216 95 -13.400 500 -14.669 2.565 0.495

YFL005W-YFL004W_221_315 221 95 -13.400 500 -13.737 2.638 0.128

YFL005W-YFL004W_226_320 226 95 -13.800 500 -15.110 2.615 0.501

YFL005W-YFL004W_231_325 231 95 -15.600 500 -15.654 2.794 0.019

YFL005W-YFL004W_236_330 236 95 -15.600 500 -14.824 2.557 -0.303

YFL005W-YFL004W_241_335 241 95 -15.600 500 -15.547 2.650 -0.020

YFL005W-YFL004W_246_340 246 95 -13.100 500 -16.417 2.865 1.158

YFL005W-YFL004W_251_345 251 95 -10.900 500 -16.561 2.755 2.055

YFL005W-YFL004W_256_350 256 95 -17.400 500 -17.373 3.160 -0.009

YFL005W-YFL004W_261_355 261 95 -17.000 500 -16.770 2.781 -0.083

YFL005W-YFL004W_266_360 266 95 -18.000 500 -16.113 2.816 -0.670

YFL005W-YFL004W_271_365 271 95 -17.600 500 -16.146 2.718 -0.535

YFL005W-YFL004W_276_370 276 95 -17.600 500 -15.423 2.655 -0.820

YFL005W-YFL004W_281_375 281 95 -18.500 500 -14.656 2.712 -1.418

YFL005W-YFL004W_286_380 286 95 -18.800 500 -13.295 2.817 -1.954

YFL005W-YFL004W_291_385 291 95 -17.600 500 -13.809 2.605 -1.455

YFL005W-YFL004W_296_390 296 95 -22.300 500 -14.673 2.797 -2.727

YFL005W-YFL004W_301_395 301 95 -23.300 500 -14.804 2.905 -2.925

YFL005W-YFL004W_306_400 306 95 -23.400 500 -14.044 2.652 -3.527

YFL005W-YFL004W_311_405 311 95 -23.400 500 -13.909 2.616 -3.628

YFL005W-YFL004W_316_410 316 95 -22.600 500 -13.877 2.770 -3.149

YFL005W-YFL004W_321_415 321 95 -21.400 500 -13.624 2.616 -2.972

YFL005W-YFL004W_326_420 326 95 -19.600 500 -11.549 2.479 -3.248

YFL005W-YFL004W_331_425 331 95 -16.700 500 -13.275 2.579 -1.328

YFL005W-YFL004W_336_430 336 95 -16.000 500 -11.600 2.628 -1.674

YFL005W-YFL004W_341_435 341 95 -10.500 500 -11.422 2.591 0.356

YFL005W-YFL004W_346_440 346 95 -11.300 500 -11.817 2.600 0.199

YFL005W-YFL004W_351_445 351 95 -11.700 500 -10.220 2.451 -0.604

YFL005W-YFL004W_356_450 356 95 -11.700 500 -9.701 2.415 -0.828

YFL005W-YFL004W_361_455 361 95 -11.700 500 -9.319 2.303 -1.034

YFL005W-YFL004W_366_460 366 95 -11.100 500 -8.883 2.267 -0.978

YFL005W-YFL004W_371_465 371 95 -11.100 500 -10.024 2.388 -0.451

YFL005W-YFL004W_376_470 376 95 -11.100 500 -10.988 2.439 -0.046

YFL005W-YFL004W_381_475 381 95 -13.100 500 -12.790 2.454 -0.126

YFL005W-YFL004W_386_480 386 95 -10.900 500 -12.568 2.647 0.630

YFL005W-YFL004W_391_485 391 95 -9.300 500 -11.932 2.513 1.047

YFL005W-YFL004W_396_490 396 95 -10.100 500 -14.368 2.583 1.652

YFL005W-YFL004W_401_495 401 95 -12.500 500 -14.794 2.668 0.860

YFL005W-YFL004W_406_500 406 95 -12.500 500 -14.873 2.786 0.852

YFL005W-YFL004W_411_505 411 95 -11.500 500 -14.367 2.810 1.020

YFL005W-YFL004W_416_510 416 95 -11.500 500 -15.128 2.724 1.332

YFL005W-YFL004W_421_515 421 95 -10.700 500 -13.078 2.582 0.921

YFL005W-YFL004W_426_520 426 95 -11.000 500 -12.013 2.640 0.384

YFL005W-YFL004W_431_525 431 95 -10.800 500 -12.291 2.627 0.567

YFL005W-YFL004W_436_530 436 95 -10.800 500 -12.822 2.522 0.802

YFL005W-YFL004W_441_535 441 95 -11.500 500 -14.010 2.695 0.931

YFL005W-YFL004W_446_540 446 95 -12.890 500 -14.198 2.647 0.494

YFL005W-YFL004W_451_545 451 95 -12.200 500 -15.880 2.749 1.339

YFL005W-YFL004W_456_550 456 95 -14.500 500 -17.536 2.780 1.092

YFL005W-YFL004W_461_555 461 95 -17.300 500 -18.817 2.893 0.524

YFL005W-YFL004W_466_560 466 95 -16.500 500 -17.848 2.882 0.468

YFL005W-YFL004W_471_565 471 95 -16.100 500 -17.938 2.818 0.652

YFL005W-YFL004W_476_570 476 95 -17.300 500 -17.082 3.015 -0.072

YFL005W-YFL004W_481_575 481 95 -17.200 500 -16.798 2.955 -0.136

YFL005W-YFL004W_486_580 486 95 -17.300 500 -16.154 2.661 -0.431

YFL005W-YFL004W_491_585 491 95 -15.860 500 -13.975 2.790 -0.676

YFL005W-YFL004W_496_590 496 95 -19.300 500 -15.754 2.762 -1.284

YFL005W-YFL004W_501_595 501 95 -20.100 500 -15.946 2.712 -1.532

YFL005W-YFL004W_506_600 506 95 -22.000 500 -17.732 2.771 -1.541

YFL005W-YFL004W_511_605 511 95 -22.000 500 -18.891 2.843 -1.094

YFL005W-YFL004W_516_610 516 95 -23.000 500 -20.383 2.904 -0.901

YFL005W-YFL004W_521_615 521 95 -25.300 500 -21.189 2.946 -1.395

YFL005W-YFL004W_526_620 526 95 -30.500 500 -19.594 3.161 -3.450

YFL005W-YFL004W_531_625 531 95 -25.200 500 -17.796 2.824 -2.622

YFL005W-YFL004W_536_630 536 95 -22.900 500 -18.000 2.885 -1.698

YFL005W-YFL004W_541_635 541 95 -21.200 500 -18.688 2.870 -0.875

YFL005W-YFL004W_546_640 546 95 -22.400 500 -17.915 2.885 -1.555

YFL005W-YFL004W_551_645 551 95 -20.100 500 -16.097 2.847 -1.406

YFL005W-YFL004W_556_650 556 95 -21.700 500 -16.167 2.903 -1.906

YFL005W-YFL004W_561_655 561 95 -21.700 500 -15.759 2.742 -2.167

YFL005W-YFL004W_566_660 566 95 -21.400 500 -14.560 2.760 -2.478

YFL005W-YFL004W_571_665 571 95 -14.600 500 -13.210 2.644 -0.526

YFL005W-YFL004W_576_670 576 95 -14.600 500 -12.216 2.615 -0.912

YFL005W-YFL004W_581_675 581 95 -11.100 500 -10.845 2.412 -0.106

YFL005W-YFL004W_586_680 586 95 -11.100 500 -11.364 2.432 0.109

YFL005W-YFL004W_591_685 591 95 -12.900 500 -13.972 2.825 0.379

YFL005W-YFL004W_596_690 596 95 -10.930 500 -13.605 2.586 1.034

YFL005W-YFL004W_601_695 601 95 -11.030 500 -12.191 2.553 0.455

YFL005W-YFL004W_606_700 606 95 -9.530 500 -11.082 2.322 0.668

YFL005W-YFL004W_611_705 611 95 -9.530 500 -10.491 2.271 0.423

YFL005W-YFL004W_616_710 616 95 -8.130 500 -8.508 2.174 0.174

YFL005W-YFL004W_621_715 621 95 -8.700 500 -8.936 2.320 0.102

YFL005W-YFL004W_626_720 626 95 -9.100 500 -12.649 2.608 1.361

YFL005W-YFL004W_631_725 631 95 -9.500 500 -10.926 2.445 0.583

YFL005W-YFL004W_636_730 636 95 -9.700 500 -12.945 2.561 1.267

YFL005W-YFL004W_641_735 641 95 -15.800 500 -16.219 2.676 0.156

YFL005W-YFL004W_646_740 646 95 -19.200 500 -18.248 3.053 -0.312

YFL005W-YFL004W_651_745 651 95 -18.000 500 -18.616 2.828 0.218

YFL005W-YFL004W_656_750 656 95 -18.000 500 -18.218 3.087 0.070

YFL005W-YFL004W_661_755 661 95 -18.000 500 -18.383 2.913 0.132

YFL005W-YFL004W_666_760 666 95 -18.000 500 -16.795 2.749 -0.438

YFL005W-YFL004W_671_765 671 95 -16.600 500 -17.624 2.913 0.352

YFL005W-YFL004W_676_770 676 95 -18.700 500 -15.425 2.854 -1.148

YFL005W-YFL004W_681_775 681 95 -25.300 500 -17.499 2.706 -2.883

YFL005W-YFL004W_686_780 686 95 -25.000 500 -17.386 2.847 -2.674

YFL005W-YFL004W_691_785 691 95 -18.800 500 -14.591 2.720 -1.547

YFL005W-YFL004W_696_790 696 95 -14.100 500 -12.566 2.691 -0.570

YFL005W-YFL004W_701_795 701 95 -12.200 500 -9.148 2.200 -1.387

YFL005W-YFL004W_706_800 706 95 -8.620 500 -8.961 2.353 0.145

YFL005W-YFL004W_711_805 711 95 -8.800 500 -8.385 2.303 -0.180

YFL005W-YFL004W_716_810 716 95 -10.000 500 -7.731 2.242 -1.012

YFL005W-YFL004W_721_815 721 95 -10.000 500 -7.415 2.218 -1.165

YFL005W-YFL004W_726_820 726 95 -10.000 500 -7.281 2.220 -1.225

YFL005W-YFL004W_731_825 731 95 -10.000 500 -6.793 2.095 -1.531

YFL005W-YFL004W_735_828 735 93 -6.000 500 -7.398 2.174 0.643

Window size = 100

YFL005W-YFL004W_1_100 1 100 -18.700 500 -18.778 3.122 0.025

YFL005W-YFL004W_6_105 6 100 -18.700 500 -17.361 2.836 -0.472

YFL005W-YFL004W_11_110 11 100 -18.700 500 -14.267 2.793 -1.587

YFL005W-YFL004W_16_115 16 100 -17.600 500 -14.172 2.508 -1.367

YFL005W-YFL004W_21_120 21 100 -16.200 500 -13.639 2.578 -0.994

YFL005W-YFL004W_26_125 26 100 -12.650 500 -12.131 2.673 -0.194

YFL005W-YFL004W_31_130 31 100 -11.150 500 -11.428 2.677 0.104

YFL005W-YFL004W_36_135 36 100 -11.900 500 -11.522 2.419 -0.156

YFL005W-YFL004W_41_140 41 100 -10.600 500 -9.823 2.229 -0.349

YFL005W-YFL004W_46_145 46 100 -7.600 500 -8.277 2.331 0.291

YFL005W-YFL004W_51_150 51 100 -5.600 500 -6.500 2.045 0.440

YFL005W-YFL004W_56_155 56 100 -5.600 500 -5.428 2.042 -0.084

YFL005W-YFL004W_61_160 61 100 -4.500 500 -5.059 2.069 0.270

YFL005W-YFL004W_66_165 66 100 -4.500 500 -3.786 1.797 -0.397

YFL005W-YFL004W_71_170 71 100 -4.500 500 -3.926 1.840 -0.312

YFL005W-YFL004W_76_175 76 100 -5.200 500 -5.070 1.948 -0.066

YFL005W-YFL004W_81_180 81 100 -5.490 500 -6.242 2.099 0.358

YFL005W-YFL004W_86_185 86 100 -7.800 500 -8.164 2.159 0.169

YFL005W-YFL004W_91_190 91 100 -7.200 500 -7.755 2.177 0.255

YFL005W-YFL004W_96_195 96 100 -7.200 500 -8.561 2.205 0.617

YFL005W-YFL004W_101_200 101 100 -6.000 500 -6.400 2.180 0.183

YFL005W-YFL004W_106_205 106 100 -7.400 500 -7.366 2.075 -0.016

YFL005W-YFL004W_111_210 111 100 -7.590 500 -8.132 2.150 0.252

YFL005W-YFL004W_116_215 116 100 -8.800 500 -7.829 2.216 -0.438

YFL005W-YFL004W_121_220 121 100 -8.800 500 -6.916 2.141 -0.880

YFL005W-YFL004W_126_225 126 100 -8.800 500 -6.523 2.132 -1.068

YFL005W-YFL004W_131_230 131 100 -6.100 500 -4.389 1.883 -0.908

YFL005W-YFL004W_136_235 136 100 -6.100 500 -4.006 1.832 -1.143

YFL005W-YFL004W_141_240 141 100 -5.200 500 -4.813 1.924 -0.201

YFL005W-YFL004W_146_245 146 100 -3.900 500 -3.130 1.613 -0.478

YFL005W-YFL004W_151_250 151 100 -5.800 500 -5.574 1.935 -0.117

YFL005W-YFL004W_156_255 156 100 -5.700 500 -5.943 2.165 0.112

YFL005W-YFL004W_161_260 161 100 -5.700 500 -6.774 2.269 0.473

YFL005W-YFL004W_166_265 166 100 -9.600 500 -9.077 2.341 -0.223

YFL005W-YFL004W_171_270 171 100 -10.900 500 -9.590 2.283 -0.574

YFL005W-YFL004W_176_275 176 100 -10.600 500 -9.449 2.271 -0.507

YFL005W-YFL004W_181_280 181 100 -7.100 500 -9.664 2.216 1.157

YFL005W-YFL004W_186_285 186 100 -7.100 500 -9.683 2.224 1.161

YFL005W-YFL004W_191_290 191 100 -8.700 500 -11.724 2.382 1.269

YFL005W-YFL004W_196_295 196 100 -9.600 500 -11.120 2.326 0.653

YFL005W-YFL004W_201_300 201 100 -10.000 500 -13.428 2.490 1.377

YFL005W-YFL004W_206_305 206 100 -13.400 500 -14.723 2.587 0.512

YFL005W-YFL004W_211_310 211 100 -13.400 500 -15.173 2.700 0.657

YFL005W-YFL004W_216_315 216 100 -13.400 500 -14.116 2.793 0.256

YFL005W-YFL004W_221_320 221 100 -13.800 500 -15.830 2.743 0.740

YFL005W-YFL004W_226_325 226 100 -15.600 500 -16.221 2.937 0.211

YFL005W-YFL004W_231_330 231 100 -15.600 500 -16.238 2.909 0.219

YFL005W-YFL004W_236_335 236 100 -17.600 500 -16.538 2.854 -0.372

YFL005W-YFL004W_241_340 241 100 -15.600 500 -17.959 2.972 0.794

YFL005W-YFL004W_246_345 246 100 -13.100 500 -17.950 2.993 1.621

YFL005W-YFL004W_251_350 251 100 -17.700 500 -18.970 2.999 0.423

YFL005W-YFL004W_256_355 256 100 -19.300 500 -19.326 3.109 0.008

YFL005W-YFL004W_261_360 261 100 -18.000 500 -18.349 3.024 0.115

YFL005W-YFL004W_266_365 266 100 -18.900 500 -16.656 2.728 -0.823

YFL005W-YFL004W_271_370 271 100 -17.600 500 -16.298 2.836 -0.459

YFL005W-YFL004W_276_375 276 100 -18.800 500 -15.881 2.723 -1.072

YFL005W-YFL004W_281_380 281 100 -19.500 500 -15.251 2.844 -1.494

YFL005W-YFL004W_286_385 286 100 -18.800 500 -13.977 2.880 -1.675

YFL005W-YFL004W_291_390 291 100 -22.300 500 -14.724 2.695 -2.811

YFL005W-YFL004W_296_395 296 100 -23.300 500 -15.396 2.886 -2.738

YFL005W-YFL004W_301_400 301 100 -23.400 500 -15.082 2.971 -2.799

YFL005W-YFL004W_306_405 306 100 -23.400 500 -14.652 2.692 -3.249

YFL005W-YFL004W_311_410 311 100 -23.400 500 -14.667 2.872 -3.041

YFL005W-YFL004W_316_415 316 100 -22.600 500 -15.195 2.738 -2.704

YFL005W-YFL004W_321_420 321 100 -21.400 500 -14.326 2.613 -2.707

YFL005W-YFL004W_326_425 326 100 -19.600 500 -14.145 2.615 -2.086

YFL005W-YFL004W_331_430 331 100 -17.700 500 -14.147 2.598 -1.367

YFL005W-YFL004W_336_435 336 100 -17.700 500 -12.222 2.547 -2.151

YFL005W-YFL004W_341_440 341 100 -11.800 500 -12.029 2.686 0.085

YFL005W-YFL004W_346_445 346 100 -13.100 500 -13.040 2.681 -0.022

YFL005W-YFL004W_351_450 351 100 -11.700 500 -10.629 2.383 -0.449

YFL005W-YFL004W_356_455 356 100 -11.700 500 -9.513 2.374 -0.921

YFL005W-YFL004W_361_460 361 100 -11.700 500 -10.157 2.500 -0.617

YFL005W-YFL004W_366_465 366 100 -11.100 500 -10.618 2.415 -0.200

YFL005W-YFL004W_371_470 371 100 -11.100 500 -11.039 2.359 -0.026

YFL005W-YFL004W_376_475 376 100 -15.300 500 -12.978 2.570 -0.903

YFL005W-YFL004W_381_480 381 100 -13.100 500 -14.095 2.768 0.360

YFL005W-YFL004W_386_485 386 100 -10.900 500 -13.409 2.513 0.999

YFL005W-YFL004W_391_490 391 100 -10.100 500 -14.862 2.731 1.744

YFL005W-YFL004W_396_495 396 100 -12.500 500 -14.876 2.774 0.856

YFL005W-YFL004W_401_500 401 100 -12.500 500 -15.200 2.901 0.931

YFL005W-YFL004W_406_505 406 100 -12.500 500 -14.975 2.722 0.909

YFL005W-YFL004W_411_510 411 100 -11.500 500 -15.387 2.525 1.539

YFL005W-YFL004W_416_515 416 100 -11.500 500 -14.905 2.650 1.285

YFL005W-YFL004W_421_520 421 100 -11.000 500 -13.527 2.566 0.985

YFL005W-YFL004W_426_525 426 100 -11.000 500 -13.585 2.716 0.952

YFL005W-YFL004W_431_530 431 100 -12.000 500 -13.972 2.594 0.760

YFL005W-YFL004W_436_535 436 100 -12.790 500 -14.152 2.635 0.517

YFL005W-YFL004W_441_540 441 100 -12.890 500 -15.097 2.756 0.801

YFL005W-YFL004W_446_545 446 100 -12.890 500 -16.304 2.779 1.228

YFL005W-YFL004W_451_550 451 100 -15.490 500 -17.770 2.632 0.866

YFL005W-YFL004W_456_555 456 100 -17.300 500 -19.048 2.831 0.618

YFL005W-YFL004W_461_560 461 100 -19.200 500 -20.684 2.977 0.499

YFL005W-YFL004W_466_565 466 100 -17.500 500 -19.354 2.953 0.628

YFL005W-YFL004W_471_570 471 100 -17.300 500 -18.821 3.054 0.498

YFL005W-YFL004W_476_575 476 100 -17.900 500 -17.446 2.975 -0.153

YFL005W-YFL004W_481_580 481 100 -18.000 500 -18.313 2.857 0.109

YFL005W-YFL004W_486_585 486 100 -18.800 500 -16.315 2.804 -0.886

YFL005W-YFL004W_491_590 491 100 -19.300 500 -15.552 2.624 -1.428

YFL005W-YFL004W_496_595 496 100 -20.100 500 -16.761 2.970 -1.124

YFL005W-YFL004W_501_600 501 100 -22.000 500 -19.058 2.980 -0.987

YFL005W-YFL004W_506_605 506 100 -22.100 500 -18.513 2.981 -1.204

YFL005W-YFL004W_511_610 511 100 -23.000 500 -20.345 2.972 -0.893

YFL005W-YFL004W_516_615 516 100 -26.600 500 -22.603 3.181 -1.257

YFL005W-YFL004W_521_620 521 100 -30.500 500 -21.674 3.117 -2.832

YFL005W-YFL004W_526_625 526 100 -30.500 500 -20.573 3.157 -3.144

YFL005W-YFL004W_531_630 531 100 -25.200 500 -18.836 2.990 -2.128

YFL005W-YFL004W_536_635 536 100 -22.900 500 -19.452 2.999 -1.150

YFL005W-YFL004W_541_640 541 100 -22.500 500 -19.568 2.904 -1.010

YFL005W-YFL004W_546_645 546 100 -22.400 500 -18.987 3.172 -1.076

YFL005W-YFL004W_551_650 551 100 -22.200 500 -17.122 3.080 -1.649

YFL005W-YFL004W_556_655 556 100 -21.700 500 -16.486 2.806 -1.858

YFL005W-YFL004W_561_660 561 100 -21.700 500 -16.076 2.719 -2.068

YFL005W-YFL004W_566_665 566 100 -21.400 500 -14.956 2.863 -2.251

YFL005W-YFL004W_571_670 571 100 -14.600 500 -13.479 2.674 -0.419

YFL005W-YFL004W_576_675 576 100 -14.600 500 -13.287 2.728 -0.481

YFL005W-YFL004W_581_680 581 100 -11.100 500 -11.942 2.671 0.315

YFL005W-YFL004W_586_685 586 100 -13.450 500 -14.395 2.673 0.354

YFL005W-YFL004W_591_690 591 100 -13.600 500 -16.593 2.734 1.095

YFL005W-YFL004W_596_695 596 100 -11.030 500 -14.012 2.559 1.165

YFL005W-YFL004W_601_700 601 100 -11.030 500 -12.230 2.562 0.468

YFL005W-YFL004W_606_705 606 100 -9.530 500 -11.119 2.355 0.675

YFL005W-YFL004W_611_710 611 100 -9.530 500 -10.252 2.386 0.303

YFL005W-YFL004W_616_715 616 100 -9.000 500 -8.975 2.432 -0.010

YFL005W-YFL004W_621_720 621 100 -10.900 500 -12.449 2.449 0.633

YFL005W-YFL004W_626_725 626 100 -10.630 500 -13.992 2.839 1.184

YFL005W-YFL004W_631_730 631 100 -9.700 500 -12.983 2.487 1.320

YFL005W-YFL004W_636_735 636 100 -15.800 500 -16.326 2.619 0.201

YFL005W-YFL004W_641_740 641 100 -19.400 500 -19.031 3.075 -0.120

YFL005W-YFL004W_646_745 646 100 -19.800 500 -20.118 2.932 0.108

YFL005W-YFL004W_651_750 651 100 -18.000 500 -18.919 3.029 0.303

YFL005W-YFL004W_656_755 656 100 -18.000 500 -19.501 3.043 0.493

YFL005W-YFL004W_661_760 661 100 -18.000 500 -18.278 3.002 0.093

YFL005W-YFL004W_666_765 666 100 -18.000 500 -18.277 2.861 0.097

YFL005W-YFL004W_671_770 671 100 -20.200 500 -17.686 2.992 -0.840

YFL005W-YFL004W_676_775 676 100 -25.300 500 -16.769 2.920 -2.921

YFL005W-YFL004W_681_780 681 100 -25.300 500 -18.489 2.912 -2.338

YFL005W-YFL004W_686_785 686 100 -25.000 500 -16.982 2.901 -2.764

YFL005W-YFL004W_691_790 691 100 -18.800 500 -15.796 2.580 -1.164

YFL005W-YFL004W_696_795 696 100 -14.100 500 -12.120 2.558 -0.774

YFL005W-YFL004W_701_800 701 100 -12.200 500 -11.020 2.372 -0.497

YFL005W-YFL004W_706_805 706 100 -9.900 500 -10.901 2.750 0.364

YFL005W-YFL004W_711_810 711 100 -12.700 500 -9.456 2.329 -1.393

YFL005W-YFL004W_716_815 716 100 -10.000 500 -8.191 2.192 -0.825

YFL005W-YFL004W_721_820 721 100 -10.000 500 -7.931 2.385 -0.868

YFL005W-YFL004W_726_825 726 100 -10.000 500 -8.912 2.345 -0.464

YFL005W-YFL004W_730_828 730 98 -10.000 500 -7.744 2.121 -1.063

Window size = 105

YFL005W-YFL004W_1_105 1 105 -18.700 500 -18.919 2.970 0.074

YFL005W-YFL004W_6_110 6 105 -21.600 500 -17.425 2.882 -1.449

YFL005W-YFL004W_11_115 11 105 -18.700 500 -14.156 2.758 -1.648

YFL005W-YFL004W_16_120 16 105 -17.600 500 -14.581 2.582 -1.169

YFL005W-YFL004W_21_125 21 105 -16.200 500 -13.758 2.620 -0.932

YFL005W-YFL004W_26_130 26 105 -12.650 500 -13.032 2.501 0.153

YFL005W-YFL004W_31_135 31 105 -13.150 500 -12.309 2.533 -0.332

YFL005W-YFL004W_36_140 36 105 -11.900 500 -11.398 2.539 -0.198

YFL005W-YFL004W_41_145 41 105 -11.500 500 -11.099 2.452 -0.164

YFL005W-YFL004W_46_150 46 105 -7.600 500 -8.054 2.125 0.214

YFL005W-YFL004W_51_155 51 105 -5.600 500 -6.668 1.956 0.546

YFL005W-YFL004W_56_160 56 105 -5.600 500 -5.630 2.095 0.014

YFL005W-YFL004W_61_165 61 105 -4.500 500 -4.815 2.017 0.156

YFL005W-YFL004W_66_170 66 105 -4.500 500 -4.322 1.769 -0.100

YFL005W-YFL004W_71_175 71 105 -5.200 500 -4.551 1.812 -0.358

YFL005W-YFL004W_76_180 76 105 -5.490 500 -6.447 1.932 0.495

YFL005W-YFL004W_81_185 81 105 -7.800 500 -7.898 2.225 0.044

YFL005W-YFL004W_86_190 86 105 -8.300 500 -8.325 2.375 0.011

YFL005W-YFL004W_91_195 91 105 -7.200 500 -8.347 2.227 0.515

YFL005W-YFL004W_96_200 96 105 -7.200 500 -8.964 2.522 0.699

YFL005W-YFL004W_101_205 101 105 -7.400 500 -7.408 2.123 0.004

YFL005W-YFL004W_106_210 106 105 -7.590 500 -7.586 2.136 -0.002

YFL005W-YFL004W_111_215 111 105 -8.800 500 -8.251 2.101 -0.261

YFL005W-YFL004W_116_220 116 105 -8.800 500 -7.853 2.183 -0.434

YFL005W-YFL004W_121_225 121 105 -8.800 500 -7.188 2.079 -0.775

YFL005W-YFL004W_126_230 126 105 -8.800 500 -6.174 1.977 -1.329

YFL005W-YFL004W_131_235 131 105 -6.100 500 -4.118 1.869 -1.061

YFL005W-YFL004W_136_240 136 105 -6.100 500 -4.667 1.941 -0.738

YFL005W-YFL004W_141_245 141 105 -5.200 500 -4.764 2.001 -0.218

YFL005W-YFL004W_146_250 146 105 -5.800 500 -5.302 1.966 -0.253

YFL005W-YFL004W_151_255 151 105 -5.800 500 -6.152 2.036 0.173

YFL005W-YFL004W_156_260 156 105 -5.700 500 -6.755 2.123 0.497

YFL005W-YFL004W_161_265 161 105 -9.600 500 -9.392 2.345 -0.089

YFL005W-YFL004W_166_270 166 105 -10.900 500 -11.372 2.416 0.196

YFL005W-YFL004W_171_275 171 105 -10.900 500 -9.784 2.269 -0.492

YFL005W-YFL004W_176_280 176 105 -10.600 500 -11.556 2.382 0.401

YFL005W-YFL004W_181_285 181 105 -7.800 500 -10.789 2.421 1.234

YFL005W-YFL004W_186_290 186 105 -8.700 500 -11.755 2.314 1.320

YFL005W-YFL004W_191_295 191 105 -9.600 500 -11.089 2.342 0.635

YFL005W-YFL004W_196_300 196 105 -10.600 500 -13.356 2.525 1.091

YFL005W-YFL004W_201_305 201 105 -13.400 500 -15.126 2.833 0.609

YFL005W-YFL004W_206_310 206 105 -13.400 500 -15.045 2.801 0.587

YFL005W-YFL004W_211_315 211 105 -13.400 500 -14.701 2.673 0.487

YFL005W-YFL004W_216_320 216 105 -14.500 500 -15.921 2.639 0.538

YFL005W-YFL004W_221_325 221 105 -15.600 500 -16.816 2.769 0.439

YFL005W-YFL004W_226_330 226 105 -15.600 500 -17.258 2.773 0.598

YFL005W-YFL004W_231_335 231 105 -17.600 500 -18.164 2.941 0.192

YFL005W-YFL004W_236_340 236 105 -17.700 500 -18.605 2.993 0.302

YFL005W-YFL004W_241_345 241 105 -16.600 500 -19.421 2.936 0.961

YFL005W-YFL004W_246_350 246 105 -17.700 500 -20.215 3.224 0.780

YFL005W-YFL004W_251_355 251 105 -20.400 500 -21.249 2.882 0.295

YFL005W-YFL004W_256_360 256 105 -20.600 500 -20.613 2.973 0.005

YFL005W-YFL004W_261_365 261 105 -21.100 500 -19.744 2.965 -0.457

YFL005W-YFL004W_266_370 266 105 -18.900 500 -16.861 2.978 -0.685

YFL005W-YFL004W_271_375 271 105 -18.800 500 -16.619 2.908 -0.750

YFL005W-YFL004W_276_380 276 105 -19.500 500 -17.040 2.925 -0.841

YFL005W-YFL004W_281_385 281 105 -19.500 500 -16.049 2.768 -1.247

YFL005W-YFL004W_286_390 286 105 -22.300 500 -14.820 2.933 -2.550

YFL005W-YFL004W_291_395 291 105 -23.300 500 -15.325 2.793 -2.855

YFL005W-YFL004W_296_400 296 105 -23.400 500 -15.836 2.844 -2.659

YFL005W-YFL004W_301_405 301 105 -24.000 500 -15.697 2.812 -2.952

YFL005W-YFL004W_306_410 306 105 -23.400 500 -15.263 2.847 -2.858

YFL005W-YFL004W_311_415 311 105 -23.400 500 -15.677 2.860 -2.701

YFL005W-YFL004W_316_420 316 105 -23.000 500 -15.565 2.728 -2.725

YFL005W-YFL004W_321_425 321 105 -21.400 500 -16.765 2.870 -1.615

YFL005W-YFL004W_326_430 326 105 -21.200 500 -15.234 2.793 -2.136

YFL005W-YFL004W_331_435 331 105 -19.400 500 -14.787 2.805 -1.645

YFL005W-YFL004W_336_440 336 105 -19.000 500 -12.541 2.454 -2.632

YFL005W-YFL004W_341_445 341 105 -13.700 500 -13.372 2.721 -0.121

YFL005W-YFL004W_346_450 346 105 -14.800 500 -13.741 2.663 -0.398

YFL005W-YFL004W_351_455 351 105 -13.000 500 -10.382 2.539 -1.031

YFL005W-YFL004W_356_460 356 105 -11.700 500 -10.371 2.402 -0.553

YFL005W-YFL004W_361_465 361 105 -14.950 500 -11.884 2.554 -1.201

YFL005W-YFL004W_366_470 366 105 -11.100 500 -11.156 2.396 0.023

YFL005W-YFL004W_371_475 371 105 -15.300 500 -12.861 2.559 -0.953

YFL005W-YFL004W_376_480 376 105 -15.300 500 -14.186 2.670 -0.417

YFL005W-YFL004W_381_485 381 105 -13.100 500 -15.001 2.581 0.737

YFL005W-YFL004W_386_490 386 105 -12.000 500 -16.455 2.703 1.648

YFL005W-YFL004W_391_495 391 105 -12.500 500 -15.412 2.787 1.045

YFL005W-YFL004W_396_500 396 105 -12.500 500 -15.089 2.684 0.965

YFL005W-YFL004W_401_505 401 105 -12.500 500 -15.334 2.610 1.086

YFL005W-YFL004W_406_510 406 105 -12.500 500 -16.258 2.529 1.486

YFL005W-YFL004W_411_515 411 105 -11.900 500 -15.759 2.810 1.373

YFL005W-YFL004W_416_520 416 105 -11.500 500 -15.463 2.902 1.366

YFL005W-YFL004W_421_525 421 105 -13.300 500 -15.325 2.732 0.741

YFL005W-YFL004W_426_530 426 105 -12.200 500 -15.436 2.816 1.149

YFL005W-YFL004W_431_535 431 105 -12.790 500 -15.640 2.584 1.103

YFL005W-YFL004W_436_540 436 105 -12.890 500 -15.240 2.769 0.849

YFL005W-YFL004W_441_545 441 105 -12.890 500 -16.667 2.817 1.341

YFL005W-YFL004W_446_550 446 105 -16.190 500 -18.439 2.722 0.826

YFL005W-YFL004W_451_555 451 105 -18.300 500 -19.436 2.828 0.402

YFL005W-YFL004W_456_560 456 105 -19.400 500 -20.754 2.898 0.467

YFL005W-YFL004W_461_565 461 105 -20.900 500 -21.759 3.031 0.283

YFL005W-YFL004W_466_570 466 105 -18.900 500 -20.127 3.032 0.405

YFL005W-YFL004W_471_575 471 105 -19.700 500 -19.584 3.067 -0.038

YFL005W-YFL004W_476_580 476 105 -18.000 500 -19.082 2.980 0.363

YFL005W-YFL004W_481_585 481 105 -18.800 500 -18.635 2.929 -0.056

YFL005W-YFL004W_486_590 486 105 -19.300 500 -18.369 3.033 -0.307

YFL005W-YFL004W_491_595 491 105 -20.100 500 -16.962 2.938 -1.068

YFL005W-YFL004W_496_600 496 105 -25.800 500 -20.020 3.106 -1.861

YFL005W-YFL004W_501_605 501 105 -22.100 500 -19.600 3.134 -0.798

YFL005W-YFL004W_506_610 506 105 -23.000 500 -20.092 2.872 -1.013

YFL005W-YFL004W_511_615 511 105 -26.600 500 -22.586 3.291 -1.220

YFL005W-YFL004W_516_620 516 105 -30.500 500 -22.968 3.127 -2.408

YFL005W-YFL004W_521_625 521 105 -30.500 500 -22.605 2.929 -2.696

YFL005W-YFL004W_526_630 526 105 -30.500 500 -21.269 3.068 -3.009

YFL005W-YFL004W_531_635 531 105 -25.200 500 -20.258 3.174 -1.557

YFL005W-YFL004W_536_640 536 105 -24.200 500 -20.104 2.948 -1.389

YFL005W-YFL004W_541_645 541 105 -22.500 500 -20.218 2.917 -0.783

YFL005W-YFL004W_546_650 546 105 -24.600 500 -20.516 3.266 -1.250

YFL005W-YFL004W_551_655 551 105 -22.200 500 -17.619 3.053 -1.501

YFL005W-YFL004W_556_660 556 105 -21.700 500 -16.433 2.841 -1.854

YFL005W-YFL004W_561_665 561 105 -21.700 500 -16.585 2.916 -1.754

YFL005W-YFL004W_566_670 566 105 -21.400 500 -14.868 2.850 -2.292

YFL005W-YFL004W_571_675 571 105 -14.600 500 -14.077 2.688 -0.195

YFL005W-YFL004W_576_680 576 105 -14.600 500 -13.835 2.439 -0.314

YFL005W-YFL004W_581_685 581 105 -13.450 500 -14.504 2.691 0.392

YFL005W-YFL004W_586_690 586 105 -15.400 500 -17.704 2.992 0.770

YFL005W-YFL004W_591_695 591 105 -13.930 500 -17.078 2.945 1.069

YFL005W-YFL004W_596_700 596 105 -11.030 500 -13.951 2.570 1.137

YFL005W-YFL004W_601_705 601 105 -11.130 500 -12.512 2.532 0.546

YFL005W-YFL004W_606_710 606 105 -9.830 500 -11.047 2.293 0.531

YFL005W-YFL004W_611_715 611 105 -9.530 500 -10.488 2.436 0.393

YFL005W-YFL004W_616_720 616 105 -10.900 500 -12.700 2.598 0.693

YFL005W-YFL004W_621_725 621 105 -10.900 500 -14.136 2.784 1.162

YFL005W-YFL004W_626_730 626 105 -11.200 500 -15.892 2.756 1.702

YFL005W-YFL004W_631_735 631 105 -15.800 500 -16.389 3.000 0.196

YFL005W-YFL004W_636_740 636 105 -19.400 500 -19.280 2.913 -0.041

YFL005W-YFL004W_641_745 641 105 -20.100 500 -20.735 2.945 0.216

YFL005W-YFL004W_646_750 646 105 -20.400 500 -20.491 3.025 0.030

YFL005W-YFL004W_651_755 651 105 -18.100 500 -20.030 3.012 0.641

YFL005W-YFL004W_656_760 656 105 -18.000 500 -19.322 2.926 0.452

YFL005W-YFL004W_661_765 661 105 -18.000 500 -19.859 3.074 0.605

YFL005W-YFL004W_666_770 666 105 -21.200 500 -18.354 2.960 -0.961

YFL005W-YFL004W_671_775 671 105 -26.100 500 -19.359 2.980 -2.262

YFL005W-YFL004W_676_780 676 105 -25.300 500 -17.692 2.797 -2.720

YFL005W-YFL004W_681_785 681 105 -25.300 500 -17.952 2.854 -2.575

YFL005W-YFL004W_686_790 686 105 -25.000 500 -18.318 2.823 -2.367

YFL005W-YFL004W_691_795 691 105 -18.800 500 -15.629 2.721 -1.165

YFL005W-YFL004W_696_800 696 105 -15.200 500 -13.843 2.612 -0.520

YFL005W-YFL004W_701_805 701 105 -15.600 500 -12.558 2.650 -1.148

YFL005W-YFL004W_706_810 706 105 -14.000 500 -11.553 2.498 -0.980

YFL005W-YFL004W_711_815 711 105 -12.700 500 -9.931 2.375 -1.166

YFL005W-YFL004W_716_820 716 105 -10.000 500 -8.564 2.208 -0.650

YFL005W-YFL004W_721_825 721 105 -10.000 500 -9.259 2.262 -0.328

YFL005W-YFL004W_725_828 725 103 -10.000 500 -9.804 2.436 -0.080

Window size = 110

YFL005W-YFL004W_1_110 1 110 -21.600 500 -19.372 3.141 -0.709

YFL005W-YFL004W_6_115 6 110 -21.600 500 -17.409 2.736 -1.532

YFL005W-YFL004W_11_120 11 110 -18.700 500 -14.517 2.725 -1.535

YFL005W-YFL004W_16_125 16 110 -17.600 500 -14.581 2.560 -1.179

YFL005W-YFL004W_21_130 21 110 -16.200 500 -14.485 2.611 -0.657

YFL005W-YFL004W_26_135 26 110 -14.650 500 -13.735 2.583 -0.354

YFL005W-YFL004W_31_140 31 110 -13.150 500 -12.330 2.481 -0.331

YFL005W-YFL004W_36_145 36 110 -12.800 500 -12.470 2.586 -0.128

YFL005W-YFL004W_41_150 41 110 -11.500 500 -11.214 2.382 -0.120

YFL005W-YFL004W_46_155 46 110 -7.600 500 -8.480 2.274 0.387

YFL005W-YFL004W_51_160 51 110 -5.600 500 -6.847 2.138 0.583

YFL005W-YFL004W_56_165 56 110 -5.600 500 -5.403 1.968 -0.100

YFL005W-YFL004W_61_170 61 110 -4.500 500 -5.372 2.145 0.407

YFL005W-YFL004W_66_175 66 110 -5.200 500 -5.381 1.925 0.094

YFL005W-YFL004W_71_180 71 110 -5.490 500 -5.923 2.074 0.209

YFL005W-YFL004W_76_185 76 110 -7.800 500 -7.832 2.283 0.014

YFL005W-YFL004W_81_190 81 110 -8.300 500 -7.991 2.281 -0.135

YFL005W-YFL004W_86_195 86 110 -8.300 500 -8.796 2.272 0.218

YFL005W-YFL004W_91_200 91 110 -7.200 500 -8.359 2.268 0.511

YFL005W-YFL004W_96_205 96 110 -8.600 500 -9.852 2.463 0.509

YFL005W-YFL004W_101_210 101 110 -7.590 500 -7.556 2.223 -0.015

YFL005W-YFL004W_106_215 106 110 -8.800 500 -7.890 2.247 -0.405

YFL005W-YFL004W_111_220 111 110 -8.800 500 -8.377 2.245 -0.188

YFL005W-YFL004W_116_225 116 110 -8.800 500 -7.914 2.282 -0.388

YFL005W-YFL004W_121_230 121 110 -8.800 500 -6.967 2.126 -0.862

YFL005W-YFL004W_126_235 126 110 -8.800 500 -6.003 1.973 -1.418

YFL005W-YFL004W_131_240 131 110 -6.100 500 -4.904 1.928 -0.620

YFL005W-YFL004W_136_245 136 110 -6.100 500 -4.622 1.929 -0.766

YFL005W-YFL004W_141_250 141 110 -7.100 500 -7.206 2.232 0.047

YFL005W-YFL004W_146_255 146 110 -5.800 500 -6.165 2.088 0.175

YFL005W-YFL004W_151_260 151 110 -5.800 500 -6.730 2.216 0.419

YFL005W-YFL004W_156_265 156 110 -9.600 500 -9.523 2.193 -0.035

YFL005W-YFL004W_161_270 161 110 -10.900 500 -11.405 2.444 0.207

YFL005W-YFL004W_166_275 166 110 -10.900 500 -11.057 2.463 0.064

YFL005W-YFL004W_171_280 171 110 -10.900 500 -11.492 2.390 0.248

YFL005W-YFL004W_176_285 176 110 -10.600 500 -12.433 2.496 0.734

YFL005W-YFL004W_181_290 181 110 -8.700 500 -13.138 2.579 1.721

YFL005W-YFL004W_186_295 186 110 -9.600 500 -11.555 2.597 0.753

YFL005W-YFL004W_191_300 191 110 -10.600 500 -13.659 2.537 1.206

YFL005W-YFL004W_196_305 196 110 -13.400 500 -15.080 2.784 0.603

YFL005W-YFL004W_201_310 201 110 -13.400 500 -15.275 2.767 0.678

YFL005W-YFL004W_206_315 206 110 -13.400 500 -14.877 2.907 0.508

YFL005W-YFL004W_211_320 211 110 -14.500 500 -16.435 2.826 0.685

YFL005W-YFL004W_216_325 216 110 -15.600 500 -17.273 2.901 0.577

YFL005W-YFL004W_221_330 221 110 -15.600 500 -18.035 2.752 0.885

YFL005W-YFL004W_226_335 226 110 -17.600 500 -19.403 3.030 0.595

YFL005W-YFL004W_231_340 231 110 -17.800 500 -19.904 2.976 0.707

YFL005W-YFL004W_236_345 236 110 -17.700 500 -20.278 3.000 0.859

YFL005W-YFL004W_241_350 241 110 -20.400 500 -21.822 3.061 0.464

YFL005W-YFL004W_246_355 246 110 -24.000 500 -22.422 3.076 -0.513

YFL005W-YFL004W_251_360 251 110 -21.700 500 -22.283 2.893 0.202

YFL005W-YFL004W_256_365 256 110 -23.300 500 -21.658 3.344 -0.491

YFL005W-YFL004W_261_370 261 110 -21.100 500 -19.682 3.066 -0.463

YFL005W-YFL004W_266_375 266 110 -19.400 500 -17.493 2.929 -0.651

YFL005W-YFL004W_271_380 271 110 -19.500 500 -17.540 3.078 -0.637

YFL005W-YFL004W_276_385 276 110 -19.500 500 -17.877 2.885 -0.563

YFL005W-YFL004W_281_390 281 110 -23.900 500 -17.339 3.198 -2.051

YFL005W-YFL004W_286_395 286 110 -23.300 500 -15.387 2.774 -2.853

YFL005W-YFL004W_291_400 291 110 -23.400 500 -15.991 3.006 -2.465

YFL005W-YFL004W_296_405 296 110 -24.000 500 -16.598 2.854 -2.593

YFL005W-YFL004W_301_410 301 110 -26.700 500 -16.375 2.893 -3.569

YFL005W-YFL004W_306_415 306 110 -23.400 500 -16.289 2.867 -2.480

YFL005W-YFL004W_311_420 311 110 -23.400 500 -16.043 2.897 -2.539

YFL005W-YFL004W_316_425 316 110 -23.000 500 -17.893 2.933 -1.741

YFL005W-YFL004W_321_430 321 110 -22.700 500 -17.606 2.812 -1.812

YFL005W-YFL004W_326_435 326 110 -21.200 500 -15.950 2.791 -1.881

YFL005W-YFL004W_331_440 331 110 -21.100 500 -15.736 2.933 -1.829

YFL005W-YFL004W_336_445 336 110 -20.600 500 -14.268 2.719 -2.329

YFL005W-YFL004W_341_450 341 110 -14.800 500 -13.975 2.595 -0.318

YFL005W-YFL004W_346_455 346 110 -14.800 500 -13.654 2.493 -0.460

YFL005W-YFL004W_351_460 351 110 -13.200 500 -11.157 2.487 -0.821

YFL005W-YFL004W_356_465 356 110 -14.950 500 -11.922 2.549 -1.188

YFL005W-YFL004W_361_470 361 110 -14.950 500 -12.703 2.473 -0.909

YFL005W-YFL004W_366_475 366 110 -15.300 500 -13.273 2.804 -0.723

YFL005W-YFL004W_371_480 371 110 -15.300 500 -13.836 2.679 -0.547

YFL005W-YFL004W_376_485 376 110 -15.300 500 -15.134 2.911 -0.057

YFL005W-YFL004W_381_490 381 110 -16.100 500 -17.917 2.909 0.625

YFL005W-YFL004W_386_495 386 110 -12.620 500 -16.924 2.708 1.589

YFL005W-YFL004W_391_500 391 110 -12.500 500 -15.710 2.656 1.209

YFL005W-YFL004W_396_505 396 110 -12.500 500 -15.484 2.805 1.064

YFL005W-YFL004W_401_510 401 110 -12.700 500 -16.674 2.744 1.448

YFL005W-YFL004W_406_515 406 110 -12.500 500 -16.115 2.720 1.329

YFL005W-YFL004W_411_520 411 110 -14.000 500 -16.366 2.973 0.796

YFL005W-YFL004W_416_525 416 110 -14.100 500 -17.212 2.898 1.074

YFL005W-YFL004W_421_530 421 110 -17.000 500 -17.304 2.835 0.107

YFL005W-YFL004W_426_535 426 110 -12.790 500 -17.107 2.671 1.616

YFL005W-YFL004W_431_540 431 110 -12.890 500 -16.599 2.694 1.377

YFL005W-YFL004W_436_545 436 110 -14.100 500 -17.188 2.661 1.161

YFL005W-YFL004W_441_550 441 110 -16.190 500 -18.554 2.784 0.849

YFL005W-YFL004W_446_555 446 110 -18.600 500 -20.001 2.958 0.474

YFL005W-YFL004W_451_560 451 110 -19.400 500 -21.151 2.944 0.595

YFL005W-YFL004W_456_565 456 110 -20.900 500 -21.997 2.860 0.384

YFL005W-YFL004W_461_570 461 110 -20.900 500 -22.548 3.015 0.546

YFL005W-YFL004W_466_575 466 110 -21.300 500 -20.748 3.036 -0.182

YFL005W-YFL004W_471_580 471 110 -19.700 500 -21.055 3.000 0.452

YFL005W-YFL004W_476_585 476 110 -18.800 500 -19.411 2.966 0.206

YFL005W-YFL004W_481_590 481 110 -20.700 500 -20.273 2.950 -0.145

YFL005W-YFL004W_486_595 486 110 -21.700 500 -19.234 2.851 -0.865

YFL005W-YFL004W_491_600 491 110 -25.900 500 -20.013 3.013 -1.954

YFL005W-YFL004W_496_605 496 110 -25.800 500 -20.473 3.021 -1.763

YFL005W-YFL004W_501_610 501 110 -23.000 500 -20.933 3.116 -0.663

YFL005W-YFL004W_506_615 506 110 -26.600 500 -22.215 3.180 -1.379

YFL005W-YFL004W_511_620 511 110 -30.500 500 -22.580 3.107 -2.549

YFL005W-YFL004W_516_625 516 110 -30.500 500 -24.024 3.121 -2.075

YFL005W-YFL004W_521_630 521 110 -30.500 500 -23.834 3.093 -2.155

YFL005W-YFL004W_526_635 526 110 -30.500 500 -22.643 3.019 -2.603

YFL005W-YFL004W_531_640 531 110 -29.800 500 -20.988 3.154 -2.794

YFL005W-YFL004W_536_645 536 110 -24.200 500 -21.282 3.179 -0.918

YFL005W-YFL004W_541_650 541 110 -24.600 500 -21.820 2.974 -0.935

YFL005W-YFL004W_546_655 546 110 -24.600 500 -20.642 2.963 -1.336

YFL005W-YFL004W_551_660 551 110 -22.200 500 -17.650 2.983 -1.525

YFL005W-YFL004W_556_665 556 110 -21.700 500 -16.810 2.680 -1.825

YFL005W-YFL004W_561_670 561 110 -21.700 500 -16.445 2.631 -1.997

YFL005W-YFL004W_566_675 566 110 -21.400 500 -15.830 2.884 -1.932

YFL005W-YFL004W_571_680 571 110 -15.000 500 -14.888 2.725 -0.041

YFL005W-YFL004W_576_685 576 110 -16.400 500 -16.772 2.952 0.126

YFL005W-YFL004W_581_690 581 110 -15.400 500 -17.743 2.940 0.797

YFL005W-YFL004W_586_695 586 110 -15.400 500 -17.675 2.740 0.830

YFL005W-YFL004W_591_700 591 110 -14.830 500 -17.112 2.695 0.847

YFL005W-YFL004W_596_705 596 110 -11.130 500 -14.287 2.714 1.163

YFL005W-YFL004W_601_710 601 110 -11.130 500 -12.559 2.559 0.558

YFL005W-YFL004W_606_715 606 110 -10.130 500 -11.582 2.417 0.601

YFL005W-YFL004W_611_720 611 110 -12.430 500 -14.387 2.620 0.747

YFL005W-YFL004W_616_725 616 110 -10.900 500 -14.256 2.681 1.252

YFL005W-YFL004W_621_730 621 110 -13.130 500 -16.040 2.550 1.141

YFL005W-YFL004W_626_735 626 110 -17.030 500 -19.372 3.016 0.777

YFL005W-YFL004W_631_740 631 110 -19.400 500 -19.016 2.731 -0.141

YFL005W-YFL004W_636_745 636 110 -20.300 500 -21.079 3.042 0.256

YFL005W-YFL004W_641_750 641 110 -20.600 500 -20.876 3.063 0.090

YFL005W-YFL004W_646_755 646 110 -20.400 500 -21.328 3.080 0.301

YFL005W-YFL004W_651_760 651 110 -18.100 500 -19.855 3.079 0.570

YFL005W-YFL004W_656_765 656 110 -18.600 500 -21.153 2.947 0.866

YFL005W-YFL004W_661_770 661 110 -21.200 500 -20.200 3.168 -0.316

YFL005W-YFL004W_666_775 666 110 -26.100 500 -19.593 2.927 -2.223

Window size = 125

YFL005W-YFL004W_1_125 1 125 -21.900 500 -19.271 2.909 -0.904

YFL005W-YFL004W_6_130 6 125 -21.600 500 -18.296 3.084 -1.071

YFL005W-YFL004W_11_135 11 125 -20.700 500 -16.069 2.748 -1.685

YFL005W-YFL004W_16_140 16 125 -19.600 500 -16.149 2.843 -1.214

YFL005W-YFL004W_21_145 21 125 -18.200 500 -16.187 2.790 -0.722

YFL005W-YFL004W_26_150 26 125 -14.650 500 -15.036 2.626 0.147

YFL005W-YFL004W_31_155 31 125 -14.300 500 -13.981 2.848 -0.112

YFL005W-YFL004W_36_160 36 125 -13.000 500 -13.128 2.759 0.047

YFL005W-YFL004W_41_165 41 125 -11.500 500 -11.361 2.333 -0.060

YFL005W-YFL004W_46_170 46 125 -7.600 500 -9.025 2.313 0.616

YFL005W-YFL004W_51_175 51 125 -6.200 500 -8.477 2.370 0.961

YFL005W-YFL004W_56_180 56 125 -5.700 500 -8.461 2.314 1.193

YFL005W-YFL004W_61_185 61 125 -7.800 500 -9.180 2.403 0.574

YFL005W-YFL004W_66_190 66 125 -8.300 500 -7.982 2.136 -0.149

YFL005W-YFL004W_71_195 71 125 -8.300 500 -7.590 2.096 -0.339

YFL005W-YFL004W_76_200 76 125 -8.300 500 -8.348 2.441 0.020

YFL005W-YFL004W_81_205 81 125 -9.000 500 -9.311 2.253 0.138

YFL005W-YFL004W_86_210 86 125 -9.890 500 -9.921 2.545 0.012

YFL005W-YFL004W_91_215 91 125 -10.000 500 -9.756 2.433 -0.100

YFL005W-YFL004W_96_220 96 125 -10.000 500 -10.217 2.320 0.094

YFL005W-YFL004W_101_225 101 125 -8.800 500 -7.943 2.285 -0.375

YFL005W-YFL004W_106_230 106 125 -8.800 500 -7.589 2.223 -0.545

YFL005W-YFL004W_111_235 111 125 -8.800 500 -7.868 2.271 -0.411

YFL005W-YFL004W_116_240 116 125 -8.800 500 -8.295 2.411 -0.210

YFL005W-YFL004W_121_245 121 125 -8.800 500 -7.487 2.151 -0.610

YFL005W-YFL004W_126_250 126 125 -10.700 500 -8.997 2.366 -0.720

YFL005W-YFL004W_131_255 131 125 -8.000 500 -7.421 2.203 -0.263

YFL005W-YFL004W_136_260 136 125 -8.000 500 -8.140 2.190 0.064

YFL005W-YFL004W_141_265 141 125 -11.000 500 -11.466 2.415 0.193

YFL005W-YFL004W_146_270 146 125 -11.000 500 -12.205 2.557 0.471

YFL005W-YFL004W_151_275 151 125 -11.000 500 -11.705 2.482 0.284

YFL005W-YFL004W_156_280 156 125 -12.400 500 -13.108 2.558 0.277

YFL005W-YFL004W_161_285 161 125 -14.100 500 -14.406 2.623 0.117

YFL005W-YFL004W_166_290 166 125 -15.800 500 -16.154 2.570 0.138

YFL005W-YFL004W_171_295 171 125 -13.400 500 -14.554 2.500 0.462

YFL005W-YFL004W_176_300 176 125 -13.500 500 -16.529 2.684 1.128

YFL005W-YFL004W_181_305 181 125 -13.400 500 -16.308 2.568 1.132

YFL005W-YFL004W_186_310 186 125 -13.400 500 -15.489 2.482 0.841

YFL005W-YFL004W_191_315 191 125 -13.400 500 -14.950 2.553 0.607

YFL005W-YFL004W_196_320 196 125 -14.500 500 -16.604 2.728 0.771

YFL005W-YFL004W_201_325 201 125 -15.600 500 -18.133 2.945 0.860

YFL005W-YFL004W_206_330 206 125 -15.600 500 -18.990 3.039 1.116

YFL005W-YFL004W_211_335 211 125 -17.600 500 -21.439 3.086 1.244

YFL005W-YFL004W_216_340 216 125 -17.800 500 -22.036 3.030 1.398

YFL005W-YFL004W_221_345 221 125 -17.800 500 -22.996 3.177 1.636

YFL005W-YFL004W_226_350 226 125 -23.100 500 -24.692 3.354 0.474

YFL005W-YFL004W_231_355 231 125 -26.800 500 -25.720 3.265 -0.331

YFL005W-YFL004W_236_360 236 125 -28.100 500 -25.729 3.199 -0.741

YFL005W-YFL004W_241_365 241 125 -28.100 500 -25.723 3.214 -0.740

YFL005W-YFL004W_246_370 246 125 -26.300 500 -24.681 3.267 -0.496

YFL005W-YFL004W_251_375 251 125 -24.400 500 -23.643 3.186 -0.238

YFL005W-YFL004W_256_380 256 125 -23.800 500 -23.204 3.135 -0.190

YFL005W-YFL004W_261_385 261 125 -22.400 500 -21.894 3.214 -0.157

YFL005W-YFL004W_266_390 266 125 -24.800 500 -20.190 3.280 -1.405

YFL005W-YFL004W_271_395 271 125 -25.900 500 -20.199 3.079 -1.852

YFL005W-YFL004W_276_400 276 125 -25.900 500 -20.421 3.207 -1.708

YFL005W-YFL004W_281_405 281 125 -25.600 500 -19.531 3.050 -1.990

YFL005W-YFL004W_286_410 286 125 -27.000 500 -17.942 2.933 -3.088

YFL005W-YFL004W_291_415 291 125 -27.000 500 -18.891 3.106 -2.611

YFL005W-YFL004W_296_420 296 125 -27.000 500 -19.291 3.326 -2.318

YFL005W-YFL004W_301_425 301 125 -26.700 500 -20.545 3.006 -2.047

YFL005W-YFL004W_306_430 306 125 -25.000 500 -19.892 3.171 -1.611

YFL005W-YFL004W_311_435 311 125 -25.000 500 -20.513 3.110 -1.443

YFL005W-YFL004W_316_440 316 125 -24.800 500 -20.679 3.094 -1.332

YFL005W-YFL004W_321_445 321 125 -26.400 500 -20.455 3.091 -1.923

YFL005W-YFL004W_326_450 326 125 -24.200 500 -18.536 2.796 -2.026

YFL005W-YFL004W_331_455 331 125 -23.900 500 -17.615 2.862 -2.196

YFL005W-YFL004W_336_460 336 125 -20.900 500 -15.271 2.839 -1.983

YFL005W-YFL004W_341_465 341 125 -16.950 500 -15.995 2.850 -0.335

YFL005W-YFL004W_346_470 346 125 -15.250 500 -16.533 2.793 0.459

YFL005W-YFL004W_351_475 351 125 -17.400 500 -15.462 2.761 -0.702

YFL005W-YFL004W_356_480 356 125 -16.250 500 -15.927 2.732 -0.118

YFL005W-YFL004W_361_485 361 125 -16.500 500 -16.906 2.811 0.144

YFL005W-YFL004W_366_490 366 125 -18.300 500 -18.462 2.769 0.059

YFL005W-YFL004W_371_495 371 125 -18.300 500 -18.641 2.916 0.117

YFL005W-YFL004W_376_500 376 125 -18.300 500 -18.688 2.890 0.134

YFL005W-YFL004W_381_505 381 125 -16.100 500 -18.749 2.909 0.911

YFL005W-YFL004W_386_510 386 125 -14.800 500 -18.597 2.846 1.334

YFL005W-YFL004W_391_515 391 125 -15.600 500 -17.542 2.729 0.712

YFL005W-YFL004W_396_520 396 125 -14.800 500 -17.757 2.967 0.997

YFL005W-YFL004W_401_525 401 125 -14.500 500 -19.011 2.811 1.605

YFL005W-YFL004W_406_530 406 125 -17.800 500 -20.373 2.992 0.860

YFL005W-YFL004W_411_535 411 125 -20.400 500 -21.483 3.285 0.330

YFL005W-YFL004W_416_540 416 125 -20.400 500 -21.460 3.021 0.351

YFL005W-YFL004W_421_545 421 125 -19.300 500 -21.389 3.137 0.666

YFL005W-YFL004W_426_550 426 125 -19.100 500 -21.877 3.160 0.879

YFL005W-YFL004W_431_555 431 125 -19.600 500 -22.093 2.985 0.835

YFL005W-YFL004W_436_560 436 125 -21.300 500 -22.229 2.913 0.319

YFL005W-YFL004W_441_565 441 125 -21.400 500 -23.465 3.237 0.638

YFL005W-YFL004W_446_570 446 125 -21.500 500 -23.907 3.277 0.735

YFL005W-YFL004W_451_575 451 125 -23.100 500 -24.181 3.273 0.330

YFL005W-YFL004W_456_580 456 125 -23.600 500 -25.524 3.187 0.604

YFL005W-YFL004W_461_585 461 125 -24.300 500 -25.736 3.370 0.426

YFL005W-YFL004W_466_590 466 125 -24.800 500 -24.481 3.043 -0.105

YFL005W-YFL004W_471_595 471 125 -23.200 500 -24.420 3.281 0.372

YFL005W-YFL004W_476_600 476 125 -27.200 500 -25.228 3.321 -0.594

YFL005W-YFL004W_481_605 481 125 -27.400 500 -24.939 3.301 -0.746

YFL005W-YFL004W_486_610 486 125 -32.700 500 -24.218 3.225 -2.630

YFL005W-YFL004W_491_615 491 125 -27.000 500 -24.293 3.110 -0.870

YFL005W-YFL004W_496_620 496 125 -30.500 500 -24.427 3.228 -1.882

YFL005W-YFL004W_501_625 501 125 -30.500 500 -24.308 3.049 -2.031

YFL005W-YFL004W_506_630 506 125 -30.500 500 -24.458 3.323 -1.818

YFL005W-YFL004W_511_635 511 125 -31.600 500 -25.869 3.167 -1.810

YFL005W-YFL004W_516_640 516 125 -31.800 500 -26.693 3.374 -1.514

YFL005W-YFL004W_521_645 521 125 -31.800 500 -27.247 3.230 -1.410

YFL005W-YFL004W_526_650 526 125 -33.400 500 -26.134 3.374 -2.153

YFL005W-YFL004W_531_655 531 125 -29.800 500 -23.647 3.327 -1.849

YFL005W-YFL004W_536_660 536 125 -26.300 500 -22.791 3.001 -1.169

YFL005W-YFL004W_541_665 541 125 -24.600 500 -22.628 3.064 -0.643

YFL005W-YFL004W_546_670 546 125 -24.600 500 -21.059 2.985 -1.186

YFL005W-YFL004W_551_675 551 125 -22.200 500 -18.810 2.935 -1.155

YFL005W-YFL004W_556_680 556 125 -22.180 500 -18.216 2.933 -1.352

YFL005W-YFL004W_561_685 561 125 -23.700 500 -20.691 3.146 -0.956

YFL005W-YFL004W_566_690 566 125 -26.500 500 -22.374 3.079 -1.340

YFL005W-YFL004W_571_695 571 125 -19.000 500 -21.483 3.018 0.823

YFL005W-YFL004W_576_700 576 125 -18.900 500 -20.399 3.054 0.491

YFL005W-YFL004W_581_705 581 125 -15.850 500 -18.148 2.897 0.793

YFL005W-YFL004W_586_710 586 125 -15.850 500 -18.063 2.750 0.805

YFL005W-YFL004W_591_715 591 125 -15.530 500 -17.582 2.785 0.737

YFL005W-YFL004W_596_720 596 125 -15.330 500 -18.257 2.704 1.083

YFL005W-YFL004W_601_725 601 125 -15.330 500 -17.779 2.819 0.868

YFL005W-YFL004W_606_730 606 125 -15.630 500 -18.887 2.950 1.104

YFL005W-YFL004W_611_735 611 125 -19.230 500 -21.025 2.910 0.617

YFL005W-YFL004W_616_740 616 125 -20.800 500 -22.406 3.060 0.525

YFL005W-YFL004W_621_745 621 125 -21.900 500 -24.254 3.158 0.745

YFL005W-YFL004W_626_750 626 125 -21.700 500 -24.230 3.105 0.815

YFL005W-YFL004W_631_755 631 125 -21.100 500 -22.958 3.046 0.610

YFL005W-YFL004W_636_760 636 125 -21.100 500 -22.923 3.015 0.605

YFL005W-YFL004W_641_765 641 125 -21.800 500 -24.244 3.279 0.745

YFL005W-YFL004W_646_770 646 125 -24.500 500 -23.469 3.214 -0.321

YFL005W-YFL004W_651_775 651 125 -27.100 500 -23.744 3.300 -1.017

YFL005W-YFL004W_656_780 656 125 -27.700 500 -24.011 3.245 -1.137

YFL005W-YFL004W_661_785 661 125 -27.000 500 -22.140 3.100 -1.568

YFL005W-YFL004W_666_790 666 125 -26.100 500 -21.978 3.213 -1.283

YFL005W-YFL004W_671_795 671 125 -26.100 500 -21.096 2.899 -1.726

YFL005W-YFL004W_676_800 676 125 -25.300 500 -19.939 2.944 -1.821

YFL005W-YFL004W_681_805 681 125 -28.200 500 -22.154 3.138 -1.926

YFL005W-YFL004W_686_810 686 125 -27.200 500 -21.560 3.101 -1.819

YFL005W-YFL004W_691_815 691 125 -21.000 500 -18.953 2.955 -0.693

YFL005W-YFL004W_696_820 696 125 -19.200 500 -17.114 2.839 -0.735

YFL005W-YFL004W_701_825 701 125 -15.700 500 -15.590 2.822 -0.039

YFL005W-YFL004W_705_828 705 123 -14.000 500 -14.642 2.718 0.236

Window size = 130

YFL005W-YFL004W_1_130 1 130 -21.900 500 -20.290 3.170 -0.508

YFL005W-YFL004W_6_135 6 130 -23.600 500 -19.256 2.832 -1.534

YFL005W-YFL004W_11_140 11 130 -20.700 500 -15.835 2.719 -1.789

YFL005W-YFL004W_16_145 16 130 -19.600 500 -17.341 2.838 -0.796

YFL005W-YFL004W_21_150 21 130 -19.090 500 -16.457 2.815 -0.935

YFL005W-YFL004W_26_155 26 130 -14.650 500 -15.433 2.641 0.296

YFL005W-YFL004W_31_160 31 130 -14.300 500 -14.181 2.829 -0.042

YFL005W-YFL004W_36_165 36 130 -13.000 500 -12.965 2.691 -0.013

YFL005W-YFL004W_41_170 41 130 -11.500 500 -11.956 2.525 0.180

YFL005W-YFL004W_46_175 46 130 -8.650 500 -9.858 2.209 0.547

YFL005W-YFL004W_51_180 51 130 -6.390 500 -9.554 2.338 1.353

YFL005W-YFL004W_56_185 56 130 -7.800 500 -9.870 2.369 0.874

YFL005W-YFL004W_61_190 61 130 -8.300 500 -8.991 2.385 0.290

YFL005W-YFL004W_66_195 66 130 -8.300 500 -8.405 2.308 0.045

YFL005W-YFL004W_71_200 71 130 -8.300 500 -7.897 2.250 -0.179

YFL005W-YFL004W_76_205 76 130 -9.000 500 -9.154 2.262 0.068

YFL005W-YFL004W_81_210 81 130 -9.890 500 -9.547 2.324 -0.148

YFL005W-YFL004W_86_215 86 130 -11.100 500 -10.018 2.244 -0.482

YFL005W-YFL004W_91_220 91 130 -10.000 500 -9.752 2.316 -0.107

YFL005W-YFL004W_96_225 96 130 -10.000 500 -10.456 2.354 0.194

YFL005W-YFL004W_101_230 101 130 -8.800 500 -7.716 2.354 -0.461

YFL005W-YFL004W_106_235 106 130 -8.800 500 -7.418 2.145 -0.644

YFL005W-YFL004W_111_240 111 130 -8.800 500 -8.601 2.208 -0.090

YFL005W-YFL004W_116_245 116 130 -8.800 500 -8.067 2.152 -0.340

YFL005W-YFL004W_121_250 121 130 -10.700 500 -9.633 2.409 -0.443

YFL005W-YFL004W_126_255 126 130 -10.700 500 -9.535 2.377 -0.490

YFL005W-YFL004W_131_260 131 130 -8.000 500 -8.505 2.276 0.222

YFL005W-YFL004W_136_265 136 130 -11.900 500 -11.069 2.508 -0.332

YFL005W-YFL004W_141_270 141 130 -12.300 500 -13.672 2.655 0.517

YFL005W-YFL004W_146_275 146 130 -11.000 500 -11.983 2.526 0.389

YFL005W-YFL004W_151_280 151 130 -12.400 500 -13.498 2.464 0.446

YFL005W-YFL004W_156_285 156 130 -14.100 500 -14.565 2.662 0.175

YFL005W-YFL004W_161_290 161 130 -17.500 500 -16.323 2.706 -0.435

YFL005W-YFL004W_166_295 166 130 -15.800 500 -15.833 2.776 0.012

YFL005W-YFL004W_171_300 171 130 -13.800 500 -16.938 2.971 1.056

YFL005W-YFL004W_176_305 176 130 -16.900 500 -18.270 2.841 0.482

YFL005W-YFL004W_181_310 181 130 -13.400 500 -16.644 2.703 1.200

YFL005W-YFL004W_186_315 186 130 -13.400 500 -15.299 2.617 0.726

YFL005W-YFL004W_191_320 191 130 -14.500 500 -16.982 2.850 0.871

YFL005W-YFL004W_196_325 196 130 -15.600 500 -17.955 2.833 0.831

YFL005W-YFL004W_201_330 201 130 -15.600 500 -19.174 3.133 1.141

YFL005W-YFL004W_206_335 206 130 -17.600 500 -21.389 3.166 1.197

YFL005W-YFL004W_211_340 211 130 -17.800 500 -23.207 3.271 1.653

YFL005W-YFL004W_216_345 216 130 -17.800 500 -23.492 2.867 1.985

YFL005W-YFL004W_221_350 221 130 -23.100 500 -25.311 3.156 0.701

YFL005W-YFL004W_226_355 226 130 -26.800 500 -26.724 3.423 -0.022

YFL005W-YFL004W_231_360 231 130 -28.100 500 -26.648 3.283 -0.442

YFL005W-YFL004W_236_365 236 130 -28.100 500 -26.284 3.355 -0.541

YFL005W-YFL004W_241_370 241 130 -28.100 500 -26.110 3.297 -0.604

YFL005W-YFL004W_246_375 246 130 -26.300 500 -25.222 3.240 -0.333

YFL005W-YFL004W_251_380 251 130 -24.500 500 -24.599 3.309 0.030

YFL005W-YFL004W_256_385 256 130 -24.200 500 -24.313 3.243 0.035

YFL005W-YFL004W_261_390 261 130 -26.500 500 -22.808 3.164 -1.167

YFL005W-YFL004W_266_395 266 130 -25.900 500 -20.929 3.074 -1.617

YFL005W-YFL004W_271_400 271 130 -25.900 500 -21.000 3.181 -1.540

YFL005W-YFL004W_276_405 276 130 -27.400 500 -21.502 3.089 -1.910

YFL005W-YFL004W_281_410 281 130 -28.600 500 -20.190 3.102 -2.711

YFL005W-YFL004W_286_415 286 130 -27.000 500 -18.875 3.054 -2.660

YFL005W-YFL004W_291_420 291 130 -27.000 500 -19.502 3.275 -2.289

YFL005W-YFL004W_296_425 296 130 -27.000 500 -21.576 3.239 -1.675

YFL005W-YFL004W_301_430 301 130 -28.300 500 -20.858 3.045 -2.444

YFL005W-YFL004W_306_435 306 130 -25.700 500 -21.330 3.172 -1.377

YFL005W-YFL004W_311_440 311 130 -25.500 500 -21.172 3.022 -1.432

YFL005W-YFL004W_316_445 316 130 -26.500 500 -21.835 3.144 -1.484

YFL005W-YFL004W_321_450 321 130 -27.100 500 -21.281 3.072 -1.894

YFL005W-YFL004W_326_455 326 130 -24.200 500 -18.541 2.804 -2.018

YFL005W-YFL004W_331_460 331 130 -23.900 500 -18.368 2.979 -1.857

YFL005W-YFL004W_336_465 336 130 -23.850 500 -16.852 2.937 -2.383

YFL005W-YFL004W_341_470 341 130 -16.950 500 -16.811 2.817 -0.050

YFL005W-YFL004W_346_475 346 130 -19.000 500 -18.345 2.847 -0.230

YFL005W-YFL004W_351_480 351 130 -18.150 500 -16.653 2.813 -0.532

YFL005W-YFL004W_356_485 356 130 -16.500 500 -16.979 2.817 0.170

YFL005W-YFL004W_361_490 361 130 -20.800 500 -19.809 2.888 -0.343

YFL005W-YFL004W_366_495 366 130 -18.300 500 -19.014 2.863 0.250

YFL005W-YFL004W_371_500 371 130 -18.300 500 -18.589 2.886 0.100

YFL005W-YFL004W_376_505 376 130 -18.300 500 -18.772 2.752 0.171

YFL005W-YFL004W_381_510 381 130 -16.500 500 -20.142 2.991 1.217

YFL005W-YFL004W_386_515 386 130 -15.600 500 -18.919 2.783 1.193

YFL005W-YFL004W_391_520 391 130 -15.600 500 -18.153 3.010 0.848

YFL005W-YFL004W_396_525 396 130 -14.800 500 -19.485 2.949 1.589

YFL005W-YFL004W_401_530 401 130 -17.800 500 -20.933 2.882 1.087

YFL005W-YFL004W_406_535 406 130 -20.400 500 -21.945 3.090 0.500

YFL005W-YFL004W_411_540 411 130 -20.400 500 -22.672 2.980 0.762

YFL005W-YFL004W_416_545 416 130 -20.400 500 -23.364 3.171 0.935

YFL005W-YFL004W_421_550 421 130 -20.500 500 -23.785 3.192 1.029

YFL005W-YFL004W_426_555 426 130 -22.000 500 -23.609 3.077 0.523

YFL005W-YFL004W_431_560 431 130 -21.600 500 -24.051 3.165 0.775

YFL005W-YFL004W_436_565 436 130 -22.800 500 -23.990 3.351 0.355

YFL005W-YFL004W_441_570 441 130 -22.000 500 -24.292 3.262 0.703

YFL005W-YFL004W_446_575 446 130 -23.400 500 -24.701 3.090 0.421

YFL005W-YFL004W_451_580 451 130 -23.600 500 -25.903 3.196 0.720

YFL005W-YFL004W_456_585 456 130 -25.000 500 -25.936 3.290 0.285

YFL005W-YFL004W_461_590 461 130 -25.600 500 -27.232 3.385 0.482

YFL005W-YFL004W_466_595 466 130 -25.600 500 -25.663 3.148 0.020

YFL005W-YFL004W_471_600 471 130 -27.800 500 -27.239 3.186 -0.176

YFL005W-YFL004W_476_605 476 130 -27.400 500 -25.855 3.234 -0.478

YFL005W-YFL004W_481_610 481 130 -33.300 500 -26.613 3.254 -2.055

YFL005W-YFL004W_486_615 486 130 -32.700 500 -26.792 3.160 -1.870

YFL005W-YFL004W_491_620 491 130 -30.500 500 -24.633 3.358 -1.747

YFL005W-YFL004W_496_625 496 130 -30.500 500 -25.096 3.398 -1.590

YFL005W-YFL004W_501_630 501 130 -30.700 500 -25.439 3.020 -1.742

YFL005W-YFL004W_506_635 506 130 -31.600 500 -25.576 3.154 -1.910

YFL005W-YFL004W_511_640 511 130 -31.800 500 -26.627 3.317 -1.559

YFL005W-YFL004W_516_645 516 130 -31.800 500 -27.596 3.153 -1.333

YFL005W-YFL004W_521_650 521 130 -33.400 500 -28.151 3.236 -1.622

YFL005W-YFL004W_526_655 526 130 -33.400 500 -26.337 3.491 -2.024

YFL005W-YFL004W_531_660 531 130 -29.800 500 -24.358 3.273 -1.663

YFL005W-YFL004W_536_665 536 130 -26.300 500 -23.172 3.149 -0.993

YFL005W-YFL004W_541_670 541 130 -24.600 500 -22.604 3.335 -0.599

YFL005W-YFL004W_546_675 546 130 -24.900 500 -22.006 3.064 -0.944

YFL005W-YFL004W_551_680 551 130 -22.200 500 -19.533 2.890 -0.923

YFL005W-YFL004W_556_685 556 130 -23.700 500 -21.273 3.146 -0.772

YFL005W-YFL004W_561_690 561 130 -26.800 500 -24.068 3.330 -0.820

YFL005W-YFL004W_566_695 566 130 -26.530 500 -22.878 3.017 -1.211

YFL005W-YFL004W_571_700 571 130 -20.130 500 -22.068 3.055 0.634

YFL005W-YFL004W_576_705 576 130 -18.900 500 -20.755 3.080 0.602

YFL005W-YFL004W_581_710 581 130 -15.850 500 -18.078 2.764 0.806

YFL005W-YFL004W_586_715 586 130 -15.850 500 -18.578 2.814 0.969

YFL005W-YFL004W_591_720 591 130 -16.360 500 -21.413 3.002 1.683

YFL005W-YFL004W_596_725 596 130 -15.330 500 -19.524 2.898 1.447

YFL005W-YFL004W_601_730 601 130 -15.630 500 -19.879 3.134 1.356

YFL005W-YFL004W_606_735 606 130 -19.530 500 -21.906 3.207 0.741

YFL005W-YFL004W_611_740 611 130 -22.330 500 -23.764 3.108 0.461

YFL005W-YFL004W_616_745 616 130 -23.600 500 -24.761 3.229 0.360

YFL005W-YFL004W_621_750 621 130 -21.900 500 -24.667 3.114 0.888

YFL005W-YFL004W_626_755 626 130 -21.900 500 -25.477 3.155 1.134

YFL005W-YFL004W_631_760 631 130 -21.200 500 -22.793 3.256 0.489

YFL005W-YFL004W_636_765 636 130 -21.800 500 -25.091 3.215 1.024

YFL005W-YFL004W_641_770 641 130 -24.800 500 -24.867 3.300 0.020

YFL005W-YFL004W_646_775 646 130 -27.100 500 -25.116 3.358 -0.591

YFL005W-YFL004W_651_780 651 130 -28.000 500 -25.001 3.257 -0.921

YFL005W-YFL004W_656_785 656 130 -27.700 500 -23.541 3.250 -1.279

YFL005W-YFL004W_661_790 661 130 -28.800 500 -23.737 3.206 -1.579

YFL005W-YFL004W_666_795 666 130 -26.100 500 -21.766 3.189 -1.359

YFL005W-YFL004W_671_800 671 130 -26.100 500 -22.898 3.078 -1.040

YFL005W-YFL004W_676_805 676 130 -28.200 500 -21.346 3.020 -2.270

YFL005W-YFL004W_681_810 681 130 -28.300 500 -22.506 3.155 -1.837

YFL005W-YFL004W_686_815 686 130 -27.200 500 -21.605 2.888 -1.938

YFL005W-YFL004W_691_820 691 130 -21.000 500 -19.940 3.113 -0.340

YFL005W-YFL004W_696_825 696 130 -19.200 500 -18.220 2.936 -0.334

YFL005W-YFL004W_700_828 700 128 -15.900 500 -16.173 2.892 0.094

Window size = 135

YFL005W-YFL004W_1_135 1 135 -23.900 500 -21.036 3.043 -0.941

YFL005W-YFL004W_6_140 6 135 -23.600 500 -19.291 2.804 -1.537

YFL005W-YFL004W_11_145 11 135 -20.700 500 -17.250 2.796 -1.234

YFL005W-YFL004W_16_150 16 135 -20.790 500 -17.175 2.773 -1.304

YFL005W-YFL004W_21_155 21 135 -19.090 500 -16.425 2.860 -0.932

YFL005W-YFL004W_26_160 26 135 -14.650 500 -15.440 2.663 0.296

YFL005W-YFL004W_31_165 31 135 -14.300 500 -13.787 2.571 -0.200

YFL005W-YFL004W_36_170 36 135 -13.000 500 -13.749 2.732 0.274

YFL005W-YFL004W_41_175 41 135 -12.200 500 -13.124 2.580 0.358

YFL005W-YFL004W_46_180 46 135 -8.650 500 -11.288 2.408 1.095

YFL005W-YFL004W_51_185 51 135 -8.700 500 -11.183 2.343 1.060

YFL005W-YFL004W_56_190 56 135 -8.300 500 -9.850 2.531 0.612

YFL005W-YFL004W_61_195 61 135 -8.300 500 -9.347 2.320 0.451

YFL005W-YFL004W_66_200 66 135 -8.300 500 -8.657 2.259 0.158

YFL005W-YFL004W_71_205 71 135 -9.000 500 -8.543 2.217 -0.206

YFL005W-YFL004W_76_210 76 135 -9.890 500 -9.028 2.334 -0.369

YFL005W-YFL004W_81_215 81 135 -11.100 500 -9.762 2.398 -0.558

YFL005W-YFL004W_86_220 86 135 -11.100 500 -10.132 2.277 -0.425

YFL005W-YFL004W_91_225 91 135 -10.000 500 -10.118 2.460 0.048

YFL005W-YFL004W_96_230 96 135 -10.700 500 -10.068 2.279 -0.277

YFL005W-YFL004W_101_235 101 135 -8.800 500 -7.564 2.285 -0.541

YFL005W-YFL004W_106_240 106 135 -8.800 500 -8.243 2.254 -0.247

YFL005W-YFL004W_111_245 111 135 -8.800 500 -8.534 2.219 -0.120

YFL005W-YFL004W_116_250 116 135 -10.700 500 -10.483 2.415 -0.090

YFL005W-YFL004W_121_255 121 135 -10.700 500 -10.055 2.373 -0.272

YFL005W-YFL004W_126_260 126 135 -10.700 500 -9.908 2.338 -0.339

YFL005W-YFL004W_131_265 131 135 -11.900 500 -11.024 2.273 -0.386

YFL005W-YFL004W_136_270 136 135 -13.200 500 -13.268 2.612 0.026

YFL005W-YFL004W_141_275 141 135 -12.400 500 -13.636 2.606 0.474

YFL005W-YFL004W_146_280 146 135 -12.400 500 -13.796 2.514 0.556

YFL005W-YFL004W_151_285 151 135 -14.100 500 -14.561 2.571 0.179

YFL005W-YFL004W_156_290 156 135 -17.500 500 -16.522 2.558 -0.382

YFL005W-YFL004W_161_295 161 135 -17.500 500 -16.171 2.797 -0.475

YFL005W-YFL004W_166_300 166 135 -15.800 500 -18.087 2.846 0.804

YFL005W-YFL004W_171_305 171 135 -17.200 500 -18.096 2.945 0.304

YFL005W-YFL004W_176_310 176 135 -16.900 500 -18.346 2.858 0.506

YFL005W-YFL004W_181_315 181 135 -13.400 500 -16.407 2.770 1.085

YFL005W-YFL004W_186_320 186 135 -14.500 500 -17.494 2.785 1.075

YFL005W-YFL004W_191_325 191 135 -15.600 500 -17.876 2.760 0.825

YFL005W-YFL004W_196_330 196 135 -15.600 500 -19.364 2.891 1.302

YFL005W-YFL004W_201_335 201 135 -17.600 500 -21.507 3.097 1.262

YFL005W-YFL004W_206_340 206 135 -18.100 500 -22.943 3.214 1.507

YFL005W-YFL004W_211_345 211 135 -17.800 500 -24.736 3.283 2.112

YFL005W-YFL004W_216_350 216 135 -23.800 500 -25.790 3.433 0.580

YFL005W-YFL004W_221_355 221 135 -26.800 500 -27.145 3.339 0.103

YFL005W-YFL004W_226_360 226 135 -28.100 500 -27.477 3.524 -0.177

YFL005W-YFL004W_231_365 231 135 -29.000 500 -27.527 3.485 -0.423

YFL005W-YFL004W_236_370 236 135 -30.000 500 -26.893 3.396 -0.915

YFL005W-YFL004W_241_375 241 135 -28.100 500 -26.316 3.424 -0.521

YFL005W-YFL004W_246_380 246 135 -26.700 500 -26.190 3.257 -0.157

YFL005W-YFL004W_251_385 251 135 -24.500 500 -25.866 3.332 0.410

YFL005W-YFL004W_256_390 256 135 -28.000 500 -25.015 3.364 -0.887

YFL005W-YFL004W_261_395 261 135 -27.600 500 -23.771 3.291 -1.163

YFL005W-YFL004W_266_400 266 135 -25.900 500 -21.746 3.199 -1.298

YFL005W-YFL004W_271_405 271 135 -27.400 500 -21.979 2.919 -1.857

YFL005W-YFL004W_276_410 276 135 -28.900 500 -22.206 3.135 -2.135

YFL005W-YFL004W_281_415 281 135 -28.600 500 -21.826 3.147 -2.152

YFL005W-YFL004W_286_420 286 135 -31.700 500 -19.516 3.159 -3.857

YFL005W-YFL004W_291_425 291 135 -27.000 500 -21.733 3.229 -1.631

YFL005W-YFL004W_296_430 296 135 -28.600 500 -21.849 3.071 -2.198

YFL005W-YFL004W_301_435 301 135 -28.300 500 -22.198 3.010 -2.027

YFL005W-YFL004W_306_440 306 135 -25.700 500 -22.141 3.155 -1.128

YFL005W-YFL004W_311_445 311 135 -26.900 500 -22.527 3.293 -1.328

YFL005W-YFL004W_316_450 316 135 -27.900 500 -23.010 3.303 -1.481

YFL005W-YFL004W_321_455 321 135 -27.100 500 -21.266 2.952 -1.976

YFL005W-YFL004W_326_460 326 135 -24.700 500 -19.229 2.978 -1.837

YFL005W-YFL004W_331_465 331 135 -27.150 500 -19.874 3.051 -2.385

YFL005W-YFL004W_336_470 336 135 -23.850 500 -17.685 2.892 -2.131

YFL005W-YFL004W_341_475 341 135 -19.000 500 -18.691 2.729 -0.113

YFL005W-YFL004W_346_480 346 135 -22.450 500 -19.776 2.856 -0.936

YFL005W-YFL004W_351_485 351 135 -18.150 500 -17.887 2.868 -0.092

YFL005W-YFL004W_356_490 356 135 -20.800 500 -20.052 2.788 -0.268

YFL005W-YFL004W_361_495 361 135 -20.800 500 -20.481 2.859 -0.111

YFL005W-YFL004W_366_500 366 135 -18.300 500 -19.181 2.869 0.307

YFL005W-YFL004W_371_505 371 135 -18.300 500 -18.664 2.799 0.130

YFL005W-YFL004W_376_510 376 135 -18.700 500 -20.379 3.125 0.537

YFL005W-YFL004W_381_515 381 135 -16.500 500 -20.599 2.943 1.393

YFL005W-YFL004W_386_520 386 135 -15.600 500 -19.328 2.776 1.343

YFL005W-YFL004W_391_525 391 135 -15.600 500 -19.660 2.929 1.386

YFL005W-YFL004W_396_530 396 135 -17.800 500 -21.220 3.002 1.139

YFL005W-YFL004W_401_535 401 135 -20.400 500 -22.333 2.979 0.649

YFL005W-YFL004W_406_540 406 135 -20.500 500 -22.905 3.123 0.770

YFL005W-YFL004W_411_545 411 135 -20.400 500 -24.230 3.221 1.189

YFL005W-YFL004W_416_550 416 135 -23.900 500 -25.226 3.073 0.431

YFL005W-YFL004W_421_555 421 135 -22.000 500 -25.319 3.118 1.064

YFL005W-YFL004W_426_560 426 135 -22.000 500 -25.264 3.181 1.026

YFL005W-YFL004W_431_565 431 135 -23.300 500 -25.152 3.383 0.547

YFL005W-YFL004W_436_570 436 135 -23.400 500 -25.208 3.261 0.554

YFL005W-YFL004W_441_575 441 135 -23.400 500 -25.138 2.927 0.594

YFL005W-YFL004W_446_580 446 135 -24.500 500 -26.116 3.134 0.516

YFL005W-YFL004W_451_585 451 135 -25.000 500 -26.151 3.342 0.344

YFL005W-YFL004W_456_590 456 135 -25.600 500 -27.439 3.235 0.568

YFL005W-YFL004W_461_595 461 135 -26.400 500 -28.246 3.257 0.567

YFL005W-YFL004W_466_600 466 135 -31.300 500 -28.335 3.438 -0.863

YFL005W-YFL004W_471_605 471 135 -28.600 500 -27.839 3.417 -0.223

YFL005W-YFL004W_476_610 476 135 -33.300 500 -27.151 3.216 -1.912

YFL005W-YFL004W_481_615 481 135 -34.000 500 -28.537 3.151 -1.734

YFL005W-YFL004W_486_620 486 135 -32.700 500 -26.825 3.209 -1.830

YFL005W-YFL004W_491_625 491 135 -30.500 500 -25.260 3.243 -1.616

YFL005W-YFL004W_496_630 496 135 -32.800 500 -26.056 3.234 -2.085

YFL005W-YFL004W_501_635 501 135 -32.100 500 -26.814 3.488 -1.515

YFL005W-YFL004W_506_640 506 135 -31.900 500 -26.703 3.535 -1.470

YFL005W-YFL004W_511_645 511 135 -31.800 500 -27.528 3.476 -1.229

YFL005W-YFL004W_516_650 516 135 -33.400 500 -29.076 3.412 -1.267

YFL005W-YFL004W_521_655 521 135 -33.400 500 -28.695 3.483 -1.351

YFL005W-YFL004W_526_660 526 135 -33.400 500 -26.692 3.318 -2.022

YFL005W-YFL004W_531_665 531 135 -29.800 500 -24.613 3.285 -1.579

YFL005W-YFL004W_536_670 536 135 -26.300 500 -23.038 3.044 -1.072

YFL005W-YFL004W_541_675 541 135 -24.900 500 -23.451 3.039 -0.477

YFL005W-YFL004W_546_680 546 135 -25.800 500 -22.741 3.211 -0.953

YFL005W-YFL004W_551_685 551 135 -24.200 500 -22.313 3.392 -0.556

YFL005W-YFL004W_556_690 556 135 -26.800 500 -24.083 3.257 -0.834

YFL005W-YFL004W_561_695 561 135 -26.830 500 -24.374 3.278 -0.749

YFL005W-YFL004W_566_700 566 135 -26.630 500 -23.677 3.283 -0.899

YFL005W-YFL004W_571_705 571 135 -20.130 500 -22.150 2.890 0.699

YFL005W-YFL004W_576_710 576 135 -18.900 500 -20.848 2.941 0.662

YFL005W-YFL004W_581_715 581 135 -15.850 500 -18.828 2.893 1.029

YFL005W-YFL004W_586_720 586 135 -22.030 500 -22.195 2.939 0.056

YFL005W-YFL004W_591_725 591 135 -17.600 500 -22.366 3.328 1.432

YFL005W-YFL004W_596_730 596 135 -17.130 500 -21.785 3.191 1.459

YFL005W-YFL004W_601_735 601 135 -20.130 500 -23.245 3.114 1.000

YFL005W-YFL004W_606_740 606 135 -22.930 500 -24.587 3.125 0.530

YFL005W-YFL004W_611_745 611 135 -23.600 500 -26.194 3.163 0.820

YFL005W-YFL004W_616_750 616 135 -24.200 500 -24.968 3.208 0.239

YFL005W-YFL004W_621_755 621 135 -22.100 500 -25.910 3.261 1.168

YFL005W-YFL004W_626_760 626 135 -21.900 500 -25.612 3.142 1.182

YFL005W-YFL004W_631_765 631 135 -21.800 500 -25.370 3.261 1.095

YFL005W-YFL004W_636_770 636 135 -24.800 500 -25.955 3.363 0.343

YFL005W-YFL004W_641_775 641 135 -27.600 500 -26.490 3.737 -0.297

YFL005W-YFL004W_646_780 646 135 -28.000 500 -26.056 3.376 -0.576

YFL005W-YFL004W_651_785 651 135 -30.500 500 -24.881 3.217 -1.747

YFL005W-YFL004W_656_790 656 135 -28.800 500 -25.450 3.367 -0.995

YFL005W-YFL004W_661_795 661 135 -28.800 500 -23.684 3.346 -1.529

YFL005W-YFL004W_666_800 666 135 -26.100 500 -23.218 3.247 -0.888

YFL005W-YFL004W_671_805 671 135 -28.300 500 -23.858 3.164 -1.404

YFL005W-YFL004W_676_810 676 135 -28.300 500 -21.781 3.254 -2.003

YFL005W-YFL004W_681_815 681 135 -28.300 500 -22.605 3.208 -1.775

YFL005W-YFL004W_686_820 686 135 -27.200 500 -22.190 3.294 -1.521

YFL005W-YFL004W_691_825 691 135 -21.000 500 -20.967 3.110 -0.011

YFL005W-YFL004W_695_828 695 133 -19.200 500 -19.210 3.056 0.003

Window size = 140

YFL005W-YFL004W_1_140 1 140 -24.000 500 -21.285 3.007 -0.903

YFL005W-YFL004W_6_145 6 140 -23.600 500 -20.384 2.917 -1.102

YFL005W-YFL004W_11_150 11 140 -20.890 500 -17.259 2.800 -1.297

YFL005W-YFL004W_16_155 16 140 -20.790 500 -17.419 2.751 -1.225

YFL005W-YFL004W_21_160 21 140 -19.090 500 -16.754 2.739 -0.853

YFL005W-YFL004W_26_165 26 140 -14.650 500 -15.603 2.854 0.334

YFL005W-YFL004W_31_170 31 140 -14.300 500 -14.721 2.792 0.151

YFL005W-YFL004W_36_175 36 140 -13.500 500 -14.704 2.664 0.452

YFL005W-YFL004W_41_180 41 140 -12.490 500 -14.152 2.534 0.656

YFL005W-YFL004W_46_185 46 140 -10.900 500 -12.689 2.645 0.676

YFL005W-YFL004W_51_190 51 140 -9.200 500 -11.190 2.395 0.831

YFL005W-YFL004W_56_195 56 140 -8.700 500 -10.074 2.399 0.573

YFL005W-YFL004W_61_200 61 140 -8.300 500 -9.664 2.420 0.564

YFL005W-YFL004W_66_205 66 140 -9.000 500 -9.202 2.280 0.089

YFL005W-YFL004W_71_210 71 140 -9.890 500 -8.797 2.279 -0.480

YFL005W-YFL004W_76_215 76 140 -11.100 500 -9.524 2.325 -0.678

YFL005W-YFL004W_81_220 81 140 -11.100 500 -9.579 2.258 -0.674

YFL005W-YFL004W_86_225 86 140 -11.100 500 -10.377 2.429 -0.298

YFL005W-YFL004W_91_230 91 140 -10.700 500 -9.709 2.244 -0.442

YFL005W-YFL004W_96_235 96 140 -10.700 500 -9.820 2.474 -0.356

YFL005W-YFL004W_101_240 101 140 -8.800 500 -8.099 2.301 -0.305

YFL005W-YFL004W_106_245 106 140 -8.800 500 -8.070 2.340 -0.312

YFL005W-YFL004W_111_250 111 140 -10.700 500 -10.853 2.458 0.062

YFL005W-YFL004W_116_255 116 140 -10.700 500 -10.727 2.428 0.011

YFL005W-YFL004W_121_260 121 140 -10.700 500 -10.876 2.521 0.070

YFL005W-YFL004W_126_265 126 140 -14.600 500 -12.971 2.518 -0.647

YFL005W-YFL004W_131_270 131 140 -13.200 500 -13.280 2.462 0.032

YFL005W-YFL004W_136_275 136 140 -13.200 500 -13.425 2.534 0.089

YFL005W-YFL004W_141_280 141 140 -13.000 500 -15.675 2.774 0.964

YFL005W-YFL004W_146_285 146 140 -14.100 500 -14.917 2.695 0.303

YFL005W-YFL004W_151_290 151 140 -17.500 500 -16.545 2.660 -0.359

YFL005W-YFL004W_156_295 156 140 -18.500 500 -16.244 2.635 -0.856

YFL005W-YFL004W_161_300 161 140 -17.500 500 -18.441 2.903 0.324

YFL005W-YFL004W_166_305 166 140 -17.200 500 -19.457 2.674 0.844

YFL005W-YFL004W_171_310 171 140 -17.200 500 -18.457 2.845 0.442

YFL005W-YFL004W_176_315 176 140 -16.900 500 -18.165 3.028 0.418

YFL005W-YFL004W_181_320 181 140 -14.500 500 -18.418 2.865 1.367

YFL005W-YFL004W_186_325 186 140 -15.600 500 -18.561 2.822 1.049

YFL005W-YFL004W_191_330 191 140 -15.600 500 -19.602 3.057 1.309

YFL005W-YFL004W_196_335 196 140 -17.600 500 -21.499 3.236 1.205

YFL005W-YFL004W_201_340 201 140 -18.100 500 -23.120 3.232 1.553

YFL005W-YFL004W_206_345 206 140 -18.100 500 -24.576 3.248 1.994

YFL005W-YFL004W_211_350 211 140 -23.800 500 -26.419 3.458 0.757

YFL005W-YFL004W_216_355 216 140 -26.800 500 -27.904 3.498 0.315

YFL005W-YFL004W_221_360 221 140 -28.100 500 -28.127 3.302 0.008

YFL005W-YFL004W_226_365 226 140 -29.000 500 -28.389 3.370 -0.181

YFL005W-YFL004W_231_370 231 140 -31.500 500 -28.000 3.384 -1.034

YFL005W-YFL004W_236_375 236 140 -30.000 500 -27.339 3.339 -0.797

YFL005W-YFL004W_241_380 241 140 -29.700 500 -27.063 3.329 -0.792

YFL005W-YFL004W_246_385 246 140 -28.100 500 -26.932 3.522 -0.332

YFL005W-YFL004W_251_390 251 140 -28.000 500 -26.795 3.290 -0.366

YFL005W-YFL004W_256_395 256 140 -29.000 500 -25.887 3.203 -0.972

YFL005W-YFL004W_261_400 261 140 -28.100 500 -24.665 3.487 -0.985

YFL005W-YFL004W_266_405 266 140 -27.400 500 -22.636 3.366 -1.415

YFL005W-YFL004W_271_410 271 140 -28.900 500 -22.552 3.061 -2.074

YFL005W-YFL004W_276_415 276 140 -29.000 500 -23.259 2.970 -1.933

YFL005W-YFL004W_281_420 281 140 -35.900 500 -22.313 3.215 -4.225 ***

YFL005W-YFL004W_286_425 286 140 -31.700 500 -22.120 3.164 -3.028

YFL005W-YFL004W_291_430 291 140 -28.600 500 -22.185 3.133 -2.048

YFL005W-YFL004W_296_435 296 140 -28.600 500 -23.313 3.265 -1.619

YFL005W-YFL004W_301_440 301 140 -28.800 500 -23.246 3.181 -1.746

YFL005W-YFL004W_306_445 306 140 -28.700 500 -23.354 2.971 -1.799

YFL005W-YFL004W_311_450 311 140 -29.900 500 -23.521 3.218 -1.982

YFL005W-YFL004W_316_455 316 140 -27.900 500 -22.775 3.165 -1.620

YFL005W-YFL004W_321_460 321 140 -27.100 500 -22.004 3.066 -1.662

YFL005W-YFL004W_326_465 326 140 -27.450 500 -20.533 2.962 -2.335

YFL005W-YFL004W_331_470 331 140 -27.150 500 -21.055 3.176 -1.919

YFL005W-YFL004W_336_475 336 140 -25.250 500 -19.503 3.024 -1.900

YFL005W-YFL004W_341_480 341 140 -22.450 500 -20.264 2.884 -0.758

YFL005W-YFL004W_346_485 346 140 -23.150 500 -21.001 2.932 -0.733

YFL005W-YFL004W_351_490 351 140 -20.800 500 -20.693 3.009 -0.036

YFL005W-YFL004W_356_495 356 140 -22.500 500 -20.668 3.043 -0.602

YFL005W-YFL004W_361_500 361 140 -20.950 500 -20.714 3.082 -0.077

YFL005W-YFL004W_366_505 366 140 -18.300 500 -18.966 2.672 0.249

YFL005W-YFL004W_371_510 371 140 -18.700 500 -20.273 3.003 0.524

YFL005W-YFL004W_376_515 376 140 -18.700 500 -20.636 2.988 0.648

YFL005W-YFL004W_381_520 381 140 -16.500 500 -20.859 3.098 1.407

YFL005W-YFL004W_386_525 386 140 -19.900 500 -21.321 3.139 0.453

YFL005W-YFL004W_391_530 391 140 -17.800 500 -21.771 3.064 1.296

YFL005W-YFL004W_396_535 396 140 -20.400 500 -22.795 3.147 0.761

YFL005W-YFL004W_401_540 401 140 -20.600 500 -23.827 3.262 0.989

YFL005W-YFL004W_406_545 406 140 -20.500 500 -24.530 3.271 1.232

YFL005W-YFL004W_411_550 411 140 -23.900 500 -26.377 3.254 0.761

YFL005W-YFL004W_416_555 416 140 -24.500 500 -27.457 3.267 0.905

YFL005W-YFL004W_421_560 421 140 -23.600 500 -27.078 3.145 1.106

YFL005W-YFL004W_426_565 426 140 -23.300 500 -26.412 3.232 0.963

YFL005W-YFL004W_431_570 431 140 -23.400 500 -26.733 3.079 1.083

YFL005W-YFL004W_436_575 436 140 -24.100 500 -25.980 3.438 0.547

YFL005W-YFL004W_441_580 441 140 -24.500 500 -26.746 3.176 0.707

YFL005W-YFL004W_446_585 446 140 -25.000 500 -26.713 3.265 0.525

YFL005W-YFL004W_451_590 451 140 -26.600 500 -27.906 3.276 0.399

YFL005W-YFL004W_456_595 456 140 -26.400 500 -28.333 3.080 0.627

YFL005W-YFL004W_461_600 461 140 -31.400 500 -31.005 3.563 -0.111

YFL005W-YFL004W_466_605 466 140 -31.300 500 -29.017 3.202 -0.713

YFL005W-YFL004W_471_610 471 140 -33.300 500 -29.168 3.364 -1.228

YFL005W-YFL004W_476_615 476 140 -34.000 500 -29.479 3.287 -1.375

YFL005W-YFL004W_481_620 481 140 -34.400 500 -28.560 3.500 -1.669

YFL005W-YFL004W_486_625 486 140 -34.100 500 -27.387 3.379 -1.987

YFL005W-YFL004W_491_630 491 140 -32.800 500 -26.675 3.493 -1.754

YFL005W-YFL004W_496_635 496 140 -34.400 500 -27.330 3.354 -2.108

YFL005W-YFL004W_501_640 501 140 -32.200 500 -27.409 3.570 -1.342

YFL005W-YFL004W_506_645 506 140 -32.000 500 -27.851 3.479 -1.192

YFL005W-YFL004W_511_650 511 140 -33.400 500 -28.933 3.562 -1.254

YFL005W-YFL004W_516_655 516 140 -33.400 500 -29.366 3.388 -1.191

YFL005W-YFL004W_521_660 521 140 -33.400 500 -29.185 3.451 -1.221

YFL005W-YFL004W_526_665 526 140 -33.400 500 -27.156 3.404 -1.834

YFL005W-YFL004W_531_670 531 140 -29.800 500 -24.375 3.277 -1.655

YFL005W-YFL004W_536_675 536 140 -26.300 500 -24.095 3.248 -0.679

YFL005W-YFL004W_541_680 541 140 -25.900 500 -24.054 3.234 -0.571

YFL005W-YFL004W_546_685 546 140 -26.600 500 -25.419 3.334 -0.354

YFL005W-YFL004W_551_690 551 140 -28.480 500 -25.471 3.387 -0.889

YFL005W-YFL004W_556_695 556 140 -26.830 500 -24.773 3.251 -0.633

YFL005W-YFL004W_561_700 561 140 -26.930 500 -25.546 3.319 -0.417

YFL005W-YFL004W_566_705 566 140 -26.630 500 -23.779 3.193 -0.893

YFL005W-YFL004W_571_710 571 140 -20.130 500 -22.289 3.229 0.669

YFL005W-YFL004W_576_715 576 140 -19.030 500 -21.188 2.944 0.733

YFL005W-YFL004W_581_720 581 140 -22.130 500 -22.049 3.023 -0.027

YFL005W-YFL004W_586_725 586 140 -23.130 500 -23.533 3.195 0.126

YFL005W-YFL004W_591_730 591 140 -19.500 500 -24.556 3.091 1.636

YFL005W-YFL004W_596_735 596 140 -20.130 500 -25.158 3.180 1.581

YFL005W-YFL004W_601_740 601 140 -24.030 500 -25.991 3.038 0.645

YFL005W-YFL004W_606_745 606 140 -24.030 500 -26.361 3.158 0.738

YFL005W-YFL004W_611_750 611 140 -24.300 500 -26.609 3.372 0.685

YFL005W-YFL004W_616_755 616 140 -24.200 500 -26.246 3.374 0.607

YFL005W-YFL004W_621_760 621 140 -22.100 500 -26.062 3.268 1.212

YFL005W-YFL004W_626_765 626 140 -25.500 500 -27.820 3.493 0.664

YFL005W-YFL004W_631_770 631 140 -24.800 500 -26.002 3.330 0.361

YFL005W-YFL004W_636_775 636 140 -27.600 500 -27.260 3.370 -0.101

YFL005W-YFL004W_641_780 641 140 -28.000 500 -27.772 3.527 -0.065

YFL005W-YFL004W_646_785 646 140 -30.500 500 -26.062 3.344 -1.327

YFL005W-YFL004W_651_790 651 140 -31.300 500 -26.671 3.310 -1.399

YFL005W-YFL004W_656_795 656 140 -28.800 500 -25.455 3.234 -1.034

YFL005W-YFL004W_661_800 661 140 -28.800 500 -25.338 3.200 -1.082

YFL005W-YFL004W_666_805 666 140 -28.300 500 -24.330 3.261 -1.217

YFL005W-YFL004W_671_810 671 140 -28.300 500 -24.090 3.367 -1.250

YFL005W-YFL004W_676_815 676 140 -28.300 500 -21.558 3.015 -2.236

YFL005W-YFL004W_681_820 681 140 -28.300 500 -23.402 3.259 -1.503

YFL005W-YFL004W_686_825 686 140 -27.200 500 -23.009 3.131 -1.338

YFL005W-YFL004W_690_828 690 138 -22.100 500 -21.607 2.981 -0.165

Window size = 145

YFL005W-YFL004W_1_145 1 145 -25.700 500 -22.537 3.199 -0.989

YFL005W-YFL004W_6_150 6 145 -23.600 500 -20.221 2.819 -1.199

YFL005W-YFL004W_11_155 11 145 -20.890 500 -17.328 2.692 -1.323

YFL005W-YFL004W_16_160 16 145 -20.790 500 -17.456 2.759 -1.208

YFL005W-YFL004W_21_165 21 145 -19.090 500 -16.363 2.718 -1.003

YFL005W-YFL004W_26_170 26 145 -14.910 500 -16.330 2.859 0.497

YFL005W-YFL004W_31_175 31 145 -14.750 500 -15.633 2.635 0.335

YFL005W-YFL004W_36_180 36 145 -13.900 500 -15.923 2.633 0.768

YFL005W-YFL004W_41_185 41 145 -14.800 500 -15.999 2.745 0.437

YFL005W-YFL004W_46_190 46 145 -11.400 500 -12.482 2.465 0.439

YFL005W-YFL004W_51_195 51 145 -9.700 500 -11.576 2.541 0.739

YFL005W-YFL004W_56_200 56 145 -8.700 500 -10.425 2.519 0.685

YFL005W-YFL004W_61_205 61 145 -9.400 500 -10.280 2.504 0.351

YFL005W-YFL004W_66_210 66 145 -9.890 500 -9.467 2.302 -0.184

YFL005W-YFL004W_71_215 71 145 -11.100 500 -9.175 2.235 -0.861

YFL005W-YFL004W_76_220 76 145 -11.100 500 -9.600 2.370 -0.633

YFL005W-YFL004W_81_225 81 145 -11.100 500 -10.090 2.300 -0.439

YFL005W-YFL004W_86_230 86 145 -11.100 500 -10.100 2.399 -0.417

YFL005W-YFL004W_91_235 91 145 -11.900 500 -9.960 2.454 -0.791

YFL005W-YFL004W_96_240 96 145 -10.700 500 -10.387 2.251 -0.139

YFL005W-YFL004W_101_245 101 145 -8.800 500 -8.063 2.259 -0.326

YFL005W-YFL004W_106_250 106 145 -10.700 500 -10.339 2.426 -0.149

YFL005W-YFL004W_111_255 111 145 -10.700 500 -11.209 2.491 0.204

YFL005W-YFL004W_116_260 116 145 -10.700 500 -11.288 2.489 0.236

YFL005W-YFL004W_121_265 121 145 -14.600 500 -13.385 2.596 -0.468

YFL005W-YFL004W_126_270 126 145 -15.900 500 -14.910 2.626 -0.377

YFL005W-YFL004W_131_275 131 145 -13.200 500 -13.235 2.590 0.014

YFL005W-YFL004W_136_280 136 145 -14.000 500 -15.138 2.615 0.435

YFL005W-YFL004W_141_285 141 145 -15.200 500 -16.495 2.803 0.462

YFL005W-YFL004W_146_290 146 145 -17.500 500 -17.194 2.809 -0.109

YFL005W-YFL004W_151_295 151 145 -18.500 500 -16.602 2.668 -0.711

YFL005W-YFL004W_156_300 156 145 -18.500 500 -18.449 2.812 -0.018

YFL005W-YFL004W_161_305 161 145 -17.500 500 -19.822 2.960 0.785

YFL005W-YFL004W_166_310 166 145 -18.200 500 -19.857 2.908 0.570

YFL005W-YFL004W_171_315 171 145 -17.200 500 -18.441 2.798 0.443

YFL005W-YFL004W_176_320 176 145 -18.000 500 -20.247 2.970 0.756

YFL005W-YFL004W_181_325 181 145 -15.600 500 -19.676 2.928 1.392

YFL005W-YFL004W_186_330 186 145 -15.600 500 -19.871 2.816 1.517

YFL005W-YFL004W_191_335 191 145 -17.600 500 -21.526 2.893 1.357

YFL005W-YFL004W_196_340 196 145 -18.100 500 -23.018 3.179 1.547

YFL005W-YFL004W_201_345 201 145 -18.100 500 -24.664 3.126 2.100

YFL005W-YFL004W_206_350 206 145 -23.800 500 -26.655 3.310 0.862

YFL005W-YFL004W_211_355 211 145 -26.800 500 -28.956 3.390 0.636

YFL005W-YFL004W_216_360 216 145 -28.100 500 -28.726 3.452 0.181

YFL005W-YFL004W_221_365 221 145 -29.000 500 -29.130 3.528 0.037

YFL005W-YFL004W_226_370 226 145 -31.500 500 -28.558 3.467 -0.849

YFL005W-YFL004W_231_375 231 145 -31.500 500 -28.588 3.605 -0.808

YFL005W-YFL004W_236_380 236 145 -30.000 500 -28.256 3.540 -0.493

YFL005W-YFL004W_241_385 241 145 -30.900 500 -28.581 3.474 -0.668

YFL005W-YFL004W_246_390 246 145 -28.700 500 -27.896 3.248 -0.248

YFL005W-YFL004W_251_395 251 145 -30.000 500 -27.734 3.419 -0.663

YFL005W-YFL004W_256_400 256 145 -29.100 500 -26.708 3.316 -0.721

YFL005W-YFL004W_261_405 261 145 -28.200 500 -25.140 3.188 -0.960

YFL005W-YFL004W_266_410 266 145 -29.500 500 -23.197 3.032 -2.079

YFL005W-YFL004W_271_415 271 145 -29.100 500 -23.832 3.319 -1.587

YFL005W-YFL004W_276_420 276 145 -35.900 500 -24.751 3.606 -3.091

YFL005W-YFL004W_281_425 281 145 -38.100 500 -24.370 3.223 -4.260 ***

YFL005W-YFL004W_286_430 286 145 -31.700 500 -22.806 3.242 -2.744

YFL005W-YFL004W_291_435 291 145 -29.000 500 -23.605 3.307 -1.631

YFL005W-YFL004W_296_440 296 145 -29.100 500 -24.363 3.196 -1.482

YFL005W-YFL004W_301_445 301 145 -29.400 500 -24.472 3.267 -1.509

YFL005W-YFL004W_306_450 306 145 -29.900 500 -24.127 3.070 -1.881

YFL005W-YFL004W_311_455 311 145 -32.000 500 -23.483 2.970 -2.867

YFL005W-YFL004W_316_460 316 145 -27.900 500 -23.445 3.276 -1.360

YFL005W-YFL004W_321_465 321 145 -27.450 500 -23.633 3.101 -1.231

YFL005W-YFL004W_326_470 326 145 -27.450 500 -21.777 3.007 -1.887

YFL005W-YFL004W_331_475 331 145 -28.100 500 -22.708 3.057 -1.764

YFL005W-YFL004W_336_480 336 145 -25.650 500 -20.955 3.026 -1.551

YFL005W-YFL004W_341_485 341 145 -23.150 500 -21.137 2.882 -0.698

YFL005W-YFL004W_346_490 346 145 -23.350 500 -23.536 3.096 0.060

YFL005W-YFL004W_351_495 351 145 -22.500 500 -21.093 3.098 -0.454

YFL005W-YFL004W_356_500 356 145 -22.500 500 -20.744 2.923 -0.601

YFL005W-YFL004W_361_505 361 145 -20.950 500 -20.758 3.349 -0.057

YFL005W-YFL004W_366_510 366 145 -18.700 500 -20.723 2.947 0.686

YFL005W-YFL004W_371_515 371 145 -18.700 500 -21.029 2.951 0.789

YFL005W-YFL004W_376_520 376 145 -18.700 500 -21.315 3.133 0.835

YFL005W-YFL004W_381_525 381 145 -19.900 500 -22.644 3.074 0.893

YFL005W-YFL004W_386_530 386 145 -22.700 500 -23.176 3.241 0.147

YFL005W-YFL004W_391_535 391 145 -20.400 500 -23.505 3.059 1.015

YFL005W-YFL004W_396_540 396 145 -20.900 500 -24.053 2.994 1.053

YFL005W-YFL004W_401_545 401 145 -20.600 500 -25.806 3.181 1.637

YFL005W-YFL004W_406_550 406 145 -24.000 500 -26.827 3.402 0.831

YFL005W-YFL004W_411_555 411 145 -25.000 500 -27.851 3.296 0.865

YFL005W-YFL004W_416_560 416 145 -24.700 500 -28.789 3.274 1.249

YFL005W-YFL004W_421_565 421 145 -28.300 500 -28.145 3.265 -0.048

YFL005W-YFL004W_426_570 426 145 -24.400 500 -28.121 3.273 1.137

YFL005W-YFL004W_431_575 431 145 -24.400 500 -26.995 3.282 0.791

YFL005W-YFL004W_436_580 436 145 -25.500 500 -27.443 3.302 0.588

YFL005W-YFL004W_441_585 441 145 -25.500 500 -26.957 3.209 0.454

YFL005W-YFL004W_446_590 446 145 -26.900 500 -28.267 3.452 0.396

YFL005W-YFL004W_451_595 451 145 -27.400 500 -28.778 3.258 0.423

YFL005W-YFL004W_456_600 456 145 -31.400 500 -31.019 3.257 -0.117

YFL005W-YFL004W_461_605 461 145 -31.400 500 -31.641 3.569 0.067

YFL005W-YFL004W_466_610 466 145 -33.300 500 -30.465 3.526 -0.804

YFL005W-YFL004W_471_615 471 145 -34.000 500 -31.479 3.596 -0.701

YFL005W-YFL004W_476_620 476 145 -34.600 500 -29.396 3.558 -1.462

YFL005W-YFL004W_481_625 481 145 -34.700 500 -29.538 3.348 -1.542

YFL005W-YFL004W_486_630 486 145 -34.600 500 -28.749 3.489 -1.677

YFL005W-YFL004W_491_635 491 145 -34.500 500 -27.499 3.328 -2.104

YFL005W-YFL004W_496_640 496 145 -34.400 500 -28.013 3.393 -1.883

YFL005W-YFL004W_501_645 501 145 -32.300 500 -28.322 3.398 -1.171

YFL005W-YFL004W_506_650 506 145 -33.400 500 -29.110 3.484 -1.231

YFL005W-YFL004W_511_655 511 145 -33.400 500 -29.184 3.397 -1.241

YFL005W-YFL004W_516_660 516 145 -33.400 500 -29.614 3.361 -1.126

YFL005W-YFL004W_521_665 521 145 -33.400 500 -29.474 3.216 -1.221

YFL005W-YFL004W_526_670 526 145 -33.400 500 -27.133 3.314 -1.891

YFL005W-YFL004W_531_675 531 145 -29.800 500 -25.327 3.324 -1.346

YFL005W-YFL004W_536_680 536 145 -26.300 500 -24.628 3.205 -0.522

YFL005W-YFL004W_541_685 541 145 -26.600 500 -26.699 3.205 0.031

YFL005W-YFL004W_546_690 546 145 -29.100 500 -28.492 3.551 -0.171

YFL005W-YFL004W_551_695 551 145 -28.580 500 -26.119 3.229 -0.762

YFL005W-YFL004W_556_700 556 145 -26.930 500 -25.643 3.209 -0.401

YFL005W-YFL004W_561_705 561 145 -26.930 500 -25.174 3.006 -0.584

YFL005W-YFL004W_566_710 566 145 -26.630 500 -23.901 3.204 -0.852

YFL005W-YFL004W_571_715 571 145 -20.430 500 -22.798 3.286 0.721

YFL005W-YFL004W_576_720 576 145 -22.130 500 -24.930 3.049 0.918

YFL005W-YFL004W_581_725 581 145 -27.830 500 -23.667 3.007 -1.385

YFL005W-YFL004W_586_730 586 145 -23.130 500 -25.591 3.210 0.767

YFL005W-YFL004W_591_735 591 145 -23.930 500 -27.631 3.177 1.165

YFL005W-YFL004W_596_740 596 145 -24.030 500 -27.505 3.298 1.054

YFL005W-YFL004W_601_745 601 145 -24.030 500 -27.580 3.286 1.080

YFL005W-YFL004W_606_750 606 145 -24.300 500 -27.030 3.397 0.804

YFL005W-YFL004W_611_755 611 145 -25.500 500 -27.977 3.398 0.729

YFL005W-YFL004W_616_760 616 145 -24.200 500 -26.464 3.321 0.682

YFL005W-YFL004W_621_765 621 145 -25.500 500 -28.112 3.316 0.788

YFL005W-YFL004W_626_770 626 145 -25.900 500 -28.480 3.335 0.774

YFL005W-YFL004W_631_775 631 145 -27.600 500 -27.589 3.468 -0.003

YFL005W-YFL004W_636_780 636 145 -29.400 500 -28.876 3.470 -0.151

YFL005W-YFL004W_641_785 641 145 -30.500 500 -27.411 3.561 -0.868

YFL005W-YFL004W_646_790 646 145 -31.600 500 -27.661 3.537 -1.114

YFL005W-YFL004W_651_795 651 145 -31.300 500 -26.574 3.275 -1.443

YFL005W-YFL004W_656_800 656 145 -28.800 500 -26.672 3.201 -0.665

YFL005W-YFL004W_661_805 661 145 -29.200 500 -26.030 3.491 -0.908

YFL005W-YFL004W_666_810 666 145 -28.300 500 -24.629 3.119 -1.177

YFL005W-YFL004W_671_815 671 145 -28.300 500 -23.837 3.137 -1.423

YFL005W-YFL004W_676_820 676 145 -28.300 500 -22.736 3.073 -1.811

YFL005W-YFL004W_681_825 681 145 -28.300 500 -24.212 3.128 -1.307

YFL005W-YFL004W_685_828 685 143 -27.200 500 -23.900 3.367 -0.980

Window size = 150

YFL005W-YFL004W_1_150 1 150 -25.700 500 -22.064 3.127 -1.163

YFL005W-YFL004W_6_155 6 150 -23.600 500 -20.439 3.183 -0.993

YFL005W-YFL004W_11_160 11 150 -20.890 500 -17.549 2.918 -1.145

YFL005W-YFL004W_16_165 16 150 -20.790 500 -17.593 2.810 -1.138

YFL005W-YFL004W_21_170 21 150 -19.090 500 -17.096 2.777 -0.718

YFL005W-YFL004W_26_175 26 150 -16.250 500 -17.121 2.908 0.299

YFL005W-YFL004W_31_180 31 150 -14.750 500 -16.866 2.859 0.740

YFL005W-YFL004W_36_185 36 150 -16.100 500 -17.531 2.957 0.484

YFL005W-YFL004W_41_190 41 150 -15.300 500 -15.775 2.695 0.176

YFL005W-YFL004W_46_195 46 150 -11.400 500 -13.347 2.678 0.727

YFL005W-YFL004W_51_200 51 150 -9.700 500 -12.116 2.613 0.925

YFL005W-YFL004W_56_205 56 150 -9.500 500 -11.104 2.500 0.642

YFL005W-YFL004W_61_210 61 150 -10.290 500 -10.582 2.454 0.119

YFL005W-YFL004W_66_215 66 150 -11.100 500 -10.033 2.543 -0.419

YFL005W-YFL004W_71_220 71 150 -11.100 500 -9.304 2.362 -0.761

YFL005W-YFL004W_76_225 76 150 -11.100 500 -10.005 2.523 -0.434

YFL005W-YFL004W_81_230 81 150 -11.100 500 -9.849 2.286 -0.547

YFL005W-YFL004W_86_235 86 150 -11.900 500 -10.410 2.453 -0.607

YFL005W-YFL004W_91_240 91 150 -11.900 500 -10.682 2.424 -0.502

YFL005W-YFL004W_96_245 96 150 -10.700 500 -10.601 2.372 -0.042

YFL005W-YFL004W_101_250 101 150 -10.700 500 -10.212 2.418 -0.202

YFL005W-YFL004W_106_255 106 150 -10.700 500 -10.547 2.438 -0.063

YFL005W-YFL004W_111_260 111 150 -10.700 500 -11.594 2.486 0.360

YFL005W-YFL004W_116_265 116 150 -14.600 500 -14.314 2.605 -0.110

YFL005W-YFL004W_121_270 121 150 -15.900 500 -15.878 2.825 -0.008

YFL005W-YFL004W_126_275 126 150 -15.900 500 -15.286 2.747 -0.224

YFL005W-YFL004W_131_280 131 150 -14.000 500 -15.673 2.827 0.592

YFL005W-YFL004W_136_285 136 150 -15.200 500 -16.217 2.665 0.382

YFL005W-YFL004W_141_290 141 150 -18.000 500 -18.842 2.824 0.298

YFL005W-YFL004W_146_295 146 150 -18.500 500 -17.124 2.839 -0.485

YFL005W-YFL004W_151_300 151 150 -19.200 500 -18.685 2.942 -0.175

YFL005W-YFL004W_156_305 156 150 -18.500 500 -20.159 2.793 0.594

YFL005W-YFL004W_161_310 161 150 -18.200 500 -20.265 2.960 0.698

YFL005W-YFL004W_166_315 166 150 -18.200 500 -19.960 2.977 0.591

YFL005W-YFL004W_171_320 171 150 -18.300 500 -20.334 2.855 0.713

YFL005W-YFL004W_176_325 176 150 -19.100 500 -21.399 3.006 0.765

YFL005W-YFL004W_181_330 181 150 -15.600 500 -21.335 3.063 1.872

YFL005W-YFL004W_186_335 186 150 -17.600 500 -22.412 3.129 1.538

YFL005W-YFL004W_191_340 191 150 -18.100 500 -23.226 3.176 1.614

YFL005W-YFL004W_196_345 196 150 -18.100 500 -24.531 3.050 2.109

YFL005W-YFL004W_201_350 201 150 -23.800 500 -26.538 3.151 0.869

YFL005W-YFL004W_206_355 206 150 -26.800 500 -29.057 3.463 0.652

YFL005W-YFL004W_211_360 211 150 -28.100 500 -29.450 3.270 0.413

YFL005W-YFL004W_216_365 216 150 -29.700 500 -29.458 3.432 -0.071

YFL005W-YFL004W_221_370 221 150 -31.500 500 -29.397 3.459 -0.608

YFL005W-YFL004W_226_375 226 150 -31.500 500 -28.987 3.263 -0.770

YFL005W-YFL004W_231_380 231 150 -31.500 500 -28.984 3.419 -0.736

YFL005W-YFL004W_236_385 236 150 -31.200 500 -29.224 3.443 -0.574

YFL005W-YFL004W_241_390 241 150 -31.500 500 -29.183 3.433 -0.675

YFL005W-YFL004W_246_395 246 150 -30.900 500 -29.075 3.421 -0.533

YFL005W-YFL004W_251_400 251 150 -30.100 500 -28.288 3.430 -0.528

YFL005W-YFL004W_256_405 256 150 -29.700 500 -27.523 3.278 -0.664

YFL005W-YFL004W_261_410 261 150 -31.200 500 -26.371 3.397 -1.422

YFL005W-YFL004W_266_415 266 150 -29.500 500 -24.348 3.110 -1.657

YFL005W-YFL004W_271_420 271 150 -35.900 500 -24.739 3.465 -3.221

YFL005W-YFL004W_276_425 276 150 -38.800 500 -26.387 3.349 -3.707

YFL005W-YFL004W_281_430 281 150 -38.100 500 -24.914 3.380 -3.901

YFL005W-YFL004W_286_435 286 150 -31.700 500 -23.673 3.085 -2.602

YFL005W-YFL004W_291_440 291 150 -29.400 500 -24.584 3.278 -1.469

YFL005W-YFL004W_296_445 296 150 -29.800 500 -25.723 3.499 -1.165

YFL005W-YFL004W_301_450 301 150 -30.900 500 -25.257 3.263 -1.729

YFL005W-YFL004W_306_455 306 150 -32.000 500 -23.942 3.091 -2.607

YFL005W-YFL004W_311_460 311 150 -32.000 500 -24.133 3.050 -2.579

YFL005W-YFL004W_316_465 316 150 -32.100 500 -25.024 3.363 -2.104

YFL005W-YFL004W_321_470 321 150 -27.450 500 -24.454 2.993 -1.001

YFL005W-YFL004W_326_475 326 150 -28.400 500 -23.146 3.198 -1.643

YFL005W-YFL004W_331_480 331 150 -28.100 500 -24.119 3.090 -1.288

YFL005W-YFL004W_336_485 336 150 -25.650 500 -22.001 3.173 -1.150

YFL005W-YFL004W_341_490 341 150 -24.550 500 -24.081 3.119 -0.150

YFL005W-YFL004W_346_495 346 150 -23.750 500 -24.218 3.155 0.148

YFL005W-YFL004W_351_500 351 150 -22.500 500 -21.241 3.095 -0.407

YFL005W-YFL004W_356_505 356 150 -22.500 500 -20.589 2.847 -0.671

YFL005W-YFL004W_361_510 361 150 -20.950 500 -21.923 2.922 0.333

YFL005W-YFL004W_366_515 366 150 -18.700 500 -21.434 3.047 0.897

YFL005W-YFL004W_371_520 371 150 -18.700 500 -21.147 3.040 0.805

YFL005W-YFL004W_376_525 376 150 -20.000 500 -23.136 3.422 0.917

YFL005W-YFL004W_381_530 381 150 -22.700 500 -24.709 3.167 0.635

YFL005W-YFL004W_386_535 386 150 -22.700 500 -25.278 3.234 0.797

YFL005W-YFL004W_391_540 391 150 -21.600 500 -24.912 3.314 0.999

YFL005W-YFL004W_396_545 396 150 -20.900 500 -25.861 3.318 1.495

YFL005W-YFL004W_401_550 401 150 -24.000 500 -27.701 3.232 1.145

YFL005W-YFL004W_406_555 406 150 -25.000 500 -28.424 3.362 1.018

YFL005W-YFL004W_411_560 411 150 -26.200 500 -29.984 3.619 1.046

YFL005W-YFL004W_416_565 416 150 -29.000 500 -30.215 3.429 0.354

YFL005W-YFL004W_421_570 421 150 -28.300 500 -29.532 3.290 0.374

YFL005W-YFL004W_426_575 426 150 -26.500 500 -28.853 3.312 0.710

YFL005W-YFL004W_431_580 431 150 -25.800 500 -28.611 3.179 0.884

YFL005W-YFL004W_436_585 436 150 -26.900 500 -27.324 3.343 0.127

YFL005W-YFL004W_441_590 441 150 -26.900 500 -28.509 3.359 0.479

YFL005W-YFL004W_446_595 446 150 -28.900 500 -29.241 3.297 0.103

YFL005W-YFL004W_451_600 451 150 -31.800 500 -31.929 3.741 0.035

YFL005W-YFL004W_456_605 456 150 -31.600 500 -32.093 3.552 0.139

YFL005W-YFL004W_461_610 461 150 -34.500 500 -33.410 3.548 -0.307

YFL005W-YFL004W_466_615 466 150 -34.000 500 -32.333 3.430 -0.486

YFL005W-YFL004W_471_620 471 150 -38.500 500 -31.493 3.651 -1.919

YFL005W-YFL004W_476_625 476 150 -34.700 500 -30.417 3.202 -1.337

YFL005W-YFL004W_481_630 481 150 -35.900 500 -30.434 3.335 -1.639

YFL005W-YFL004W_486_635 486 150 -34.600 500 -29.720 3.360 -1.452

YFL005W-YFL004W_491_640 491 150 -34.600 500 -28.566 3.485 -1.731

YFL005W-YFL004W_496_645 496 150 -34.400 500 -29.109 3.560 -1.486

YFL005W-YFL004W_501_650 501 150 -33.400 500 -30.143 3.541 -0.920

YFL005W-YFL004W_506_655 506 150 -33.400 500 -29.235 3.299 -1.263

YFL005W-YFL004W_511_660 511 150 -33.400 500 -29.649 3.361 -1.116

YFL005W-YFL004W_516_665 516 150 -33.400 500 -29.771 3.269 -1.110

YFL005W-YFL004W_521_670 521 150 -33.400 500 -29.265 3.017 -1.371

YFL005W-YFL004W_526_675 526 150 -33.400 500 -27.745 3.580 -1.579

YFL005W-YFL004W_531_680 531 150 -29.800 500 -25.947 3.406 -1.131

YFL005W-YFL004W_536_685 536 150 -28.300 500 -27.598 3.530 -0.199

YFL005W-YFL004W_541_690 541 150 -32.200 500 -29.602 3.214 -0.808

YFL005W-YFL004W_546_695 546 150 -29.130 500 -29.151 3.393 0.006

YFL005W-YFL004W_551_700 551 150 -28.580 500 -26.824 3.268 -0.537

YFL005W-YFL004W_556_705 556 150 -26.930 500 -25.606 3.207 -0.413

YFL005W-YFL004W_561_710 561 150 -26.930 500 -25.730 3.080 -0.390

YFL005W-YFL004W_566_715 566 150 -27.500 500 -24.410 3.131 -0.987

YFL005W-YFL004W_571_720 571 150 -22.130 500 -26.408 3.278 1.305

YFL005W-YFL004W_576_725 576 150 -27.830 500 -26.323 3.440 -0.438

YFL005W-YFL004W_581_730 581 150 -27.830 500 -25.438 3.365 -0.711

YFL005W-YFL004W_586_735 586 150 -24.950 500 -28.503 3.325 1.068

YFL005W-YFL004W_591_740 591 150 -26.300 500 -30.288 3.429 1.163

YFL005W-YFL004W_596_745 596 150 -24.130 500 -29.867 3.506 1.636

YFL005W-YFL004W_601_750 601 150 -24.300 500 -28.345 3.333 1.214

YFL005W-YFL004W_606_755 606 150 -25.500 500 -28.931 3.359 1.021

YFL005W-YFL004W_611_760 611 150 -26.300 500 -27.743 3.056 0.472

YFL005W-YFL004W_616_765 616 150 -25.500 500 -28.561 3.466 0.883

YFL005W-YFL004W_621_770 621 150 -26.100 500 -28.815 3.253 0.835

YFL005W-YFL004W_626_775 626 150 -28.700 500 -29.724 3.415 0.300

YFL005W-YFL004W_631_780 631 150 -29.700 500 -29.106 3.606 -0.165

YFL005W-YFL004W_636_785 636 150 -30.500 500 -28.631 3.539 -0.528

YFL005W-YFL004W_641_790 641 150 -31.600 500 -29.770 3.508 -0.522

YFL005W-YFL004W_646_795 646 150 -31.600 500 -27.783 3.539 -1.079

YFL005W-YFL004W_651_800 651 150 -31.300 500 -28.307 3.348 -0.894

YFL005W-YFL004W_656_805 656 150 -29.900 500 -27.524 3.394 -0.700

YFL005W-YFL004W_661_810 661 150 -29.200 500 -26.387 3.402 -0.827

YFL005W-YFL004W_666_815 666 150 -30.200 500 -24.765 3.389 -1.604

YFL005W-YFL004W_671_820 671 150 -28.300 500 -24.700 3.267 -1.102

YFL005W-YFL004W_676_825 676 150 -31.100 500 -23.839 3.285 -2.211

YFL005W-YFL004W_680_828 680 148 -28.300 500 -25.319 3.361 -0.887

Window size = 155

YFL005W-YFL004W_1_155 1 155 -25.700 500 -22.459 3.060 -1.059

YFL005W-YFL004W_6_160 6 155 -23.600 500 -20.329 2.717 -1.204

YFL005W-YFL004W_11_165 11 155 -20.890 500 -17.069 2.641 -1.447

YFL005W-YFL004W_16_170 16 155 -20.790 500 -17.939 2.745 -1.039

YFL005W-YFL004W_21_175 21 155 -19.800 500 -18.388 2.962 -0.477

YFL005W-YFL004W_26_180 26 155 -16.250 500 -18.743 2.930 0.851

YFL005W-YFL004W_31_185 31 155 -16.650 500 -18.262 2.946 0.547

YFL005W-YFL004W_36_190 36 155 -17.200 500 -17.564 2.738 0.133

YFL005W-YFL004W_41_195 41 155 -15.300 500 -16.289 2.768 0.357

YFL005W-YFL004W_46_200 46 155 -12.400 500 -13.440 2.692 0.386

YFL005W-YFL004W_51_205 51 155 -10.000 500 -12.266 2.490 0.910

YFL005W-YFL004W_56_210 56 155 -10.290 500 -11.082 2.540 0.312

YFL005W-YFL004W_61_215 61 155 -11.100 500 -10.993 2.487 -0.043

YFL005W-YFL004W_66_220 66 155 -11.100 500 -9.891 2.223 -0.544

YFL005W-YFL004W_71_225 71 155 -11.100 500 -9.483 2.275 -0.711

YFL005W-YFL004W_76_230 76 155 -11.100 500 -9.688 2.440 -0.579

YFL005W-YFL004W_81_235 81 155 -11.900 500 -10.103 2.454 -0.733

YFL005W-YFL004W_86_240 86 155 -11.900 500 -10.989 2.561 -0.356

YFL005W-YFL004W_91_245 91 155 -11.900 500 -10.632 2.322 -0.546

YFL005W-YFL004W_96_250 96 155 -12.600 500 -12.677 2.646 0.029

YFL005W-YFL004W_101_255 101 155 -10.700 500 -10.738 2.531 0.015

YFL005W-YFL004W_106_260 106 155 -10.700 500 -11.036 2.493 0.135

YFL005W-YFL004W_111_265 111 155 -14.600 500 -14.751 2.679 0.056

YFL005W-YFL004W_116_270 116 155 -16.100 500 -16.534 2.779 0.156

YFL005W-YFL004W_121_275 121 155 -15.900 500 -16.031 2.786 0.047

YFL005W-YFL004W_126_280 126 155 -15.900 500 -17.062 2.653 0.438

YFL005W-YFL004W_131_285 131 155 -15.200 500 -16.354 2.667 0.433

YFL005W-YFL004W_136_290 136 155 -18.000 500 -18.507 2.812 0.180

YFL005W-YFL004W_141_295 141 155 -18.500 500 -18.786 2.905 0.099

YFL005W-YFL004W_146_300 146 155 -19.200 500 -19.068 2.898 -0.045

YFL005W-YFL004W_151_305 151 155 -20.500 500 -20.616 3.021 0.038

YFL005W-YFL004W_156_310 156 155 -18.500 500 -20.452 2.959 0.660

YFL005W-YFL004W_161_315 161 155 -18.200 500 -20.093 3.005 0.630

YFL005W-YFL004W_166_320 166 155 -18.300 500 -21.819 3.020 1.165

YFL005W-YFL004W_171_325 171 155 -19.400 500 -21.542 2.930 0.731

YFL005W-YFL004W_176_330 176 155 -19.100 500 -22.812 3.269 1.136

YFL005W-YFL004W_181_335 181 155 -17.600 500 -23.728 3.298 1.858

YFL005W-YFL004W_186_340 186 155 -18.100 500 -23.791 3.256 1.748

YFL005W-YFL004W_191_345 191 155 -18.100 500 -24.785 3.286 2.034

YFL005W-YFL004W_196_350 196 155 -23.800 500 -26.859 3.503 0.873

YFL005W-YFL004W_201_355 201 155 -26.800 500 -29.051 3.338 0.674

YFL005W-YFL004W_206_360 206 155 -28.100 500 -29.586 3.300 0.450

YFL005W-YFL004W_211_365 211 155 -29.700 500 -30.302 3.441 0.175

YFL005W-YFL004W_216_370 216 155 -31.500 500 -29.607 3.294 -0.575

YFL005W-YFL004W_221_375 221 155 -31.500 500 -29.567 3.692 -0.523

YFL005W-YFL004W_226_380 226 155 -31.500 500 -29.705 3.514 -0.511

YFL005W-YFL004W_231_385 231 155 -31.500 500 -30.088 3.576 -0.395

YFL005W-YFL004W_236_390 236 155 -31.900 500 -30.030 3.549 -0.527

YFL005W-YFL004W_241_395 241 155 -31.500 500 -30.562 3.655 -0.257

YFL005W-YFL004W_246_400 246 155 -31.000 500 -29.566 3.486 -0.411

YFL005W-YFL004W_251_405 251 155 -30.100 500 -28.820 3.351 -0.382

YFL005W-YFL004W_256_410 256 155 -32.700 500 -28.053 3.413 -1.362

YFL005W-YFL004W_261_415 261 155 -31.200 500 -27.258 3.325 -1.185

YFL005W-YFL004W_266_420 266 155 -35.900 500 -25.074 3.262 -3.319

YFL005W-YFL004W_271_425 271 155 -38.800 500 -26.939 3.192 -3.716

YFL005W-YFL004W_276_430 276 155 -39.500 500 -27.126 3.462 -3.574

YFL005W-YFL004W_281_435 281 155 -38.100 500 -26.294 3.349 -3.525

YFL005W-YFL004W_286_440 286 155 -33.500 500 -25.049 3.398 -2.487

YFL005W-YFL004W_291_445 291 155 -34.700 500 -25.911 3.045 -2.887

YFL005W-YFL004W_296_450 296 155 -31.200 500 -26.535 3.249 -1.436

YFL005W-YFL004W_301_455 301 155 -32.000 500 -25.458 3.155 -2.074

YFL005W-YFL004W_306_460 306 155 -32.000 500 -25.014 3.362 -2.078

YFL005W-YFL004W_311_465 311 155 -32.300 500 -25.889 3.444 -1.862

YFL005W-YFL004W_316_470 316 155 -33.300 500 -25.776 3.119 -2.412

YFL005W-YFL004W_321_475 321 155 -31.300 500 -25.975 3.242 -1.643

YFL005W-YFL004W_326_480 326 155 -28.400 500 -24.948 3.188 -1.083

YFL005W-YFL004W_331_485 331 155 -28.700 500 -25.387 3.143 -1.054

YFL005W-YFL004W_336_490 336 155 -29.700 500 -24.916 3.132 -1.528

YFL005W-YFL004W_341_495 341 155 -26.250 500 -24.456 3.144 -0.571

YFL005W-YFL004W_346_500 346 155 -23.950 500 -24.569 3.341 0.185

YFL005W-YFL004W_351_505 351 155 -22.700 500 -21.634 3.282 -0.325

YFL005W-YFL004W_356_510 356 155 -22.500 500 -22.421 3.120 -0.025

YFL005W-YFL004W_361_515 361 155 -20.950 500 -22.377 2.998 0.476

YFL005W-YFL004W_366_520 366 155 -18.700 500 -21.354 3.006 0.883

YFL005W-YFL004W_371_525 371 155 -20.000 500 -23.054 3.192 0.957

YFL005W-YFL004W_376_530 376 155 -22.700 500 -24.896 3.123 0.703

YFL005W-YFL004W_381_535 381 155 -23.300 500 -26.298 3.238 0.926

YFL005W-YFL004W_386_540 386 155 -22.700 500 -26.169 3.343 1.038

YFL005W-YFL004W_391_545 391 155 -21.600 500 -26.584 3.355 1.485

YFL005W-YFL004W_396_550 396 155 -24.300 500 -27.792 3.078 1.135

YFL005W-YFL004W_401_555 401 155 -25.000 500 -29.340 3.438 1.262

YFL005W-YFL004W_406_560 406 155 -26.900 500 -30.505 3.503 1.029

YFL005W-YFL004W_411_565 411 155 -29.000 500 -30.797 3.370 0.533

YFL005W-YFL004W_416_570 416 155 -29.000 500 -31.301 3.452 0.666

YFL005W-YFL004W_421_575 421 155 -28.300 500 -30.386 3.377 0.618

YFL005W-YFL004W_426_580 426 155 -26.500 500 -30.308 3.497 1.089

YFL005W-YFL004W_431_585 431 155 -28.000 500 -29.085 3.341 0.325

YFL005W-YFL004W_436_590 436 155 -27.600 500 -29.321 3.424 0.503

YFL005W-YFL004W_441_595 441 155 -28.900 500 -29.599 3.421 0.204

YFL005W-YFL004W_446_600 446 155 -33.100 500 -32.540 3.520 -0.159

YFL005W-YFL004W_451_605 451 155 -32.900 500 -32.542 3.416 -0.105

YFL005W-YFL004W_456_610 456 155 -37.500 500 -33.617 3.648 -1.064

YFL005W-YFL004W_461_615 461 155 -34.600 500 -35.381 3.496 0.223

YFL005W-YFL004W_466_620 466 155 -40.700 500 -33.041 3.419 -2.240

YFL005W-YFL004W_471_625 471 155 -38.500 500 -32.775 3.383 -1.692

YFL005W-YFL004W_476_630 476 155 -35.900 500 -31.879 3.663 -1.098

YFL005W-YFL004W_481_635 481 155 -35.900 500 -32.001 3.541 -1.101

YFL005W-YFL004W_486_640 486 155 -35.400 500 -30.499 3.529 -1.389

YFL005W-YFL004W_491_645 491 155 -34.600 500 -29.261 3.438 -1.553

YFL005W-YFL004W_496_650 496 155 -34.400 500 -30.426 3.386 -1.174

YFL005W-YFL004W_501_655 501 155 -33.400 500 -30.211 3.265 -0.977

YFL005W-YFL004W_506_660 506 155 -36.500 500 -29.881 3.367 -1.966

YFL005W-YFL004W_511_665 511 155 -33.400 500 -30.017 3.472 -0.974

YFL005W-YFL004W_516_670 516 155 -33.400 500 -29.585 3.251 -1.174

YFL005W-YFL004W_521_675 521 155 -33.400 500 -29.949 3.377 -1.022

YFL005W-YFL004W_526_680 526 155 -33.880 500 -28.890 3.400 -1.468

YFL005W-YFL004W_531_685 531 155 -31.800 500 -28.740 3.505 -0.873

YFL005W-YFL004W_536_690 536 155 -32.580 500 -30.668 3.488 -0.548

YFL005W-YFL004W_541_695 541 155 -32.200 500 -30.416 3.395 -0.526

YFL005W-YFL004W_546_700 546 155 -31.580 500 -29.934 3.505 -0.470

YFL005W-YFL004W_551_705 551 155 -28.580 500 -27.047 3.401 -0.451

YFL005W-YFL004W_556_710 556 155 -26.930 500 -25.856 3.220 -0.334

YFL005W-YFL004W_561_715 561 155 -27.800 500 -26.506 3.522 -0.367

YFL005W-YFL004W_566_720 566 155 -29.400 500 -27.992 3.432 -0.410

YFL005W-YFL004W_571_725 571 155 -27.830 500 -27.294 3.342 -0.161

YFL005W-YFL004W_576_730 576 155 -27.830 500 -28.123 3.246 0.090

YFL005W-YFL004W_581_735 581 155 -27.830 500 -28.854 3.222 0.318

YFL005W-YFL004W_586_740 586 155 -27.000 500 -31.296 3.331 1.289

YFL005W-YFL004W_591_745 591 155 -29.100 500 -32.249 3.598 0.875

YFL005W-YFL004W_596_750 596 155 -26.530 500 -29.727 3.297 0.970

YFL005W-YFL004W_601_755 601 155 -25.500 500 -29.683 3.325 1.258

YFL005W-YFL004W_606_760 606 155 -26.400 500 -28.788 3.403 0.702

YFL005W-YFL004W_611_765 611 155 -26.900 500 -30.465 3.381 1.054

YFL005W-YFL004W_616_770 616 155 -26.100 500 -29.658 3.430 1.037

YFL005W-YFL004W_621_775 621 155 -30.000 500 -29.993 3.648 -0.002

YFL005W-YFL004W_626_780 626 155 -29.700 500 -31.617 3.442 0.557

YFL005W-YFL004W_631_785 631 155 -30.500 500 -29.211 3.402 -0.379

YFL005W-YFL004W_636_790 636 155 -31.700 500 -30.915 3.511 -0.223

YFL005W-YFL004W_641_795 641 155 -35.000 500 -29.777 3.741 -1.396

YFL005W-YFL004W_646_800 646 155 -31.600 500 -29.168 3.271 -0.744

YFL005W-YFL004W_651_805 651 155 -32.700 500 -28.944 3.524 -1.066

YFL005W-YFL004W_656_810 656 155 -29.900 500 -27.413 3.544 -0.702

YFL005W-YFL004W_661_815 661 155 -30.200 500 -26.251 3.323 -1.188

YFL005W-YFL004W_666_820 666 155 -30.200 500 -25.328 3.212 -1.517

YFL005W-YFL004W_671_825 671 155 -31.100 500 -25.847 3.454 -1.521

YFL005W-YFL004W_675_828 675 153 -34.500 500 -24.857 3.317 -2.907

Window size = 160

YFL005W-YFL004W_1_160 1 160 -25.700 500 -22.271 3.010 -1.139

YFL005W-YFL004W_6_165 6 160 -23.600 500 -20.505 3.144 -0.984

YFL005W-YFL004W_11_170 11 160 -20.960 500 -17.974 2.810 -1.063

YFL005W-YFL004W_16_175 16 160 -21.200 500 -19.565 2.959 -0.553

YFL005W-YFL004W_21_180 21 160 -19.800 500 -19.705 2.900 -0.033

YFL005W-YFL004W_26_185 26 160 -18.150 500 -19.771 2.986 0.543

YFL005W-YFL004W_31_190 31 160 -17.500 500 -18.213 2.851 0.250

YFL005W-YFL004W_36_195 36 160 -17.200 500 -17.962 2.745 0.278

YFL005W-YFL004W_41_200 41 160 -15.300 500 -16.530 2.607 0.472

YFL005W-YFL004W_46_205 46 160 -14.200 500 -13.797 2.669 -0.151

YFL005W-YFL004W_51_210 51 160 -10.890 500 -12.641 2.720 0.644

YFL005W-YFL004W_56_215 56 160 -11.100 500 -11.663 2.624 0.214

YFL005W-YFL004W_61_220 61 160 -11.400 500 -10.984 2.413 -0.172

YFL005W-YFL004W_66_225 66 160 -11.100 500 -10.115 2.293 -0.429

YFL005W-YFL004W_71_230 71 160 -11.100 500 -9.222 2.294 -0.819

YFL005W-YFL004W_76_235 76 160 -11.900 500 -9.883 2.494 -0.809

YFL005W-YFL004W_81_240 81 160 -11.900 500 -10.809 2.417 -0.452

YFL005W-YFL004W_86_245 86 160 -11.900 500 -10.789 2.558 -0.434

YFL005W-YFL004W_91_250 91 160 -12.600 500 -12.777 2.747 0.065

YFL005W-YFL004W_96_255 96 160 -12.600 500 -12.993 2.419 0.162

YFL005W-YFL004W_101_260 101 160 -10.700 500 -11.334 2.573 0.246

YFL005W-YFL004W_106_265 106 160 -14.600 500 -14.433 2.691 -0.062

YFL005W-YFL004W_111_270 111 160 -16.100 500 -16.863 2.704 0.282

YFL005W-YFL004W_116_275 116 160 -16.100 500 -16.439 2.765 0.122

YFL005W-YFL004W_121_280 121 160 -15.900 500 -17.794 2.807 0.675

YFL005W-YFL004W_126_285 126 160 -16.700 500 -18.175 2.766 0.533

YFL005W-YFL004W_131_290 131 160 -18.000 500 -18.714 2.681 0.266

YFL005W-YFL004W_136_295 136 160 -18.500 500 -18.620 2.782 0.043

YFL005W-YFL004W_141_300 141 160 -19.200 500 -20.807 2.813 0.571

YFL005W-YFL004W_146_305 146 160 -20.500 500 -21.007 2.950 0.172

YFL005W-YFL004W_151_310 151 160 -20.500 500 -20.403 2.838 -0.034

YFL005W-YFL004W_156_315 156 160 -18.700 500 -20.837 3.139 0.681

YFL005W-YFL004W_161_320 161 160 -18.300 500 -22.285 3.003 1.327

YFL005W-YFL004W_166_325 166 160 -19.400 500 -23.084 3.213 1.147

YFL005W-YFL004W_171_330 171 160 -19.400 500 -22.684 3.118 1.053

YFL005W-YFL004W_176_335 176 160 -21.100 500 -25.235 3.320 1.245

YFL005W-YFL004W_181_340 181 160 -18.100 500 -24.686 3.271 2.013

YFL005W-YFL004W_186_345 186 160 -18.100 500 -25.395 3.192 2.285

YFL005W-YFL004W_191_350 191 160 -23.800 500 -26.527 3.215 0.848

YFL005W-YFL004W_196_355 196 160 -26.800 500 -29.089 3.498 0.655

YFL005W-YFL004W_201_360 201 160 -28.100 500 -29.423 3.564 0.371

YFL005W-YFL004W_206_365 206 160 -29.700 500 -30.201 3.497 0.143

YFL005W-YFL004W_211_370 211 160 -31.500 500 -30.658 3.557 -0.237

YFL005W-YFL004W_216_375 216 160 -31.500 500 -30.236 3.292 -0.384

YFL005W-YFL004W_221_380 221 160 -31.500 500 -30.523 3.388 -0.288

YFL005W-YFL004W_226_385 226 160 -31.500 500 -30.766 3.358 -0.218

YFL005W-YFL004W_231_390 231 160 -31.900 500 -31.193 3.612 -0.196

YFL005W-YFL004W_236_395 236 160 -33.000 500 -31.089 3.776 -0.506

YFL005W-YFL004W_241_400 241 160 -31.500 500 -30.965 3.415 -0.157

YFL005W-YFL004W_246_405 246 160 -31.000 500 -30.377 3.553 -0.175

YFL005W-YFL004W_251_410 251 160 -32.700 500 -29.615 3.436 -0.898

YFL005W-YFL004W_256_415 256 160 -32.700 500 -29.510 3.259 -0.979

YFL005W-YFL004W_261_420 261 160 -35.900 500 -28.506 3.229 -2.290

YFL005W-YFL004W_266_425 266 160 -38.800 500 -27.472 3.596 -3.150

YFL005W-YFL004W_271_430 271 160 -39.500 500 -27.186 3.563 -3.456

YFL005W-YFL004W_276_435 276 160 -39.500 500 -28.185 3.249 -3.483

YFL005W-YFL004W_281_440 281 160 -38.400 500 -27.434 3.521 -3.115

YFL005W-YFL004W_286_445 286 160 -34.700 500 -26.348 3.261 -2.561

YFL005W-YFL004W_291_450 291 160 -34.700 500 -26.676 3.349 -2.396

YFL005W-YFL004W_296_455 296 160 -32.000 500 -26.589 3.451 -1.568

YFL005W-YFL004W_301_460 301 160 -32.000 500 -26.194 3.212 -1.807

YFL005W-YFL004W_306_465 306 160 -32.300 500 -26.601 3.278 -1.739

YFL005W-YFL004W_311_470 311 160 -34.000 500 -26.933 3.306 -2.137

YFL005W-YFL004W_316_475 316 160 -33.300 500 -27.457 3.434 -1.702

YFL005W-YFL004W_321_480 321 160 -31.850 500 -27.645 3.404 -1.235

YFL005W-YFL004W_326_485 326 160 -29.000 500 -25.591 3.196 -1.066

YFL005W-YFL004W_331_490 331 160 -33.000 500 -27.747 3.409 -1.541

YFL005W-YFL004W_336_495 336 160 -31.400 500 -25.455 3.179 -1.870

YFL005W-YFL004W_341_500 341 160 -26.250 500 -24.696 3.161 -0.492

YFL005W-YFL004W_346_505 346 160 -24.400 500 -24.452 3.254 0.016

YFL005W-YFL004W_351_510 351 160 -23.250 500 -23.168 3.151 -0.026

YFL005W-YFL004W_356_515 356 160 -22.500 500 -22.671 3.027 0.056

YFL005W-YFL004W_361_520 361 160 -20.950 500 -22.859 3.298 0.579

YFL005W-YFL004W_366_525 366 160 -20.000 500 -23.478 3.303 1.053

YFL005W-YFL004W_371_530 371 160 -22.700 500 -24.902 3.240 0.680

YFL005W-YFL004W_376_535 376 160 -26.100 500 -26.580 3.199 0.150

YFL005W-YFL004W_381_540 381 160 -23.700 500 -27.502 3.504 1.085

YFL005W-YFL004W_386_545 386 160 -25.000 500 -27.898 3.398 0.853

YFL005W-YFL004W_391_550 391 160 -25.000 500 -28.857 3.551 1.086

YFL005W-YFL004W_396_555 396 160 -25.800 500 -29.422 3.197 1.133

YFL005W-YFL004W_401_560 401 160 -26.900 500 -30.704 3.436 1.107

YFL005W-YFL004W_406_565 406 160 -29.000 500 -31.208 3.320 0.665

YFL005W-YFL004W_411_570 411 160 -29.200 500 -32.290 3.343 0.924

YFL005W-YFL004W_416_575 416 160 -29.000 500 -32.367 3.402 0.990

YFL005W-YFL004W_421_580 421 160 -32.000 500 -31.928 3.618 -0.020

YFL005W-YFL004W_426_585 426 160 -30.400 500 -30.387 3.180 -0.004

YFL005W-YFL004W_431_590 431 160 -28.400 500 -30.747 3.424 0.685

YFL005W-YFL004W_436_595 436 160 -28.900 500 -30.044 3.424 0.334

YFL005W-YFL004W_441_600 441 160 -33.100 500 -32.937 3.461 -0.047

YFL005W-YFL004W_446_605 446 160 -34.200 500 -33.114 3.624 -0.300

YFL005W-YFL004W_451_610 451 160 -37.500 500 -34.003 3.612 -0.968

YFL005W-YFL004W_456_615 456 160 -38.200 500 -35.691 3.602 -0.697

YFL005W-YFL004W_461_620 461 160 -40.700 500 -35.368 3.559 -1.498

YFL005W-YFL004W_466_625 466 160 -41.500 500 -34.051 3.642 -2.045

YFL005W-YFL004W_471_630 471 160 -38.500 500 -33.702 3.531 -1.359

YFL005W-YFL004W_476_635 476 160 -35.900 500 -33.035 3.535 -0.810

YFL005W-YFL004W_481_640 481 160 -36.000 500 -32.788 3.531 -0.910

YFL005W-YFL004W_486_645 486 160 -35.400 500 -31.359 3.579 -1.129

YFL005W-YFL004W_491_650 491 160 -34.600 500 -30.909 3.558 -1.037

YFL005W-YFL004W_496_655 496 160 -34.400 500 -30.558 3.314 -1.159

YFL005W-YFL004W_501_660 501 160 -36.800 500 -30.907 3.651 -1.614

YFL005W-YFL004W_506_665 506 160 -36.500 500 -30.453 3.235 -1.869

YFL005W-YFL004W_511_670 511 160 -33.400 500 -29.940 3.539 -0.978

YFL005W-YFL004W_516_675 516 160 -34.000 500 -30.812 3.336 -0.956

YFL005W-YFL004W_521_680 521 160 -33.880 500 -31.150 3.411 -0.800

YFL005W-YFL004W_526_685 526 160 -35.400 500 -31.040 3.256 -1.339

YFL005W-YFL004W_531_690 531 160 -36.500 500 -31.458 3.367 -1.497

YFL005W-YFL004W_536_695 536 160 -32.680 500 -31.130 3.541 -0.438

YFL005W-YFL004W_541_700 541 160 -32.200 500 -31.474 3.546 -0.205

YFL005W-YFL004W_546_705 546 160 -31.580 500 -30.352 3.342 -0.368

YFL005W-YFL004W_551_710 551 160 -28.580 500 -27.136 3.061 -0.472

YFL005W-YFL004W_556_715 556 160 -27.800 500 -26.494 3.222 -0.405

YFL005W-YFL004W_561_720 561 160 -29.700 500 -29.501 3.277 -0.061

YFL005W-YFL004W_566_725 566 160 -29.400 500 -28.920 3.204 -0.150

YFL005W-YFL004W_571_730 571 160 -28.130 500 -29.780 3.456 0.477

YFL005W-YFL004W_576_735 576 160 -28.000 500 -31.525 3.356 1.050

YFL005W-YFL004W_581_740 581 160 -27.830 500 -31.409 3.405 1.051

YFL005W-YFL004W_586_745 586 160 -29.100 500 -33.142 3.632 1.113

YFL005W-YFL004W_591_750 591 160 -29.100 500 -32.576 3.433 1.013

YFL005W-YFL004W_596_755 596 160 -29.130 500 -31.775 3.437 0.770

YFL005W-YFL004W_601_760 601 160 -26.400 500 -29.818 3.654 0.936

YFL005W-YFL004W_606_765 606 160 -27.500 500 -30.857 3.429 0.979

YFL005W-YFL004W_611_770 611 160 -27.500 500 -30.760 3.293 0.990

YFL005W-YFL004W_616_775 616 160 -30.000 500 -30.468 3.496 0.134

YFL005W-YFL004W_621_780 621 160 -30.900 500 -31.846 3.550 0.266

YFL005W-YFL004W_626_785 626 160 -31.600 500 -31.554 3.420 -0.014

YFL005W-YFL004W_631_790 631 160 -31.700 500 -31.330 3.371 -0.110

YFL005W-YFL004W_636_795 636 160 -37.700 500 -31.382 3.734 -1.692

YFL005W-YFL004W_641_800 641 160 -35.000 500 -30.653 3.469 -1.253

YFL005W-YFL004W_646_805 646 160 -32.700 500 -29.658 3.467 -0.878

YFL005W-YFL004W_651_810 651 160 -32.700 500 -28.814 3.385 -1.148

YFL005W-YFL004W_656_815 656 160 -30.200 500 -27.197 3.625 -0.828

YFL005W-YFL004W_661_820 661 160 -30.200 500 -26.722 3.379 -1.029

YFL005W-YFL004W_666_825 666 160 -31.100 500 -26.565 3.373 -1.345

YFL005W-YFL004W_670_828 670 158 -35.900 500 -26.551 3.371 -2.773

Window size = 165

YFL005W-YFL004W_1_165 1 165 -25.700 500 -22.459 3.191 -1.015

YFL005W-YFL004W_6_170 6 165 -23.860 500 -21.179 3.103 -0.864

YFL005W-YFL004W_11_175 11 165 -22.300 500 -19.105 2.826 -1.131

YFL005W-YFL004W_16_180 16 165 -21.200 500 -20.507 2.885 -0.240

YFL005W-YFL004W_21_185 21 165 -21.700 500 -21.363 3.046 -0.111

YFL005W-YFL004W_26_190 26 165 -18.650 500 -19.957 3.032 0.431

YFL005W-YFL004W_31_195 31 165 -17.500 500 -18.886 3.036 0.457

YFL005W-YFL004W_36_200 36 165 -17.200 500 -18.291 2.867 0.381

YFL005W-YFL004W_41_205 41 165 -16.000 500 -17.084 2.880 0.376

YFL005W-YFL004W_46_210 46 165 -14.200 500 -14.257 2.749 0.021

YFL005W-YFL004W_51_215 51 165 -12.000 500 -12.741 2.710 0.273

YFL005W-YFL004W_56_220 56 165 -11.400 500 -11.522 2.585 0.047

YFL005W-YFL004W_61_225 61 165 -11.400 500 -11.045 2.499 -0.142

YFL005W-YFL004W_66_230 66 165 -11.100 500 -9.835 2.212 -0.572

YFL005W-YFL004W_71_235 71 165 -11.900 500 -9.605 2.422 -0.947

YFL005W-YFL004W_76_240 76 165 -11.900 500 -10.439 2.444 -0.598

YFL005W-YFL004W_81_245 81 165 -11.900 500 -10.819 2.618 -0.413

YFL005W-YFL004W_86_250 86 165 -13.000 500 -13.265 2.516 0.105

YFL005W-YFL004W_91_255 91 165 -12.600 500 -12.922 2.656 0.121

YFL005W-YFL004W_96_260 96 165 -12.600 500 -13.601 2.614 0.383

YFL005W-YFL004W_101_265 101 165 -14.600 500 -14.287 2.802 -0.112

YFL005W-YFL004W_106_270 106 165 -16.100 500 -16.667 2.706 0.209

YFL005W-YFL004W_111_275 111 165 -16.100 500 -16.854 2.778 0.271

YFL005W-YFL004W_116_280 116 165 -16.100 500 -18.366 2.926 0.774

YFL005W-YFL004W_121_285 121 165 -18.700 500 -18.730 2.838 0.011

YFL005W-YFL004W_126_290 126 165 -18.400 500 -20.408 2.919 0.688

YFL005W-YFL004W_131_295 131 165 -18.500 500 -18.701 2.751 0.073

YFL005W-YFL004W_136_300 136 165 -20.900 500 -20.910 2.977 0.003

YFL005W-YFL004W_141_305 141 165 -20.500 500 -22.647 3.201 0.671

YFL005W-YFL004W_146_310 146 165 -20.500 500 -20.986 3.070 0.158

YFL005W-YFL004W_151_315 151 165 -20.500 500 -20.627 2.946 0.043

YFL005W-YFL004W_156_320 156 165 -21.800 500 -22.795 3.154 0.315

YFL005W-YFL004W_161_325 161 165 -19.400 500 -23.342 3.137 1.257

YFL005W-YFL004W_166_330 166 165 -19.400 500 -24.220 3.360 1.434

YFL005W-YFL004W_171_335 171 165 -21.400 500 -25.174 3.027 1.247

YFL005W-YFL004W_176_340 176 165 -21.600 500 -26.386 3.421 1.399

YFL005W-YFL004W_181_345 181 165 -18.100 500 -26.577 3.200 2.649

YFL005W-YFL004W_186_350 186 165 -23.800 500 -27.158 3.207 1.047

YFL005W-YFL004W_191_355 191 165 -26.800 500 -29.401 3.555 0.732

YFL005W-YFL004W_196_360 196 165 -28.100 500 -29.746 3.510 0.469

YFL005W-YFL004W_201_365 201 165 -29.700 500 -30.476 3.735 0.208

YFL005W-YFL004W_206_370 206 165 -31.500 500 -30.666 3.558 -0.234

YFL005W-YFL004W_211_375 211 165 -31.500 500 -30.671 3.514 -0.236

YFL005W-YFL004W_216_380 216 165 -31.500 500 -30.550 3.335 -0.285

YFL005W-YFL004W_221_385 221 165 -31.500 500 -31.503 3.478 0.001

YFL005W-YFL004W_226_390 226 165 -32.900 500 -31.491 3.381 -0.417

YFL005W-YFL004W_231_395 231 165 -34.000 500 -32.092 3.580 -0.533

YFL005W-YFL004W_236_400 236 165 -33.000 500 -31.613 3.574 -0.388

YFL005W-YFL004W_241_405 241 165 -35.100 500 -31.442 3.419 -1.070

YFL005W-YFL004W_246_410 246 165 -32.700 500 -31.040 3.519 -0.472

YFL005W-YFL004W_251_415 251 165 -32.700 500 -30.777 3.587 -0.536

YFL005W-YFL004W_256_420 256 165 -40.200 500 -30.289 3.737 -2.652

YFL005W-YFL004W_261_425 261 165 -38.800 500 -30.113 3.385 -2.566

YFL005W-YFL004W_266_430 266 165 -39.500 500 -27.652 3.407 -3.478

YFL005W-YFL004W_271_435 271 165 -39.500 500 -28.677 3.667 -2.951

YFL005W-YFL004W_276_440 276 165 -39.500 500 -29.235 3.300 -3.111

YFL005W-YFL004W_281_445 281 165 -38.400 500 -28.958 3.562 -2.650

YFL005W-YFL004W_286_450 286 165 -34.700 500 -27.198 3.436 -2.184

YFL005W-YFL004W_291_455 291 165 -34.700 500 -27.169 3.312 -2.274

YFL005W-YFL004W_296_460 296 165 -32.100 500 -27.413 3.374 -1.389

YFL005W-YFL004W_301_465 301 165 -32.300 500 -27.778 3.394 -1.332

YFL005W-YFL004W_306_470 306 165 -34.000 500 -27.387 3.189 -2.074

YFL005W-YFL004W_311_475 311 165 -36.200 500 -28.449 3.315 -2.338

YFL005W-YFL004W_316_480 316 165 -33.300 500 -29.082 3.300 -1.278

YFL005W-YFL004W_321_485 321 165 -36.550 500 -28.877 3.207 -2.392

YFL005W-YFL004W_326_490 326 165 -33.300 500 -28.865 3.466 -1.280

YFL005W-YFL004W_331_495 331 165 -33.700 500 -28.286 3.273 -1.654

YFL005W-YFL004W_336_500 336 165 -31.400 500 -25.525 3.033 -1.937

YFL005W-YFL004W_341_505 341 165 -26.250 500 -25.013 3.176 -0.390

YFL005W-YFL004W_346_510 346 165 -24.400 500 -25.936 3.166 0.485

YFL005W-YFL004W_351_515 351 165 -23.250 500 -23.454 3.311 0.062

YFL005W-YFL004W_356_520 356 165 -22.500 500 -23.027 3.145 0.168

YFL005W-YFL004W_361_525 361 165 -21.500 500 -25.021 3.443 1.023

YFL005W-YFL004W_366_530 366 165 -22.700 500 -25.048 3.086 0.761

YFL005W-YFL004W_371_535 371 165 -26.100 500 -26.748 3.454 0.188

YFL005W-YFL004W_376_540 376 165 -26.100 500 -28.066 3.238 0.607

YFL005W-YFL004W_381_545 381 165 -25.000 500 -29.450 3.455 1.288

YFL005W-YFL004W_386_550 386 165 -26.200 500 -29.999 3.460 1.098

YFL005W-YFL004W_391_555 391 165 -26.600 500 -30.127 3.425 1.030

YFL005W-YFL004W_396_560 396 165 -26.900 500 -31.321 3.479 1.271

YFL005W-YFL004W_401_565 401 165 -29.000 500 -32.409 3.497 0.975

YFL005W-YFL004W_406_570 406 165 -29.400 500 -32.714 3.501 0.947

YFL005W-YFL004W_411_575 411 165 -29.900 500 -33.126 3.681 0.876

YFL005W-YFL004W_416_580 416 165 -32.000 500 -33.888 3.460 0.546

YFL005W-YFL004W_421_585 421 165 -32.000 500 -32.581 3.552 0.163

YFL005W-YFL004W_426_590 426 165 -30.500 500 -32.188 3.468 0.487

YFL005W-YFL004W_431_595 431 165 -28.900 500 -31.600 3.269 0.826

YFL005W-YFL004W_436_600 436 165 -33.720 500 -33.631 3.480 -0.026

YFL005W-YFL004W_441_605 441 165 -34.200 500 -33.517 3.437 -0.199

YFL005W-YFL004W_446_610 446 165 -38.200 500 -34.475 3.502 -1.063

YFL005W-YFL004W_451_615 451 165 -39.200 500 -36.402 3.591 -0.779

YFL005W-YFL004W_456_620 456 165 -40.700 500 -35.559 3.404 -1.510

YFL005W-YFL004W_461_625 461 165 -41.500 500 -36.493 3.756 -1.333

YFL005W-YFL004W_466_630 466 165 -41.500 500 -35.129 3.572 -1.784

YFL005W-YFL004W_471_635 471 165 -38.500 500 -35.072 3.665 -0.935

YFL005W-YFL004W_476_640 476 165 -36.000 500 -33.313 3.399 -0.790

YFL005W-YFL004W_481_645 481 165 -36.000 500 -33.287 3.536 -0.767

YFL005W-YFL004W_486_650 486 165 -37.130 500 -32.886 3.419 -1.241

YFL005W-YFL004W_491_655 491 165 -34.600 500 -30.832 3.554 -1.060

YFL005W-YFL004W_496_660 496 165 -36.800 500 -31.708 3.510 -1.451

YFL005W-YFL004W_501_665 501 165 -36.800 500 -30.898 3.509 -1.682

YFL005W-YFL004W_506_670 506 165 -36.500 500 -30.430 3.506 -1.731

YFL005W-YFL004W_511_675 511 165 -34.000 500 -30.643 3.415 -0.983

YFL005W-YFL004W_516_680 516 165 -34.000 500 -31.368 3.616 -0.728

YFL005W-YFL004W_521_685 521 165 -35.400 500 -33.499 3.738 -0.509

YFL005W-YFL004W_526_690 526 165 -38.500 500 -34.525 3.608 -1.102

YFL005W-YFL004W_531_695 531 165 -36.500 500 -31.930 3.371 -1.356

YFL005W-YFL004W_536_700 536 165 -33.180 500 -32.275 3.537 -0.256

YFL005W-YFL004W_541_705 541 165 -32.200 500 -31.625 3.431 -0.168

YFL005W-YFL004W_546_710 546 165 -31.580 500 -30.552 3.550 -0.290

YFL005W-YFL004W_551_715 551 165 -28.580 500 -27.987 3.315 -0.179

YFL005W-YFL004W_556_720 556 165 -33.930 500 -30.085 3.377 -1.138

YFL005W-YFL004W_561_725 561 165 -29.700 500 -30.666 3.565 0.271

YFL005W-YFL004W_566_730 566 165 -33.630 500 -31.196 3.440 -0.707

YFL005W-YFL004W_571_735 571 165 -29.630 500 -32.683 3.439 0.888

YFL005W-YFL004W_576_740 576 165 -30.100 500 -33.884 3.463 1.093

YFL005W-YFL004W_581_745 581 165 -29.100 500 -33.508 3.464 1.273

YFL005W-YFL004W_586_750 586 165 -29.100 500 -33.772 3.426 1.364

YFL005W-YFL004W_591_755 591 165 -29.130 500 -33.941 3.544 1.357

YFL005W-YFL004W_596_760 596 165 -29.130 500 -31.772 3.565 0.741

YFL005W-YFL004W_601_765 601 165 -27.500 500 -32.025 3.617 1.251

YFL005W-YFL004W_606_770 606 165 -28.130 500 -31.736 3.563 1.012

YFL005W-YFL004W_611_775 611 165 -31.530 500 -32.232 3.709 0.189

YFL005W-YFL004W_616_780 616 165 -30.900 500 -32.267 3.684 0.371

YFL005W-YFL004W_621_785 621 165 -33.400 500 -31.848 3.594 -0.432

YFL005W-YFL004W_626_790 626 165 -32.700 500 -33.456 3.549 0.213

YFL005W-YFL004W_631_795 631 165 -38.500 500 -31.833 3.579 -1.863

YFL005W-YFL004W_636_800 636 165 -37.700 500 -32.716 3.603 -1.383

YFL005W-YFL004W_641_805 641 165 -35.000 500 -31.410 3.580 -1.003

YFL005W-YFL004W_646_810 646 165 -32.700 500 -29.869 3.726 -0.760

YFL005W-YFL004W_651_815 651 165 -32.700 500 -28.571 3.434 -1.203

YFL005W-YFL004W_656_820 656 165 -30.200 500 -28.090 3.336 -0.632

YFL005W-YFL004W_661_825 661 165 -31.100 500 -28.217 3.602 -0.800

YFL005W-YFL004W_665_828 665 163 -35.900 500 -27.625 3.492 -2.370

Window size = 170

YFL005W-YFL004W_1_170 1 170 -25.700 500 -22.921 2.981 -0.932

YFL005W-YFL004W_6_175 6 170 -25.200 500 -22.140 3.048 -1.004

YFL005W-YFL004W_11_180 11 170 -22.300 500 -20.163 2.976 -0.718

YFL005W-YFL004W_16_185 16 170 -23.100 500 -22.170 3.003 -0.310

YFL005W-YFL004W_21_190 21 170 -22.200 500 -21.044 2.837 -0.407

YFL005W-YFL004W_26_195 26 170 -18.650 500 -20.320 2.944 0.567

YFL005W-YFL004W_31_200 31 170 -17.500 500 -19.096 2.899 0.551

YFL005W-YFL004W_36_205 36 170 -17.300 500 -18.860 2.872 0.543

YFL005W-YFL004W_41_210 41 170 -16.890 500 -17.215 2.784 0.117

YFL005W-YFL004W_46_215 46 170 -14.200 500 -14.605 2.813 0.144

YFL005W-YFL004W_51_220 51 170 -12.000 500 -12.773 2.656 0.291

YFL005W-YFL004W_56_225 56 170 -11.400 500 -11.777 2.517 0.150

YFL005W-YFL004W_61_230 61 170 -11.400 500 -11.027 2.325 -0.160

YFL005W-YFL004W_66_235 66 170 -11.900 500 -10.198 2.338 -0.728

YFL005W-YFL004W_71_240 71 170 -11.900 500 -10.225 2.326 -0.720

YFL005W-YFL004W_76_245 76 170 -11.900 500 -10.484 2.470 -0.573

YFL005W-YFL004W_81_250 81 170 -13.000 500 -12.779 2.423 -0.091

YFL005W-YFL004W_86_255 86 170 -13.000 500 -13.660 2.804 0.236

YFL005W-YFL004W_91_260 91 170 -16.900 500 -13.938 2.568 -1.154

YFL005W-YFL004W_96_265 96 170 -16.500 500 -16.829 2.756 0.119

YFL005W-YFL004W_101_270 101 170 -16.100 500 -16.687 2.864 0.205

YFL005W-YFL004W_106_275 106 170 -16.100 500 -16.416 2.661 0.119

YFL005W-YFL004W_111_280 111 170 -16.100 500 -18.725 2.717 0.966

YFL005W-YFL004W_116_285 116 170 -18.700 500 -19.326 2.672 0.234

YFL005W-YFL004W_121_290 121 170 -18.900 500 -20.983 2.801 0.744

YFL005W-YFL004W_126_295 126 170 -19.400 500 -20.861 2.992 0.488

YFL005W-YFL004W_131_300 131 170 -21.000 500 -20.990 3.046 -0.003

YFL005W-YFL004W_136_305 136 170 -22.400 500 -22.364 3.019 -0.012

YFL005W-YFL004W_141_310 141 170 -20.500 500 -22.997 3.186 0.784

YFL005W-YFL004W_146_315 146 170 -20.500 500 -21.161 2.978 0.222

YFL005W-YFL004W_151_320 151 170 -24.600 500 -22.595 3.148 -0.637

YFL005W-YFL004W_156_325 156 170 -21.800 500 -23.863 3.199 0.645

YFL005W-YFL004W_161_330 161 170 -19.400 500 -24.543 3.435 1.497

YFL005W-YFL004W_166_335 166 170 -21.400 500 -26.086 3.279 1.429

YFL005W-YFL004W_171_340 171 170 -21.900 500 -26.397 3.276 1.373

YFL005W-YFL004W_176_345 176 170 -21.600 500 -28.306 3.491 1.921

YFL005W-YFL004W_181_350 181 170 -23.800 500 -28.184 3.348 1.310

YFL005W-YFL004W_186_355 186 170 -26.800 500 -29.605 3.526 0.796

YFL005W-YFL004W_191_360 191 170 -28.100 500 -29.594 3.410 0.438

YFL005W-YFL004W_196_365 196 170 -29.700 500 -30.437 3.431 0.215

YFL005W-YFL004W_201_370 201 170 -31.500 500 -30.681 3.496 -0.234

YFL005W-YFL004W_206_375 206 170 -31.500 500 -31.010 3.556 -0.138

YFL005W-YFL004W_211_380 211 170 -31.500 500 -31.518 3.429 0.005

YFL005W-YFL004W_216_385 216 170 -31.500 500 -31.953 3.396 0.133

YFL005W-YFL004W_221_390 221 170 -32.900 500 -32.199 3.577 -0.196

YFL005W-YFL004W_226_395 226 170 -36.600 500 -33.111 3.728 -0.936

YFL005W-YFL004W_231_400 231 170 -34.000 500 -32.258 3.620 -0.481

YFL005W-YFL004W_236_405 236 170 -35.100 500 -32.287 3.292 -0.854

YFL005W-YFL004W_241_410 241 170 -35.200 500 -32.256 3.337 -0.882

YFL005W-YFL004W_246_415 246 170 -32.700 500 -31.756 3.514 -0.269

YFL005W-YFL004W_251_420 251 170 -40.300 500 -31.613 3.547 -2.449

YFL005W-YFL004W_256_425 256 170 -42.000 500 -32.280 3.640 -2.670

YFL005W-YFL004W_261_430 261 170 -39.500 500 -31.043 3.751 -2.255

YFL005W-YFL004W_266_435 266 170 -39.500 500 -29.352 3.501 -2.899

YFL005W-YFL004W_271_440 271 170 -39.500 500 -29.532 3.362 -2.965

YFL005W-YFL004W_276_445 276 170 -40.200 500 -30.763 3.503 -2.694

YFL005W-YFL004W_281_450 281 170 -38.400 500 -29.849 3.649 -2.344

YFL005W-YFL004W_286_455 286 170 -34.700 500 -27.026 3.326 -2.307

YFL005W-YFL004W_291_460 291 170 -34.700 500 -27.359 3.212 -2.285

YFL005W-YFL004W_296_465 296 170 -32.300 500 -29.057 3.598 -0.901

YFL005W-YFL004W_301_470 301 170 -34.000 500 -28.687 3.459 -1.536

YFL005W-YFL004W_306_475 306 170 -36.200 500 -29.324 3.528 -1.949

YFL005W-YFL004W_311_480 311 170 -36.200 500 -30.011 3.347 -1.849

YFL005W-YFL004W_316_485 316 170 -36.850 500 -30.144 3.347 -2.004

YFL005W-YFL004W_321_490 321 170 -36.550 500 -31.412 3.368 -1.525

YFL005W-YFL004W_326_495 326 170 -33.700 500 -29.270 3.350 -1.322

YFL005W-YFL004W_331_500 331 170 -33.800 500 -28.725 3.493 -1.453

YFL005W-YFL004W_336_505 336 170 -31.400 500 -25.821 3.094 -1.803

YFL005W-YFL004W_341_510 341 170 -26.250 500 -26.316 3.184 0.021

YFL005W-YFL004W_346_515 346 170 -24.400 500 -26.442 3.057 0.668

YFL005W-YFL004W_351_520 351 170 -23.250 500 -23.716 2.978 0.157

YFL005W-YFL004W_356_525 356 170 -23.600 500 -24.820 3.147 0.388

YFL005W-YFL004W_361_530 361 170 -24.300 500 -26.852 3.313 0.770

YFL005W-YFL004W_366_535 366 170 -26.100 500 -27.254 3.513 0.328

YFL005W-YFL004W_371_540 371 170 -27.500 500 -28.243 3.280 0.226

YFL005W-YFL004W_376_545 376 170 -26.100 500 -29.511 3.257 1.047

YFL005W-YFL004W_381_550 381 170 -26.800 500 -31.258 3.325 1.340

YFL005W-YFL004W_386_555 386 170 -26.800 500 -31.742 3.212 1.539

YFL005W-YFL004W_391_560 391 170 -26.900 500 -31.860 3.327 1.491

YFL005W-YFL004W_396_565 396 170 -29.000 500 -32.514 3.329 1.056

YFL005W-YFL004W_401_570 401 170 -29.700 500 -33.661 3.609 1.098

YFL005W-YFL004W_406_575 406 170 -31.000 500 -33.878 3.634 0.792

YFL005W-YFL004W_411_580 411 170 -32.000 500 -34.630 3.516 0.748

YFL005W-YFL004W_416_585 416 170 -32.000 500 -34.279 3.681 0.619

YFL005W-YFL004W_421_590 421 170 -32.000 500 -33.977 3.502 0.564

YFL005W-YFL004W_426_595 426 170 -30.500 500 -32.997 3.572 0.699

YFL005W-YFL004W_431_600 431 170 -34.400 500 -35.160 3.683 0.206

YFL005W-YFL004W_436_605 436 170 -34.300 500 -34.291 3.446 -0.003

YFL005W-YFL004W_441_610 441 170 -38.200 500 -34.947 3.393 -0.959

YFL005W-YFL004W_446_615 446 170 -39.200 500 -36.877 3.538 -0.656

YFL005W-YFL004W_451_620 451 170 -40.700 500 -36.559 3.582 -1.156

YFL005W-YFL004W_456_625 456 170 -41.500 500 -36.603 3.397 -1.442

YFL005W-YFL004W_461_630 461 170 -43.000 500 -37.639 3.748 -1.430

YFL005W-YFL004W_466_635 466 170 -41.500 500 -36.179 3.570 -1.490

YFL005W-YFL004W_471_640 471 170 -39.800 500 -35.690 3.729 -1.102

YFL005W-YFL004W_476_645 476 170 -36.000 500 -33.982 3.622 -0.557

YFL005W-YFL004W_481_650 481 170 -37.730 500 -34.941 3.507 -0.795

YFL005W-YFL004W_486_655 486 170 -37.730 500 -33.011 3.561 -1.325

YFL005W-YFL004W_491_660 491 170 -36.800 500 -31.412 3.690 -1.460

YFL005W-YFL004W_496_665 496 170 -36.800 500 -31.585 3.625 -1.439

YFL005W-YFL004W_501_670 501 170 -36.800 500 -31.319 3.589 -1.527

YFL005W-YFL004W_506_675 506 170 -36.500 500 -31.111 3.548 -1.519

YFL005W-YFL004W_511_680 511 170 -34.000 500 -31.274 3.576 -0.762

YFL005W-YFL004W_516_685 516 170 -35.400 500 -34.235 3.780 -0.308

YFL005W-YFL004W_521_690 521 170 -39.480 500 -37.059 3.714 -0.652

YFL005W-YFL004W_526_695 526 170 -38.530 500 -35.165 3.851 -0.874

YFL005W-YFL004W_531_700 531 170 -36.500 500 -33.459 3.461 -0.879

YFL005W-YFL004W_536_705 536 170 -33.180 500 -32.251 3.590 -0.259

YFL005W-YFL004W_541_710 541 170 -32.200 500 -31.626 3.409 -0.168

YFL005W-YFL004W_546_715 546 170 -31.580 500 -31.290 3.456 -0.084

YFL005W-YFL004W_551_720 551 170 -33.930 500 -31.277 3.485 -0.761

YFL005W-YFL004W_556_725 556 170 -34.530 500 -31.202 3.604 -0.923

YFL005W-YFL004W_561_730 561 170 -34.800 500 -32.832 3.307 -0.595

YFL005W-YFL004W_566_735 566 170 -35.730 500 -34.022 3.537 -0.483

YFL005W-YFL004W_571_740 571 170 -31.700 500 -35.019 3.671 0.904

YFL005W-YFL004W_576_745 576 170 -32.600 500 -36.093 3.532 0.989

YFL005W-YFL004W_581_750 581 170 -29.100 500 -33.927 3.359 1.437

YFL005W-YFL004W_586_755 586 170 -29.130 500 -35.045 3.827 1.545

YFL005W-YFL004W_591_760 591 170 -29.130 500 -34.263 3.860 1.330

YFL005W-YFL004W_596_765 596 170 -29.130 500 -33.932 3.547 1.354

YFL005W-YFL004W_601_770 601 170 -28.730 500 -32.655 3.513 1.117

YFL005W-YFL004W_606_775 606 170 -33.030 500 -32.795 3.568 -0.066

YFL005W-YFL004W_611_780 611 170 -32.430 500 -34.014 3.440 0.460

YFL005W-YFL004W_616_785 616 170 -33.400 500 -32.667 3.533 -0.208

YFL005W-YFL004W_621_790 621 170 -34.500 500 -33.357 3.766 -0.304

YFL005W-YFL004W_626_795 626 170 -38.500 500 -34.034 3.692 -1.210

YFL005W-YFL004W_631_800 631 170 -38.500 500 -33.342 3.806 -1.355

YFL005W-YFL004W_636_805 636 170 -37.700 500 -32.828 3.768 -1.293

YFL005W-YFL004W_641_810 641 170 -35.000 500 -31.389 3.587 -1.007

YFL005W-YFL004W_646_815 646 170 -32.700 500 -29.798 3.320 -0.874

YFL005W-YFL004W_651_820 651 170 -32.700 500 -29.239 3.383 -1.023

YFL005W-YFL004W_656_825 656 170 -31.100 500 -29.155 3.552 -0.548

YFL005W-YFL004W_660_828 660 168 -35.900 500 -28.520 3.564 -2.071

Window size = 175

YFL005W-YFL004W_1_175 1 175 -25.900 500 -24.426 3.266 -0.451

YFL005W-YFL004W_6_180 6 175 -25.200 500 -23.323 3.007 -0.624

YFL005W-YFL004W_11_185 11 175 -24.200 500 -21.848 2.976 -0.790

YFL005W-YFL004W_16_190 16 175 -23.600 500 -22.316 3.076 -0.417

YFL005W-YFL004W_21_195 21 175 -22.200 500 -21.677 2.927 -0.179

YFL005W-YFL004W_26_200 26 175 -18.650 500 -20.711 2.910 0.708

YFL005W-YFL004W_31_205 31 175 -18.550 500 -19.548 2.903 0.344

YFL005W-YFL004W_36_210 36 175 -18.190 500 -19.215 2.923 0.351

YFL005W-YFL004W_41_215 41 175 -18.100 500 -17.627 2.716 -0.174

YFL005W-YFL004W_46_220 46 175 -14.200 500 -14.866 2.698 0.247

YFL005W-YFL004W_51_225 51 175 -12.000 500 -13.167 2.751 0.424

YFL005W-YFL004W_56_230 56 175 -11.400 500 -11.738 2.487 0.136

YFL005W-YFL004W_61_235 61 175 -15.000 500 -11.488 2.520 -1.393

YFL005W-YFL004W_66_240 66 175 -11.900 500 -10.984 2.437 -0.376

YFL005W-YFL004W_71_245 71 175 -11.900 500 -10.046 2.500 -0.742

YFL005W-YFL004W_76_250 76 175 -13.000 500 -12.487 2.604 -0.197

YFL005W-YFL004W_81_255 81 175 -13.000 500 -13.158 2.646 0.060

YFL005W-YFL004W_86_260 86 175 -16.900 500 -14.296 2.812 -0.926

YFL005W-YFL004W_91_265 91 175 -17.700 500 -16.986 2.834 -0.252

YFL005W-YFL004W_96_270 96 175 -17.800 500 -19.106 2.772 0.471

YFL005W-YFL004W_101_275 101 175 -16.100 500 -16.543 2.702 0.164

YFL005W-YFL004W_106_280 106 175 -18.060 500 -18.684 2.755 0.227

YFL005W-YFL004W_111_285 111 175 -18.700 500 -19.670 2.854 0.340

YFL005W-YFL004W_116_290 116 175 -20.000 500 -21.499 2.712 0.553

YFL005W-YFL004W_121_295 121 175 -19.400 500 -21.287 2.851 0.662

YFL005W-YFL004W_126_300 126 175 -21.000 500 -22.683 3.072 0.548

YFL005W-YFL004W_131_305 131 175 -23.400 500 -22.864 3.056 -0.175

YFL005W-YFL004W_136_310 136 175 -22.400 500 -23.178 3.074 0.253

YFL005W-YFL004W_141_315 141 175 -20.500 500 -22.992 3.093 0.806

YFL005W-YFL004W_146_320 146 175 -24.600 500 -23.293 2.937 -0.445

YFL005W-YFL004W_151_325 151 175 -24.600 500 -24.003 3.086 -0.193

YFL005W-YFL004W_156_330 156 175 -21.800 500 -25.290 3.370 1.036

YFL005W-YFL004W_161_335 161 175 -21.400 500 -26.868 3.417 1.600

YFL005W-YFL004W_166_340 166 175 -21.900 500 -27.681 3.358 1.721

YFL005W-YFL004W_171_345 171 175 -21.900 500 -28.109 3.440 1.805

YFL005W-YFL004W_176_350 176 175 -27.300 500 -29.668 3.266 0.725

YFL005W-YFL004W_181_355 181 175 -26.800 500 -30.557 3.537 1.062

YFL005W-YFL004W_186_360 186 175 -28.100 500 -30.150 3.350 0.612

YFL005W-YFL004W_191_365 191 175 -29.700 500 -30.424 3.574 0.202

YFL005W-YFL004W_196_370 196 175 -31.500 500 -31.110 3.547 -0.110

YFL005W-YFL004W_201_375 201 175 -31.500 500 -31.097 3.345 -0.121

YFL005W-YFL004W_206_380 206 175 -31.500 500 -31.413 3.562 -0.025

YFL005W-YFL004W_211_385 211 175 -31.500 500 -32.652 3.448 0.334

YFL005W-YFL004W_216_390 216 175 -32.900 500 -32.281 3.461 -0.179

YFL005W-YFL004W_221_395 221 175 -36.700 500 -33.621 3.497 -0.881

YFL005W-YFL004W_226_400 226 175 -36.700 500 -32.852 3.237 -1.189

YFL005W-YFL004W_231_405 231 175 -35.100 500 -33.083 3.750 -0.538

YFL005W-YFL004W_236_410 236 175 -36.600 500 -32.800 3.510 -1.083

YFL005W-YFL004W_241_415 241 175 -35.200 500 -33.299 3.532 -0.538

YFL005W-YFL004W_246_420 246 175 -40.300 500 -32.806 3.589 -2.088

YFL005W-YFL004W_251_425 251 175 -42.000 500 -33.860 3.605 -2.258

YFL005W-YFL004W_256_430 256 175 -42.000 500 -33.067 3.761 -2.376

YFL005W-YFL004W_261_435 261 175 -39.500 500 -31.681 3.416 -2.289

YFL005W-YFL004W_266_440 266 175 -39.500 500 -30.062 3.492 -2.703

YFL005W-YFL004W_271_445 271 175 -42.900 500 -31.465 3.729 -3.066

YFL005W-YFL004W_276_450 276 175 -40.200 500 -31.986 3.565 -2.304

YFL005W-YFL004W_281_455 281 175 -39.800 500 -29.876 3.573 -2.778

YFL005W-YFL004W_286_460 286 175 -36.500 500 -28.075 3.432 -2.455

YFL005W-YFL004W_291_465 291 175 -34.700 500 -29.418 3.510 -1.505

YFL005W-YFL004W_296_470 296 175 -34.000 500 -30.111 3.456 -1.125

YFL005W-YFL004W_301_475 301 175 -36.200 500 -30.700 3.699 -1.487

YFL005W-YFL004W_306_480 306 175 -36.200 500 -30.659 3.470 -1.597

YFL005W-YFL004W_311_485 311 175 -36.850 500 -31.275 3.540 -1.575

YFL005W-YFL004W_316_490 316 175 -37.200 500 -33.777 3.466 -0.988

YFL005W-YFL004W_321_495 321 175 -36.550 500 -31.888 3.373 -1.382

YFL005W-YFL004W_326_500 326 175 -34.100 500 -29.610 3.408 -1.317

YFL005W-YFL004W_331_505 331 175 -35.500 500 -29.079 3.401 -1.888

YFL005W-YFL004W_336_510 336 175 -31.400 500 -27.348 3.382 -1.198

YFL005W-YFL004W_341_515 341 175 -26.250 500 -26.635 3.358 0.115

YFL005W-YFL004W_346_520 346 175 -27.000 500 -27.093 3.318 0.028

YFL005W-YFL004W_351_525 351 175 -26.500 500 -25.294 3.252 -0.371

YFL005W-YFL004W_356_530 356 175 -26.400 500 -26.697 3.305 0.090

YFL005W-YFL004W_361_535 361 175 -27.800 500 -28.729 3.530 0.263

YFL005W-YFL004W_366_540 366 175 -27.500 500 -28.547 3.270 0.320

YFL005W-YFL004W_371_545 371 175 -27.500 500 -29.784 3.317 0.689

YFL005W-YFL004W_376_550 376 175 -29.600 500 -31.630 3.663 0.554

YFL005W-YFL004W_381_555 381 175 -27.500 500 -33.275 3.569 1.618

YFL005W-YFL004W_386_560 386 175 -29.000 500 -33.176 3.196 1.307

YFL005W-YFL004W_391_565 391 175 -29.000 500 -33.251 3.530 1.204

YFL005W-YFL004W_396_570 396 175 -30.300 500 -33.875 3.579 0.999

YFL005W-YFL004W_401_575 401 175 -31.000 500 -34.662 3.494 1.048

YFL005W-YFL004W_406_580 406 175 -32.000 500 -35.369 3.801 0.887

YFL005W-YFL004W_411_585 411 175 -32.000 500 -35.329 3.492 0.953

YFL005W-YFL004W_416_590 416 175 -32.500 500 -35.607 3.555 0.874

YFL005W-YFL004W_421_595 421 175 -32.700 500 -34.751 3.277 0.626

YFL005W-YFL004W_426_600 426 175 -34.600 500 -36.491 3.490 0.542

YFL005W-YFL004W_431_605 431 175 -34.600 500 -35.851 3.890 0.322

YFL005W-YFL004W_436_610 436 175 -39.400 500 -35.750 3.464 -1.054

YFL005W-YFL004W_441_615 441 175 -39.400 500 -37.547 3.471 -0.534

YFL005W-YFL004W_446_620 446 175 -40.700 500 -37.098 3.518 -1.024

YFL005W-YFL004W_451_625 451 175 -41.500 500 -37.280 3.636 -1.161

YFL005W-YFL004W_456_630 456 175 -43.000 500 -38.072 3.706 -1.330

YFL005W-YFL004W_461_635 461 175 -44.100 500 -38.737 3.611 -1.485

YFL005W-YFL004W_466_640 466 175 -42.800 500 -36.828 3.644 -1.639

YFL005W-YFL004W_471_645 471 175 -39.800 500 -36.485 3.541 -0.936

YFL005W-YFL004W_476_650 476 175 -37.730 500 -35.455 3.452 -0.659

YFL005W-YFL004W_481_655 481 175 -38.130 500 -35.162 3.627 -0.818

YFL005W-YFL004W_486_660 486 175 -37.730 500 -33.628 3.625 -1.132

YFL005W-YFL004W_491_665 491 175 -36.800 500 -31.788 3.533 -1.419

YFL005W-YFL004W_496_670 496 175 -36.800 500 -31.651 3.482 -1.479

YFL005W-YFL004W_501_675 501 175 -36.800 500 -31.845 3.485 -1.422

YFL005W-YFL004W_506_680 506 175 -36.500 500 -31.823 3.504 -1.335

YFL005W-YFL004W_511_685 511 175 -35.400 500 -34.234 3.691 -0.316

YFL005W-YFL004W_516_690 516 175 -39.680 500 -37.050 3.694 -0.712

YFL005W-YFL004W_521_695 521 175 -39.480 500 -37.252 3.719 -0.599

YFL005W-YFL004W_526_700 526 175 -38.630 500 -36.288 3.487 -0.672

YFL005W-YFL004W_531_705 531 175 -36.500 500 -33.636 3.416 -0.838

YFL005W-YFL004W_536_710 536 175 -33.180 500 -32.456 3.318 -0.218

YFL005W-YFL004W_541_715 541 175 -32.200 500 -32.447 3.555 0.069

YFL005W-YFL004W_546_720 546 175 -35.330 500 -34.653 3.552 -0.191

YFL005W-YFL004W_551_725 551 175 -34.730 500 -32.438 3.700 -0.620

YFL005W-YFL004W_556_730 556 175 -34.900 500 -33.198 3.433 -0.496

YFL005W-YFL004W_561_735 561 175 -36.030 500 -35.404 3.709 -0.169

YFL005W-YFL004W_566_740 566 175 -39.300 500 -36.927 3.655 -0.649

YFL005W-YFL004W_571_745 571 175 -34.300 500 -37.203 3.491 0.832

YFL005W-YFL004W_576_750 576 175 -32.600 500 -36.142 3.673 0.964

YFL005W-YFL004W_581_755 581 175 -29.130 500 -34.999 3.393 1.730

YFL005W-YFL004W_586_760 586 175 -29.300 500 -35.518 3.475 1.789

YFL005W-YFL004W_591_765 591 175 -29.900 500 -36.206 3.675 1.716

YFL005W-YFL004W_596_770 596 175 -29.130 500 -34.763 3.688 1.527

YFL005W-YFL004W_601_775 601 175 -33.030 500 -33.923 3.651 0.245

YFL005W-YFL004W_606_780 606 175 -34.030 500 -34.329 3.584 0.083

YFL005W-YFL004W_611_785 611 175 -34.930 500 -34.157 3.876 -0.199

YFL005W-YFL004W_616_790 616 175 -34.500 500 -34.146 3.642 -0.097

YFL005W-YFL004W_621_795 621 175 -38.500 500 -34.199 3.631 -1.184

YFL005W-YFL004W_626_800 626 175 -42.600 500 -35.691 3.793 -1.822

YFL005W-YFL004W_631_805 631 175 -38.500 500 -33.360 3.731 -1.378

YFL005W-YFL004W_636_810 636 175 -37.700 500 -32.896 3.918 -1.226

YFL005W-YFL004W_641_815 641 175 -35.000 500 -31.054 3.353 -1.177

YFL005W-YFL004W_646_820 646 175 -32.700 500 -30.439 3.632 -0.623

YFL005W-YFL004W_651_825 651 175 -32.700 500 -30.363 3.556 -0.657

YFL005W-YFL004W_655_828 655 173 -35.900 500 -29.811 3.572 -1.704

Window size = 180

YFL005W-YFL004W_1_180 1 180 -25.900 500 -25.530 3.134 -0.118

YFL005W-YFL004W_6_185 6 180 -27.100 500 -24.887 3.117 -0.710

YFL005W-YFL004W_11_190 11 180 -24.700 500 -21.769 2.818 -1.040

YFL005W-YFL004W_16_195 16 180 -23.890 500 -22.520 2.979 -0.460

YFL005W-YFL004W_21_200 21 180 -22.200 500 -22.266 2.809 0.024

YFL005W-YFL004W_26_205 26 180 -20.050 500 -21.555 3.137 0.480

YFL005W-YFL004W_31_210 31 180 -18.740 500 -20.049 3.114 0.420

YFL005W-YFL004W_36_215 36 180 -19.400 500 -19.224 2.780 -0.063

YFL005W-YFL004W_41_220 41 180 -18.100 500 -17.619 2.694 -0.178

YFL005W-YFL004W_46_225 46 180 -14.200 500 -14.861 2.858 0.231

YFL005W-YFL004W_51_230 51 180 -12.000 500 -13.051 2.693 0.390

YFL005W-YFL004W_56_235 56 180 -15.000 500 -12.080 2.600 -1.123

YFL005W-YFL004W_61_240 61 180 -15.000 500 -12.255 2.661 -1.031

YFL005W-YFL004W_66_245 66 180 -11.900 500 -11.088 2.570 -0.316

YFL005W-YFL004W_71_250 71 180 -13.000 500 -11.915 2.604 -0.417

YFL005W-YFL004W_76_255 76 180 -13.000 500 -12.728 2.553 -0.106

YFL005W-YFL004W_81_260 81 180 -16.900 500 -14.217 2.627 -1.021

YFL005W-YFL004W_86_265 86 180 -17.700 500 -17.432 2.748 -0.098

YFL005W-YFL004W_91_270 91 180 -19.000 500 -19.544 2.976 0.183

YFL005W-YFL004W_96_275 96 180 -17.800 500 -18.916 2.872 0.389

YFL005W-YFL004W_101_280 101 180 -18.060 500 -18.559 2.919 0.171

YFL005W-YFL004W_106_285 106 180 -18.700 500 -19.756 2.939 0.359

YFL005W-YFL004W_111_290 111 180 -20.000 500 -21.983 2.983 0.665

YFL005W-YFL004W_116_295 116 180 -20.500 500 -21.783 2.901 0.442

YFL005W-YFL004W_121_300 121 180 -21.000 500 -23.319 3.071 0.755

YFL005W-YFL004W_126_305 126 180 -23.400 500 -24.818 3.196 0.444

YFL005W-YFL004W_131_310 131 180 -23.400 500 -23.262 3.231 -0.043

YFL005W-YFL004W_136_315 136 180 -22.400 500 -23.344 3.145 0.300

YFL005W-YFL004W_141_320 141 180 -24.600 500 -24.851 3.281 0.076

YFL005W-YFL004W_146_325 146 180 -25.000 500 -24.375 3.232 -0.193

YFL005W-YFL004W_151_330 151 180 -24.600 500 -25.826 3.479 0.352

YFL005W-YFL004W_156_335 156 180 -21.800 500 -27.137 3.367 1.585

YFL005W-YFL004W_161_340 161 180 -21.900 500 -28.043 3.250 1.890

YFL005W-YFL004W_166_345 166 180 -21.900 500 -29.285 3.400 2.172

YFL005W-YFL004W_171_350 171 180 -27.600 500 -29.693 3.481 0.601

YFL005W-YFL004W_176_355 176 180 -30.300 500 -32.227 3.498 0.551

YFL005W-YFL004W_181_360 181 180 -28.100 500 -31.298 3.451 0.927

YFL005W-YFL004W_186_365 186 180 -29.700 500 -31.035 3.435 0.389

YFL005W-YFL004W_191_370 191 180 -31.500 500 -31.379 3.523 -0.034

YFL005W-YFL004W_196_375 196 180 -31.500 500 -31.391 3.712 -0.029

YFL005W-YFL004W_201_380 201 180 -31.500 500 -31.666 3.586 0.046

YFL005W-YFL004W_206_385 206 180 -31.500 500 -32.901 3.567 0.393

YFL005W-YFL004W_211_390 211 180 -32.900 500 -33.237 3.390 0.099

YFL005W-YFL004W_216_395 216 180 -36.700 500 -33.538 3.730 -0.848

YFL005W-YFL004W_221_400 221 180 -36.800 500 -33.671 3.498 -0.894

YFL005W-YFL004W_226_405 226 180 -36.700 500 -34.212 3.684 -0.675

YFL005W-YFL004W_231_410 231 180 -36.600 500 -33.874 3.659 -0.745

YFL005W-YFL004W_236_415 236 180 -36.600 500 -34.053 3.609 -0.706

YFL005W-YFL004W_241_420 241 180 -40.500 500 -34.063 3.557 -1.809

YFL005W-YFL004W_246_425 246 180 -42.000 500 -34.854 3.385 -2.111

YFL005W-YFL004W_251_430 251 180 -42.000 500 -34.412 3.438 -2.207

YFL005W-YFL004W_256_435 256 180 -42.000 500 -34.253 3.586 -2.160

YFL005W-YFL004W_261_440 261 180 -41.530 500 -33.363 3.751 -2.177

YFL005W-YFL004W_266_445 266 180 -43.100 500 -31.782 3.579 -3.162

YFL005W-YFL004W_271_450 271 180 -42.900 500 -32.059 3.525 -3.076

YFL005W-YFL004W_276_455 276 180 -40.900 500 -32.202 3.560 -2.443

YFL005W-YFL004W_281_460 281 180 -40.300 500 -30.903 3.717 -2.528

YFL005W-YFL004W_286_465 286 180 -36.600 500 -29.620 3.546 -1.968

YFL005W-YFL004W_291_470 291 180 -34.700 500 -30.108 3.490 -1.316

YFL005W-YFL004W_296_475 296 180 -36.200 500 -31.641 3.546 -1.286

YFL005W-YFL004W_301_480 301 180 -39.520 500 -32.138 3.524 -2.095

YFL005W-YFL004W_306_485 306 180 -36.850 500 -31.921 3.706 -1.330

YFL005W-YFL004W_311_490 311 180 -37.700 500 -34.245 3.518 -0.982

YFL005W-YFL004W_316_495 316 180 -40.900 500 -34.224 3.444 -1.938

YFL005W-YFL004W_321_500 321 180 -37.050 500 -32.457 3.437 -1.336

YFL005W-YFL004W_326_505 326 180 -35.800 500 -29.450 3.476 -1.827

YFL005W-YFL004W_331_510 331 180 -35.500 500 -30.165 3.233 -1.650

YFL005W-YFL004W_336_515 336 180 -31.400 500 -27.837 3.350 -1.063

YFL005W-YFL004W_341_520 341 180 -27.000 500 -27.786 3.295 0.239

YFL005W-YFL004W_346_525 346 180 -27.000 500 -28.530 3.560 0.430

YFL005W-YFL004W_351_530 351 180 -28.000 500 -27.357 3.281 -0.196

YFL005W-YFL004W_356_535 356 180 -29.700 500 -28.667 3.323 -0.311

YFL005W-YFL004W_361_540 361 180 -27.800 500 -29.892 3.575 0.585

YFL005W-YFL004W_366_545 366 180 -27.500 500 -29.910 3.464 0.696

YFL005W-YFL004W_371_550 371 180 -30.900 500 -31.717 3.462 0.236

YFL005W-YFL004W_376_555 376 180 -30.200 500 -33.358 3.577 0.883

YFL005W-YFL004W_381_560 381 180 -29.000 500 -34.715 3.367 1.697

YFL005W-YFL004W_386_565 386 180 -34.600 500 -34.741 3.321 0.042

YFL005W-YFL004W_391_570 391 180 -30.800 500 -34.421 3.634 0.997

YFL005W-YFL004W_396_575 396 180 -31.000 500 -34.733 3.369 1.108

YFL005W-YFL004W_401_580 401 180 -32.000 500 -36.441 3.588 1.238

YFL005W-YFL004W_406_585 406 180 -32.000 500 -35.380 3.430 0.985

YFL005W-YFL004W_411_590 411 180 -33.400 500 -36.850 3.547 0.973

YFL005W-YFL004W_416_595 416 180 -34.700 500 -37.198 3.804 0.657

YFL005W-YFL004W_421_600 421 180 -35.800 500 -38.371 3.827 0.672

YFL005W-YFL004W_426_605 426 180 -34.600 500 -37.366 3.594 0.770

YFL005W-YFL004W_431_610 431 180 -39.600 500 -37.233 3.642 -0.650

YFL005W-YFL004W_436_615 436 180 -40.100 500 -38.022 3.553 -0.585

YFL005W-YFL004W_441_620 441 180 -41.200 500 -37.551 3.637 -1.003

YFL005W-YFL004W_446_625 446 180 -41.500 500 -37.975 3.902 -0.904

YFL005W-YFL004W_451_630 451 180 -43.000 500 -38.536 3.775 -1.182

YFL005W-YFL004W_456_635 456 180 -44.100 500 -39.056 3.697 -1.364

YFL005W-YFL004W_461_640 461 180 -44.100 500 -39.600 3.734 -1.205

YFL005W-YFL004W_466_645 466 180 -42.800 500 -37.753 3.704 -1.363

YFL005W-YFL004W_471_650 471 180 -41.400 500 -37.756 3.591 -1.015

YFL005W-YFL004W_476_655 476 180 -38.130 500 -35.887 3.599 -0.623

YFL005W-YFL004W_481_660 481 180 -38.130 500 -36.053 3.690 -0.563

YFL005W-YFL004W_486_665 486 180 -37.730 500 -33.933 3.704 -1.025

YFL005W-YFL004W_491_670 491 180 -36.800 500 -31.912 3.518 -1.389

YFL005W-YFL004W_496_675 496 180 -36.800 500 -32.218 3.399 -1.348

YFL005W-YFL004W_501_680 501 180 -36.800 500 -32.578 3.653 -1.156

YFL005W-YFL004W_506_685 506 180 -38.500 500 -34.659 3.833 -1.002

YFL005W-YFL004W_511_690 511 180 -39.680 500 -37.264 3.752 -0.644

YFL005W-YFL004W_516_695 516 180 -39.680 500 -37.639 3.753 -0.544

YFL005W-YFL004W_521_700 521 180 -39.480 500 -38.183 3.799 -0.341

YFL005W-YFL004W_526_705 526 180 -38.630 500 -36.247 3.657 -0.652

YFL005W-YFL004W_531_710 531 180 -36.500 500 -33.780 3.410 -0.798

YFL005W-YFL004W_536_715 536 180 -33.180 500 -33.058 3.541 -0.035

YFL005W-YFL004W_541_720 541 180 -35.830 500 -35.751 3.352 -0.024

YFL005W-YFL004W_546_725 546 180 -35.330 500 -35.432 3.600 0.028

YFL005W-YFL004W_551_730 551 180 -34.900 500 -34.469 3.686 -0.117

YFL005W-YFL004W_556_735 556 180 -36.700 500 -36.055 3.438 -0.188

YFL005W-YFL004W_561_740 561 180 -39.600 500 -38.636 3.611 -0.267

YFL005W-YFL004W_566_745 566 180 -40.700 500 -38.564 3.756 -0.569

YFL005W-YFL004W_571_750 571 180 -34.900 500 -37.714 3.645 0.772

YFL005W-YFL004W_576_755 576 180 -32.630 500 -37.902 3.933 1.340

YFL005W-YFL004W_581_760 581 180 -29.400 500 -35.461 3.594 1.686

YFL005W-YFL004W_586_765 586 180 -32.400 500 -37.395 3.669 1.361

YFL005W-YFL004W_591_770 591 180 -32.300 500 -37.141 3.470 1.395

YFL005W-YFL004W_596_775 596 180 -33.030 500 -35.753 3.789 0.719

YFL005W-YFL004W_601_780 601 180 -34.030 500 -35.963 3.748 0.516

YFL005W-YFL004W_606_785 606 180 -36.430 500 -34.466 3.665 -0.536

YFL005W-YFL004W_611_790 611 180 -35.730 500 -35.879 3.602 0.041

YFL005W-YFL004W_616_795 616 180 -38.500 500 -35.164 3.853 -0.866

YFL005W-YFL004W_621_800 621 180 -42.600 500 -35.513 3.906 -1.815

YFL005W-YFL004W_626_805 626 180 -42.600 500 -35.840 3.839 -1.761

YFL005W-YFL004W_631_810 631 180 -38.500 500 -33.406 3.544 -1.437

YFL005W-YFL004W_636_815 636 180 -37.700 500 -32.388 3.814 -1.393

YFL005W-YFL004W_641_820 641 180 -35.000 500 -31.688 3.668 -0.903

YFL005W-YFL004W_646_825 646 180 -32.700 500 -31.179 3.480 -0.437

YFL005W-YFL004W_650_828 650 178 -35.900 500 -30.976 3.494 -1.409

Window size = 185

YFL005W-YFL004W_1_185 1 185 -27.400 500 -26.952 3.312 -0.135

YFL005W-YFL004W_6_190 6 185 -27.600 500 -25.204 3.185 -0.752

YFL005W-YFL004W_11_195 11 185 -24.700 500 -22.383 3.189 -0.726

YFL005W-YFL004W_16_200 16 185 -23.890 500 -22.845 3.123 -0.334

YFL005W-YFL004W_21_205 21 185 -23.600 500 -22.632 2.963 -0.327

YFL005W-YFL004W_26_210 26 185 -20.400 500 -21.445 3.223 0.324

YFL005W-YFL004W_31_215 31 185 -19.950 500 -19.933 3.107 -0.005

YFL005W-YFL004W_36_220 36 185 -21.900 500 -19.764 2.841 -0.752

YFL005W-YFL004W_41_225 41 185 -18.100 500 -17.840 2.842 -0.091

YFL005W-YFL004W_46_230 46 185 -14.200 500 -14.312 2.641 0.043

YFL005W-YFL004W_51_235 51 185 -15.000 500 -13.572 2.638 -0.541

YFL005W-YFL004W_56_240 56 185 -15.000 500 -12.838 2.454 -0.881

YFL005W-YFL004W_61_245 61 185 -15.000 500 -12.083 2.556 -1.141

YFL005W-YFL004W_66_250 66 185 -13.000 500 -12.690 2.665 -0.116

YFL005W-YFL004W_71_255 71 185 -13.000 500 -12.370 2.683 -0.235

YFL005W-YFL004W_76_260 76 185 -16.900 500 -13.615 2.684 -1.224

YFL005W-YFL004W_81_265 81 185 -17.700 500 -16.936 2.795 -0.273

YFL005W-YFL004W_86_270 86 185 -19.000 500 -19.707 2.837 0.249

YFL005W-YFL004W_91_275 91 185 -19.000 500 -19.074 2.805 0.026

YFL005W-YFL004W_96_280 96 185 -18.060 500 -21.022 3.016 0.982

YFL005W-YFL004W_101_285 101 185 -18.700 500 -19.607 2.832 0.320

YFL005W-YFL004W_106_290 106 185 -20.000 500 -21.972 2.905 0.679

YFL005W-YFL004W_111_295 111 185 -20.500 500 -22.170 3.009 0.555

YFL005W-YFL004W_116_300 116 185 -22.900 500 -23.872 3.096 0.314

YFL005W-YFL004W_121_305 121 185 -23.400 500 -25.226 3.207 0.569

YFL005W-YFL004W_126_310 126 185 -23.400 500 -24.734 3.139 0.425

YFL005W-YFL004W_131_315 131 185 -23.400 500 -23.569 3.297 0.051

YFL005W-YFL004W_136_320 136 185 -24.600 500 -25.120 3.125 0.166

YFL005W-YFL004W_141_325 141 185 -25.000 500 -26.413 3.421 0.413

YFL005W-YFL004W_146_330 146 185 -26.100 500 -26.045 3.320 -0.016

YFL005W-YFL004W_151_335 151 185 -24.600 500 -27.396 3.375 0.829

YFL005W-YFL004W_156_340 156 185 -23.800 500 -28.164 3.339 1.307

YFL005W-YFL004W_161_345 161 185 -21.900 500 -29.635 3.430 2.255

YFL005W-YFL004W_166_350 166 185 -27.600 500 -31.313 3.436 1.080

YFL005W-YFL004W_171_355 171 185 -30.600 500 -32.130 3.688 0.415

YFL005W-YFL004W_176_360 176 185 -31.600 500 -32.784 3.493 0.339

YFL005W-YFL004W_181_365 181 185 -29.700 500 -32.021 3.389 0.685

YFL005W-YFL004W_186_370 186 185 -31.500 500 -31.452 3.370 -0.014

YFL005W-YFL004W_191_375 191 185 -31.500 500 -31.476 3.681 -0.007

YFL005W-YFL004W_196_380 196 185 -31.500 500 -31.431 3.358 -0.020

YFL005W-YFL004W_201_385 201 185 -31.500 500 -32.753 3.575 0.351

YFL005W-YFL004W_206_390 206 185 -32.900 500 -33.347 3.648 0.123

YFL005W-YFL004W_211_395 211 185 -36.700 500 -34.006 3.549 -0.759

YFL005W-YFL004W_216_400 216 185 -36.800 500 -33.758 3.707 -0.821

YFL005W-YFL004W_221_405 221 185 -36.800 500 -34.522 3.426 -0.665

YFL005W-YFL004W_226_410 226 185 -36.700 500 -34.454 3.492 -0.643

YFL005W-YFL004W_231_415 231 185 -37.500 500 -34.682 3.464 -0.813

YFL005W-YFL004W_236_420 236 185 -43.000 500 -34.273 3.605 -2.421

YFL005W-YFL004W_241_425 241 185 -43.100 500 -36.343 3.702 -1.825

YFL005W-YFL004W_246_430 246 185 -42.000 500 -35.433 3.683 -1.783

YFL005W-YFL004W_251_435 251 185 -42.000 500 -35.446 3.623 -1.809

YFL005W-YFL004W_256_440 256 185 -42.300 500 -35.146 3.765 -1.900

YFL005W-YFL004W_261_445 261 185 -43.100 500 -34.746 3.577 -2.336

YFL005W-YFL004W_266_450 266 185 -43.500 500 -32.631 3.359 -3.236

YFL005W-YFL004W_271_455 271 185 -42.900 500 -32.334 3.581 -2.950

YFL005W-YFL004W_276_460 276 185 -41.400 500 -32.814 3.586 -2.395

YFL005W-YFL004W_281_465 281 185 -41.100 500 -32.244 3.530 -2.509

YFL005W-YFL004W_286_470 286 185 -36.600 500 -31.137 3.550 -1.539

YFL005W-YFL004W_291_475 291 185 -38.900 500 -31.633 3.740 -1.943

YFL005W-YFL004W_296_480 296 185 -39.520 500 -33.109 3.515 -1.824

YFL005W-YFL004W_301_485 301 185 -39.520 500 -32.661 3.378 -2.030

YFL005W-YFL004W_306_490 306 185 -37.700 500 -34.525 3.562 -0.891

YFL005W-YFL004W_311_495 311 185 -41.200 500 -34.581 3.602 -1.838

YFL005W-YFL004W_316_500 316 185 -40.900 500 -34.313 3.530 -1.866

YFL005W-YFL004W_321_505 321 185 -37.050 500 -32.550 3.529 -1.275

YFL005W-YFL004W_326_510 326 185 -35.800 500 -31.119 3.428 -1.366

YFL005W-YFL004W_331_515 331 185 -35.500 500 -30.497 3.563 -1.404

YFL005W-YFL004W_336_520 336 185 -31.400 500 -28.288 3.451 -0.902

YFL005W-YFL004W_341_525 341 185 -27.000 500 -29.196 3.508 0.626

YFL005W-YFL004W_346_530 346 185 -28.000 500 -30.131 3.288 0.648

YFL005W-YFL004W_351_535 351 185 -29.700 500 -29.161 3.472 -0.155

YFL005W-YFL004W_356_540 356 185 -29.700 500 -29.954 3.366 0.076

YFL005W-YFL004W_361_545 361 185 -27.800 500 -31.463 3.485 1.051

YFL005W-YFL004W_366_550 366 185 -30.900 500 -32.169 3.646 0.348

YFL005W-YFL004W_371_555 371 185 -31.500 500 -33.489 3.462 0.575

YFL005W-YFL004W_376_560 376 185 -30.500 500 -35.033 3.526 1.286

YFL005W-YFL004W_381_565 381 185 -34.600 500 -35.817 3.482 0.350

YFL005W-YFL004W_386_570 386 185 -34.600 500 -36.078 3.584 0.412

YFL005W-YFL004W_391_575 391 185 -31.400 500 -35.411 3.607 1.112

YFL005W-YFL004W_396_580 396 185 -32.000 500 -36.418 3.676 1.202

YFL005W-YFL004W_401_585 401 185 -34.600 500 -36.824 3.617 0.615

YFL005W-YFL004W_406_590 406 185 -34.500 500 -37.527 3.903 0.776

YFL005W-YFL004W_411_595 411 185 -34.700 500 -37.973 3.648 0.897

YFL005W-YFL004W_416_600 416 185 -36.600 500 -39.963 3.747 0.898

YFL005W-YFL004W_421_605 421 185 -36.300 500 -38.887 3.734 0.693

YFL005W-YFL004W_426_610 426 185 -39.600 500 -38.783 3.649 -0.224

YFL005W-YFL004W_431_615 431 185 -40.300 500 -39.641 3.669 -0.180

YFL005W-YFL004W_436_620 436 185 -42.600 500 -37.969 3.599 -1.287

YFL005W-YFL004W_441_625 441 185 -42.000 500 -38.227 3.751 -1.006

YFL005W-YFL004W_446_630 446 185 -43.000 500 -38.853 3.413 -1.215

YFL005W-YFL004W_451_635 451 185 -44.100 500 -39.603 3.563 -1.262

YFL005W-YFL004W_456_640 456 185 -44.100 500 -39.794 3.795 -1.135

YFL005W-YFL004W_461_645 461 185 -44.100 500 -39.701 3.619 -1.216

YFL005W-YFL004W_466_650 466 185 -44.100 500 -39.267 3.893 -1.241

YFL005W-YFL004W_471_655 471 185 -41.400 500 -38.304 3.607 -0.858

YFL005W-YFL004W_476_660 476 185 -38.900 500 -36.101 3.526 -0.794

YFL005W-YFL004W_481_665 481 185 -39.530 500 -36.383 3.521 -0.894

YFL005W-YFL004W_486_670 486 185 -37.730 500 -34.007 3.713 -1.003

YFL005W-YFL004W_491_675 491 185 -36.800 500 -32.314 3.464 -1.295

YFL005W-YFL004W_496_680 496 185 -36.800 500 -33.374 3.454 -0.992

YFL005W-YFL004W_501_685 501 185 -38.800 500 -35.088 3.604 -1.030

YFL005W-YFL004W_506_690 506 185 -39.680 500 -37.719 3.860 -0.508

YFL005W-YFL004W_511_695 511 185 -39.680 500 -37.521 3.875 -0.557

YFL005W-YFL004W_516_700 516 185 -39.680 500 -38.962 3.671 -0.195

YFL005W-YFL004W_521_705 521 185 -39.480 500 -38.604 3.549 -0.247

YFL005W-YFL004W_526_710 526 185 -38.630 500 -36.701 3.773 -0.511

YFL005W-YFL004W_531_715 531 185 -36.500 500 -34.488 3.568 -0.564

YFL005W-YFL004W_536_720 536 185 -38.030 500 -36.841 3.736 -0.318

YFL005W-YFL004W_541_725 541 185 -36.930 500 -36.587 3.500 -0.098

YFL005W-YFL004W_546_730 546 185 -41.030 500 -37.493 3.661 -0.966

YFL005W-YFL004W_551_735 551 185 -37.680 500 -37.049 3.662 -0.172

YFL005W-YFL004W_556_740 556 185 -39.600 500 -38.675 3.782 -0.245

YFL005W-YFL004W_561_745 561 185 -41.000 500 -40.377 3.860 -0.161

YFL005W-YFL004W_566_750 566 185 -41.200 500 -38.806 3.926 -0.610

YFL005W-YFL004W_571_755 571 185 -35.000 500 -39.212 3.934 1.071

YFL005W-YFL004W_576_760 576 185 -33.200 500 -37.582 3.596 1.218

YFL005W-YFL004W_581_765 581 185 -32.400 500 -37.782 4.034 1.334

YFL005W-YFL004W_586_770 586 185 -33.850 500 -38.583 3.957 1.196

YFL005W-YFL004W_591_775 591 185 -37.900 500 -38.185 4.023 0.071

YFL005W-YFL004W_596_780 596 185 -34.030 500 -37.586 3.617 0.983

YFL005W-YFL004W_601_785 601 185 -36.430 500 -35.664 3.759 -0.204

YFL005W-YFL004W_606_790 606 185 -36.430 500 -36.714 3.743 0.076

YFL005W-YFL004W_611_795 611 185 -40.400 500 -36.589 3.902 -0.977

YFL005W-YFL004W_616_800 616 185 -42.600 500 -36.246 3.951 -1.608

YFL005W-YFL004W_621_805 621 185 -42.600 500 -35.954 3.574 -1.860

YFL005W-YFL004W_626_810 626 185 -42.600 500 -35.581 3.717 -1.888

YFL005W-YFL004W_631_815 631 185 -38.500 500 -32.855 3.848 -1.467

YFL005W-YFL004W_636_820 636 185 -37.700 500 -33.558 3.762 -1.101

YFL005W-YFL004W_641_825 641 185 -35.000 500 -32.830 3.880 -0.559

YFL005W-YFL004W_645_828 645 183 -35.900 500 -32.299 3.919 -0.919

Window size = 190

YFL005W-YFL004W_1_190 1 190 -27.900 500 -27.136 3.262 -0.234

YFL005W-YFL004W_6_195 6 190 -27.600 500 -25.507 3.171 -0.660

YFL005W-YFL004W_11_200 11 190 -24.700 500 -22.677 3.067 -0.660

YFL005W-YFL004W_16_205 16 190 -25.000 500 -23.519 2.983 -0.497

YFL005W-YFL004W_21_210 21 190 -23.790 500 -22.774 3.024 -0.336

YFL005W-YFL004W_26_215 26 190 -21.450 500 -21.962 3.137 0.163

YFL005W-YFL004W_31_220 31 190 -22.200 500 -20.253 2.889 -0.674

YFL005W-YFL004W_36_225 36 190 -21.900 500 -19.767 2.897 -0.736

YFL005W-YFL004W_41_230 41 190 -18.100 500 -17.682 2.809 -0.149

YFL005W-YFL004W_46_235 46 190 -15.000 500 -14.912 2.710 -0.033

YFL005W-YFL004W_51_240 51 190 -15.000 500 -13.998 2.491 -0.402

YFL005W-YFL004W_56_245 56 190 -15.000 500 -12.627 2.455 -0.967

YFL005W-YFL004W_61_250 61 190 -15.000 500 -13.705 2.620 -0.494

YFL005W-YFL004W_66_255 66 190 -13.000 500 -13.117 2.483 0.047

YFL005W-YFL004W_71_260 71 190 -16.900 500 -13.161 2.721 -1.374

YFL005W-YFL004W_76_265 76 190 -17.700 500 -16.899 2.820 -0.284

YFL005W-YFL004W_81_270 81 190 -19.000 500 -19.280 2.874 0.097

YFL005W-YFL004W_86_275 86 190 -19.000 500 -19.693 2.948 0.235

YFL005W-YFL004W_91_280 91 190 -19.000 500 -21.028 2.885 0.703

YFL005W-YFL004W_96_285 96 190 -19.900 500 -22.036 3.041 0.703

YFL005W-YFL004W_101_290 101 190 -20.040 500 -21.976 2.968 0.652

YFL005W-YFL004W_106_295 106 190 -20.500 500 -22.212 3.013 0.568

YFL005W-YFL004W_111_300 111 190 -22.900 500 -24.423 3.159 0.482

YFL005W-YFL004W_116_305 116 190 -25.000 500 -25.999 3.408 0.293

YFL005W-YFL004W_121_310 121 190 -23.400 500 -25.475 3.285 0.632

YFL005W-YFL004W_126_315 126 190 -23.900 500 -25.022 3.034 0.370

YFL005W-YFL004W_131_320 131 190 -24.600 500 -25.187 3.072 0.191

YFL005W-YFL004W_136_325 136 190 -25.000 500 -26.252 3.250 0.385

YFL005W-YFL004W_141_330 141 190 -26.100 500 -27.536 3.104 0.463

YFL005W-YFL004W_146_335 146 190 -26.100 500 -28.258 3.536 0.610

YFL005W-YFL004W_151_340 151 190 -26.600 500 -28.934 3.375 0.692

YFL005W-YFL004W_156_345 156 190 -23.800 500 -30.092 3.345 1.881

YFL005W-YFL004W_161_350 161 190 -27.600 500 -31.260 3.404 1.075

YFL005W-YFL004W_166_355 166 190 -30.600 500 -33.582 3.642 0.819

YFL005W-YFL004W_171_360 171 190 -31.900 500 -32.947 3.504 0.299

YFL005W-YFL004W_176_365 176 190 -33.200 500 -33.551 3.581 0.098

YFL005W-YFL004W_181_370 181 190 -31.500 500 -32.572 3.649 0.294

YFL005W-YFL004W_186_375 186 190 -31.500 500 -31.332 3.521 -0.048

YFL005W-YFL004W_191_380 191 190 -31.500 500 -31.605 3.609 0.029

YFL005W-YFL004W_196_385 196 190 -31.500 500 -32.991 3.756 0.397

YFL005W-YFL004W_201_390 201 190 -32.900 500 -33.433 3.544 0.150

YFL005W-YFL004W_206_395 206 190 -36.700 500 -34.326 3.585 -0.662

YFL005W-YFL004W_211_400 211 190 -36.800 500 -34.851 3.612 -0.540

YFL005W-YFL004W_216_405 216 190 -36.800 500 -34.696 3.527 -0.597

YFL005W-YFL004W_221_410 221 190 -36.800 500 -35.142 3.689 -0.449

YFL005W-YFL004W_226_415 226 190 -37.500 500 -35.538 3.710 -0.529

YFL005W-YFL004W_231_420 231 190 -43.000 500 -35.586 3.821 -1.940

YFL005W-YFL004W_236_425 236 190 -45.900 500 -37.010 3.722 -2.388

YFL005W-YFL004W_241_430 241 190 -43.100 500 -37.114 3.761 -1.591

YFL005W-YFL004W_246_435 246 190 -42.000 500 -36.543 3.741 -1.459

YFL005W-YFL004W_251_440 251 190 -42.300 500 -36.420 3.486 -1.687

YFL005W-YFL004W_256_445 256 190 -43.630 500 -36.637 3.608 -1.938

YFL005W-YFL004W_261_450 261 190 -45.400 500 -36.204 3.872 -2.375

YFL005W-YFL004W_266_455 266 190 -43.500 500 -32.959 3.619 -2.912

YFL005W-YFL004W_271_460 271 190 -42.900 500 -32.933 3.451 -2.888

YFL005W-YFL004W_276_465 276 190 -42.800 500 -34.794 3.782 -2.117

YFL005W-YFL004W_281_470 281 190 -41.700 500 -33.292 3.621 -2.322

YFL005W-YFL004W_286_475 286 190 -38.900 500 -32.175 3.721 -1.807

YFL005W-YFL004W_291_480 291 190 -39.520 500 -33.271 3.431 -1.821

YFL005W-YFL004W_296_485 296 190 -39.520 500 -34.615 3.614 -1.357

YFL005W-YFL004W_301_490 301 190 -39.520 500 -35.785 3.581 -1.043

YFL005W-YFL004W_306_495 306 190 -41.200 500 -34.993 3.511 -1.768

YFL005W-YFL004W_311_500 311 190 -41.200 500 -34.718 3.554 -1.824

YFL005W-YFL004W_316_505 316 190 -40.900 500 -34.251 3.540 -1.878

YFL005W-YFL004W_321_510 321 190 -37.050 500 -33.745 3.210 -1.030

YFL005W-YFL004W_326_515 326 190 -35.800 500 -31.301 3.564 -1.262

YFL005W-YFL004W_331_520 331 190 -35.500 500 -31.360 3.608 -1.147

YFL005W-YFL004W_336_525 336 190 -34.100 500 -29.474 3.379 -1.369

YFL005W-YFL004W_341_530 341 190 -29.700 500 -30.707 3.509 0.287

YFL005W-YFL004W_346_535 346 190 -31.100 500 -32.411 3.656 0.359

YFL005W-YFL004W_351_540 351 190 -31.400 500 -30.749 3.512 -0.185

YFL005W-YFL004W_356_545 356 190 -29.700 500 -31.475 3.420 0.519

YFL005W-YFL004W_361_550 361 190 -31.300 500 -33.474 3.474 0.626

YFL005W-YFL004W_366_555 366 190 -31.500 500 -33.966 3.697 0.667

YFL005W-YFL004W_371_560 371 190 -31.500 500 -35.109 3.469 1.040

YFL005W-YFL004W_376_565 376 190 -34.600 500 -36.075 3.591 0.411

YFL005W-YFL004W_381_570 381 190 -34.600 500 -37.109 3.641 0.689

YFL005W-YFL004W_386_575 386 190 -34.600 500 -37.040 3.795 0.643

YFL005W-YFL004W_391_580 391 190 -32.000 500 -37.319 3.548 1.499

YFL005W-YFL004W_396_585 396 190 -37.000 500 -37.180 3.498 0.051

YFL005W-YFL004W_401_590 401 190 -34.800 500 -38.575 3.802 0.993

YFL005W-YFL004W_406_595 406 190 -34.700 500 -38.269 3.837 0.930

YFL005W-YFL004W_411_600 411 190 -36.600 500 -40.581 3.914 1.017

YFL005W-YFL004W_416_605 416 190 -36.900 500 -41.045 3.665 1.131

YFL005W-YFL004W_421_610 421 190 -40.400 500 -40.470 3.704 0.019

YFL005W-YFL004W_426_615 426 190 -40.300 500 -40.696 3.811 0.104

YFL005W-YFL004W_431_620 431 190 -43.700 500 -39.658 3.642 -1.110

YFL005W-YFL004W_436_625 436 190 -43.400 500 -38.952 3.664 -1.214

YFL005W-YFL004W_441_630 441 190 -43.500 500 -39.797 3.858 -0.960

YFL005W-YFL004W_446_635 446 190 -44.100 500 -40.729 4.058 -0.831

YFL005W-YFL004W_451_640 451 190 -44.100 500 -39.965 3.514 -1.177

YFL005W-YFL004W_456_645 456 190 -44.100 500 -40.281 3.903 -0.978

YFL005W-YFL004W_461_650 461 190 -44.100 500 -41.740 3.931 -0.600

YFL005W-YFL004W_466_655 466 190 -44.100 500 -39.173 3.790 -1.300

YFL005W-YFL004W_471_660 471 190 -41.400 500 -38.931 3.612 -0.684

YFL005W-YFL004W_476_665 476 190 -39.530 500 -36.671 3.668 -0.779

YFL005W-YFL004W_481_670 481 190 -39.530 500 -36.320 3.859 -0.832

YFL005W-YFL004W_486_675 486 190 -37.790 500 -34.556 3.659 -0.884

YFL005W-YFL004W_491_680 491 190 -36.800 500 -33.135 3.583 -1.023

YFL005W-YFL004W_496_685 496 190 -38.800 500 -35.429 3.747 -0.900

YFL005W-YFL004W_501_690 501 190 -39.680 500 -38.132 3.828 -0.404

YFL005W-YFL004W_506_695 506 190 -39.680 500 -38.155 3.830 -0.398

YFL005W-YFL004W_511_700 511 190 -39.680 500 -38.880 3.557 -0.225

YFL005W-YFL004W_516_705 516 190 -39.680 500 -39.185 3.797 -0.130

YFL005W-YFL004W_521_710 521 190 -39.480 500 -38.719 3.648 -0.209

YFL005W-YFL004W_526_715 526 190 -39.500 500 -37.308 3.760 -0.583

YFL005W-YFL004W_531_720 531 190 -38.030 500 -38.011 3.954 -0.005

YFL005W-YFL004W_536_725 536 190 -38.830 500 -37.639 3.439 -0.346

YFL005W-YFL004W_541_730 541 190 -41.030 500 -38.571 3.712 -0.662

YFL005W-YFL004W_546_735 546 190 -41.030 500 -40.363 3.880 -0.172

YFL005W-YFL004W_551_740 551 190 -40.300 500 -39.804 3.856 -0.129

YFL005W-YFL004W_556_745 556 190 -41.000 500 -40.704 3.905 -0.076

YFL005W-YFL004W_561_750 561 190 -41.200 500 -40.579 3.983 -0.156

YFL005W-YFL004W_566_755 566 190 -41.200 500 -40.044 3.655 -0.316

YFL005W-YFL004W_571_760 571 190 -35.000 500 -38.844 3.802 1.011

YFL005W-YFL004W_576_765 576 190 -33.800 500 -40.159 3.839 1.657

YFL005W-YFL004W_581_770 581 190 -33.850 500 -38.457 3.535 1.303

YFL005W-YFL004W_586_775 586 190 -38.950 500 -39.654 3.853 0.183

YFL005W-YFL004W_591_780 591 190 -37.900 500 -39.734 3.826 0.479

YFL005W-YFL004W_596_785 596 190 -36.430 500 -37.292 3.979 0.217

YFL005W-YFL004W_601_790 601 190 -37.300 500 -37.403 3.719 0.028

YFL005W-YFL004W_606_795 606 190 -40.500 500 -37.077 3.976 -0.861

YFL005W-YFL004W_611_800 611 190 -44.000 500 -38.222 3.796 -1.522

YFL005W-YFL004W_616_805 616 190 -42.600 500 -36.317 3.550 -1.770

YFL005W-YFL004W_621_810 621 190 -42.600 500 -35.922 3.686 -1.812

YFL005W-YFL004W_626_815 626 190 -42.600 500 -35.435 3.941 -1.818

YFL005W-YFL004W_631_820 631 190 -38.500 500 -33.733 3.575 -1.334

YFL005W-YFL004W_636_825 636 190 -37.700 500 -33.730 3.752 -1.058

YFL005W-YFL004W_640_828 640 188 -35.900 500 -33.493 3.848 -0.626

Window size = 195

YFL005W-YFL004W_1_195 1 195 -28.800 500 -27.441 3.203 -0.424

YFL005W-YFL004W_6_200 6 195 -27.600 500 -25.675 3.071 -0.627

YFL005W-YFL004W_11_205 11 195 -26.100 500 -23.142 3.045 -0.972

YFL005W-YFL004W_16_210 16 195 -25.190 500 -23.963 3.051 -0.402

YFL005W-YFL004W_21_215 21 195 -25.000 500 -23.149 3.163 -0.585

YFL005W-YFL004W_26_220 26 195 -22.200 500 -22.086 2.935 -0.039

YFL005W-YFL004W_31_225 31 195 -22.200 500 -20.231 2.983 -0.660

YFL005W-YFL004W_36_230 36 195 -21.900 500 -19.748 3.097 -0.695

YFL005W-YFL004W_41_235 41 195 -18.900 500 -18.245 2.699 -0.243

YFL005W-YFL004W_46_240 46 195 -15.000 500 -15.842 2.626 0.320

YFL005W-YFL004W_51_245 51 195 -15.000 500 -14.113 2.657 -0.334

YFL005W-YFL004W_56_250 56 195 -15.000 500 -14.289 2.666 -0.267

YFL005W-YFL004W_61_255 61 195 -15.000 500 -14.246 2.711 -0.278

YFL005W-YFL004W_66_260 66 195 -16.900 500 -14.195 2.709 -0.999

YFL005W-YFL004W_71_265 71 195 -17.700 500 -16.624 2.805 -0.383

YFL005W-YFL004W_76_270 76 195 -19.000 500 -19.237 2.884 0.082

YFL005W-YFL004W_81_275 81 195 -19.000 500 -19.327 2.900 0.113

YFL005W-YFL004W_86_280 86 195 -19.000 500 -21.342 3.149 0.744

YFL005W-YFL004W_91_285 91 195 -22.500 500 -22.334 2.980 -0.056

YFL005W-YFL004W_96_290 96 195 -21.590 500 -24.255 3.094 0.861

YFL005W-YFL004W_101_295 101 195 -20.500 500 -22.300 2.982 0.604

YFL005W-YFL004W_106_300 106 195 -22.900 500 -24.621 3.446 0.499

YFL005W-YFL004W_111_305 111 195 -25.000 500 -26.235 3.012 0.410

YFL005W-YFL004W_116_310 116 195 -25.000 500 -25.797 3.353 0.238

YFL005W-YFL004W_121_315 121 195 -24.200 500 -26.153 3.281 0.595

YFL005W-YFL004W_126_320 126 195 -27.200 500 -27.101 3.094 -0.032

YFL005W-YFL004W_131_325 131 195 -25.000 500 -26.684 3.406 0.495

YFL005W-YFL004W_136_330 136 195 -26.100 500 -27.915 3.568 0.509

YFL005W-YFL004W_141_335 141 195 -26.100 500 -29.564 3.383 1.024

YFL005W-YFL004W_146_340 146 195 -26.600 500 -29.123 3.282 0.769

YFL005W-YFL004W_151_345 151 195 -26.600 500 -30.510 3.635 1.076

YFL005W-YFL004W_156_350 156 195 -31.100 500 -31.564 3.538 0.131

YFL005W-YFL004W_161_355 161 195 -30.600 500 -33.285 3.598 0.746

YFL005W-YFL004W_166_360 166 195 -31.900 500 -34.121 3.532 0.629

YFL005W-YFL004W_171_365 171 195 -33.500 500 -33.348 3.538 -0.043

YFL005W-YFL004W_176_370 176 195 -35.000 500 -34.262 3.405 -0.217

YFL005W-YFL004W_181_375 181 195 -31.500 500 -32.861 3.557 0.383

YFL005W-YFL004W_186_380 186 195 -31.500 500 -32.319 3.546 0.231

YFL005W-YFL004W_191_385 191 195 -31.500 500 -33.070 3.513 0.447

YFL005W-YFL004W_196_390 196 195 -32.900 500 -33.394 3.501 0.141

YFL005W-YFL004W_201_395 201 195 -36.700 500 -34.291 3.568 -0.675

YFL005W-YFL004W_206_400 206 195 -36.800 500 -34.730 3.604 -0.574

YFL005W-YFL004W_211_405 211 195 -36.800 500 -35.424 3.514 -0.392

YFL005W-YFL004W_216_410 216 195 -36.800 500 -35.579 3.682 -0.332

YFL005W-YFL004W_221_415 221 195 -37.500 500 -36.291 3.824 -0.316

YFL005W-YFL004W_226_420 226 195 -43.000 500 -35.765 3.674 -1.970

YFL005W-YFL004W_231_425 231 195 -45.900 500 -37.918 3.646 -2.189

YFL005W-YFL004W_236_430 236 195 -46.600 500 -37.826 3.709 -2.365

YFL005W-YFL004W_241_435 241 195 -43.100 500 -38.030 3.887 -1.304

YFL005W-YFL004W_246_440 246 195 -42.300 500 -38.106 3.651 -1.149

YFL005W-YFL004W_251_445 251 195 -43.700 500 -37.987 3.849 -1.484

YFL005W-YFL004W_256_450 256 195 -45.400 500 -37.742 3.642 -2.103

YFL005W-YFL004W_261_455 261 195 -47.200 500 -36.459 3.909 -2.748

YFL005W-YFL004W_266_460 266 195 -43.500 500 -33.562 3.634 -2.735

YFL005W-YFL004W_271_465 271 195 -42.900 500 -34.773 4.013 -2.025

YFL005W-YFL004W_276_470 276 195 -46.000 500 -35.899 3.811 -2.651

YFL005W-YFL004W_281_475 281 195 -44.500 500 -34.779 3.589 -2.709

YFL005W-YFL004W_286_480 286 195 -39.520 500 -34.187 3.795 -1.405

YFL005W-YFL004W_291_485 291 195 -39.520 500 -34.539 3.609 -1.380

YFL005W-YFL004W_296_490 296 195 -39.520 500 -37.401 3.704 -0.572

YFL005W-YFL004W_301_495 301 195 -41.200 500 -36.491 3.668 -1.284

YFL005W-YFL004W_306_500 306 195 -41.200 500 -35.537 3.517 -1.610

YFL005W-YFL004W_311_505 311 195 -41.200 500 -35.242 3.651 -1.632

YFL005W-YFL004W_316_510 316 195 -40.900 500 -35.644 3.694 -1.423

YFL005W-YFL004W_321_515 321 195 -37.050 500 -34.143 3.609 -0.805

YFL005W-YFL004W_326_520 326 195 -35.800 500 -31.709 3.540 -1.156

YFL005W-YFL004W_331_525 331 195 -35.800 500 -32.933 3.451 -0.831

YFL005W-YFL004W_336_530 336 195 -36.900 500 -31.415 3.266 -1.680

YFL005W-YFL004W_341_535 341 195 -32.700 500 -32.975 3.614 0.076

YFL005W-YFL004W_346_540 346 195 -31.400 500 -33.875 3.695 0.670

YFL005W-YFL004W_351_545 351 195 -32.100 500 -32.104 3.508 0.001

YFL005W-YFL004W_356_550 356 195 -33.200 500 -33.665 3.467 0.134

YFL005W-YFL004W_361_555 361 195 -31.950 500 -35.156 3.514 0.912

YFL005W-YFL004W_366_560 366 195 -31.500 500 -35.281 3.484 1.085

YFL005W-YFL004W_371_565 371 195 -34.600 500 -36.108 3.598 0.419

YFL005W-YFL004W_376_570 376 195 -35.800 500 -37.458 3.686 0.450

YFL005W-YFL004W_381_575 381 195 -34.600 500 -38.500 3.496 1.116

YFL005W-YFL004W_386_580 386 195 -34.600 500 -39.112 3.560 1.267

YFL005W-YFL004W_391_585 391 195 -37.000 500 -37.734 3.669 0.200

YFL005W-YFL004W_396_590 396 195 -37.000 500 -38.631 3.755 0.435

YFL005W-YFL004W_401_595 401 195 -34.800 500 -39.361 3.666 1.244

YFL005W-YFL004W_406_600 406 195 -38.400 500 -41.518 4.003 0.779

YFL005W-YFL004W_411_605 411 195 -37.200 500 -41.403 3.741 1.124

YFL005W-YFL004W_416_610 416 195 -42.200 500 -41.900 3.666 -0.082

YFL005W-YFL004W_421_615 421 195 -41.100 500 -42.870 3.730 0.475

YFL005W-YFL004W_426_620 426 195 -44.300 500 -40.746 3.531 -1.007

YFL005W-YFL004W_431_625 431 195 -44.500 500 -40.163 3.856 -1.125

YFL005W-YFL004W_436_630 436 195 -44.900 500 -40.458 3.959 -1.122

YFL005W-YFL004W_441_635 441 195 -44.600 500 -40.811 3.816 -0.993

YFL005W-YFL004W_446_640 446 195 -44.100 500 -40.496 3.607 -0.999

YFL005W-YFL004W_451_645 451 195 -44.100 500 -40.762 3.738 -0.893

YFL005W-YFL004W_456_650 456 195 -44.100 500 -42.118 3.724 -0.532

YFL005W-YFL004W_461_655 461 195 -44.700 500 -41.943 3.854 -0.716

YFL005W-YFL004W_466_660 466 195 -44.100 500 -39.932 3.749 -1.112

YFL005W-YFL004W_471_665 471 195 -41.400 500 -39.276 3.788 -0.561

YFL005W-YFL004W_476_670 476 195 -39.530 500 -36.839 3.708 -0.726

YFL005W-YFL004W_481_675 481 195 -39.890 500 -37.177 3.673 -0.739

YFL005W-YFL004W_486_680 486 195 -37.790 500 -35.208 3.649 -0.708

YFL005W-YFL004W_491_685 491 195 -38.800 500 -35.781 3.550 -0.850

YFL005W-YFL004W_496_690 496 195 -41.100 500 -38.592 3.656 -0.686

YFL005W-YFL004W_501_695 501 195 -39.680 500 -38.425 3.679 -0.341

YFL005W-YFL004W_506_700 506 195 -39.680 500 -39.154 3.756 -0.140

YFL005W-YFL004W_511_705 511 195 -39.680 500 -39.115 3.644 -0.155

YFL005W-YFL004W_516_710 516 195 -39.680 500 -39.332 3.620 -0.096

YFL005W-YFL004W_521_715 521 195 -39.500 500 -39.603 3.741 0.028

YFL005W-YFL004W_526_720 526 195 -41.900 500 -40.540 3.700 -0.368

YFL005W-YFL004W_531_725 531 195 -39.300 500 -39.268 3.900 -0.008

YFL005W-YFL004W_536_730 536 195 -41.030 500 -39.775 3.545 -0.354

YFL005W-YFL004W_541_735 541 195 -41.300 500 -41.310 3.711 0.003

YFL005W-YFL004W_546_740 546 195 -42.500 500 -43.074 4.072 0.141

YFL005W-YFL004W_551_745 551 195 -41.000 500 -42.066 3.713 0.287

YFL005W-YFL004W_556_750 556 195 -41.200 500 -40.978 3.909 -0.057

YFL005W-YFL004W_561_755 561 195 -41.200 500 -41.968 4.091 0.188

YFL005W-YFL004W_566_760 566 195 -41.200 500 -40.292 3.634 -0.250

YFL005W-YFL004W_571_765 571 195 -36.800 500 -41.393 3.884 1.183

YFL005W-YFL004W_576_770 576 195 -35.800 500 -40.926 3.730 1.374

YFL005W-YFL004W_581_775 581 195 -38.950 500 -39.455 3.839 0.131

YFL005W-YFL004W_586_780 586 195 -38.950 500 -41.106 3.722 0.579

YFL005W-YFL004W_591_785 591 195 -37.900 500 -39.640 3.807 0.457

YFL005W-YFL004W_596_790 596 195 -37.300 500 -39.404 3.855 0.546

YFL005W-YFL004W_601_795 601 195 -40.500 500 -37.998 3.862 -0.648

YFL005W-YFL004W_606_800 606 195 -44.600 500 -38.297 3.689 -1.708

YFL005W-YFL004W_611_805 611 195 -44.500 500 -38.506 3.848 -1.558

YFL005W-YFL004W_616_810 616 195 -42.600 500 -36.452 3.771 -1.630

YFL005W-YFL004W_621_815 621 195 -42.600 500 -35.766 3.773 -1.811

YFL005W-YFL004W_626_820 626 195 -42.600 500 -36.402 3.657 -1.695

YFL005W-YFL004W_631_825 631 195 -38.500 500 -34.354 3.658 -1.134

YFL005W-YFL004W_635_828 635 193 -37.700 500 -35.207 3.502 -0.712

Window size = 200

YFL005W-YFL004W_1_200 1 200 -28.800 500 -28.203 3.318 -0.180

YFL005W-YFL004W_6_205 6 200 -29.000 500 -26.347 3.334 -0.796

YFL005W-YFL004W_11_210 11 200 -26.290 500 -23.678 3.036 -0.860

YFL005W-YFL004W_16_215 16 200 -26.400 500 -24.018 3.207 -0.743

YFL005W-YFL004W_21_220 21 200 -25.050 500 -23.339 3.172 -0.539

YFL005W-YFL004W_26_225 26 200 -22.200 500 -22.085 2.996 -0.038

YFL005W-YFL004W_31_230 31 200 -22.200 500 -20.102 3.000 -0.699

YFL005W-YFL004W_36_235 36 200 -21.900 500 -20.081 2.876 -0.632

YFL005W-YFL004W_41_240 41 200 -18.900 500 -18.747 2.943 -0.052

YFL005W-YFL004W_46_245 46 200 -15.800 500 -15.913 2.759 0.041

YFL005W-YFL004W_51_250 51 200 -15.000 500 -15.706 2.831 0.249

YFL005W-YFL004W_56_255 56 200 -15.000 500 -14.841 2.753 -0.058

YFL005W-YFL004W_61_260 61 200 -16.900 500 -15.345 2.803 -0.555

YFL005W-YFL004W_66_265 66 200 -17.700 500 -17.314 2.891 -0.134

YFL005W-YFL004W_71_270 71 200 -19.000 500 -19.013 2.864 0.005

YFL005W-YFL004W_76_275 76 200 -19.000 500 -19.398 3.117 0.128

YFL005W-YFL004W_81_280 81 200 -19.000 500 -21.302 3.046 0.756

YFL005W-YFL004W_86_285 86 200 -22.500 500 -22.863 3.077 0.118

YFL005W-YFL004W_91_290 91 200 -22.500 500 -24.969 3.268 0.755

YFL005W-YFL004W_96_295 96 200 -22.590 500 -24.435 2.961 0.623

YFL005W-YFL004W_101_300 101 200 -22.900 500 -24.380 3.173 0.466

YFL005W-YFL004W_106_305 106 200 -25.000 500 -26.404 3.249 0.432

YFL005W-YFL004W_111_310 111 200 -25.000 500 -26.528 3.160 0.483

YFL005W-YFL004W_116_315 116 200 -25.000 500 -26.439 3.210 0.448

YFL005W-YFL004W_121_320 121 200 -27.500 500 -27.592 3.122 0.029

YFL005W-YFL004W_126_325 126 200 -28.600 500 -28.448 3.357 -0.045

YFL005W-YFL004W_131_330 131 200 -26.100 500 -28.283 3.297 0.662

YFL005W-YFL004W_136_335 136 200 -27.300 500 -29.928 3.403 0.772

YFL005W-YFL004W_141_340 141 200 -26.600 500 -30.713 3.451 1.192

YFL005W-YFL004W_146_345 146 200 -26.800 500 -31.056 3.515 1.211

YFL005W-YFL004W_151_350 151 200 -33.900 500 -32.149 3.546 -0.494

YFL005W-YFL004W_156_355 156 200 -33.000 500 -34.323 3.642 0.363

YFL005W-YFL004W_161_360 161 200 -31.900 500 -34.023 3.571 0.594

YFL005W-YFL004W_166_365 166 200 -33.500 500 -34.793 3.620 0.357

YFL005W-YFL004W_171_370 171 200 -35.300 500 -34.661 3.551 -0.180

YFL005W-YFL004W_176_375 176 200 -35.000 500 -34.487 3.397 -0.151

YFL005W-YFL004W_181_380 181 200 -31.500 500 -33.368 3.419 0.546

YFL005W-YFL004W_186_385 186 200 -31.500 500 -33.603 3.696 0.569

YFL005W-YFL004W_191_390 191 200 -32.900 500 -33.740 3.651 0.230

YFL005W-YFL004W_196_395 196 200 -36.700 500 -34.582 3.808 -0.556

YFL005W-YFL004W_201_400 201 200 -36.800 500 -34.493 3.369 -0.685

YFL005W-YFL004W_206_405 206 200 -36.800 500 -35.561 3.688 -0.336

YFL005W-YFL004W_211_410 211 200 -36.800 500 -35.342 3.496 -0.417

YFL005W-YFL004W_216_415 216 200 -37.500 500 -36.291 3.733 -0.324

YFL005W-YFL004W_221_420 221 200 -43.000 500 -36.520 3.584 -1.808

YFL005W-YFL004W_226_425 226 200 -45.900 500 -38.278 3.714 -2.052

YFL005W-YFL004W_231_430 231 200 -46.600 500 -38.795 3.907 -1.998

YFL005W-YFL004W_236_435 236 200 -46.600 500 -38.714 3.756 -2.100

YFL005W-YFL004W_241_440 241 200 -43.100 500 -39.052 3.764 -1.075

YFL005W-YFL004W_246_445 246 200 -43.700 500 -38.734 3.502 -1.418

YFL005W-YFL004W_251_450 251 200 -45.400 500 -39.105 3.877 -1.624

YFL005W-YFL004W_256_455 256 200 -48.000 500 -38.042 3.913 -2.545

YFL005W-YFL004W_261_460 261 200 -47.200 500 -37.092 3.756 -2.691

YFL005W-YFL004W_266_465 266 200 -43.500 500 -35.392 3.529 -2.298

YFL005W-YFL004W_271_470 271 200 -46.300 500 -35.980 3.554 -2.904

YFL005W-YFL004W_276_475 276 200 -46.000 500 -37.064 3.869 -2.310

YFL005W-YFL004W_281_480 281 200 -44.500 500 -36.449 3.822 -2.106

YFL005W-YFL004W_286_485 286 200 -39.520 500 -35.155 3.803 -1.148

YFL005W-YFL004W_291_490 291 200 -41.900 500 -37.258 3.774 -1.230

YFL005W-YFL004W_296_495 296 200 -41.200 500 -37.569 3.845 -0.944

YFL005W-YFL004W_301_500 301 200 -41.200 500 -36.524 3.764 -1.242

YFL005W-YFL004W_306_505 306 200 -41.200 500 -35.514 3.357 -1.694

YFL005W-YFL004W_311_510 311 200 -41.200 500 -36.225 3.744 -1.329

YFL005W-YFL004W_316_515 316 200 -40.900 500 -35.814 3.725 -1.365

YFL005W-YFL004W_321_520 321 200 -38.250 500 -34.783 3.837 -0.904

YFL005W-YFL004W_326_525 326 200 -35.800 500 -33.836 3.710 -0.529

YFL005W-YFL004W_331_530 331 200 -38.600 500 -34.875 3.669 -1.015

YFL005W-YFL004W_336_535 336 200 -38.600 500 -33.628 3.453 -1.440

YFL005W-YFL004W_341_540 341 200 -33.400 500 -34.467 3.469 0.308

YFL005W-YFL004W_346_545 346 200 -35.100 500 -35.350 3.577 0.070

YFL005W-YFL004W_351_550 351 200 -34.700 500 -33.870 3.522 -0.236

YFL005W-YFL004W_356_555 356 200 -33.800 500 -34.989 3.386 0.351

YFL005W-YFL004W_361_560 361 200 -32.800 500 -36.781 3.585 1.111

YFL005W-YFL004W_366_565 366 200 -34.600 500 -36.689 3.742 0.558

YFL005W-YFL004W_371_570 371 200 -35.800 500 -37.596 3.474 0.517

YFL005W-YFL004W_376_575 376 200 -35.800 500 -38.900 3.585 0.865

YFL005W-YFL004W_381_580 381 200 -34.600 500 -40.273 3.801 1.492

YFL005W-YFL004W_386_585 386 200 -37.000 500 -39.931 3.703 0.792

YFL005W-YFL004W_391_590 391 200 -37.000 500 -39.181 3.652 0.597

YFL005W-YFL004W_396_595 396 200 -37.000 500 -39.442 3.580 0.682

YFL005W-YFL004W_401_600 401 200 -38.400 500 -42.533 3.791 1.090

YFL005W-YFL004W_406_605 406 200 -38.400 500 -41.590 3.792 0.841

YFL005W-YFL004W_411_610 411 200 -42.400 500 -43.014 3.940 0.156

YFL005W-YFL004W_416_615 416 200 -43.600 500 -44.329 3.771 0.193

YFL005W-YFL004W_421_620 421 200 -47.100 500 -43.021 3.886 -1.050

YFL005W-YFL004W_426_625 426 200 -45.100 500 -41.508 3.982 -0.902

YFL005W-YFL004W_431_630 431 200 -45.800 500 -42.043 3.939 -0.954

YFL005W-YFL004W_436_635 436 200 -46.000 500 -41.637 4.086 -1.068

YFL005W-YFL004W_441_640 441 200 -44.600 500 -41.244 3.793 -0.885

YFL005W-YFL004W_446_645 446 200 -44.100 500 -41.132 3.731 -0.796

YFL005W-YFL004W_451_650 451 200 -44.100 500 -42.231 3.959 -0.472

YFL005W-YFL004W_456_655 456 200 -45.400 500 -42.150 3.780 -0.860

YFL005W-YFL004W_461_660 461 200 -44.700 500 -42.610 3.836 -0.545

YFL005W-YFL004W_466_665 466 200 -44.100 500 -40.386 3.780 -0.982

YFL005W-YFL004W_471_670 471 200 -41.670 500 -39.482 3.581 -0.611

YFL005W-YFL004W_476_675 476 200 -39.890 500 -37.449 3.435 -0.710

YFL005W-YFL004W_481_680 481 200 -39.890 500 -37.687 3.845 -0.573

YFL005W-YFL004W_486_685 486 200 -39.730 500 -37.683 3.557 -0.575

YFL005W-YFL004W_491_690 491 200 -41.300 500 -38.698 3.674 -0.708

YFL005W-YFL004W_496_695 496 200 -41.100 500 -38.879 3.707 -0.599

YFL005W-YFL004W_501_700 501 200 -41.080 500 -39.713 3.746 -0.365

YFL005W-YFL004W_506_705 506 200 -39.900 500 -39.585 3.677 -0.086

YFL005W-YFL004W_511_710 511 200 -39.680 500 -39.693 3.894 0.003

YFL005W-YFL004W_516_715 516 200 -39.880 500 -40.239 3.653 0.098

YFL005W-YFL004W_521_720 521 200 -44.630 500 -42.815 3.568 -0.509

YFL005W-YFL004W_526_725 526 200 -41.900 500 -41.555 3.959 -0.087

YFL005W-YFL004W_531_730 531 200 -41.030 500 -40.696 3.605 -0.093

YFL005W-YFL004W_536_735 536 200 -42.280 500 -42.280 4.020 0.000

YFL005W-YFL004W_541_740 541 200 -43.980 500 -44.028 3.809 0.013

YFL005W-YFL004W_546_745 546 200 -43.300 500 -44.915 3.964 0.408

YFL005W-YFL004W_551_750 551 200 -41.500 500 -42.253 3.858 0.195

YFL005W-YFL004W_556_755 556 200 -41.200 500 -42.269 3.819 0.280

YFL005W-YFL004W_561_760 561 200 -41.200 500 -41.870 3.748 0.179

YFL005W-YFL004W_566_765 566 200 -44.000 500 -42.287 3.766 -0.455

YFL005W-YFL004W_571_770 571 200 -37.100 500 -42.385 3.992 1.324

YFL005W-YFL004W_576_775 576 200 -41.400 500 -42.049 3.710 0.175

YFL005W-YFL004W_581_780 581 200 -38.950 500 -41.714 3.918 0.705

YFL005W-YFL004W_586_785 586 200 -39.330 500 -40.655 3.908 0.339

YFL005W-YFL004W_591_790 591 200 -39.800 500 -41.326 3.724 0.410

YFL005W-YFL004W_596_795 596 200 -40.500 500 -40.205 3.886 -0.076

YFL005W-YFL004W_601_800 601 200 -44.600 500 -39.485 3.645 -1.403

YFL005W-YFL004W_606_805 606 200 -44.600 500 -38.709 3.902 -1.510

YFL005W-YFL004W_611_810 611 200 -44.500 500 -38.317 3.882 -1.593

YFL005W-YFL004W_616_815 616 200 -42.600 500 -36.485 3.930 -1.556

YFL005W-YFL004W_621_820 621 200 -42.600 500 -36.396 3.744 -1.657

YFL005W-YFL004W_626_825 626 200 -42.600 500 -37.185 3.626 -1.494

YFL005W-YFL004W_630_828 630 198 -38.500 500 -35.213 3.644 -0.902
