# Supplementary material for: Non-Coding RNA Prediction and Verification in Saccharomyces cerevisiae
Source: PLoS Genet. 2009 Jan 2;5(1):e1000321. doi: 10.1371/journal.pgen.1000321 (PMC2603021; doi:10.1371/journal.pgen.1000321)
Supplement: Table S8 — The 5′ UTRs mapped by RACE. A “W” means the gene is on the Watson strand and a “C” means the gene is on the Crick strand. The cap for GYP8 was not obtained so the UTR is shown as greater than 249 nt long. (0.10 MB DOC) [file pgen.1000321.s019.doc]

Table S8. The 5' UTRs mapped by RACE. A “W” means the gene is on the Watson strand and a “C” means the gene is on the Crick strand. The cap for GYP8 was not obtained so the UTR is shown as greater than 249nt long.

| **Gene** | **Strand** | **Gene end** | **UTR**  **start** | **ORF**  **start** | **5' UTR length** |
| --- | --- | --- | --- | --- | --- |
| *ACT1* | C | 5' | 54815 | 54695 | 120 |
| *ALR2* | C | 5' | 36598 | 35848 | 750 |
| *DUG1* | C | 5' | 241447 | 241424 | 23 |
| *GSY1* | C | 5' | 176472 | 176383 | 89 |
| *GYP8* | C | 5' | 82159 | 81910 | ≥ 249 |
| *HAC1* | W | 5' | 75111 | 75177 | 66 |
| *IES1* | C | 5' | no data | 109924 | no data |
| *PES4* | W | 5' | 199751 | 199862 | 111 |
| *RIM15* | C | 5' | no data | 74425 | no data |
| *RPL2A* | C | 5' | 221452 | 221406 | 46 |
| *RPO41* | W | 5' | 58565 | 58781 | ≥ 216 |
| *SMC1* | W | 5' | no data | 119424 | no data |
| *STE2* | W | 5' | no data | 82578 | no data |
| *SWP82* | W | 5' | no data | 36803 | no data |
| *VTC2* | W | 5' | 131673 | 131805 | 132 |
| *YFL012W* | W | 5' | 110549 | 110641 | 92 |
| *YFL051C* | C | 5' | 30610 | 30540 | 70 |
| *YFR017C* | C | 5' | 182927 | 182849 | 78 |
| *YFR045W* | W | 5' | 241955 | 241985 | 30 |
