# Supplementary material for: Non-Coding RNA Prediction and Verification in Saccharomyces cerevisiae
Source: PLoS Genet. 2009 Jan 2;5(1):e1000321. doi: 10.1371/journal.pgen.1000321 (PMC2603021; doi:10.1371/journal.pgen.1000321)
Supplement: Table S9 — 3′ UTRs mapped by RACE. A “W” means the gene is on the Watson strand and a “C” means the gene is on the Crick strand. (0.04 MB DOC) [file pgen.1000321.s020.doc]

Table S9. 3' UTRs mapped by RACE. A “W” means the gene is on the Watson strand and a “C” means the gene is on the Crick strand.

| **Gene** | **Strand** | **Gene end** | **UTR**  **end** | **ORF**  **end** | **3' UTR length** |
| --- | --- | --- | --- | --- | --- |
| *ALR2* | C | 3' | 32992 | 33272 | 280 |
| *CDC4* | W | 3' | 118579 | 118478 | 101 |
| *ROG3* | W | 3' | 199188 | 199022 | 166 |
| *SEC4* | W | 3' | 131196 | 130976 | 220 |
| *TUB2* | W | 3' | 57931 | 57708 | 223 |
| *YFR016C* | C | 3' | 176978 | 177034 | 56 |
| *YFR018C* | C | 3' | 182955 | 183119 | 164 |
| *YFR032C* | C | 3' | 221976 | 222078 | 102 |
| *YPT1* | C | 3' | 55256 | 55365 | 109 |
