# Supplementary material for: Non-Coding RNA Prediction and Verification in Saccharomyces cerevisiae
Source: PLoS Genet. 2009 Jan 2;5(1):e1000321. doi: 10.1371/journal.pgen.1000321 (PMC2603021; doi:10.1371/journal.pgen.1000321)
Supplement: Table S10 — Detection of each snoRNA for each window size (step size = 25). The table provides a list of each H/ACA snoRNA and the window sizes at which the snoRNA was detected (X in box). A blank box means that the snoRNA was undetected using the window size specified at the top of the column. A step size of 25 was used for all cases. (0.13 MB DOC) [file pgen.1000321.s021.doc]

Table S10. Detection of each snoRNA for each window size (step size = 25). The table provides a list of each H/ACA snoRNA and the window sizes at which the snoRNA was detected (X in box). A blank box means that the snoRNA was undetected using the window size specified at the top of the column. A step size of 25 was used for all cases.

|  | **Single Window Size (step size = 25)** | | | | | | | | | | | | |
| --- | --- | --- | --- | --- | --- | --- | --- | --- | --- | --- | --- | --- | --- |
| **snoRNA** | **80** | **90** | **100** | **110** | **120** | **130** | **140** | **150** | **160** | **170** | **180** | **190** | **200** |
| *snR30* | X | X | X |  | X | X | X |  | X |  | X | X | X |
| *snR32* |  |  |  |  | X | X | X | X |  |  | X |  |  |
| *snR37* | X | X | X | X | X | X | X | X | X | X | X | X | X |
| *snR44* |  | X |  |  | X | X | X |  |  |  | X | X | X |
| *snR49* | X |  | X | X | X | X | X | X | X | X | X | X | X |
| *snR161* |  | X | X | X | X |  | X |  | X |  | X | X |  |
| *snR42* | X |  | X | X |  | X |  | X | X | X | X | X | X |
| *snR83* | X |  |  | X |  |  |  | X |  |  |  |  |  |
| *snR84* |  |  | X |  |  | X | X |  | X |  | X | X | X |
| *snR191* |  |  |  |  |  |  |  |  |  |  |  |  | X |
| *snR36* | X | X | X | X | X | X | X |  | X | X | X | X | X |
| *snR34* | X |  |  | X | X | X | X |  | X | X |  |  |  |
| *snR46* | X |  | X |  | X |  |  |  |  |  |  |  |  |
| *snR86* | X | X | X |  | X |  |  |  |  |  |  |  |  |
| *snR10* | X |  |  |  |  |  |  |  |  |  |  |  |  |
| *snR3* |  |  |  |  |  |  |  |  |  |  |  |  |  |
| *snR11* |  |  |  |  |  |  |  |  |  |  |  |  |  |
| *snR82* |  |  |  |  | X | X | X |  |  |  |  |  |  |
| *snR81* |  |  |  |  |  |  |  |  | X |  |  |  | X |
| *snR80* |  |  |  |  |  |  |  |  |  |  |  |  |  |
| *snR35* |  |  |  |  |  |  |  | X |  |  |  |  |  |
| *snR8* |  |  |  |  |  |  |  |  |  |  |  |  |  |
| *snR5* |  |  |  |  |  |  |  |  |  |  |  |  |  |
| *snR9* |  |  |  |  |  |  |  |  |  |  |  |  |  |
| *snR31* |  |  |  |  |  |  |  |  |  |  |  |  |  |
| *snR33* |  |  |  |  |  |  |  |  |  |  |  |  |  |
| *snR43* |  |  |  |  |  |  |  |  |  |  |  |  |  |
| *snR85* |  |  |  |  |  |  |  |  |  |  |  |  |  |
| *snR189* |  |  |  |  |  |  |  |  |  |  |  |  |  |
