# Supplementary material for: Non-Coding RNA Prediction and Verification in Saccharomyces cerevisiae
Source: PLoS Genet. 2009 Jan 2;5(1):e1000321. doi: 10.1371/journal.pgen.1000321 (PMC2603021; doi:10.1371/journal.pgen.1000321)
Supplement: Table S12 — All Random and shuffled sequences used in this study. All sequences provided in fasta format. (0.11 MB DOC) [file pgen.1000321.s023.doc]

**Table S12. All Random and shuffled sequences used in this study.** All sequences provided in fasta format.

>Random10

cggttacttgattatttctaactttaagtttactttaatgtttcgc

ttaaatataagcataaacgtcaaacctagaatataactaggcaagt

cgatattaagaatacatgaactcagaactcaactaggaataggagt

gaggtggctagtgcctacatatgtccccaattctgcttaacatgtt

tgtattccaaggaactttttttcaaatgtgaagattaacataatag

atcttgttgtataaccgggtacctttttgtaatcccccgtgcatgc

gactcctaatccaacagtgtgcag

>Random11

tttattgatttgtaaacgttatgccacaaacgcgagtccattaact

aatcttgatcgctatctatgccctagtcagtgtccctaagataatt

ctcttagtactgtggcacaagggttatcacattagctacagagcag

gtcttcaattgacgatatacgtgtaaatgaaccagttttatggtag

tgaagagagtcgctgcaaaaccaggctaccaagaatatacacatat

ctccggattgcgcactcttcaggttggatgactttaactattgaag

accgcaactcctcatgttctatag

>Random12

cagcattccgtgatagatgaattaccgagcacgcatactacgaact

cttccctgtatagggttctatacgttcacatgtacaatattcacgt

gggattatcaaagcgtctgctagacttcggatttttagatggcttg

agtatcgattatgttttcagactttccgcctaatgatacattgttt

atcatatcgttcatatgaagtaattgtaaagtccttactctagcgt

tatattgagctgattttaaatgatatgaaagtaagtgtcaatcata

aagtacgttacttaaaaagaaagc

>Random13

aaaagcgttcgttgagttgactcaggcatcaataactgccgacgga

ctattgatcgcgaaagcaattagatggtctatatggactggtgtta

gagccgggtatggtatatttcagacagataagtacttctatcaact

aagcaacatgtgtataactaggttgaattctgcgcttcacaaatgg

atctaatttcatcgagcttggctaattactacaccgctcgccctat

acggaaatagtagtctctacgaaacataatatcaagggttctctga

tggacacaatgttatcctacttcg

>Random14

tacagagataacaaagaattttaatgacgcaaaaccaagagctcta

atgaggccgggtcactattgggaaagtatttaaatttcgtgttaat

ccatacccaagagactagatctctctggatcaaaaacgataaaggc

tcttggatgctagaaagttcaatttttatgcgggcacctttccaat

catcttaaacaatgtgattgctccattcaaattgaaaactgattgt

ttctggtcattaacgtgattattatagactcataaagggatctctg

tttcccagttgagagcaccgtgta

>Random15

taaatatcacctaggggtgtggaaggctttgtaagatgttcgtga

ttgtccagccacgaactgagtaggctgtctagatcctacccgggg

gagagactttgaagctaactcaaaagagaacataatgttaagagc

gtctaatgtgttttccgaaaaatctcacctttaagagacagtagt

acactattcacacgccaacgcaaggtcattcaatattagtaagta

attcgagtcaacacgaagtacaattcttttctaatcaagagaagg

cgctctatcaaatccacgtgactactaaca

>Random16

aaatcgatgaacacccaaaaaaacagaataaaatgaactatataga

ctgcggtctaaacgaacattaatcaggattctacacttgacacaac

gcatcatcacacgtcattgaatcatgttagttgttgtaattttgta

aacgctagtcaagcaactattgtttcgatttatagaaaaggttgtc

agaaacgtctaccccgaaggatatctataagtccaagcacatgttc

ccaggcactcttgttgcgaattttattaagcctgagacttaaactt

gcaattttcatgcgccctgcagcc

>Random17

ttggtaaggatttcttccttatacgcggacttttcgcagtagttga

tttttagtccgggcggcaatttttctaattggccagttactcggcc

agcctattaacagcaccgatttattaatgattgggaatgaaaatag

tgtcgtaagtacacgtgcaatcagtagtaccttataagtttcacgt

ggtttctttttttcttcacgtcctgaggacggcaagtcatttaatg

ataaccaaattctgggttgcttgggttgtggatggacgagattatt

tgcgcggggactatatgccctagg

>Random18

ctattaatcttaagaccgacaaatgcgtatgcaaagggagaatagaa

acgtccatattatagcgttaaactagaggaggaactacggggatctt

aactaaagataatggatgtctgcctgagctttgaaagtgtgtcccat

gggaataggcccctattatgccttgagcgagctctcctcagccggcc

taaaacatattaaggcgttccgttattaaggttttaaagcatgctat

ttggctgaagtctttaccaatgtaagtcattcccgattaacacgcta

gtccctgaatatagaagt

>Random19

tgtcacaccttccaacaggcagtttaacctgtaaaatatacagtgca

ctaaacataattagatttttatttttgcatatcgtgttgctcgcgca

ctttaacaaacactctcagacgatggaatagtctactgtgtatatgc

tcagttaaagtgtgcaaccttacttcgtgatctgctctggaaaacca

atattttaaaactggctacttacatgaggaatagaagttacacgcga

tgtatatgagctgatctccaagataggaataagtggctcaagtagat

atggtgtaggcacatgtc

>Random1

tatttactaagtctagttgccaacatcaagtctacagcgacacaaaa

tgttcccacatgggaaagatatcaaccagtaaggtggccaggcgaat

agcaagtgcaatacttcggaaggttctgggctcaacagaagatgcac

ccgactgagtatgcgaagatccaggatgaagccggcaatggtggtcg

tgttattcacgatgtcttgtcctgtgtgtgcgtcggtgaccttaaat

cataatacattttcttctaaaactgtgatgcataagggaaacaatta

cacaccctcgtaattata

>Random20

taattgccaacacatttagaactctggcgtagtatctttgttgtcta

tatctgaagaatagtttaaaccaagtattgatcgcgcaaaatagtct

gttttcaagtagaacgattgataataatcgaaccccttaaccactgg

caagttgtagcgctttcgacagagcctaattttcattaatagtccgc

cagtttacaagtacctgtttgtacaatgttgaaccatcagacagtca

gttcccgaagagcactacttccgataaatgattactggtcctttcaa

cgacttttacgctttcgt

>Random2

atgacgcaaactttgagtgggataatgctctttaatggtgaacgaatt

atcgaataattgttcttttcaccagattagtccaatcgctctctgcat

aaagtaccaattgttacaagcattgattcctcagacgctgaaccgtat

tatgagtagtttcgccgtcagcagtttcgaaaatgaacctaccgccta

cattcagtcatcaccctttgaacaggcggatacctataaagacgaggc

tttaggtttcaatggcggaaatgtcgggtagatttatgtcattggtat

taatacacacac

>Random3

tactgaagtccctttataattatcgtcagtgttcgagtctttaaatta

attattttcgtccatgagaatagagaagaaatgcagatcggtaaatca

attcttcgacaatatcggagaaggcaggccatataggatctcccggac

accgaagcagttattgaattctccaagctcgatgatcagccgttgtag

ctatacaaatcaaaaaaaagtgattgcaaaactcagtgagatttctac

ccaactataggatatgtatattgagtgaacttagtacgaaaccaccaa

tatatcgcagcc

>Random4

actagccgcttgttcacgtttttcaattgacttctaacgcttccttaa

catatagcttatagagtgccatcagtcttttgggcttaatttttggcg

ggtgtccaaaccagtaagtacaactgtcacagaatattaacctgtccg

ccttactatgcttggggagcaaaatataggacatttgttcttaacgcg

cacaaataattgcggagtctttcttatactctaactgcctactcttta

gtaatgaaataaagtaatcgtcatccctaagtaaagccccgtgttatg

tctgataggagc

>Random5

gttcgcgaaacgctccatgattagtgtggtacctgacgacaattaca

acgtggagaagctttgtattcaagctagttatttgataagtaggaag

gatagaagtcgccctagcctaaagatgagcacatactccaagtaggac

gcatagactagactatgctactattttatagaccatagaccaatcctt

gccgtgtggatcattatcattaaagtaaatctacttttaacattttgt

atattcatattggggtctactgcaggcacaaaccgcaagtaatatgtg

ccataaagtgtgtt

>Random6

aaatgattcatacaggccttcaggataggactttactgagtagactcc

ttccgttgcctagacgagaacaggactcgggaatccagttacgtccat

tcattctgtcctaaacgcgctacaaaataactacaaagaccggcttat

tgagtgttcaaatatatcaatactcgagggtagttcgctccatcgata

ccatcgatctgttaggatcgttcaactattaattacttctagtcctaa

aagttaaagaacacactgccaaggaacaacccgtgtgaatggatttgt

cggaggccatct

>Random7

accatgtaaatctattaatgattgtcatatgaacaaccatacacttta

cgttttaactactacttgactaagtgtaggacttgcgacttctgctag

tgcgaacctatttacaaccgaacttgactattcatgacttcgaccatt

cacccgaatcaaattcgcgcagtgtagtgcttctttttttgtctgatg

tgttttatgcagtattatctttggcttaagtccgcataaattctagga

ttttctacgaaattgtgtgcccagacaggaacgacacggacgtgtagg

gttttggacaat

>Random8

cggaatattttaagcgggcttcagaatgctaactctagaagattcaaac

gtgttatcgtgatgaaaaagatgccatgcggtgcactgttgcagactgt

gtttgccttttatgtgaacacagacgttaaccatggcactgtcgaaagc

cacttaaacttttatagctagctatttacttcaccccgctaaccgatat

tttcaaggttggatgtatgctacatatgacttcagtggagatcgtttac

atcggtactgccctattggtaattttataatacagcaattctaaacgca

ttgtgt

>Random9

tgtcaccgtttttaattgcccgtgtctggttaacgtataaaggtcagac

ttcaaggctgggtctgtgtacaatcgataagtgttttcgctaatctggt

actaaaggataaatagtgagaactcttagtacaaatcagtacgtctgta

tgaattataaaatacgctacaaagacgtacctagattacatggcctatg

cggatgttcctgctgaagaccaatttctttccatactctgtataggcta

gcgatcagtacttggctccggctatcagtattctcagcgccccatttcg

aactgt

>LSR1scramble Chr 2 reverse complement 681857 to 680683

AAATTCGAATTCGCGTGTACTTGTTCTTTTTTAGGGTAGGTGTTTAATTT

GATTCTTTTCCTGAGAGAAGTGGAAACCAGGATTTGATGTGGGACTAGAT

CAGTCCCTCTTGGGTTGCCTTCTCAGTTTGTTGCGGGAATCCTAGAAAGT

GAAGGCTAGCTAGGTGCTTTGTGCTCGAACAGTTTACACATGGCGTGTAC

CTCTTGTGATCTTTTTGAGCGCGTTTTTTTTTTGTTGAAGATGTGTCCCT

GGAGCCTTTTTTTACGTGCTGATTGTAATTTTTCGCCATGGGGGATCAAG

ATCTTTGTTGCAGTTGTTATAAGAATTGTGGCGCCTAGATTGGACAATCT

TAAGGTGTCTAAAGTCCAGTCTGCAAGGAAAGTTTTTGGCATTTTGCTAG

GTGATTTTGGATTTCGGTCTTCAGGTCGCTACTTAGCACTTTAGAATATT

ATCTTTCGATAGGACACAATGTACTATAATTCTGGGAGTTGCTTTCTTTG

CGGAATTCTTTAATTCTGGCAAAACCTTATCTGAACTAGAGTTTCTTGAC

TCTTTTTCTTGTAATGCTTATGAGGCCCGAGTTCGAATGAAGTGCTTTTT

AAGAACCTAGCAAGACGCGGGCCTAAACTTTCGTTTGGAATTTTCTAATT

AAAGATTGGGTTGAGTTTCGACTTGCTTGCAATTTTTCTTCTCCCATTGT

TCGTTTAGGTGGGGACAGGGTTTATTTTTTGGGGGACGTGTTTTTGCGTT

GTAGCCTTTACTGAGCTTCGGAGTGGGATTTTCATGATAATTAAATTTTG

AATTGTTGAAAAGGCTTCTCTTTTATGAAGCAAGCCCTTCCAGTTGCGGT

TCATGCCTCAGAGTTATTTTCAGCTTTGTGATCTGTGTTTCGTTCTTTCG

GGGTTCTCCGTTTTATTTCGGTGCGTGGAAGGTGCGGGGTAAGAGTGTCT

ATTTTGCTTTTTACGAGATCGAAGGTTCCGTTTTCTATTCGATGTTTGTC

AAAAAATTCTTTTTGGTGAGCCTTTCTCTACTAAGACGAAGGCAGGCCTT

TGAGACGCTTGGGACTTTATCAGTTGGGGGTCTTTGTTTCCGTAACCATG

GGGATCTCTTGTTACTTTTTTCCGCAATTGACATACTTGAGGAAATTATG

CTTCGTGGCCAGATAGGCATAAATT

>NME1scramble Chr 14 (585588 to 585927)

AGCTTTTTTACTGGATTTTATGTTCGGACTCGGACTGTTGAATGTGATCA

AACTCCTTGCAAATAACGTTTTATCTAGGATTGGGGTGAGTATTCTGTGG

TCTTTGTATTCGCCCCAAATGTTCAACCTGCCAATTAAACTGGTTCTCCA

TTGAACCGGTAATTCAGGTTCCTAAAAATACGCTTATTAATGACGAAAAC

TATGCGGTATTTGGATCCGAGAATTTGTCAAACATCTCTCCCATGAACCC

TAAAATTATTTCTTCCATGGGCTGAAGACAGTTTTAACTATATTCACCGT

AGTCAATGCTGAAGTTTAGGAAATAAGGTGATCAGGGCC

>RNA170scramble Chr 13 reverse complement 667455 to 667287

TGAGTTTTCCCGGGCGATCCCAATGCTTGCCTGCATAATGAAATGGACGT

CCGCTAGGCACTAATAGAACTAATTGCAGAATCATCGGTTTCGGTTACCA

TATATCCTGGCAACGGCTCAGGAGTATTACTATCAAGGTGCAGCTTCCAG

CTTTGAATCGATCGATAAT

>RPR1scramble Chr 5 (Coding sequence)

GCTTGCCGTTGGCCCTGTGGATTGTCGCCACGTAATATGAGACGCGATTA

ATTTAGTCTTGTTCCATACCTTCCAAAATACGGTGAGATTGCTTCGGGAA

AGTCAAGATGGGTTAAAGCACCCTTTGTCGGGAAGGTCCAAACGGTGTCG

GGTGGGATGTCGAGATAATACTTCCCGACGTGAGCCCTGTTAAGTTTTCC

GACAACCTGACGTACTAGTCGGAACACTCTAGATTTGGGTGCCAGCTTCA

CTAGGGGGGTTGGGATTAGGCTACGTCCAGCAGTGTGGGCCCAGAGAACT

CGAGCGATCGTTCGACTAAGTTGCATGGTCGAACTTTCTCCATAACCGCC

CGATGATACCATGTGGGGC

>RUF5-1scramble Chr 8 212411 to 213120

ATCTAATATGGAGCAAACCAAAGGTGAACTGTCTTGCTCTGTGGTTCTTT

ACAATAACAAGCTTACATAGATTAATGCTCTGACCTTCATTCCGCGTGCT

CATTGATTAGGATCTGTAAGTAAACTAGTTGATATCAGAAAACATTTGAC

AAGCTAAAAGCTATCTATCAATAAAAAAATAGACAGACATAAAGAACATG

CGTAAGCTTGCATGACCACAGTAGGTTTTTAAATTTTGTTGTGGTGGGAA

CATTTATTTTTTCGCGCAGAGACTCTTTGTTTCTTTCCGATGTTTAGTCT

TCTCCTTTGTGAACTTAAATTTTTATTGGAAACCGATAAATGAGTTAAAT

ATTTTATTAGTGCTTGCAGGTAGGATTTCCACATCAAATTTGTGGAACCA

AATAATTTGTATTTGATAGAAGGCAACTATTTACGACTTCTTGGGGATGA

CATGATCGAATTAGCAAAATTTTAAGCAAATTATTCATCTCTCTTCTGCA

TTCATAAGTAAACCTTATTAGCCTACATTTCGAATGTGTCTTTTAACGAA

TCTTGTTGCAATATATAGTCTGAGTGATTTTCAATCAACACTTTATGCGT

TGAGTAATGCCGCAGAGAACGATATCAACAAGAAATAAGGTTAAGCTTTG

CAGGCATGACACACAATTTATTAGATACGGGATCAATCATAACTGTTATC

CACTGCCCTT

>SCR1scramble Chr 5 441983 to 442504

AAGACACAAGGTGTTTGAGCAGCCCGGGGACAACGTGGTGCCTCGGTTGT

TCCTTAGGCCGCGTCTACTTTGGCTGCGTCCGGGAGTGGTTTGTGCGGGA

TCCCGCCTGACGAAAGGGAATGCTGTGGGTGGGGGTGCGCCATCTACGGG

CCCATTCTTCGAGGGATCTATATCATTGGGGTTGCGTCTATATTCGCGGG

GTGCTCCTGGGGCTAGAAGGGACATATGATAAGCGTGGATCGTTACAGAA

TGAATGTGCAAGTTGATGATTCATCAAGTTTAGTGGGGAGCTCGTCGGGA

GATCTTAGCCTCGTCTGTCAAGCTCGTCCCCACCAGACCGTGGCGTCCTG

GTAGCCGGGTGATTCGGATCCAGCAGGCGGCAGAATTCCTGCATCCCAGC

TTCGTTGCCAGGTCCTAACTTTTGGGCCGTGTTTTGTATGAGCGAAAACT

GGTAATTCACAGAATCCGCTCTAATTTTCTCGCCTCTTTCCCCGTAGCCA

GCCTTGGCGATTTGGGGTCTTT

>SNR14scramble Chr 5 (Coding Sequence)

AATATGAGCCTCTTGTTTAAAAGTATGGATGAGACGTGCCGGGGTTTTCT

TTCATGTAAGTATCGTTTGGATGATATGTCTAGGCGTTTCACCTCGGTCT

TTAACACAATTTTTTTAACCCCACAGATTGATGAATTCAAATGGCTCTCG

GAATACGATT

>SNR19scramble Chr 14 (Coding Sequence)

ATTTGAAGGGAAATTCTCTGTTCTGCCAAGGGAGCTTGGAAGGTTTTCTT

CTTTTATTGCACCCATGGTATAGTGGGATCCAATTTTTTACGATGTACAC

TTGTTATAGGAGCACGCTTAAAATAGCAGTTAAACACTGTTGTTGATTTT

TTTTTGGTTGTTTTACGAGTGGCGATTCTTCTCTTGGCGGCATACTTGAT

TATTTCTTGACTGCAACTTTTTTGATCCACAATGCATTCTTGGACTATGC

GCAGGGATTGATCCGAGATGTTGATTTCATGTTGCGGCTGGATTTTAGAG

TCTCTTTATTTAGAGCGTTGCTTAAAAAATTCCAAAGTTGAGTACATGGG

TCTTAGACTTACATCCATCTTTTCATGGTTGGGATCGAAATCAAGGGACT

TGGGTGACAGGAGAATAGGTTAGTTCCCGCCTTGAGAAAAGAGATGCCTT

TTCGATCTGTGTGGGTCCATTAACTTATACCCTCAATCCCGGAAAAAAAG

AGGTCAAGTTTTCCGTGGCAAGTTTATTATTTTTGTTGAAGAACATGCTT

CTTGGATGGGAGGTACTT

>SNR30scramble Chr 12 198785 to 199385

ACTATTCTCAATGATGCCTGGAAAGCTAATACATAGGGATAGAGACCTGC

CTCGCATGAGTTGATTACGAGGCGCTACATTCTGGCTTGTCGTGGAATGT

TCTTTGGGATCCTTGCCTTTACATAAGCACAAGGCTAAGTCGTGTCTAGG

AGAGAGATACATAATAGGGAGACTCTGCGCTGTCCGAATAGTAGTGGATC

ATCTCAATTTCGGAGGTTATCTAGCCCCTCCTGGGTTTTTATCAGTCACG

ACGGTCGCTCCATTGAGTTTTGTCGAAGGTGCGAAGACTATTACTCACAG

AAGTGAGTCTGGCCGAGTATGAGGTCACTCGCTACTGACTCCCTTTCGTA

AGTTTTTACGTTTCTCACCTGATTTTGGCTGTATCCCTGAACATGGTATG

CAGTATCACAGTGAAAGTGCGAGCATTTTGATATCACGAAGCTCTTACCG

ATTGGCCTCTGACATGTTATGTTCTGTTAGGCTGGCCTTAGATATAAAGC

ATCCGCGCAAAGTAGACACTGCAAATTTCTCGGTGTCATTCAATCTTCAT

CGTGGGTGGGCTTAGGGTGGGCGTCGGTACTTGGTGTGCACTTGCTCCTT

C

>snR49 Chr 14 716122 to 716286

ACGCTCCACAAATTCTTCGAGTTTGTACAAAGTACGTTTTTATTGACATC

TTATACAGAGCCCCTTAAAAGTATGATCTCAGGTCGGCAGTTAATTAGAA

AGTTATGATGATGTCAATTCCACAAAATTGGACCTGTTTTGTATTACTCT

TTTGCAACTTTTTTT

>SNR6scramble Chr 12 (Coding sequence)

GATACGTTTTTACGATGTTTGCGCAATTGTGAGGAAAAATCAGTGAAAGA

GTCAATCAGGACAGTACCCTCGATCCTTTTCGGTATTAATGCAAGACACC

CCAGATTTTTTT

>SNR76scramble Chr 13 297724 to 297832

AGGCTTGGCCAATTTCACCCATCTGGGCTAGATAGCTTGACTGATGACAA

GGTGCACGGAACATGACAAAAGAAAAAAATGGGGATAGGCGCTTCAAATC

CCCGACATG

>SNR7-Lscramble Chr 7 (Coding sequence)

ATTGAAGCCTTTTTCCATAAAATTAGGCTCTTGACTTTTTGTCACTATTT

GCCGCCTCAGACAATAGGAAGCTTCGAAAACAACAAAGTTGGGGTTAATT

CTACAACCGGAACTGCCGCTGTACTTTCTTGGTTATCCAGGTTTGAAGTC

CCAGGAGTGATATAGTTCCTGGTATTTTGAGCGGTGGGCTCAGCACATGG

CACCTGGGGTAGAT

>SNR83scramble Chr 13 626348 to 626653

ACTTCCAAAACCTCATTTTTAAAAAACGGGCCGAAAAAACCTTAATTTGT

TGATGTTTCCCATGAGTTTTTGGGTTTGACCCTTCTTTTTTTTATATCTT

TACATAGGGTCAGCATGTGATTATTGAATACCCTGTTCCTTACTTTCGTT

CGCTTTTTTAGGAACAAGAAACTTTCAGCGGGGGATGGAATAAGCAACTT

TTCAATTTCCTTTTAAAGTATTCGAGGCGATTGGGACTAATTAAATTGCG

GGTCAATAACGTACCCGAAAAAAAGAAGATTCCCAATTCAACCTACACGT

TTATTT

>SRG1scramble Chr 5 (322208 to 322758)

TGCAACTGTCTTATTTGTTTTAGCTAGGCTAGCATATAATTCCAGAACTA

TTTCCTTATACAACCCTCGACTCACGCTATCCCGAAATAGAGGCAATTTA

GGAGCCCCCATATTGGGAGTTACTTAAACAATTGGGAACCACAAAGATAA

TAATAAAAGGGTGCCACGGCAACAATTACTTAATTGGGCGACTCCCTATC

TTGAAAAAAAACGTTGAACTACCGTAAGCGTTGCAAATTAATCAAACGTT

TTTTTGCTTATGGAGGACTGTATGCTTTTGATTAAAATGGTAAAAACTAA

GAACAAAAAAGAGAACAGATCAAAAAGACACTGCAAATCAATAATTCCAG

TGCAAGTAACAATTGTATCATCAGGAAACTATTAAATAATTTGGCTTTGC

TAATAAGACAAAAGCACTTTTTCCATGTGTCACAGGATTTTTTATCTCTG

AGAATTTTAGCATCGGTTTCAATTGTGAGGGTATTACTTTTTCGACTTCA

AATTGATTTCCTAGGGACTTCCAAGAGAGACCAAAAAGTCTCATTGCTGA

T

>TLC1scramble Chr 2 307587 to 308887

ATACATTCCCGTTCAGGATTTTGATCGATTTGGGACTTGAAGCTTTATTT

TTTTTAGTCAATGTGCTTGTTAGAGCCTTTGCAGTGTGTTTTTCACAACT

TGGTTTTTAAATAGCGTCTTGGTTGGTATTAAATAGAAGCGATTTTTTTC

GATTAACTCAGTGCGGGTCTCCCAAAACATTCTTTTCCAATTGCTTGATT

AATCACCTCATACGAGGGTTGCATTAAAAACAAGTTTCAATTAACCAAGT

TTTGAATACTAGAAACTAAACACTGTTGAAATAAACGTGGTTTTGTAACT

AAATCGCTACCAATGATAAGTTATAGGTAACTCTAGTGACCAGAACTAGA

ATTAGCTAACGACGGTATTTCATTGTGTGATAGAAGAAAACGCGTTTCTA

TGTTCATCGCTCTTTGATCAGCAACTTAAATTTGTATTTTGAAAGATAGC

CTAGTTCAGGCAATAACTCGTACGAAGTGTGAGTTCAATCAACGGTTTGA

AATCCCCAAATTTTGTGCATCTTTATTAATCAAATTTAAAAGAGCGTGTC

GTGAAACGTGTTGTTTGTAAGGTAAGTTATTGCGCTTGTTGGACTAGGAG

TAGTGTCATGCTGAACAAGAGAAGTCGCTGTACTTTTTACTTTGGAATCC

TGGAAAGTTTAAATTCTCAAGCTCGAACCAGAACGGGTGAAGCTAGAACC

TACAAGCTTAATTTTTATCATCAATGGTTTGATTTAATTCTGCCCGATTG

TAAATACTTTGTTCTTACAGGGTTTGGAAGACTTTAAGCGCTTTTTTTTT

TATTATATCGCTTGAAAAACGTGAGCTATTTTAAGTCCTAGTTTTCTCTG

AGTAATACTTGGCCTATCACAGAATGAAGTAAGAGACAGTAAAGGCTTAA

TGGAAGTTCATTTTATTCTTTTGCTTTAATATAGGCCTTCTAGATACCAA

AACTTTCATAGGTATGGTTTATTATTTAATTTAATTTATAAAATCAACTG

TAGTGGGATTGCGGTAACACTTCCACATCTGCTTTTAATAAGATCGAGCG

ACCCTCTGATTAATAGAACTTCATGTGCGTGCAATTCAAAGATGCCCGAT

CATTTCCAGATTAGCGTTTAAACACGTTACTTTAATTTGACCATTCATTG

ACTTATCTTTCCCTTGCGTATTTTTGAGAGGCGGGTCGATTGGCGTTACT

TCGTTGTGCGATCTGTAAAGTGAGTTTTTAATGGGATCTAGGTTCCATTG

TAATGCAATTCGGCATTTCCCTTATTGCCTAGATGTCAATGTGATTGTGG

A
